# Semen quality and seminal plasma metabolites in male rabbits (*Oryctolagus cuniculus*) under heat stress

**DOI:** 10.7717/peerj.15112

**Published:** 2023-04-07

**Authors:** Dongwei Huang, Jiawei Cai, Chen Zhang, Rongshuai Jin, Shaocheng Bai, Fan Yao, Haisheng Ding, Bohao Zhao, Yang Chen, Xinsheng Wu, Huiling Zhao

**Affiliations:** 1 Anhui Provincial Key Laboratory of Livestock and Poultry Product Safety Engineering, Institute of Animal Husbandry and Veterinary Medicine, Anhui Academy of Agricultural Sciences, Hefei, Anhui, China; 2 College of Animal Science and Technology, Yangzhou University, Yangzhou, China

**Keywords:** Heat stress, Male rabbit, Semen quality, Seminal plasma, Metabolism

## Abstract

Heat stress causes infertility in male rabbits in summer. This study was conducted to determine the effects of heat stress on semen quality and seminal plasma metabolites of male rabbits. To achieve these objectives, the temperature and humidity index (THI) was used to determine the stress state of male rabbits during different months, thereby the rabbits were divided into heat stress and no heat stress groups. The quality of the semen and the biochemical indices of seminal plasma were then analyzed. Next the plasma metabolites of rabbits in both groups were evaluated using the ultra-high performance liquid chromatography-mass spectroscopy (UPLC-MS)/MS technique. Our results showed that the THI value of the rabbit housing in May was 20.94 (no heat stress). The THI value of the housing in August was 29.10 (heat stress group, *n* = 10). Compared with the non-heat stress group, the sperm motility, density, and pH in the heat stress group (*n* = 10) were significantly decreased (*P* < 0.01); the semen volume decreased significantly (*P* < 0.05); and the sperm malformation rate increased significantly (*P* < 0.01). The number of grade A sperm significantly decreased, while the numbers of B and C grade sperm significantly increased (*P* < 0.01). The total sperm output (TSO), total motile sperm (TMS), and total functional sperm fraction (TFSF) decreased significantly (*P* < 0.01). Heat stress protein 70 (HSP70) and acid phosphatase (ACP) in the seminal plasma of rabbits in the heat stress group (*n* = 20) were significantly increased (*P* < 0.01). Seminal plasma testosterone (T), α-glucosidase (α-Glu), and fructose decreased significantly (*P* < 0.01). The concentrations of Mg^2+^ (*P* < 0.05), Na^+^ (*P* < 0.01), and K^+^ (*P* < 0.01) in metal ions were significantly decreased. These findings indicated that heat stress severely affected the quality of the male rabbit semen. Furthermore, UPLC-MS/MS technology was used to analyze the seminal plasma samples of rabbits in the heat stress group and non-heat stress group (*n* = 9 for each group). In total, 346 metabolites were identified, with variable importance in project (VIP) > 1.0, fold change (FC) > 1.5 or < 0.667, and *P* < 0.05 as the threshold. A total of 71 differential metabolites were matched, including stearic acid, betaine, arachidonic acid, L-malic acid, and indole. The Kyoto Encyclopedia of Genes and Genomes (KEGG) enrichment analysis of differential metabolites revealed 51 metabolic pathways, including synthesis and degradation of ketones, serine and threonine metabolism, tryptophan metabolism, and the citric acid cycle. Our study has shown that the sperm motility, sperm pH value, and sperm density of male rabbits decreased significantly under heat stress, and the sperm malformation rate increased significantly. Furthermore, the quality of semen was shown to deteriorate and the energy metabolism pathway was disturbed. These findings provide a theoretical reference for alleviating the adaptive heat stress in male rabbits.

## Introduction

Heat stress (HS) refers to the sum of a series of non-specific defense responses of the animal body to the elevated environmental temperature. Due to the increase in the average global temperature, the frequency of extremely high temperatures during summer is increasing; climate change causes heat stress to become more frequent and severe (Dunshea, 2021). It has been reported that increased ambient temperature affects male reproduction. Bulls exposed to high ambient temperature (40 °C) have reduced sperm quality, *i.e*., decreased percentage of motile sperm cells and increased percentage of abnormal sperm cells. It takes approximately 8 weeks for the semen quality to return to normal (Shahat, Rizzoto & Kastelic, 2020). Similarly, in a study where the rams were exposed to a high ambient temperature (40 °C) for 5 h, the scrotum sweat glands were activated. The temperature of the scrotum increased and its difference to body temperature decreased under extremely high temperatures, thereby adversely affecting the seminal plasma enzyme activity and testis in all aspects (Shahat, Rizzoto & Kastelic, 2020). The percentage of abnormal sperm cells has been shown to increase after heat stress stimulation in males (Ross et al., 2015). Under high environmental temperature, rabbits tend to adjust their physiological responses on the hypothalamic-pituitary-adrenal (HPA) axis. The hypothalamic adrenocorticotropic hormone releasing hormone (CRH) and adrenocorticotropic hormone (ACTH) increase significantly and lead to the synthesis and secretion of glucocorticoid (Harbuz et al., 1995). Conversely, the synthesis of the hypothalamic thyroid stimulating hormone (TSH) and thyroid hormone is significantly reduced, thus causing the metabolic rate and heat production of rabbits to lower and infertility issues in the summer (Garcia & Argente, 2017). The result is that the testis atrophy, reducing sperm count, or producing dead sperm or no sperm, at all. These effects decrease the sexual functions of male rabbits and seriously affect the reproductive performance (van Wettere et al., 2021).

The seminal plasma is composed of prostatic fluid, epididymis fluid, seminal vesicle fluid, and a small amount of fluid secreted by the urethral bulb gland. It contains a large amount of water, fructose, protein, enzymes, hormones, and a variety of substances that provide energy and nutrition to the sperm (Kasimanickam, Buhr & Kasimanickam, 2019). The exposure of an animal to a high ambient temperature adversely affects its physiological and reproductive functions (Hansen, 2009; Takahashi, 2012). Mid-year high-temperature months or experimental exposure to heat stress reduce pregnancy rates, fertility, and embryonic development (Sabés-Alsina et al., 2016). Under heat stress, the α-glucosidase and lactate dehydrogenase isoenzyme C_4_ content in the seminal plasma decreased significantly, resulting in changes in semen quality. Spermatozoa were activated in a significant amount of inorganic ions such as K, Na, and pH especially in fish (Dziewulska & Pilarska, 2018; Hamamah & Gatti, 1998). Ca stimulus could increase the early occurrence of the acrosome reaction leading to infertility in mammals in the summer (Murase et al., 2007). However, other literature suggests that declining semen quality under heat-stress perhaps may have no relationship with the seminal plasma ions (Li et al., 2015). Many studies have been conducted on the influence of heat stress on the quality of spermatozoa. However, not many have been carried out on the composition of the seminal plasma. Changes in the quality of the semen and metabolites in “summer infertility” male rabbits are required to be systematically studied.

Based on the results of previous studies, we hypothesized that heat stress would result in lower fertility in male rabbits. However, it is not well known about the specific changes of semen quality and seminal plasma metabolites under heat stress. Therefore, the objective of this study was to investigate the effect of heat stress on semen quality and seminal plasma metabolites in male rabbits. The findings of this study will provide a theoretical basis for further understanding the mechanism of heat stress on the declining semen quality in male rabbits. This in turn should help us develop mitigation techniques of heat stress in male animals, and solve the accompanying problems of summer infertility.

## Experimental materials and methods

### Experimental animals and design

This study was conducted at the Dongfang Rabbit Farm in Pizhou, Jiangsu Province, China (latitude 34.441208°N, longitude 117.986541°E, and altitude 28.8 m). Eight-month-old New Zealand White male rabbits (*n* = 20) were included in the study. All experimental male rabbits were raised in the same rabbit house by the same breeder in accordance with the conventional feeding and management mode of large-scale rabbit farms to maintain the same environmental conditions. Each male rabbit was raised in a single cage measuring 50 cm × 60 cm × 40 cm, and a nipple drinker was provided for clean water. The rabbits were fed in the morning and afternoon, every day. The rabbits were fed with commercial pellets (crude protein 16%, crude fiber 18%, crude ash 12%, calcium 1%, phosphorus 0.4%, lysine 0.6%, and H_2_O 14%), containing corn, wheat bran, soybean meal, alfalfa hay powder, L-lysine, sodium chloride, calcium hydrogen phosphate, vitamin A, and vitamin D1. The animal experiments were conducted in strict accordance with protocols approved by the Animal Care Advisory Committee of the Anhui Academy of Agricultural Sciences (AAAS2022-17) under the “Guidelines for Experimental Animals” of the Ministry of Science and Technology (Beijing, China). No death or disease in the rabbits were observed during the experimental period.

The male rabbits were acclimated to the environment for 7 days prior to the start of the experiment. The rabbits were used for semen collection prior to artificial insemination. Semen was collected using an artificial vagina, a device which includes a cylindrical shell with three openings, an elastic sheath as inner tube, two rubber bands, and an attached semen collecting cup. Male rabbits were given regular contact with female rabbits to improve their sexual desire. During semen collection, a rabbit skin was fixed on the right arm of the ejaculator, the right hand held the false vagina, the gas nozzle was downward, and the finger was stuck. At the beginning of the training period, the assistant held an estrous rabbit on the rabbit skin-covered ejaculation arm. When the male rabbit climbed on the back of the female rabbit, the assistant slowly removed the female rabbit. The male rabbit fell on the rabbit skin, and the penis was inserted into the lubricated false vagina. Ejaculation was complete when the back of male rabbit curled up, the ‘cooing’ sound was made, and the male rabbit slipped to one side. The sperm extractor immediately erected the false vagina upward, deflated the sperm collection bottle, and plugged the disinfected cork. Sperm collection was performed thrice for each rabbit for subsequent experiments, with 2 to 3 day interval between each sperm collection. The semen collection dates were May 1 to 20 and August 1 to 20, 2021. The THI value was calculated; May was defined as NH for the non-heat stress group, and August as the H for the heat stress group. The semen collection occurred from 8:30 a.m. to 9:30 a.m., with 20 male rabbits participating in each session. The first sperm collection was used to detect the quality of the semen. The two remaining semen collections were centrifuged, and the supernatant and the bottom sperm were sucked out, respectively, and transferred to the enzyme-free cryo storage tubes. The samples were frozen in liquid nitrogen, and then transferred to –80 °C for storage.

After the last sperm collection, the humane endpoint for study was the death of 20 rabbits with a final live weight of approximately 4 kg each. The rabbits were stunned and exsanguinated *via* their carotid arteries and jugular veins. The testicular tissue of male rabbits was collected, washed with 0.9% saline, fixed with 4% paraformaldehyde, and used to produce tissue sections.

### Determination of temperature and humidity index (THI)

Throughout the duration of the experiment, the ambient temperature and relative humidity were recorded thrice a day with a hygrograph. The hygrograph was located in the rabbit housing to ensure that it was not exposed to light and rain. The temperature and humidity at 8:00, 14:00, and 20:00 were measured each day. The daily ambient temperature and relative humidity were used to calculate the average daily temperature and humidity.

The temperature and humidity index (THI) was calculated using the following equation:

THI = db °C − [(0.31 − 0.31 RH) × (db °C − 14.4)]

db °C = dry bulb temperature and RH = relative humidity percentage/100. The obtained values were next classified as follows: <27.8 is no heat stress, 27.8–28.9 is moderate heat stress, 28.9–30.0 is severe heat stress, and 30.0 and above are very severe heat stress (Marai, Ayyat & Abd El-Monem, 2001).

### Semen quality test of male rabbits

#### Sperm motility test

The sperm motility was analysed using the sperm analysis system MX7.5 of Tsinghua Tongfang, and sperm cells were divided into four grades of A, B, C, and D according to the movement form of sperm cells. Among them, grade A sperm advanced rapidly along the straight line, grade B sperm advanced slowly along the straight line, grade C sperm moved *in situ*, and grade D sperm cells were immobile. Sperm density, progressive motility, and viability were assessed in accordance with the WHO Human Semen Test and Processing Experimental Manual (Cooper et al., 2010).

#### Analysis of sperm malformation rate

Eosin staining was used to count 200 sperm cells to evaluate the number of deformed sperm using the following formula: sperm malformation rate = malformed sperm count/200 × 100%.

#### Detection of semen collection

After semen collection, the gel in the semen was removed. The semen volume was read out and recorded according to the scale on the semen collection cup.

#### Semen pH measure

A total of 5 μL of semen was taken and its pH was measured using a pH test paper. The pH value was obtained by comparing it with the standard colorimetric card. The pH value was measured thrice and the average value was calculated.

#### Analysis of TSO, TMS, and TFSF

Total sperm output (TSO), total motile sperm (TMS), and total functional sperm fraction (TFSF) were calculated using the percentage of ejaculation, sperm density, motile sperm, living sperm cells, and normal sperm cells (Yousef, 2005).

TSO = semen volume (mL) × sperm density (× 10^6^/mL)

TMS = percentage of motile sperm × total sperm output (× 10^6^/ejaculation )

TFSF = total sperm output (× 10^6^/ejaculation ) × percentage of progressive motility × percentage of normal sperm

### Determination of biochemical components in seminal plasma

The collected semen samples were placed in an icebox and thawed slowly. The seminal palsma biochemical index levels were determined using Heat shock protein 70 (HSP70), testosterone (T), alkaline phosphatase (ALP), acid phosphatase (ACP), α-glucosidase (α-Glu), and lactate dehydrogenase (LDH) ELISA Kit (Enzyme-linked, Shanghai, China), Fructose, potassium ion (K^+^), magnesium ion (Mg^2+^), sodium (Na^+^) in the seminal plasma were detected using fructose, potassium ion (K^+^) detection kit., a magnesium ion (Mg^2+^), and a sodium ion (Na^+^) detection kits (Jiancheng, Nanjing, China), respectively. All tests were conducted following the manufacturer’s recommendations.

### Extraction of sample metabolites

To extract the metabolites, 100 μL of seminal plasma samples were placed in EP tubes, and 400 μL of 80% methanol aqueous solution was added. The samples were subjected to vortex oscillation, water bath treatment for 5 min at 15,000 rpm, and were then centrifuged at 4 °C for 20 min. A certain volume of supernatant was diluted with mass spectrometry water until the methanol content was 53%. It was then centrifuged at 15,000 rpm at 4 °C for 20 min by high-speed freezing centrifuge (Scilogex, Rocky Hill, CT, USA). Lastly, the supernatant was collected for analysis (Batzias, Theodosiadou & Delis, 2004; Qiao et al., 2017).

### Preparation of QC sample and blank sample

The data quality control (QC) was composed of 18 experimental samples (nine for each group) of heat stress group and non-heat stress group in equal volume. The first three QCs were used to monitor the instrument state before injection using the equilibrium chromatography–mass spectrometry system (Thermo Fisher Scientific, Waltham, MA, USA). The next three QCs were scanned in segments, together with the secondary spectra obtained from the experimental samples for the qualitative analysis of metabolites. The QC in the sample test was used to evaluate the stability of the system during the experiment and the the data were analyzed quality control. QC1, QC2, and QC3 denoted repetitive operations. In a blank sample, a 53% methanol–aqueous solution replaced the experimental sample. The pre-treatment process was the same as that of the experimental samples.

### Instrument parameters

Vanquish UHPLC from Thermo Fisher was used for liquid chromatography. The Hypesil Gold column was used (100 × 2.1 mm, 1.9 μm); the column temperature was 40 °C, and the flow rate was 0.2 mL/min. Under positive ion mode, mobile phase A was 0.1% formic acid, and mobile phase B was methanol. Under negative ion mode, mobile phase A was 5 mM ammonium acetate, pH 9.0, and mobile phase B was methanol. The chromatographic gradient elution procedure is shown in Table 1.

**Table 1:** Elution conditions of mobile phase gradient.

Time (min)	Flow velocity (mL/min)	Mobile phase A (%)	Mobile phase B (%)
0	0.2	98	2
1.5	0.2	98	2
3	0.2	0	85
10	0.2	0	100
10.1	0.2	98	2

MS conditions were scanning range of m/z 100–1,500; the eSI source was set as follows: spray voltage: 3.2 kV; sheath gas flow rate was 40 arb; aux gas flow rate was 10 arb; the capillary temperature was 320 °C. Polarity was positive; negative; the MS/MS secondary scan was a data-dependent scan.

### Data preprocessing and metabolite identification

The CD 3.1 database search software was used to import the offline data file and process it. The retention time, mass charge ratio, and other parameters of each metabolite were simply screened. The retention time deviation was set to 0.2 min and the mass deviation was set to 5 ppm. The peak alignment of different samples was performed for more accurate identification. Afterward, information such as mass deviation of 5 ppm, signal strength deviation of 30%, and minimum signal strength was set to extract the peaks. In addition, the area of the peak was quantified, and then the target ions were integrated. Finally, the molecular formula was predicted using molecular ion peaks and fragment ions, and compared with the mzCloud (https://www.mzcloud.org/; mzVault, and Masslist databases). The background ions were removed by blank sample, and the original quantitative results were standardized to obtain the identification and relative quantitative results of metabolites. The data were processed using the Linux operating system (CentOS version 6.6) and R software in Python.

### Data analysis

The KEGG database (https://www.genome.jp/kegg/pathway.html) and the HMDB database (https://hmdb.ca/metabolites) were used to identify the metabolites. The metaX software (Wen et al., 2017) was used to convert the data, and principal component analysis (PCA) and partial least squares discriminant analysis (PLS-DA) were performed. The screening of differential metabolites was largely based on the results of PLS-DA. The variable importance in project (VIP) of the PLS-DA model was analyzed from the obtained multivariate analysis. The VIP value represents the contribution of metabolites to grouping, and the metabolites with different varieties or tissues can be preliminarily screened. In univariate analysis, the statistical significance (*P*-value) of metabolites between the two groups was calculated using a *t*-test, and the fold change (FC) value of the metabolites between the two groups was calculated. The default criteria for screening differential metabolites were VIP > 1.0, FC > 1.5 or < 0.667, and *P* < 0.05.

## Results

### Changes in temperature, humidity, and THI index of rabbit farm in different months

**Figure 1:**
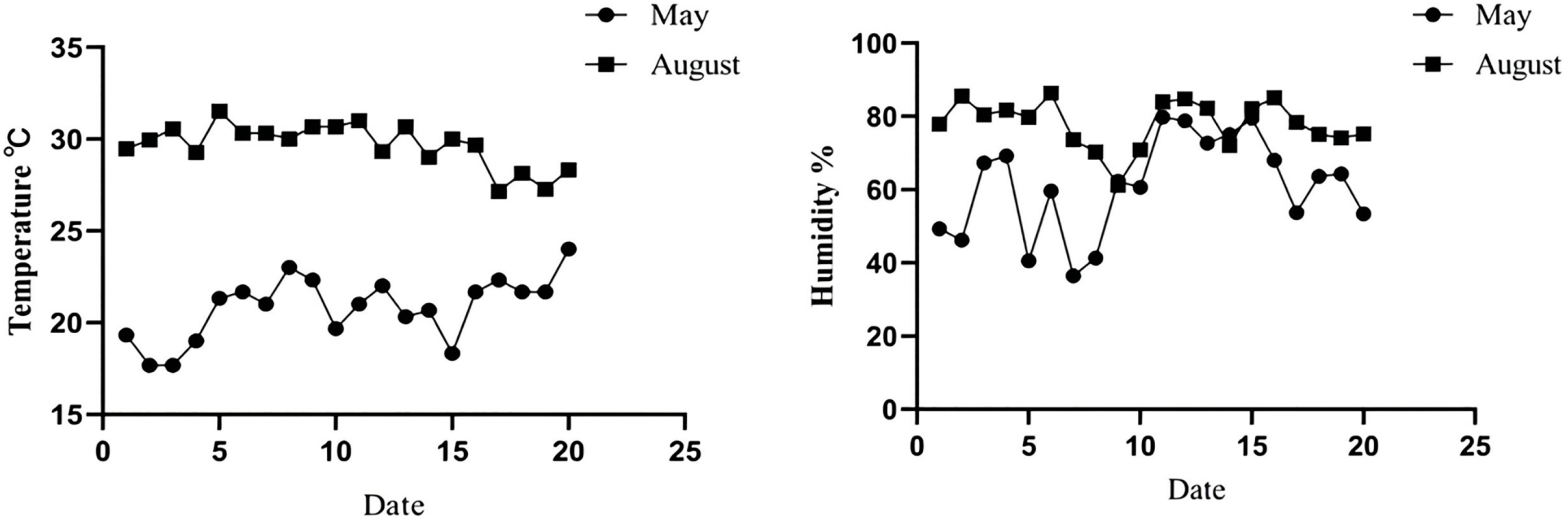
Temperature and humidity changes in rabbit house in May and August.

The temperature of the New Zealand white rabbit housing from May to August was monitored. The results showed that the average temperature, humidity, and THI of the rabbit housing in May were 22.04 ± 2.52 °C, 61.82 ± 13.35%, and 20.94 ± 2.22, respectively, which was defined as the non-heat stress group. In August, the average temperature, average humidity, and THI of the rabbit houses were 30.15 ± 0.67 °C, 78.62 ± 6.88%, and 29.10 ± 2.52, respectively, which were defined as the heat stress group, as shown in Fig. 1 and Table 2.

**Table 2:** Temperature, humidity, and THI index changes in rabbit house from May to August.

Time	Temperature (°C)	Humidity (%)	THI
May (31 days)	22.04 ± 2.52	61.82 ± 13.35	20.94 ± 2.22
June (30 days)	26.10 ± 2.47	69.93 ± 15.47	24.92 ± 1.95
July (31 days)	27.66 ± 2.24	80.10 ± 4.69	26.84 ± 2.12
August (31 days)	30.15 ± 0.67	78.62 ± 6.88	29.10 ± 2.52

**Note:**

The data were shown as the means ± SD.

### Effect of heat stress on sperm motility in male rabbits

According to the state of sperm movement, the sperm was classified into fast straight forward (A), slow straight forward (B), non-forward movement (C), and non-movement (D). As shown in Table 3, compared with the non-heat stress group, the number of grade A sperm in the heat stress group was significantly decreased (*P* < 0.01). The number of grade B and C sperm cells were significantly increased (*P* < 0.01). No significant difference in grade D sperm count was observed between the two groups (*P* = 0.112).

**Table 3:** Effects of heat stress on sperm motility of male rabbits (*n* = 10).

Semen indicators	Non-heat stress group	Heat stress group	*P* value
Grade A sperm count (%)	79.60 ± 6.15**	39.16 ± 8.52	<0.001
Grade B sperm count (%)	7.31 ± 2.29**	32.00 ± 6.55	<0.001
Grade C sperm count (%)	2.63 ± 1.38**	16.46 ± 4.10	<0.001
Grade D sperm count (%)	10.59 ± 3.08	13.91 ± 3.23	0.112

**Note:**

The same line (**) represents *P* < 0.01, indicating extremely significant difference. Grade A, fast straight forward; grade B, slow straight forward; grade C, non-forward movement; grade D, and non-movement. The data were shown as the means ± SD.

### Changes in semen quality of male rabbits under heat stress

Sperm motility, semen pH, sperm malformation rate, semen volume, and semen density were calculated. The results showed that sperm motility and semen pH were significantly decreased in the heat stress group (*P* < 0.01), the sperm malformation rate was significantly increased (*P* < 0.01), and the semen volume decreased significantly (*P* < 0.05). The sperm density was significantly decreased (*P* < 0.01). TSO, TMS, and TFSF were calculated to find that TSO, TMS, and TFSF in the heat stress group were significantly lower than those in the non-heat stress group (*P* < 0.01). The results are shown in Table 4.

**Table 4:** Effects of heat stress on semen quality parameters of male rabbits (*n* = 10).

Semen indicators	Non-heat stress group	Heat stress group	*P* value
Sperm motility (%)	86.81 ± 7.13**	71.20 ± 9.75	<0.001
Sperm motility rate (%)	88.55 ± 5.55**	80.16 ± 8.94	0.008
Sperm density (10^8^/mL)	5.03 ± 0.88**	3.80 ± 0.82	0.005
Semen pH	7.27 ± 0.12**	7.02 ± 0.11	<0.001
Sperm malformations (%)	12.56 ± 1.72**	16.11 ± 2.26	<0.001
Amount of sperm (mL)	0.96 ± 0.34*	0.77 ± 0.29	0.014
Total sperm output (TSO) (10^8^/the amount of ejaculation at one time)	4.82 ± 0.30**	2.92 ± 0.24	0.011
Total motile sperm (TMS) (10^8^/the amount of ejaculation at one time)	4.24 ± 0.02**	2.34 ± 0.02	0.006
Total functional sperm fraction (TFSF) (10^8^/the amount of ejaculation at one time)	3.61 ± 0.16**	1.72 ± 0.33	0.002

**Note:**

The same line (**) represents *P* < 0.01, indicating extremely significant difference; (*) represents *P* < 0.05, indicating significant difference. The data were shown as the means ± SD.

### Effect of heat stress on biochemical indexes of seminal plasma in male rabbits

The biochemical indexes of seminal plasma in male rabbits were analyzed; the results are shown in Table 5. Compared with the non-heat stress group, the heat stress protein 70 (HSP70) and acid phosphatase (ACP) in the heat stress group increased significantly (*P* < 0.01), and the testosterone (T), α-glucosidase (α-Glu), and fructose (fructose) in the seminal plasma decreased significantly (*P* < 0.01). Mg^2+^, Na^+^, and K^+^ in metal ions decreased significantly (*P* < 0.05 for Mg^2+^, *P* < 0.01 for Na^+^ and K^+^); no significant difference was observed in alkaline phosphatase (*P* = 0.148) and lactate dehydrogenase isoenzyme (*P* = 0.775) between the two groups.

**Table 5:** Effects of heat stress on seminal plasma biochemical indices of male rabbits (*n* = 20).

Seminal plasma biochemical indexes	Non-heat stress group	Heat stress group	*P* value
HSP70, pg/mL	1,255.38 ± 245.28**	1,523.25 ± 108.99	<0.001
Fructose, mg/mL	1.46 ± 0.10**	1.30 ± 0.06	<0.001
Testosterone, pg/mL	341.49 ± 40.60**	266.71 ± 40.97	<0.001
ALP, ng/mL	162.93 ± 23.70	172.08 ± 25.19	0.148
ACP, ng/mL	56.02 ± 4.19**	72.36 ± 6.26	<0.001
α-Glu, μg/mL	25.94 ± 4.46**	22.33 ± 4.21	<0.001
LDH, ng/mL	59.05 ± 10.09	58.49 ± 9.15	0.775
Na^+^, mmol/L	67.82 ± 21.09**	47.92 ± 15.99	0.002
K^+^, mmol/L	8.30 ± 0.24**	7.89 ± 0.43	<0.001
Mg^2+^, mmol/L	2.32 ± 0.13*	2.22 ± 0.14	0.039

**Note:**

The same line (**) represents *P* < 0.01, indicating extremely significant difference; (*) represents *P* < 0.05, indicating significant difference. The data were shown as the means ± SD. HSP70, Heat shock protein 70; ALP, alkaline phosphatase; ACP, acid phosphatase; α-Glu, α-glucosidase; LDH, lactate dehydrogenase; Na^+^, sodium ion; K^+^, potassium ion; Mg^2+^, magnesium ion.

**Figure 2:**
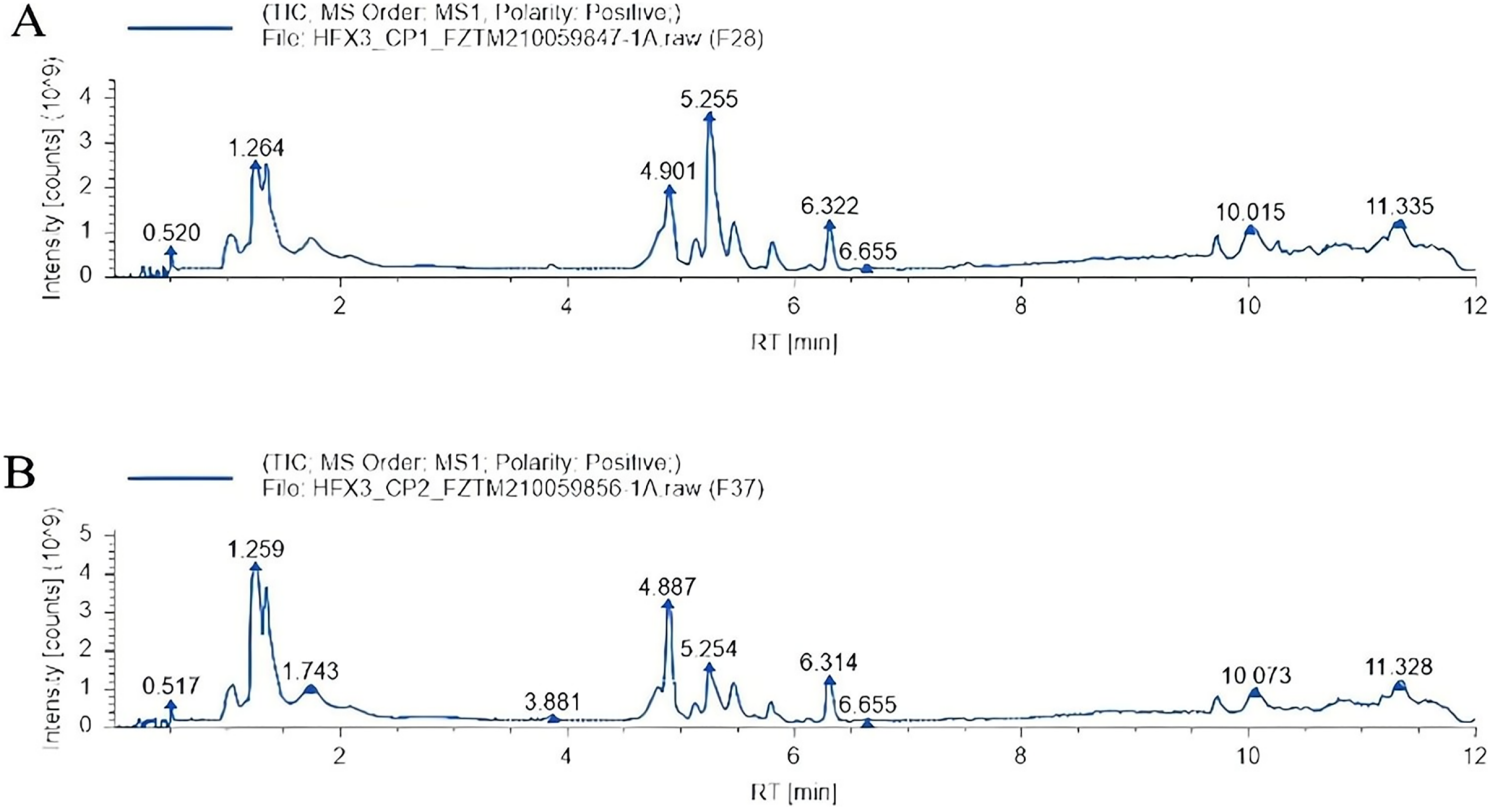
Total ion chromatography. (A) Heat stress group. (B) Non-heat stress.

Total ion chromatography (TIC): After adding the intensity of all ions in the mass spectrum at each time point, the spectrum was continuously depicted and which contained the ionic strength and the retention time of each component metabolite in chromatography. The total ion flow patterns of samples in the heat stress group and non-heat stress group are shown in Fig. 2. A total of 346 metabolites were identified by screening the data.

### Data quality control analysis and principal component analysis

**Figure 3:**
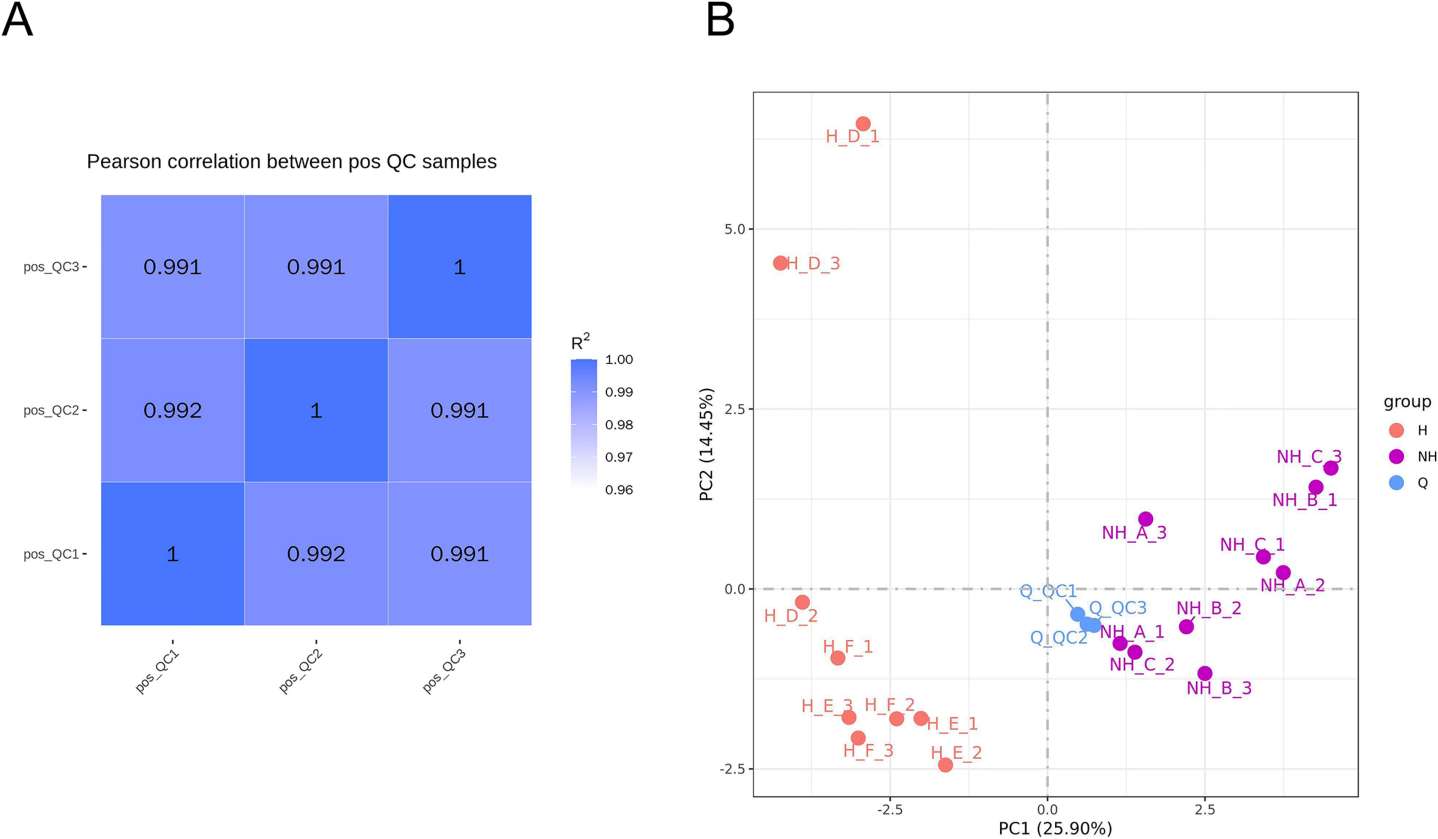
Data quality control analysis and principal component analysis. (A) Correlation analysis of QC samples. (B) PCA analysis of metabolites. NH is the group without heat stress; H is the heat stress group; Q is the sample group.

The relative quantitative values of metabolites were used to calculate Pearson’s correlation coefficients between QC samples (Rao, Sui & Zhang, 2016). The calculation results are shown in Fig. 3A. QC sample correlation was close to 1, indicating that the stability of the whole detection was good. The PCA analysis of peaks extracted from all experimental samples and QC samples was performed, and the results are shown in Fig. 3B. The QC samples were collected together, indicating that the difference in QC samples was small. This shows that the whole method had good stability and high data quality. There was a significant difference in data between the heat stress group and the non-heat stress group, indicating that the seminal plasma metabolism of male rabbits was significantly changed after heat stress.

### Selection of differential metabolites

**Figure 4:**
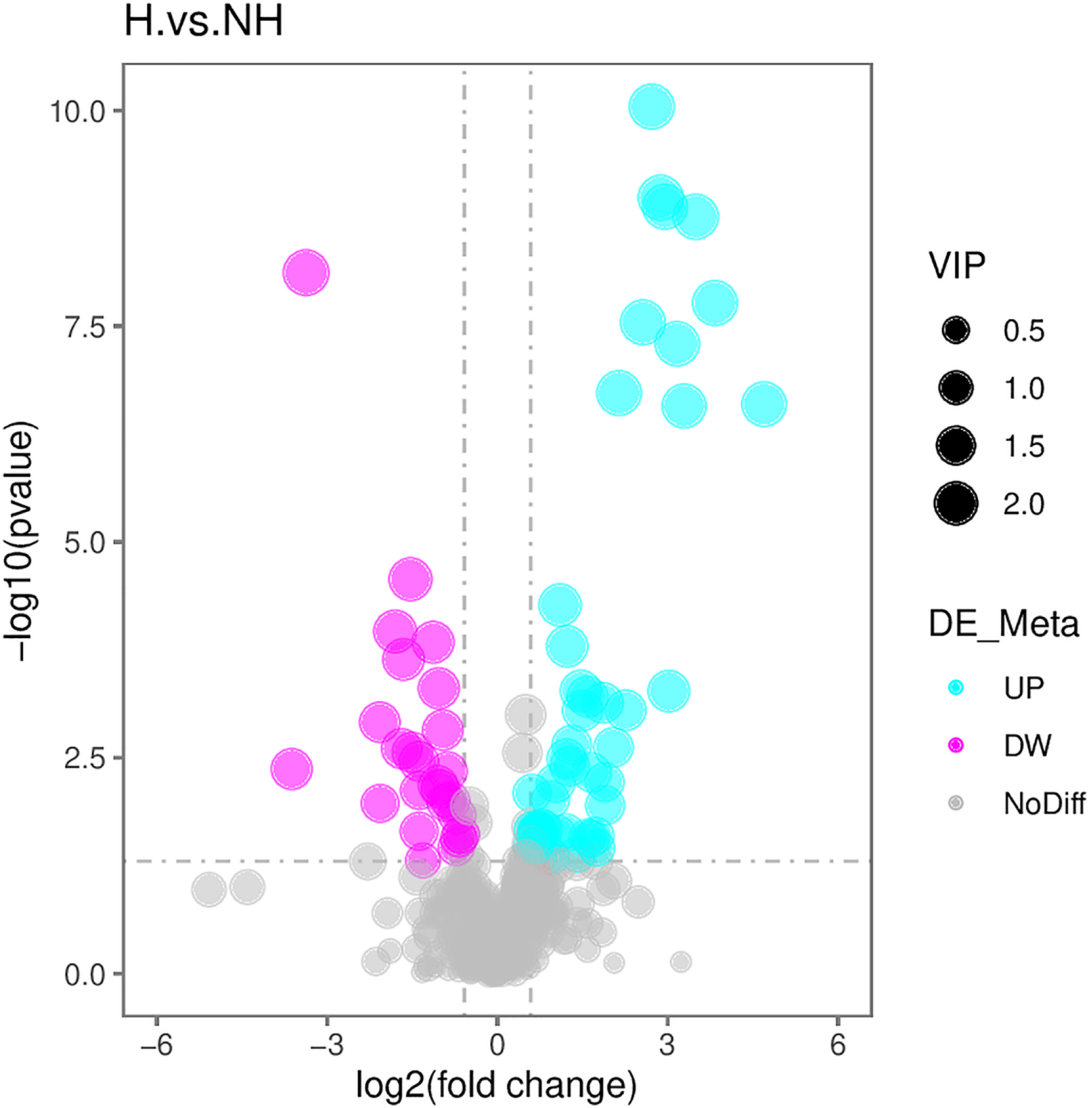
Volcanic maps of differential metabolites. The abscissa is the difference between multiple changes of metabolites in different groups (log2FoldChange), and the ordinate is the different significance level (–log10P-value). Each point represents a metabolite, red dots represent the upregulated metabolites, green dots represent the downregulated metabolites, and the dot size represents the VIP value.

With reference to the VIP, FC, and *P*-value, the identified metabolites were screened. A total of 71 differential metabolites were screened (Table S1). The R program package ggplot2 software was used to draw the volcanic map of differential metabolites. The volcanic map depicted the overall distribution of differential metabolites (Fig. 4): there were 48 upregulated metabolites and 23 downregulated metabolites.

### Enrichment results from metabolic pathways of differential metabolites

**Figure 5:**
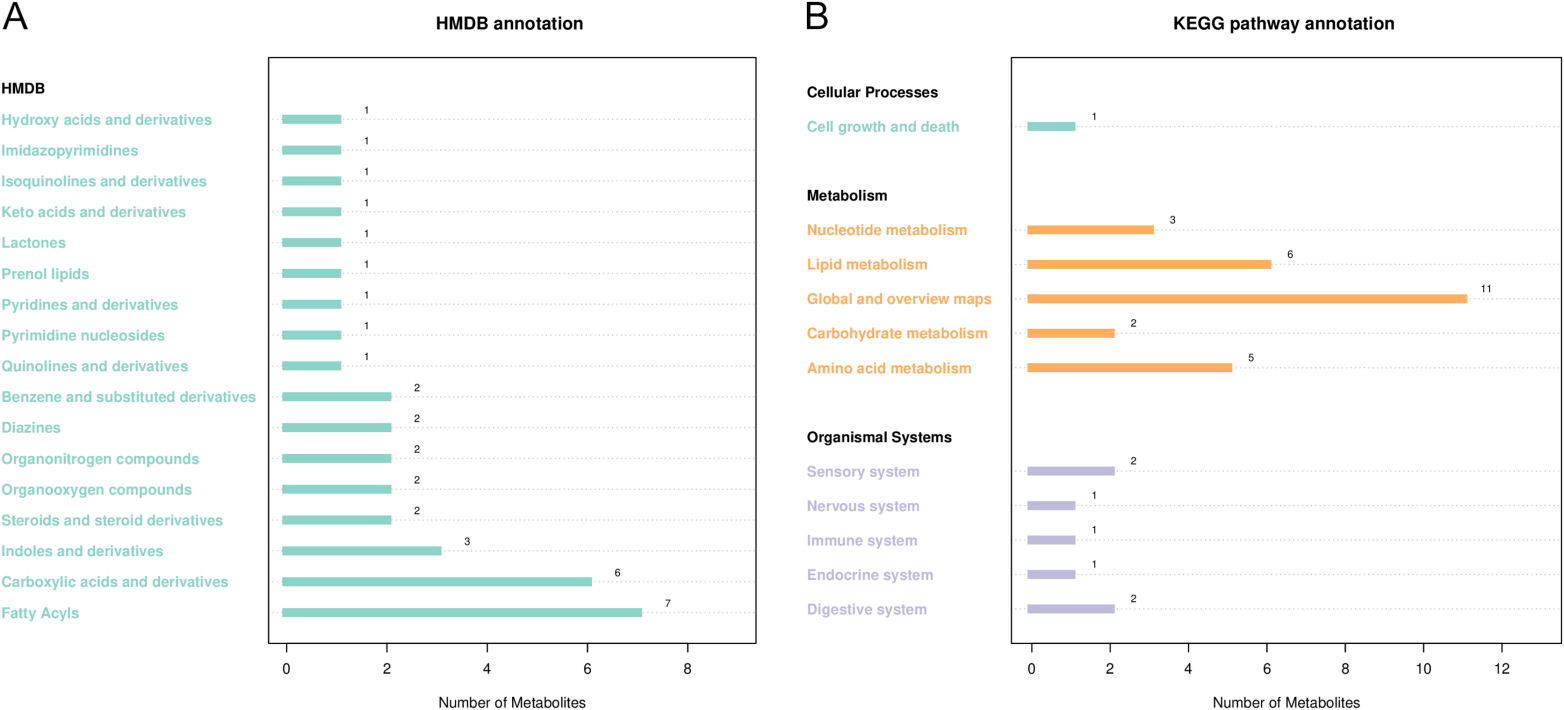
Notes on the classification of differential metabolic pathways. (A) KEGG pathway annotation, the abscissa represents the number of metabolites, and the ordinate represents the annotated KEGG pathway. The number of metabolites labeled by each secondary classification of the pathway is shown in the figure. (B) HMDB access notes, the abscissa is the number of metabolites and the ordinate is the labeled HMDB classification. The figure shows the number of metabolites annotated in the secondary classification (SuperClass) of HMDB.

HMDB and KEGG were used to annotate differential metabolites (Fig. 5). As shown in Fig. 5A, HMDB annotated 35 differential metabolites, including carboxylic acids and their derivatives, aliphatic acyl, indoles, and their derivatives. KEGG showed enrichment for 51 metabolic pathways, including ketone body synthesis and degradation, serine and threonine metabolism, tryptophan metabolism, citric acid cycle, and other metabolic pathways (Fig. 5B).

## Discussion

The reproduction and productivity of domestic rabbits were affected by environmental factors such as temperature, relative humidity, and sunlight exposure (El-Ratel et al., 2021; Farghly et al., 2020). Environmental conditions with severe heat stress can reduce the quality of semen and the ability to fertilize male rabbits, resulting in significant economic losses (El-Desoky et al., 2017). Therefore, maintaining the reproductive performance of male rabbits reared at high temperatures is of great significance to the overall efficiency of the rabbit farm (Hashem, Abd El-Hady & Hassan, 2013).

Under heat stress, the semen quality of male animals were significantly affected. In this study, temperature and humidity in the rabbit house were calculated. In the months of May, June and July the animals are not in heat stress (THI < 27.8), and the THI index in August was 29.10, indicating that there was a severe heat stress environment. In addition, the heat stress protein HSP70 was used as an indicator to determine whether animals were under heat stress. It was found that HSP70 in the seminal plasma increased significantly, and male rabbits were under heat stress in August. Heat stress changes the testicular activity and body weight of animals, causes dysfunction in the body, and decreases testosterone concentration, sperm quality, and quantity (including reducing sperm motility, abnormal sperm morphology, *etc*.) (Hosny et al., 2020). The sperm motility, viability, pH, volume, and sperm density of male rabbits under thermal stress were significantly decreased, and the sperm malformation rate was significantly increased. The sperm motility was assessed and we found that heat stress significantly reduced the number of grade A sperm and increased the number of grade B and C sperm cells. Studies have demonstrated that increased ejaculation, sperm cell concentration, and seminal plasma composition were primarily related to testosterone levels (Abu Zeid, Alam & Abd El-Hameed, 2017). Testosterone concentration is essential to maintain the auxiliary gland function of male animals (such as protein and fructose synthesis), and fructose in the seminal plasma is secreted by seminal vesicles, which is very sensitive to androgen stimulation (Walker, 2009). Fructose is an essential nutrient for sperm cells, which can be metabolized and converted into pyruvic acid and lactic acid, thereby supporting sperm motility. The decrease in the seminal fructose concentration is known to weak sperm motility (Hosny et al., 2020). In this experiment, it was found that heat stress significantly decreased the testosterone level and fructose content in the seminal plasma of male rabbits. Under the influence of heat stress, the progressive movement and motility of sperm of male rabbits were decreased. TSO, TMS, and TFSF decreased, indicating that the low semen fertilization rate produced by heat stress male rabbits was attributed to the decrease in the total sperm motility and total functional sperm.

Our study identified possible causes on the impact of heat stress on the metabolism of seminal plasma in rabbits from an endocrine and metabolic view. Epididymis is the primary source of α-glucosidase in human seminal plasma, and α-glucosidase is an important parameter for evaluating semen activity, and epididymis function and patency (Peña et al., 2004). α-glucosidase and lactate dehydrogenase isoenzyme are related to sperm motility. Heat stress damages the epididymis of male rabbits and significantly reduces α-glucosidase (α-Glu) and fructose in the seminal plasma. Studies have reported that the levels of α-glucosidase activity in the semen of men stimulated by scrotal heat stress were low, and there was a positive correlation between α-glucosidase and sperm density, motility, and normal morphology, and normal housing (Zhang et al., 2018). The results of this study are consistent with those of previous studies. Heat stress significantly reduced the concentrations of Mg^2+^, Na^+^, and K^+^ in the metal ions in the seminal plasma of male rabbits. Sperm function was highly dependent on the ionic environment (Hamamah & Gatti, 1998). When the body is in a state of thermal stress, inorganic salt ions in the seminal plasma change, and cations such as Na^+^, K^+^, and Mg^2+^ in the seminal plasma play a role in osmotic balance (Cevik et al., 2007). Na^+^ is the primary cation in the seminal plasma, and K^+^ is a natural metabolic inhibitor. Higher K^+^ concentrations in the seminal plasma will reduce sperm metabolism, thereby reducing sperm motility (Massanyi et al., 2003). Mg^2+^ ion exists in almost all enzymatic systems, which is considered a marker of seminal vesicle secretion (Wong et al., 2001) and may play an important role in sperm motility (Jobim et al., 2004). The results of this study were consistent with those of previous studies, indicating that the sperm metabolism of male rabbits was enhanced and osmotic pressure was changed under thermal stress.

In addition, UPLC-MS/MS analysis was performed to determine the metabolites of the heat stress group and non-heat stress group. We detected a total of 346 metabolites and screened 71 differential metabolites, including 48 significantly up-regulated metabolites and 23 significantly down-regulated metabolites. The metabolites were present in several biochemical pathways (Dunn et al., 2011) and may be used as potential biomarkers for male fertility (Gilany et al., 2017; Kovac, Pastuszak & Lamb, 2013; Qiao et al., 2017). During ejaculation, sperm cells are suspended in the seminal plasma, and the difference in the quality and quantity of biochemical components in the plasma significantly affects the state of sperm cells. The contact of sperm cells with small molecules such as metabolites can improve or hinder the fertilization ability of sperm cells, even when sperm cells are preserved in diluents. In addition, metabolites such as amino acids, carbohydrates, fatty acids, and nucleosides are involved in physiological changes in the livestock, affecting sperm energy production, motility, pH control, and metabolic activities (Juyena & Stelletta, 2012; Patel et al., 1999; Williams & Ford, 2001). The metabolites affecting the quality of semen were screened by analyzing the function and metabolic pathway of differential metabolites, which provided a new solution for the “summer infertility” of male rabbits.

## Conclusions

In conclusion, ambient temperature leads to heat stress in male New Zealand white rabbits. After heat stress, the sperm motility, pH value, and density of male rabbits decreased significantly, and the sperm malformation rate increased significantly. Consequently, the quality of semen deteriorated and the energy metabolism pathway was disturbed. The results of this study provide clues for studying the decline in the quality of the semen of male New Zealand white rabbits under heat stress.

## Supplemental Information

10.7717/peerj.15112/supp-1 Supplemental Information 1 Author Checklist.

10.7717/peerj.15112/supp-2 Supplemental Information 2 The original results of metabolites.

10.7717/peerj.15112/supp-3 Supplemental Information 3 Raw test data.

10.7717/peerj.15112/supp-4 Supplemental Information 4 The supplemental table of differential metabolites.

10.7717/peerj.15112/supp-5 Supplemental Information 5 Elution conditions of mobile phase gradient.

10.7717/peerj.15112/supp-6 Supplemental Information 6 Temperature, humidity, and THI index changes in rabbit house in May and August.

10.7717/peerj.15112/supp-7 Supplemental Information 7 Effects of heat stress on sperm motility of male rabbits.

10.7717/peerj.15112/supp-8 Supplemental Information 8 Effects of heat stress on semen quality parameters of male rabbits.

10.7717/peerj.15112/supp-9 Supplemental Information 9 Effects of heat stress on seminal plasma biochemical indices of male rabbits.

## Additional Information and Declarations

### Data Availability

The following information was supplied regarding data availability: The raw measurements are available in the Supplemental Files.
