# Supplementary material for: Semen quality and seminal plasma metabolites in male rabbits (Oryctolagus cuniculus) under heat stress
Source: PeerJ. 2023 Apr 7;11:e15112. doi: 10.7717/peerj.15112 (PMC10103697; doi:10.7717/peerj.15112)
Supplement: Supplemental Information 2 [file peerj-11-15112-s002.zip › peerj-75361-Raw_data_result/Raw data/Result-X101SC21103966-Z01-J001-B1-42/2.MetAnnotation/HMDB/meta_neg.HMDB.Anno.pdf]

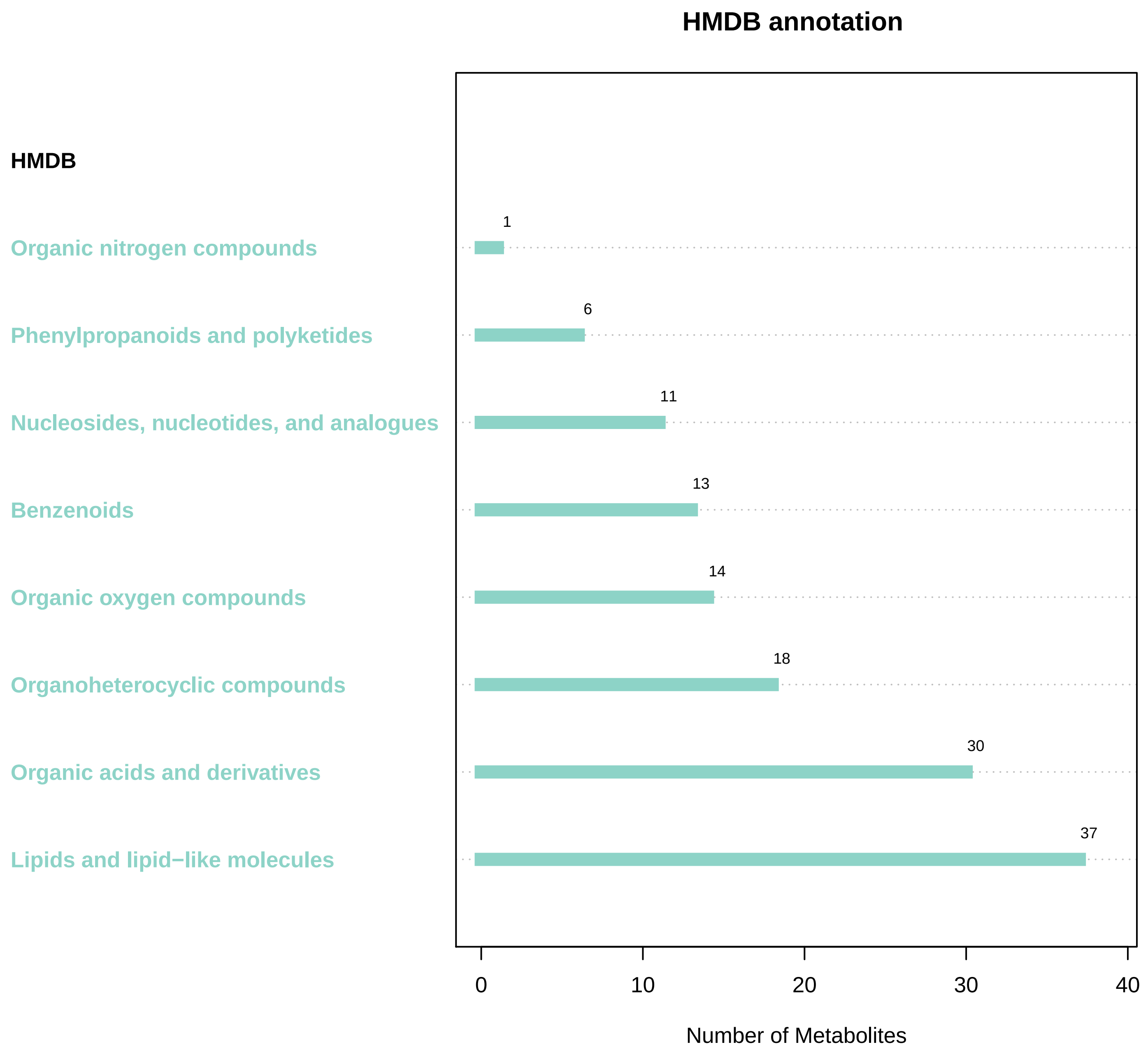
HMDB annotation
HMDB
Organic nitrogen compounds
Phenylpropanoids and polyketides
Nucleosides, nucleotides, and analogues
Benzenoids
Organic oxygen compounds
Organoheterocyclic compounds
Organic acids and derivatives
Lipids and lipid−like molecules
1
6
11
13
14
18
30
37
0
10
20
30
40
Number of Metabolites
