# Supplementary material for: Semen quality and seminal plasma metabolites in male rabbits (Oryctolagus cuniculus) under heat stress
Source: PeerJ. 2023 Apr 7;11:e15112. doi: 10.7717/peerj.15112 (PMC10103697; doi:10.7717/peerj.15112)
Supplement: Supplemental Information 2 [file peerj-11-15112-s002.zip › peerj-75361-Raw_data_result/Raw data/Result-X101SC21103966-Z01-J001-B1-42/2.MetAnnotation/KEGG/meta_pos.KEGG.Anno.pdf]

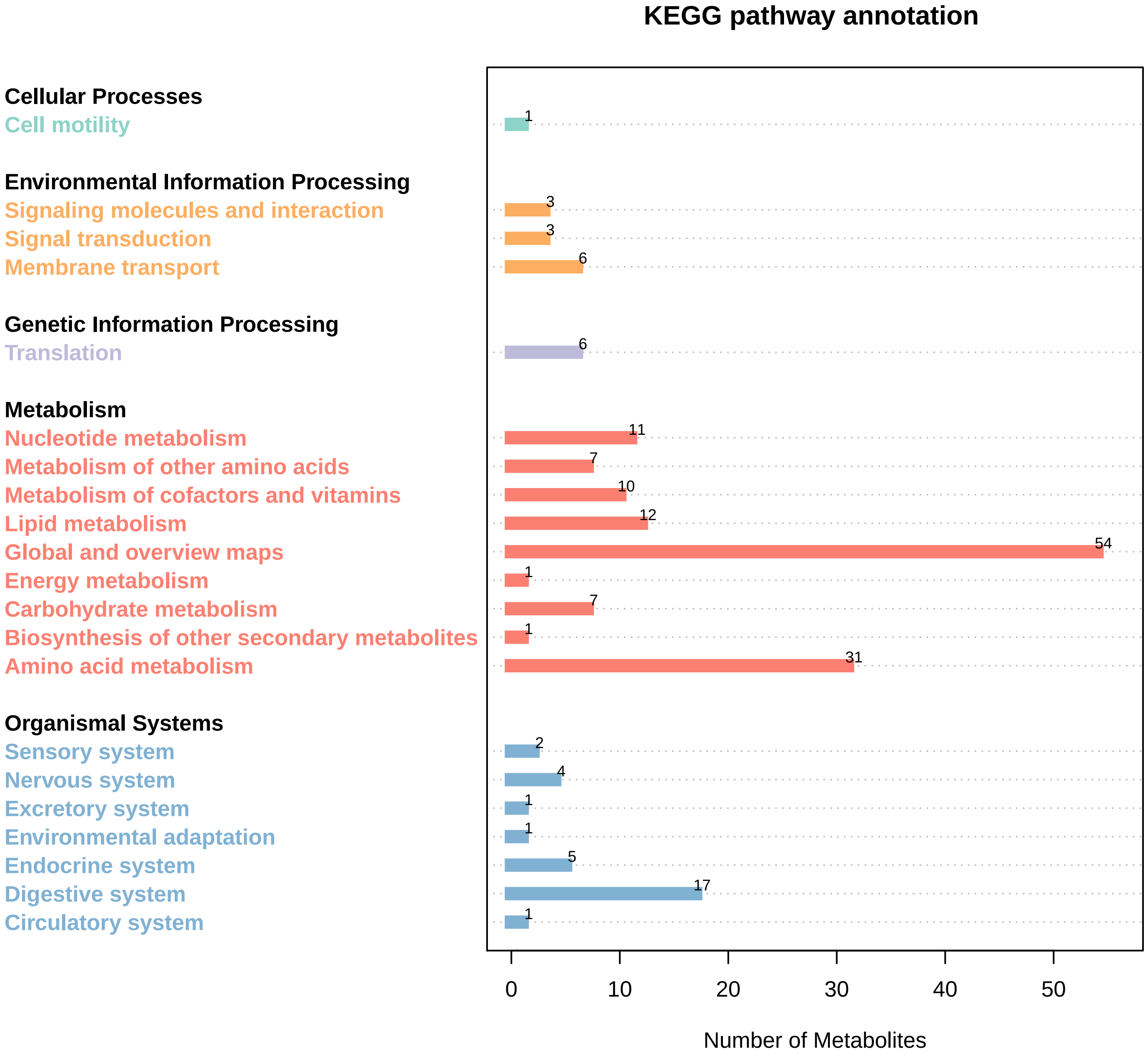
KEGG pathway annotation
Cellular Processes
Cell motility
Environmental Information Processing
Signaling molecules and interaction
Signal transduction
Membrane transport
Genetic Information Processing
Translation
Metabolism
Nucleotide metabolism
Metabolism of other amino acids
Metabolism of cofactors and vitamins
Lipid metabolism
Global and overview maps
Energy metabolism
Carbohydrate metabolism
Biosynthesis of other secondary metabolites
Amino acid metabolism
Organismal Systems
Sensory system
Nervous system
Excretory system
Environmental adaptation
Endocrine system
Digestive system
Circulatory system
1
3
3
6
6
11
7
10
12
54
1
7
1
31
2
4
1
1
5
17
1
0
10
20
30
40
50
Number of Metabolites
