# Supplementary material for: Semen quality and seminal plasma metabolites in male rabbits (Oryctolagus cuniculus) under heat stress
Source: PeerJ. 2023 Apr 7;11:e15112. doi: 10.7717/peerj.15112 (PMC10103697; doi:10.7717/peerj.15112)
Supplement: Supplemental Information 2 [file peerj-11-15112-s002.zip › peerj-75361-Raw_data_result/Raw data/Result-X101SC21103966-Z01-J001-B1-42/2.MetAnnotation/Lipidmaps/meta_neg.Lipidmaps.Anno.pdf]

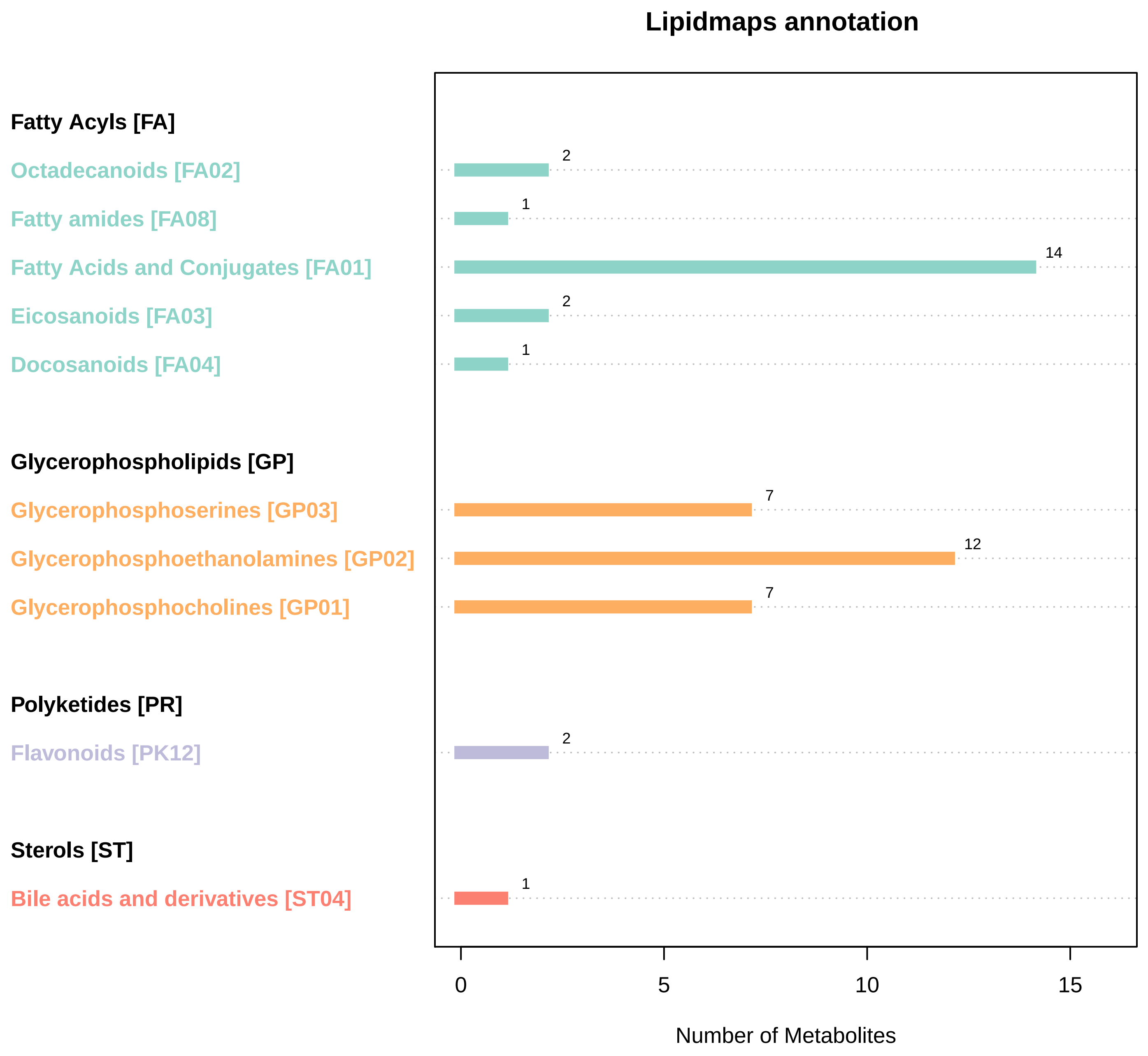
Lipidmaps annotation
Fatty Acyls [FA]
Octadecanoids [FA02]
Fatty amides [FA08]
Fatty Acids and Conjugates [FA01]
Eicosanoids [FA03]
Docosanoids [FA04]
Glycerophospholipids [GP]
Glycerophosphoserines [GP03]
Glycerophosphoethanolamines [GP02]
Glycerophosphocholines [GP01]
Polyketides [PR]
Flavonoids [PK12]
Sterols [ST]
Bile acids and derivatives [ST04]
2
1
14
2
1
7
12
7
2
1
0
5
10
15
Number of Metabolites
