# Supplementary material for: Semen quality and seminal plasma metabolites in male rabbits (Oryctolagus cuniculus) under heat stress
Source: PeerJ. 2023 Apr 7;11:e15112. doi: 10.7717/peerj.15112 (PMC10103697; doi:10.7717/peerj.15112)
Supplement: Supplemental Information 2 [file peerj-11-15112-s002.zip › peerj-75361-Raw_data_result/Raw data/Result-X101SC21103966-Z01-J001-B1-42/2.MetAnnotation/Lipidmaps/meta_pos.Lipidmaps.Anno.pdf]

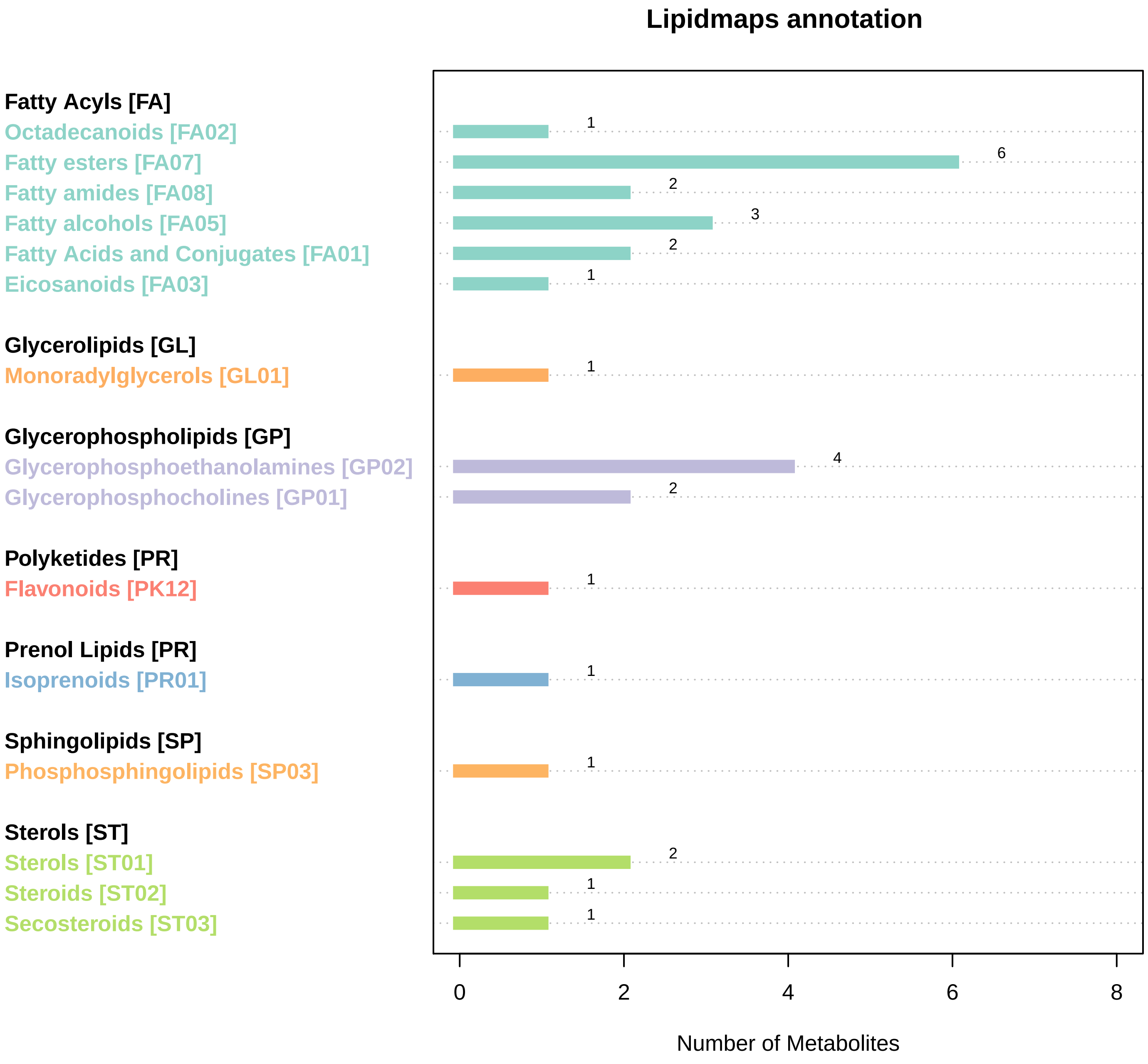
Lipidmaps annotation
Fatty Acyls [FA]
Octadecanoids [FA02]
Fatty esters [FA07]
Fatty amides [FA08]
Fatty alcohols [FA05]
Fatty Acids and Conjugates [FA01]
Eicosanoids [FA03]
Glycerolipids [GL]
Monoradylglycerols [GL01]
Glycerophospholipids [GP]
Glycerophosphoethanolamines [GP02]
Glycerophosphocholines [GP01]
Polyketides [PR]
Flavonoids [PK12]
Prenol Lipids [PR]
Isoprenoids [PR01]
Sphingolipids [SP]
Phosphosphingolipids [SP03]
Sterols [ST]
Sterols [ST01]
Steroids [ST02]
Secosteroids [ST03]
1
6
2
3
2
1
1
4
2
1
1
1
2
1
1
0
2
4
6
8
Number of Metabolites
