# Supplementary figures and images for: Semen quality and seminal plasma metabolites in male rabbits (Oryctolagus cuniculus) under heat stress (part 2 of 2)
Source: PeerJ. 2023 Apr 7;11:e15112. doi: 10.7717/peerj.15112 (PMC10103697; doi:10.7717/peerj.15112)

# H.vs.NH

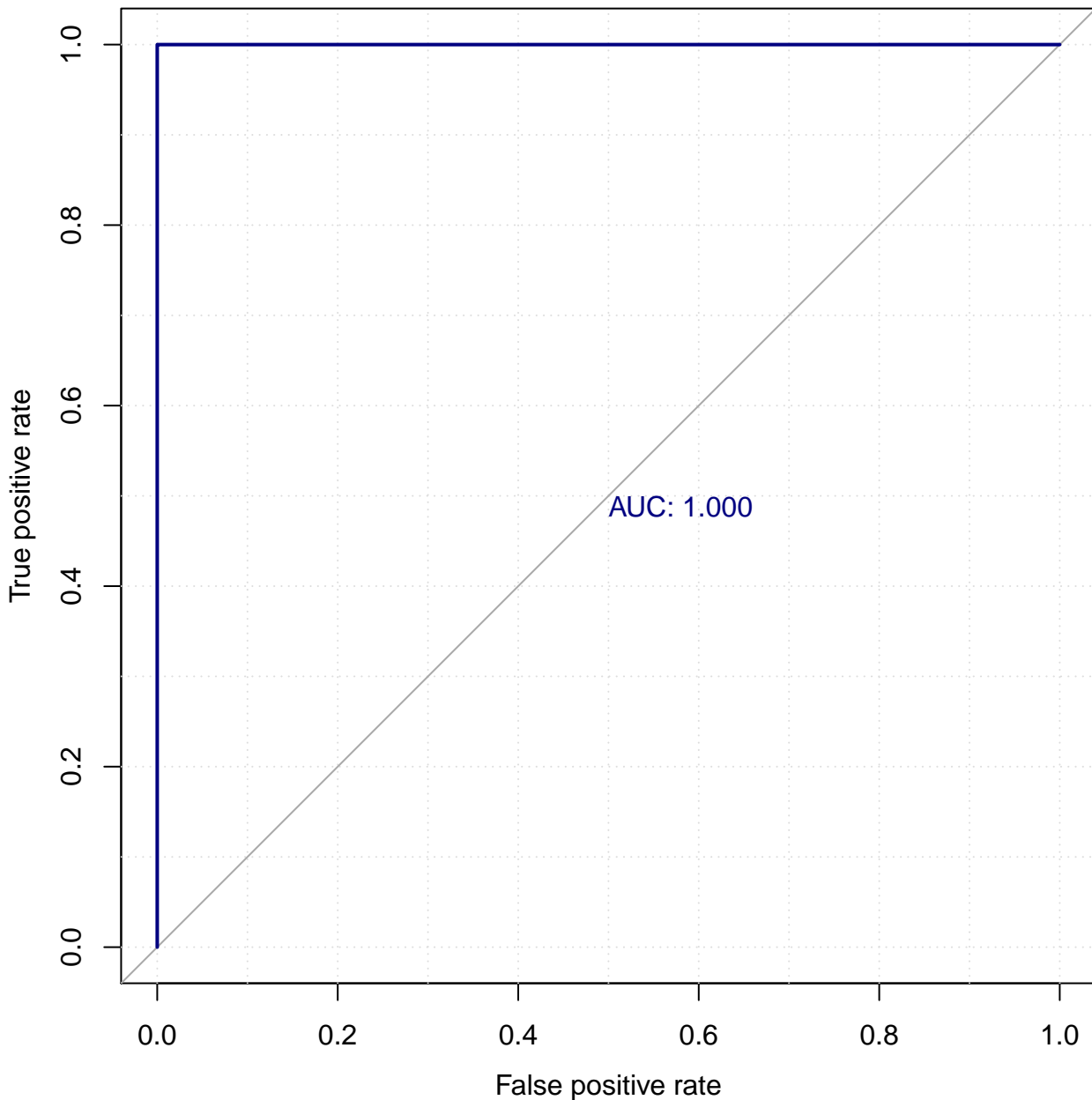

Supplement: Supplemental Information 2 [file peerj-11-15112-s002.zip › peerj-75361-Raw_data_result/Raw data/Result-X101SC21103966-Z01-J001-B1-42/4.MetDiffAnalysis/H.vs.NH/ROC_neg/Com_232_neg_ROC.pdf]

# H.vs.NH

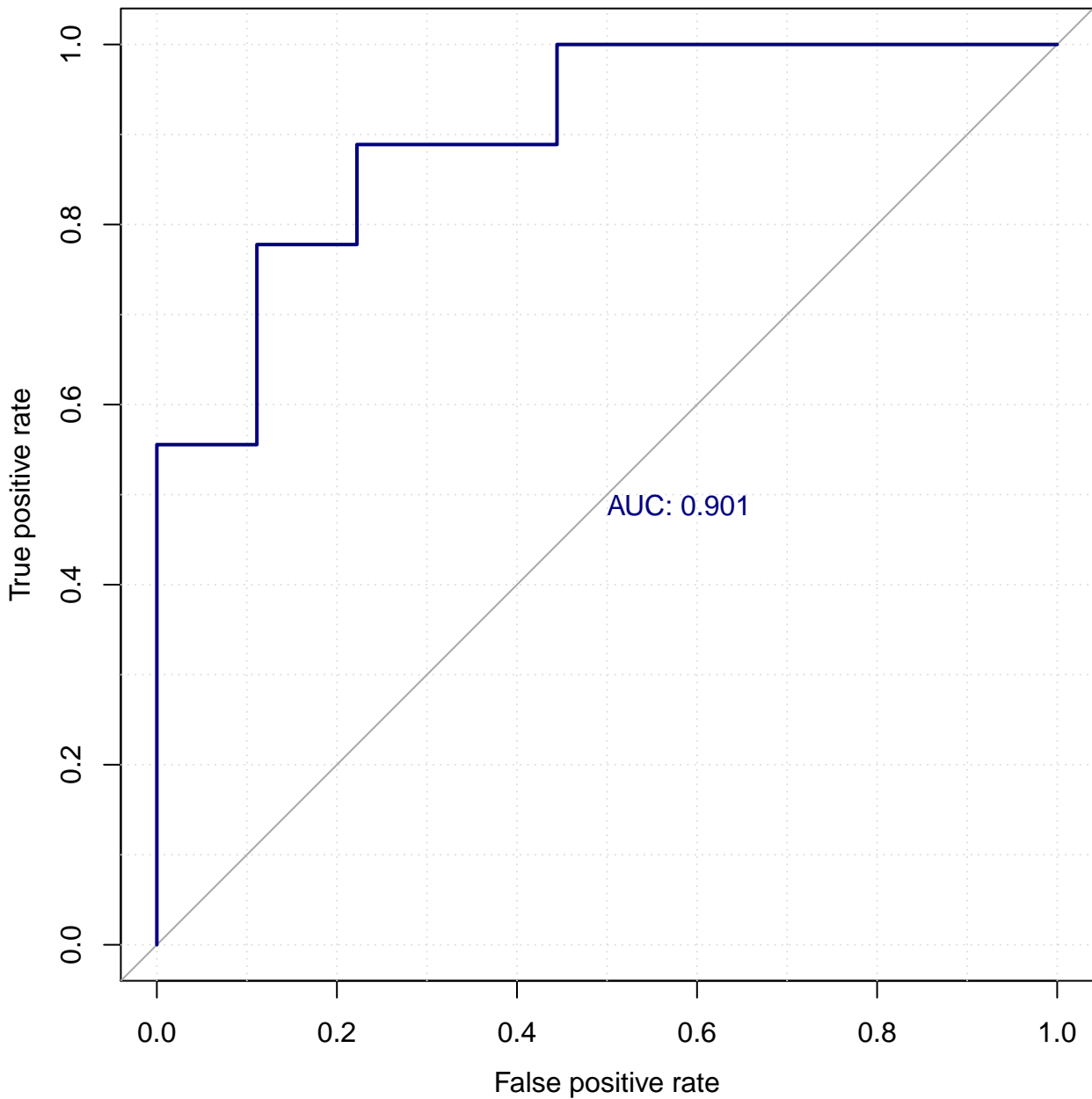

Supplement: Supplemental Information 2 [file peerj-11-15112-s002.zip › peerj-75361-Raw_data_result/Raw data/Result-X101SC21103966-Z01-J001-B1-42/4.MetDiffAnalysis/H.vs.NH/ROC_neg/Com_2469_neg_ROC.pdf]

# H.vs.NH

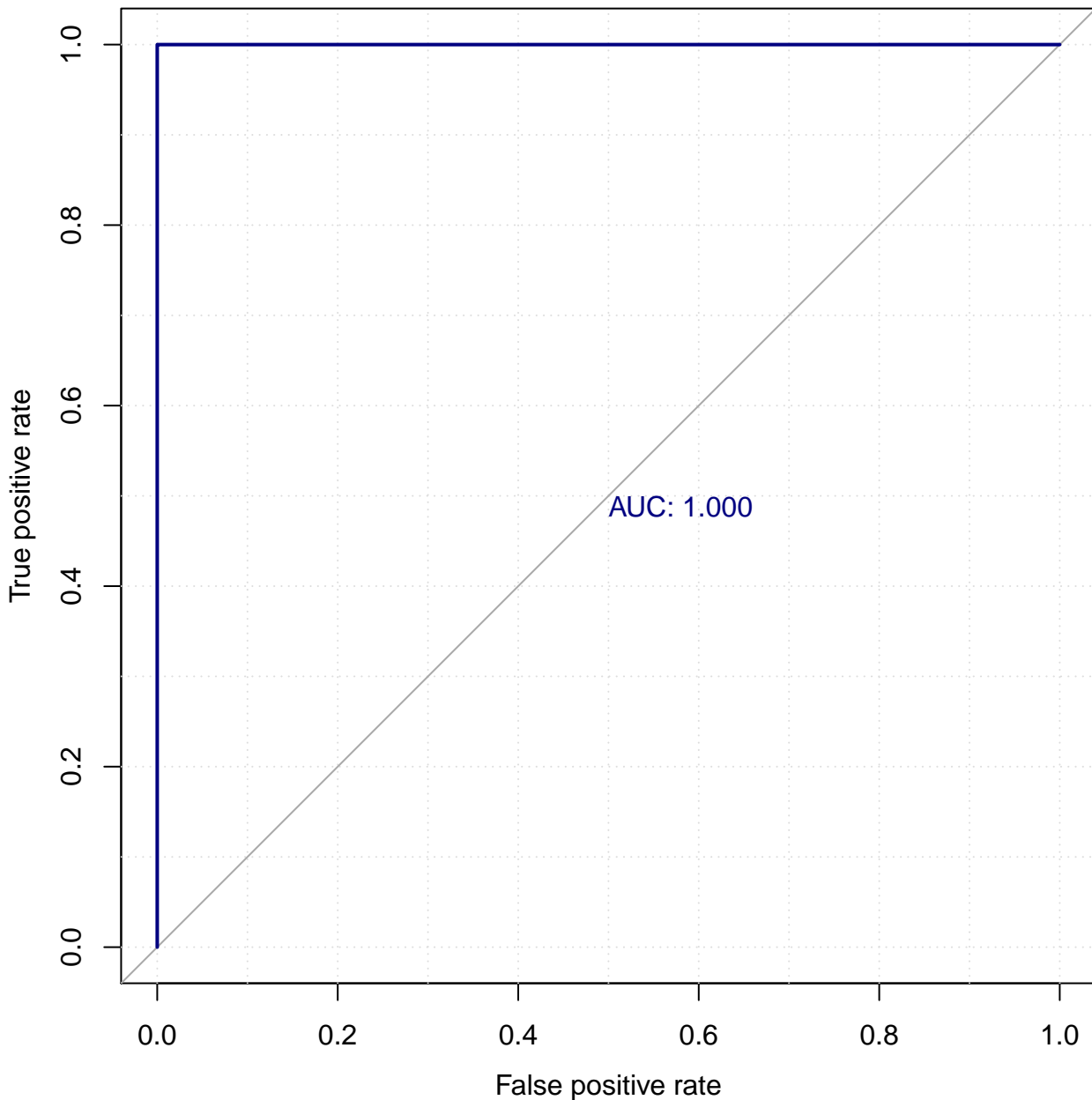

Supplement: Supplemental Information 2 [file peerj-11-15112-s002.zip › peerj-75361-Raw_data_result/Raw data/Result-X101SC21103966-Z01-J001-B1-42/4.MetDiffAnalysis/H.vs.NH/ROC_neg/Com_2522_neg_ROC.pdf]

# H.vs.NH

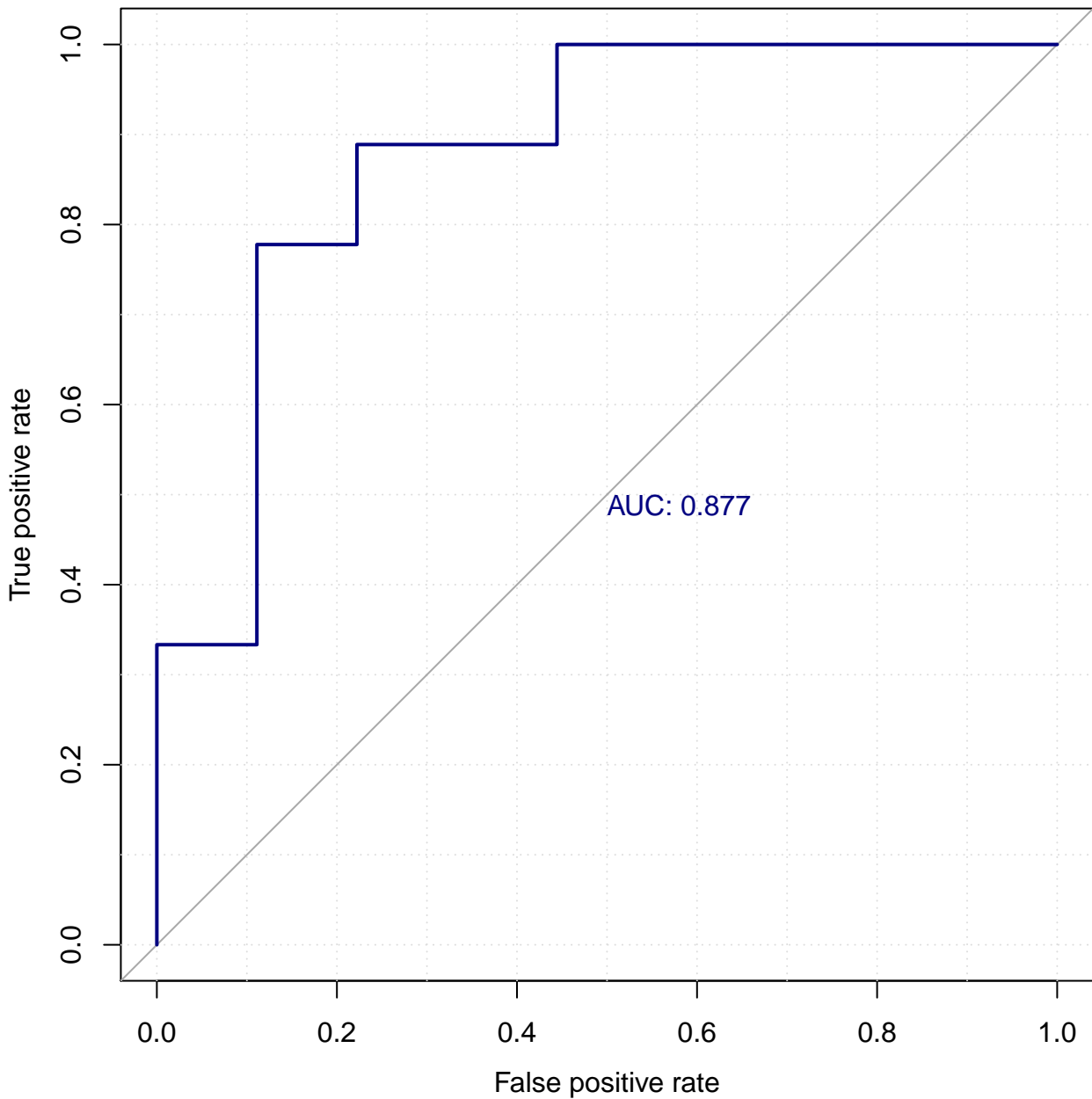

Supplement: Supplemental Information 2 [file peerj-11-15112-s002.zip › peerj-75361-Raw_data_result/Raw data/Result-X101SC21103966-Z01-J001-B1-42/4.MetDiffAnalysis/H.vs.NH/ROC_neg/Com_256_neg_ROC.pdf]

# H.vs.NH

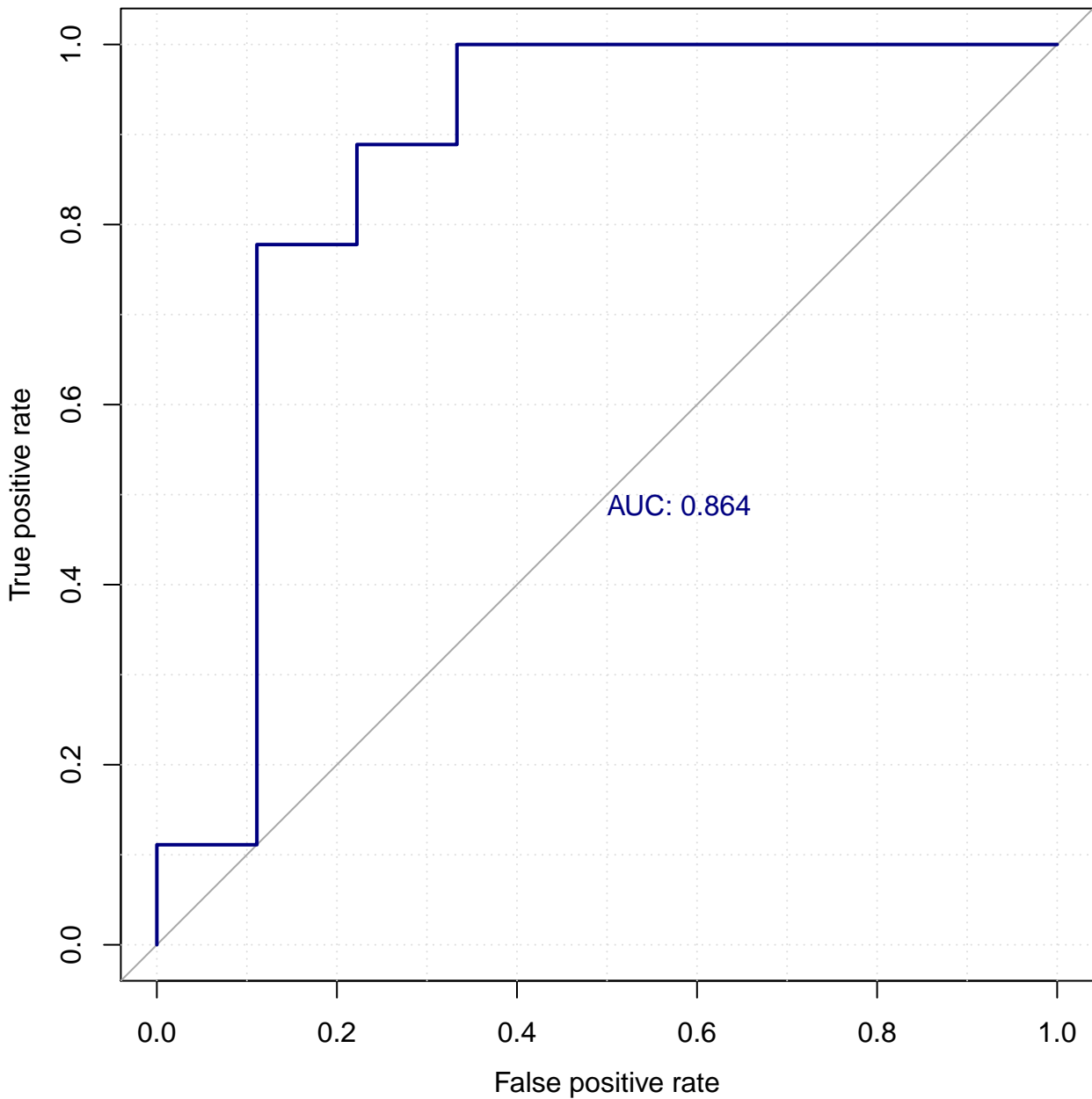

Supplement: Supplemental Information 2 [file peerj-11-15112-s002.zip › peerj-75361-Raw_data_result/Raw data/Result-X101SC21103966-Z01-J001-B1-42/4.MetDiffAnalysis/H.vs.NH/ROC_neg/Com_2635_neg_ROC.pdf]

H.vs.NH

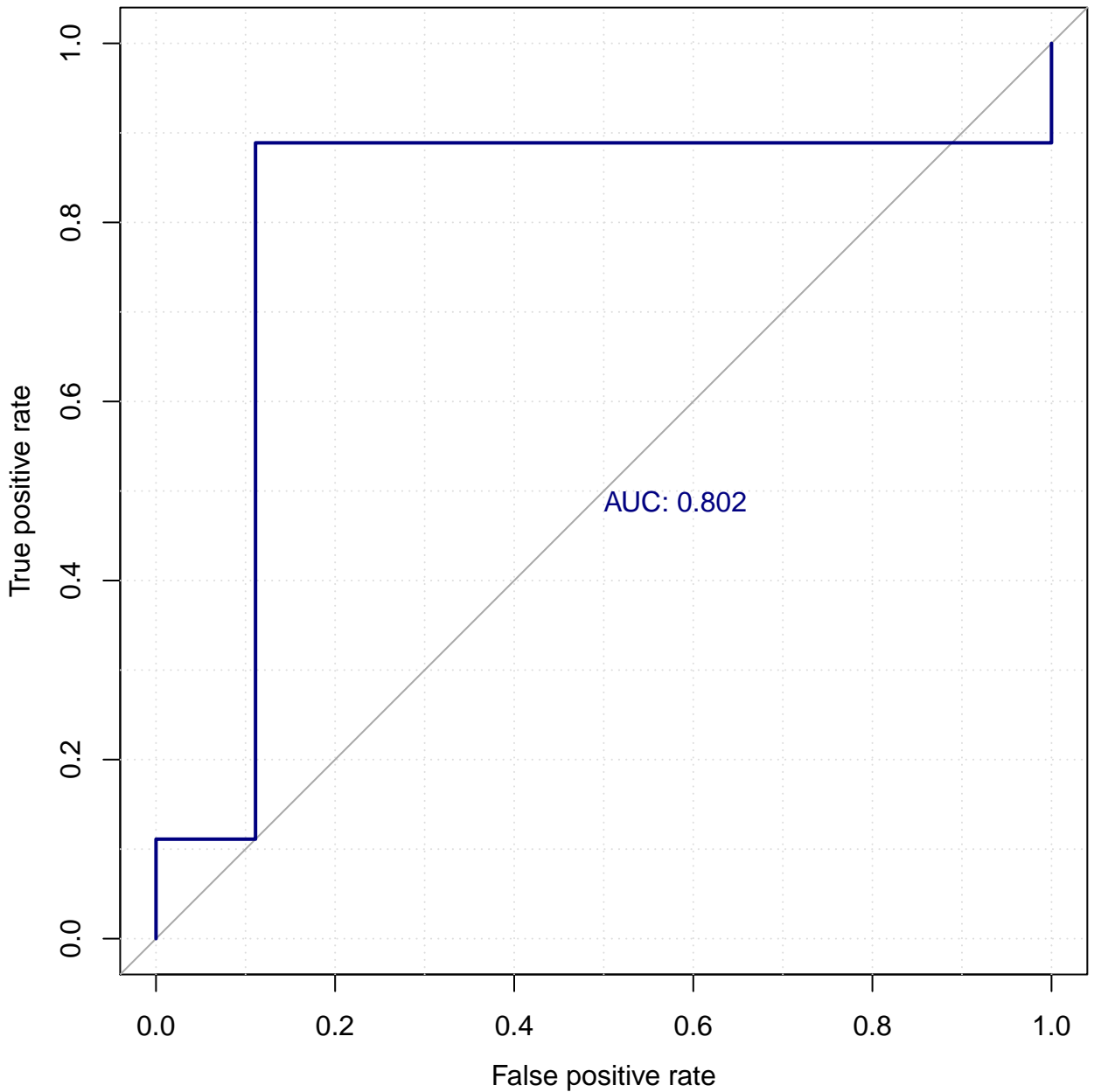

Supplement: Supplemental Information 2 [file peerj-11-15112-s002.zip › peerj-75361-Raw_data_result/Raw data/Result-X101SC21103966-Z01-J001-B1-42/4.MetDiffAnalysis/H.vs.NH/ROC_neg/Com_3132_neg_ROC.pdf]

# H.vs.NH

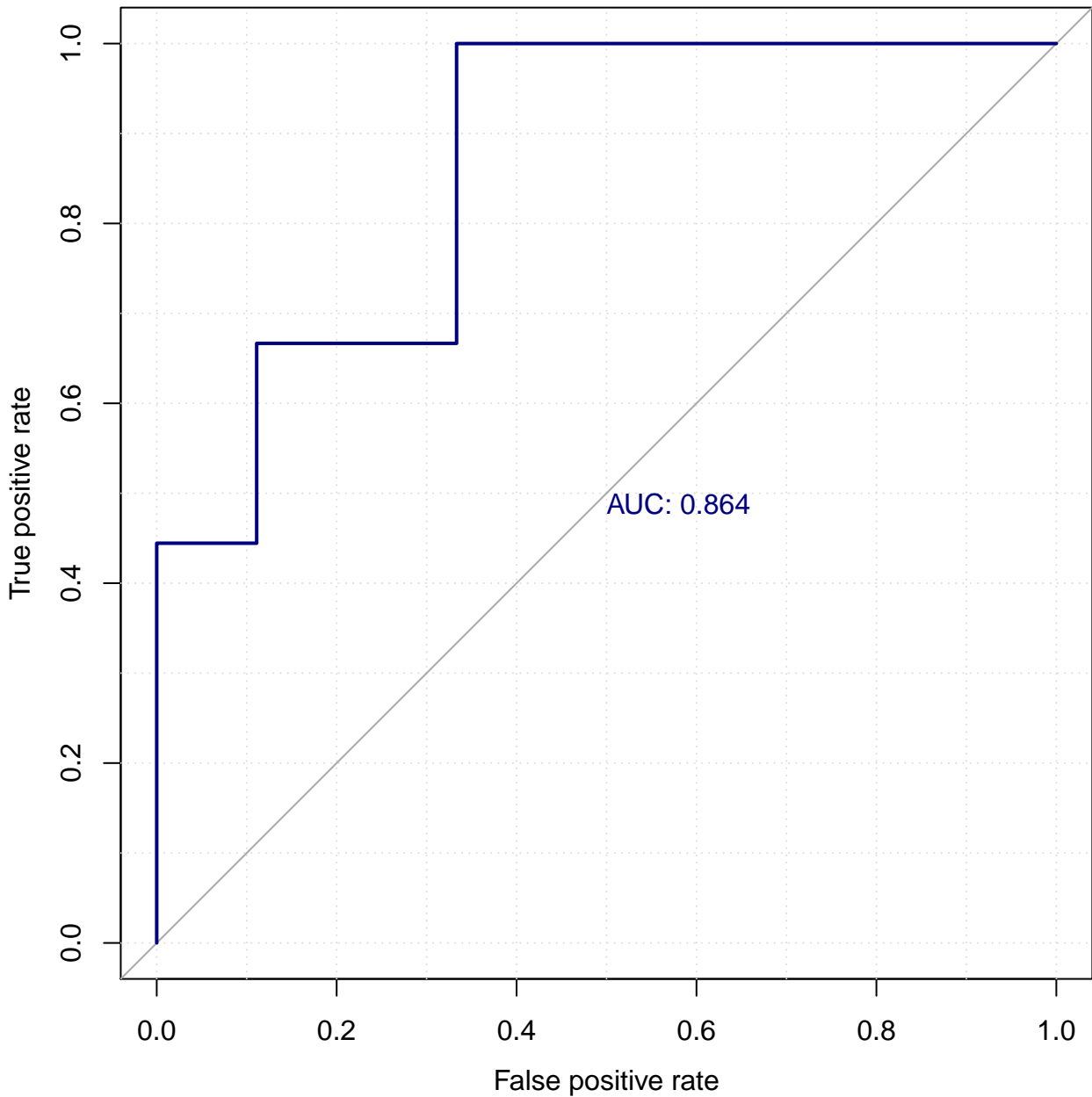

Supplement: Supplemental Information 2 [file peerj-11-15112-s002.zip › peerj-75361-Raw_data_result/Raw data/Result-X101SC21103966-Z01-J001-B1-42/4.MetDiffAnalysis/H.vs.NH/ROC_neg/Com_325_neg_ROC.pdf]

H.vs.NH

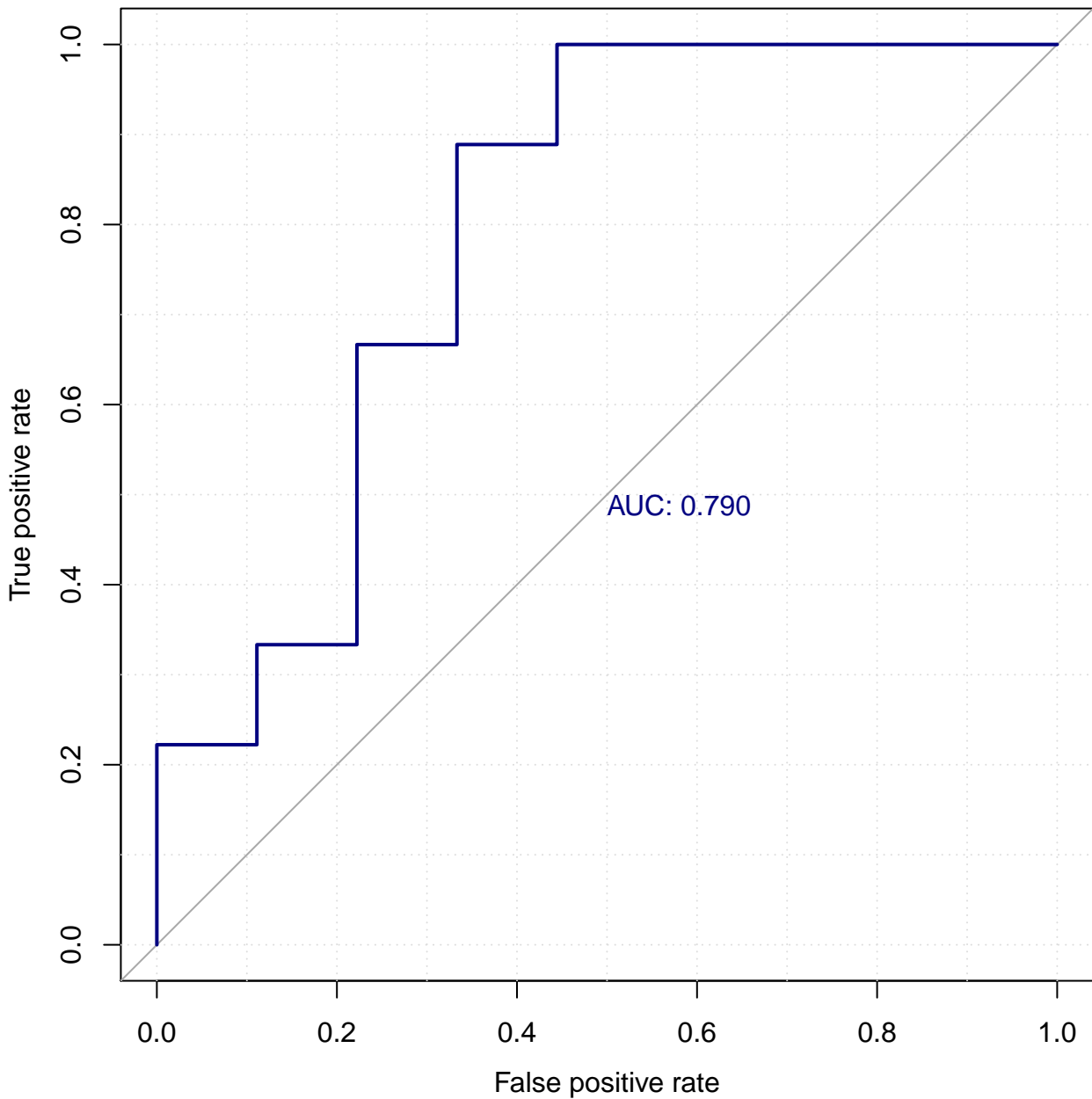

Supplement: Supplemental Information 2 [file peerj-11-15112-s002.zip › peerj-75361-Raw_data_result/Raw data/Result-X101SC21103966-Z01-J001-B1-42/4.MetDiffAnalysis/H.vs.NH/ROC_neg/Com_34_neg_ROC.pdf]

# H.vs.NH

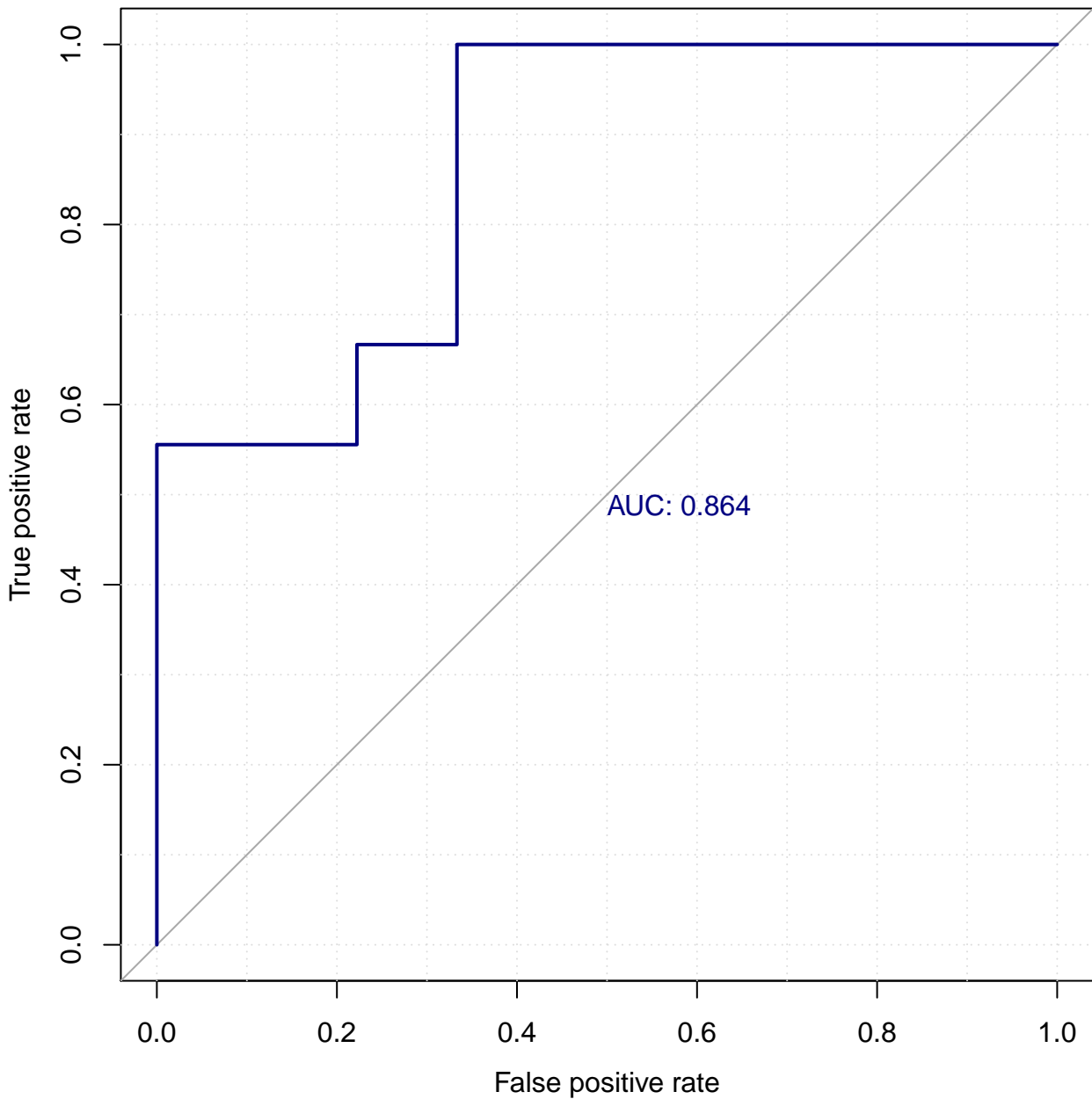

Supplement: Supplemental Information 2 [file peerj-11-15112-s002.zip › peerj-75361-Raw_data_result/Raw data/Result-X101SC21103966-Z01-J001-B1-42/4.MetDiffAnalysis/H.vs.NH/ROC_neg/Com_383_neg_ROC.pdf]

# H.vs.NH

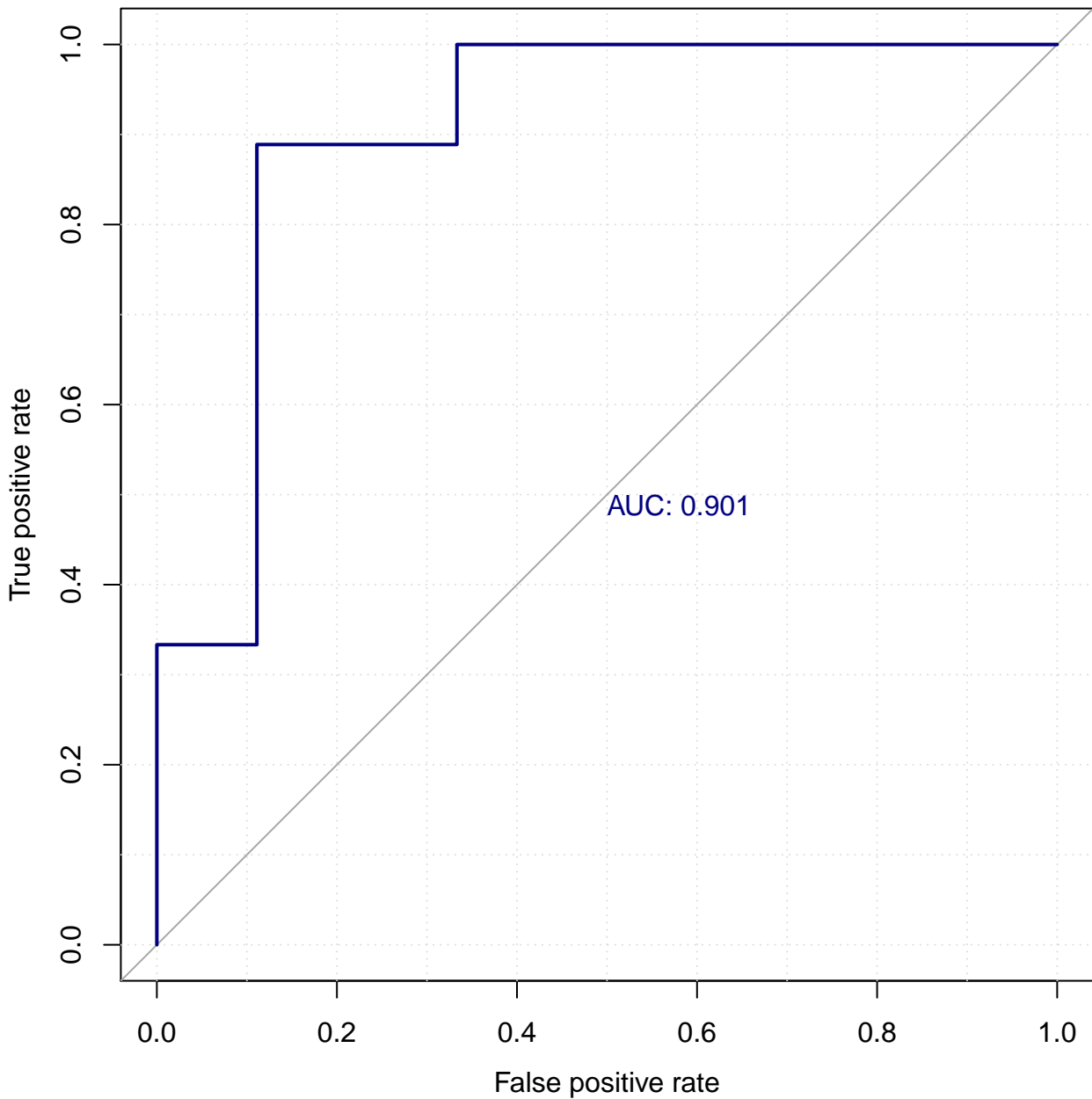

Supplement: Supplemental Information 2 [file peerj-11-15112-s002.zip › peerj-75361-Raw_data_result/Raw data/Result-X101SC21103966-Z01-J001-B1-42/4.MetDiffAnalysis/H.vs.NH/ROC_neg/Com_4206_neg_ROC.pdf]

# H.vs.NH

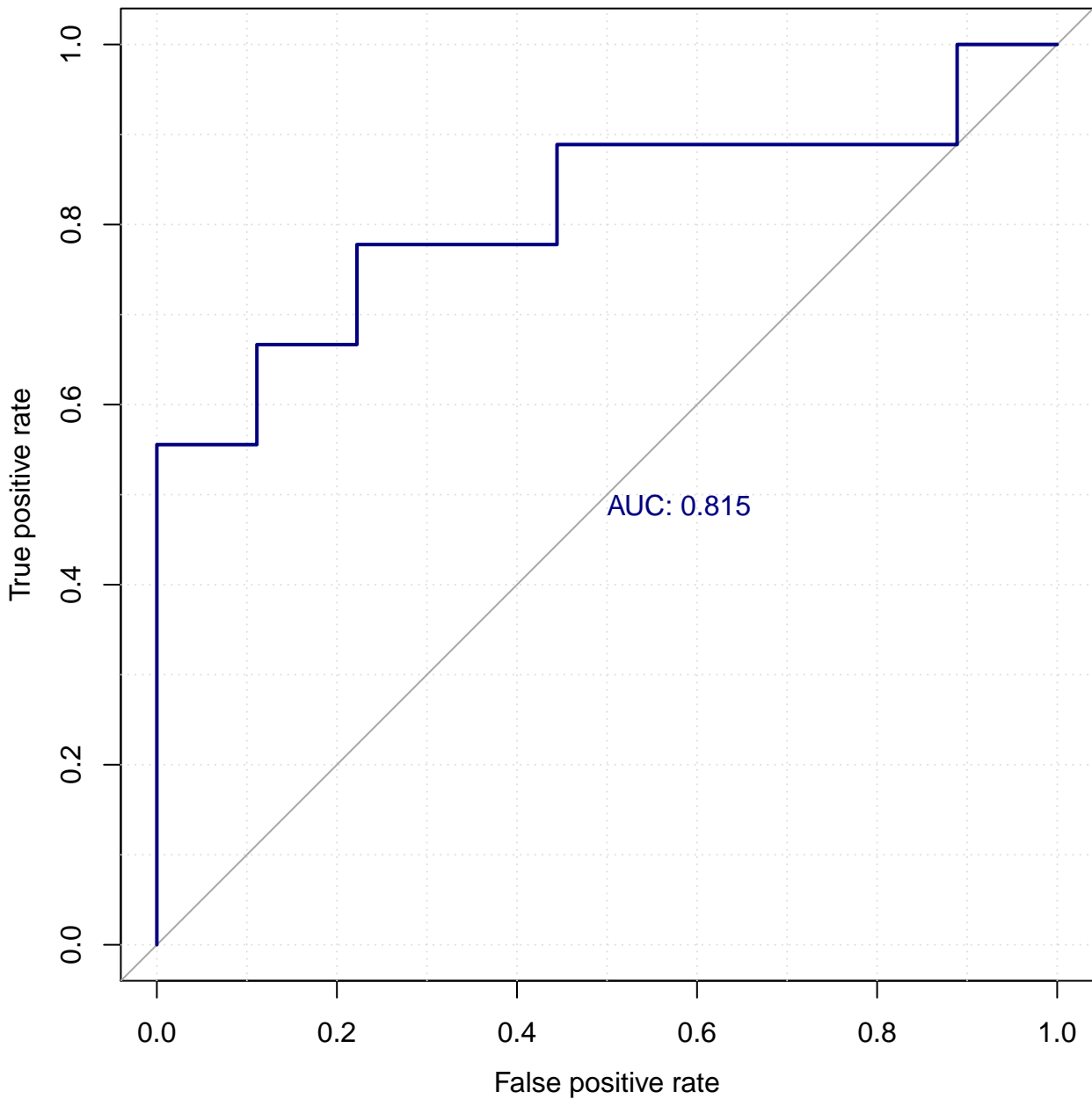

Supplement: Supplemental Information 2 [file peerj-11-15112-s002.zip › peerj-75361-Raw_data_result/Raw data/Result-X101SC21103966-Z01-J001-B1-42/4.MetDiffAnalysis/H.vs.NH/ROC_neg/Com_439_neg_ROC.pdf]

H.vs.NH

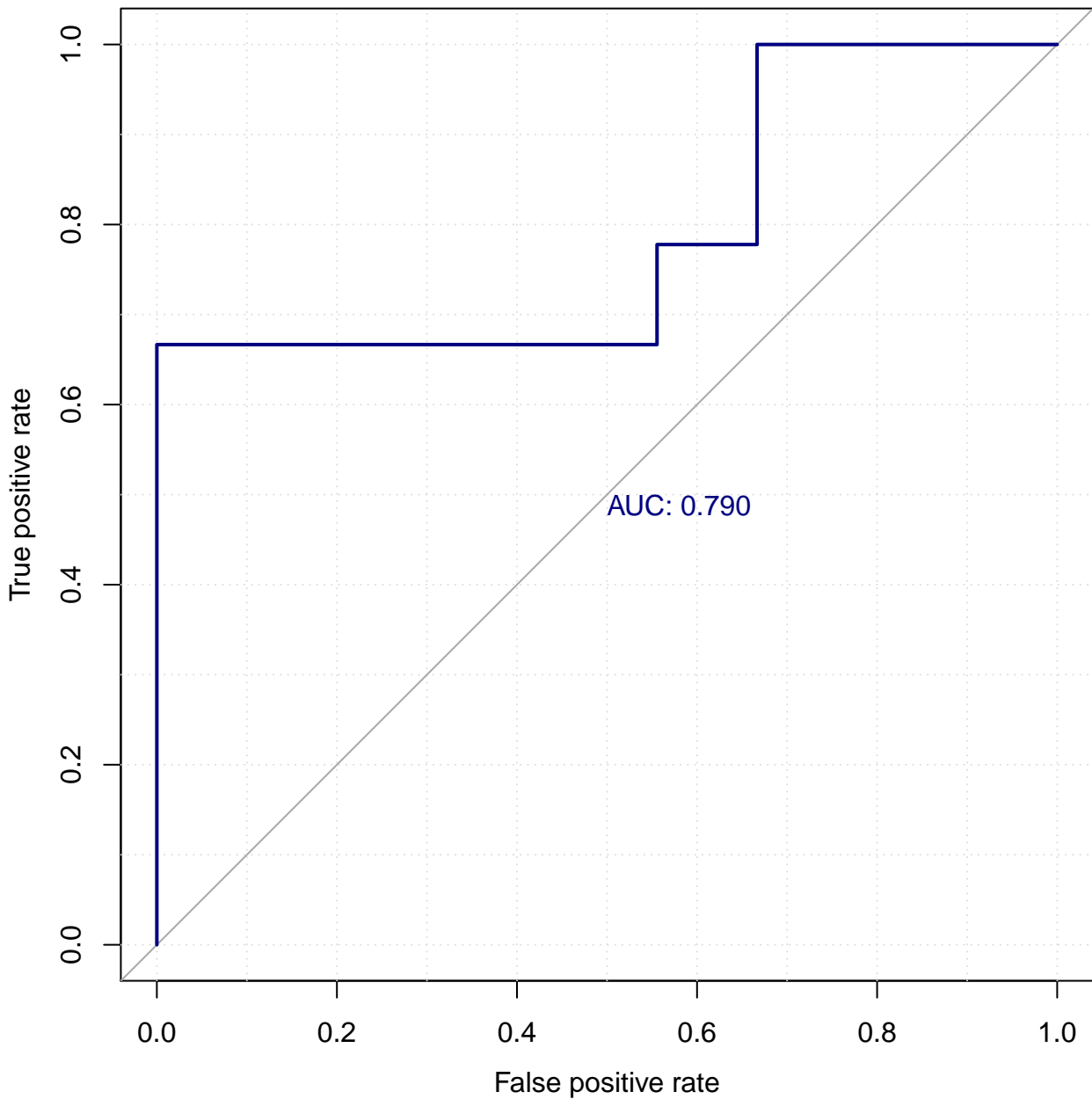

Supplement: Supplemental Information 2 [file peerj-11-15112-s002.zip › peerj-75361-Raw_data_result/Raw data/Result-X101SC21103966-Z01-J001-B1-42/4.MetDiffAnalysis/H.vs.NH/ROC_neg/Com_475_neg_ROC.pdf]

H.vs.NH

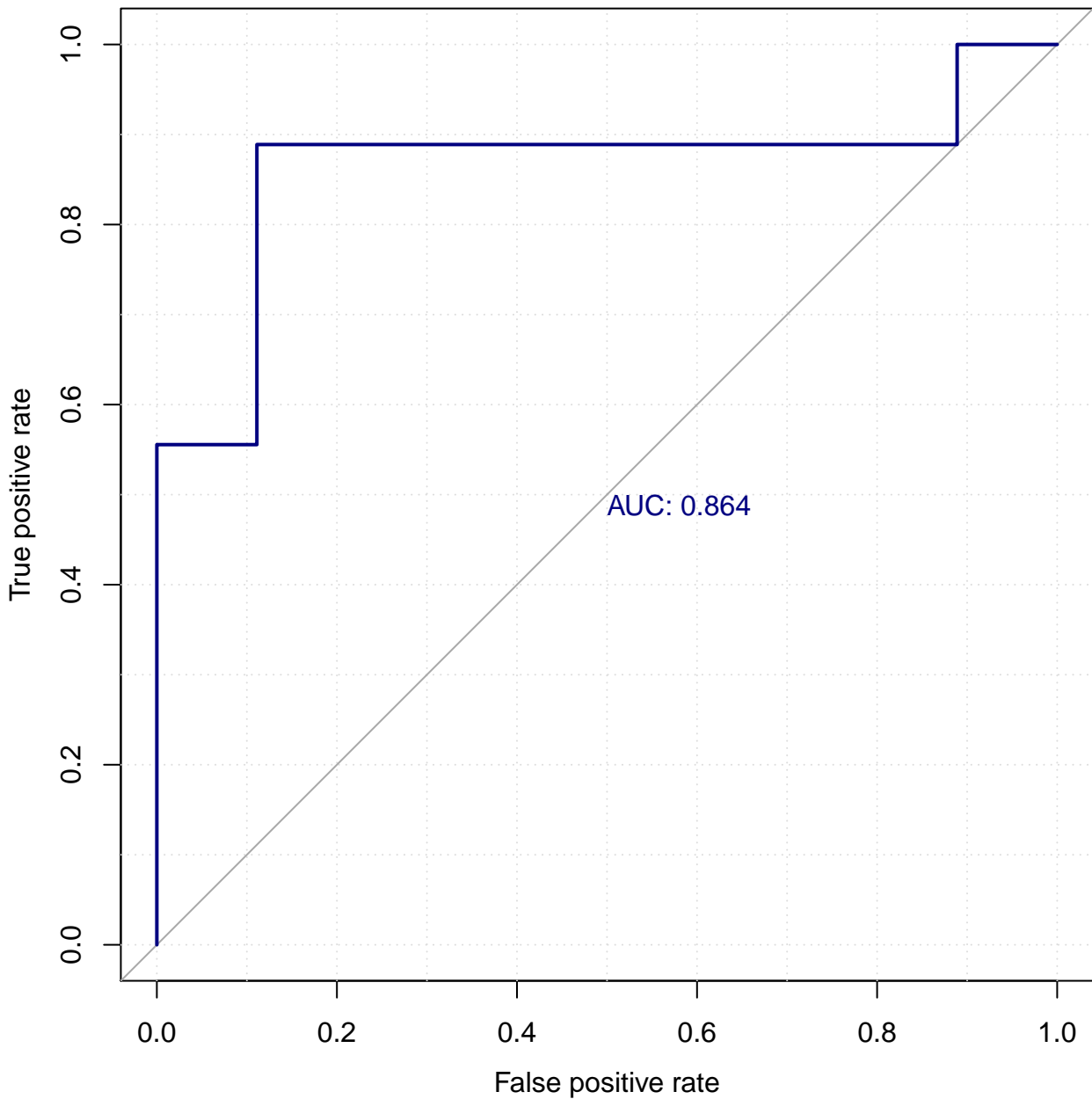

Supplement: Supplemental Information 2 [file peerj-11-15112-s002.zip › peerj-75361-Raw_data_result/Raw data/Result-X101SC21103966-Z01-J001-B1-42/4.MetDiffAnalysis/H.vs.NH/ROC_neg/Com_4787_neg_ROC.pdf]

# H.vs.NH

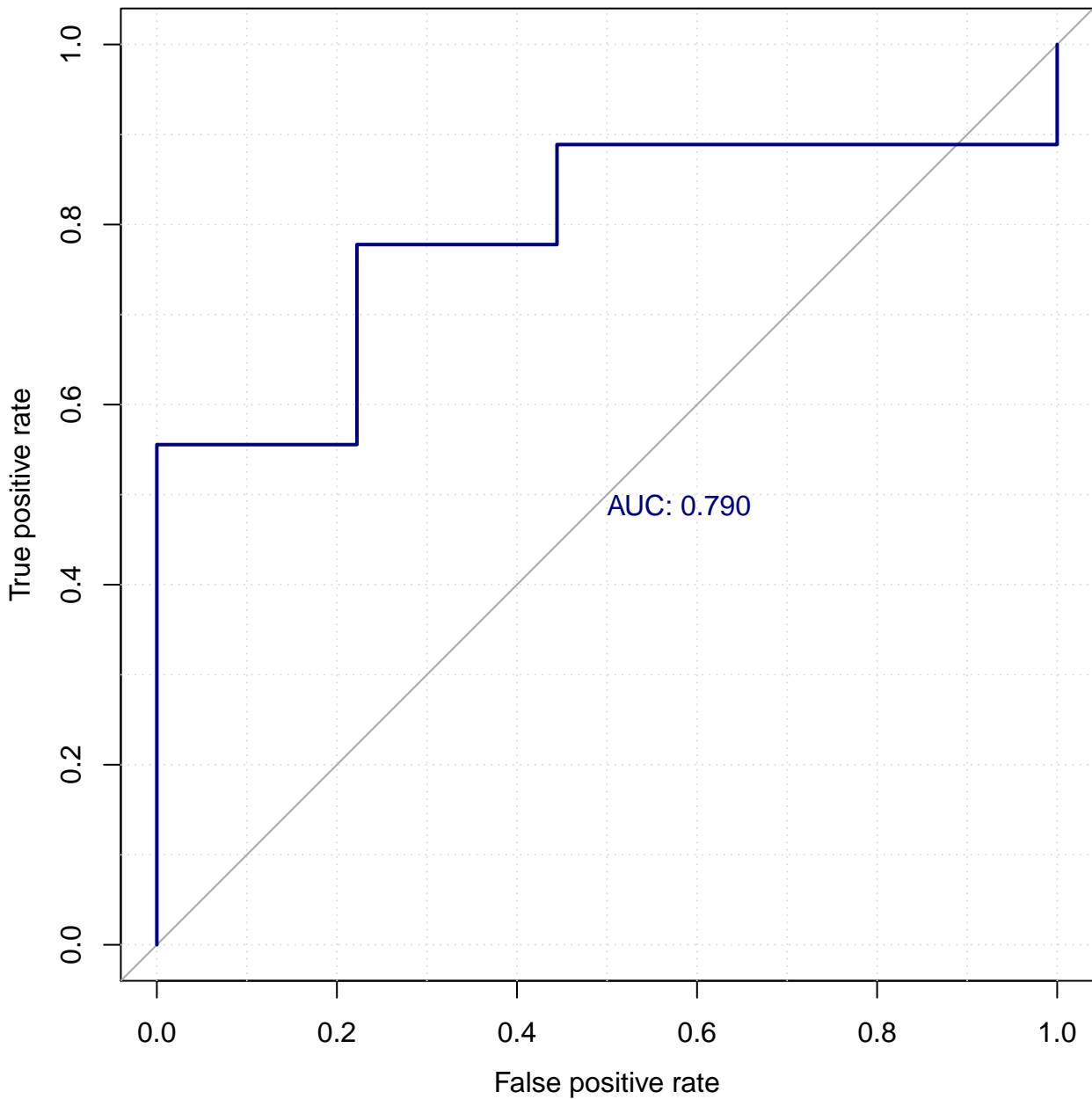

Supplement: Supplemental Information 2 [file peerj-11-15112-s002.zip › peerj-75361-Raw_data_result/Raw data/Result-X101SC21103966-Z01-J001-B1-42/4.MetDiffAnalysis/H.vs.NH/ROC_neg/Com_4826_neg_ROC.pdf]

# H.vs.NH

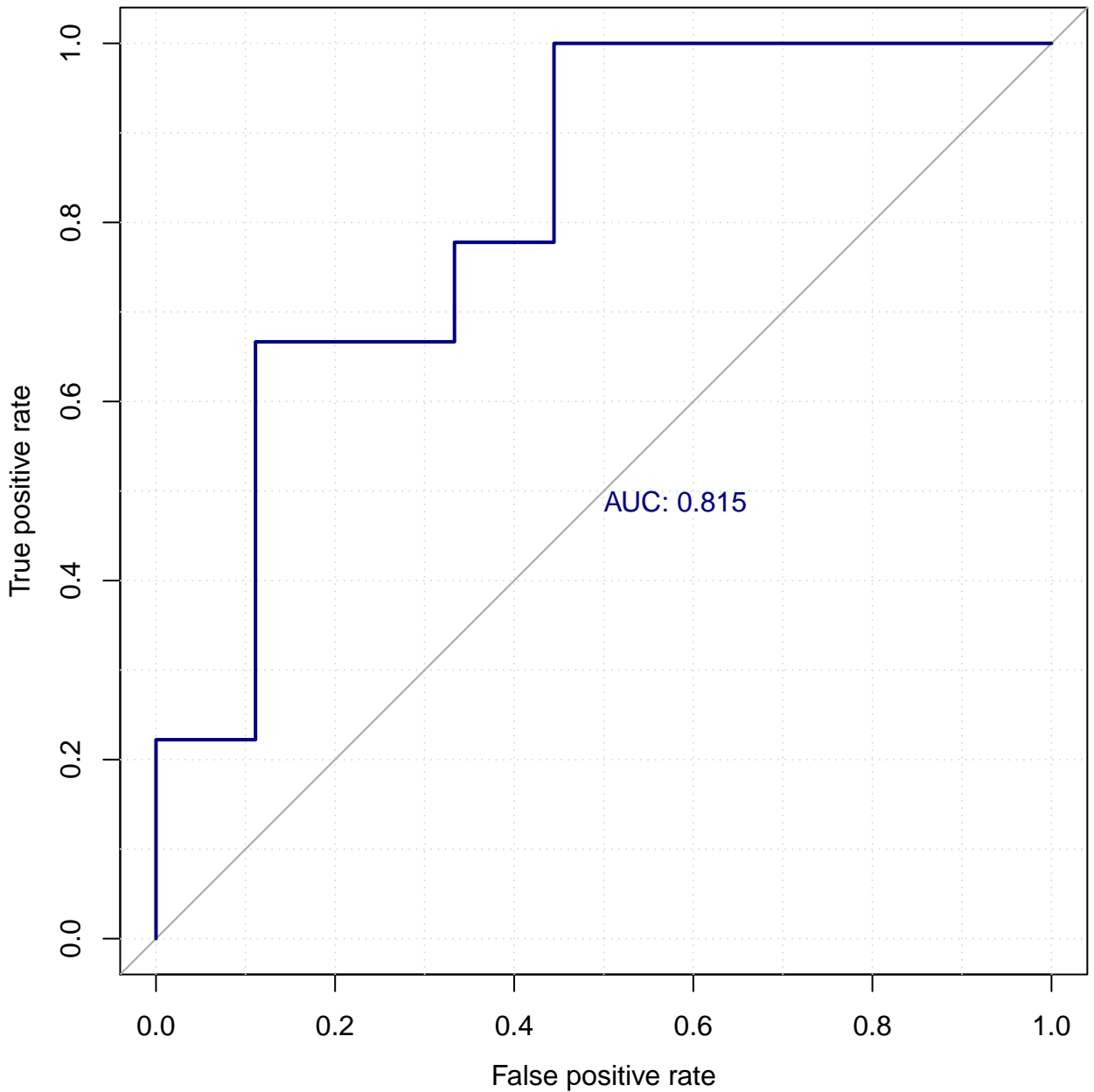

Supplement: Supplemental Information 2 [file peerj-11-15112-s002.zip › peerj-75361-Raw_data_result/Raw data/Result-X101SC21103966-Z01-J001-B1-42/4.MetDiffAnalysis/H.vs.NH/ROC_neg/Com_4908_neg_ROC.pdf]

# H.vs.NH

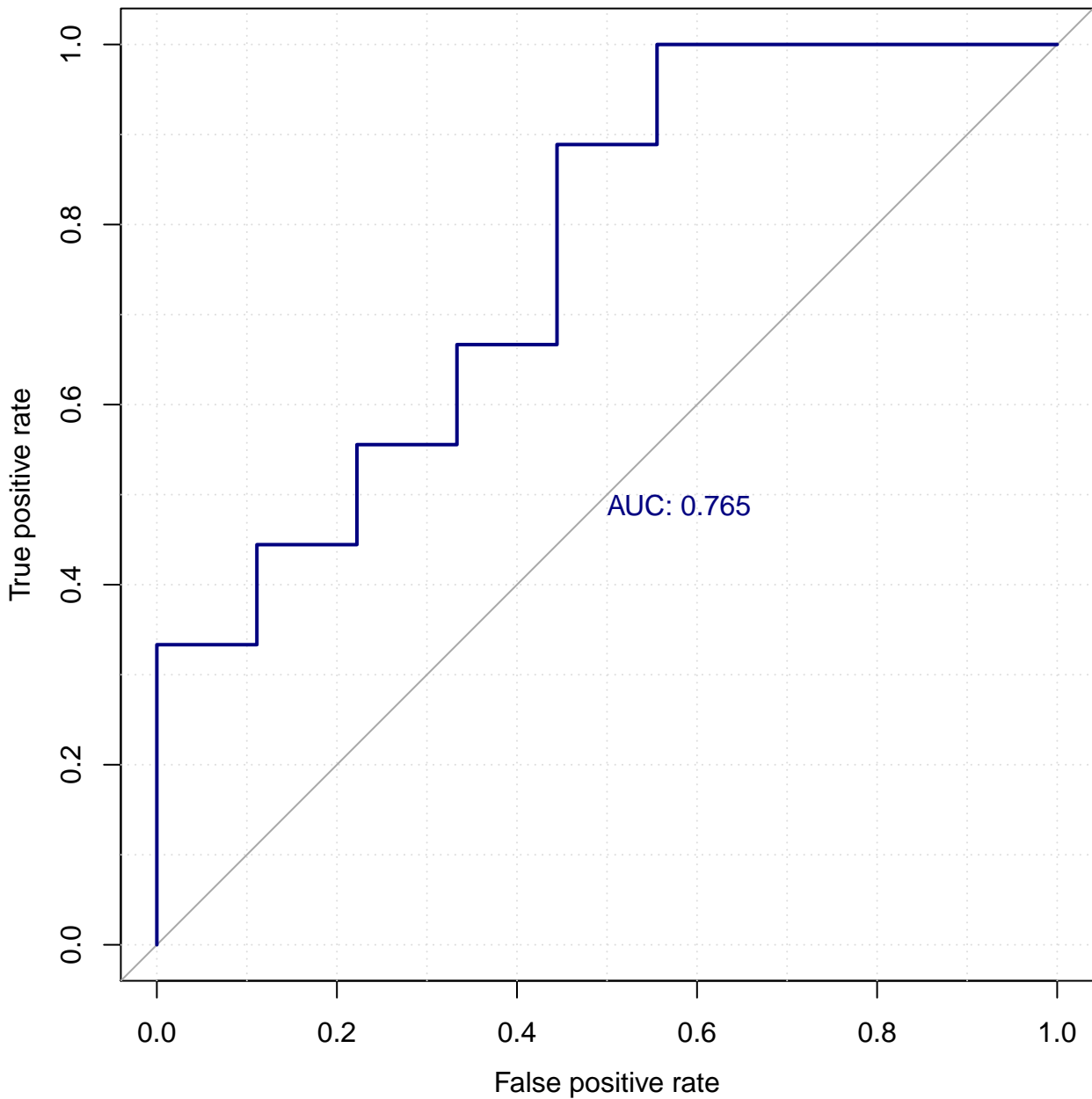

Supplement: Supplemental Information 2 [file peerj-11-15112-s002.zip › peerj-75361-Raw_data_result/Raw data/Result-X101SC21103966-Z01-J001-B1-42/4.MetDiffAnalysis/H.vs.NH/ROC_neg/Com_5237_neg_ROC.pdf]

H.vs.NH

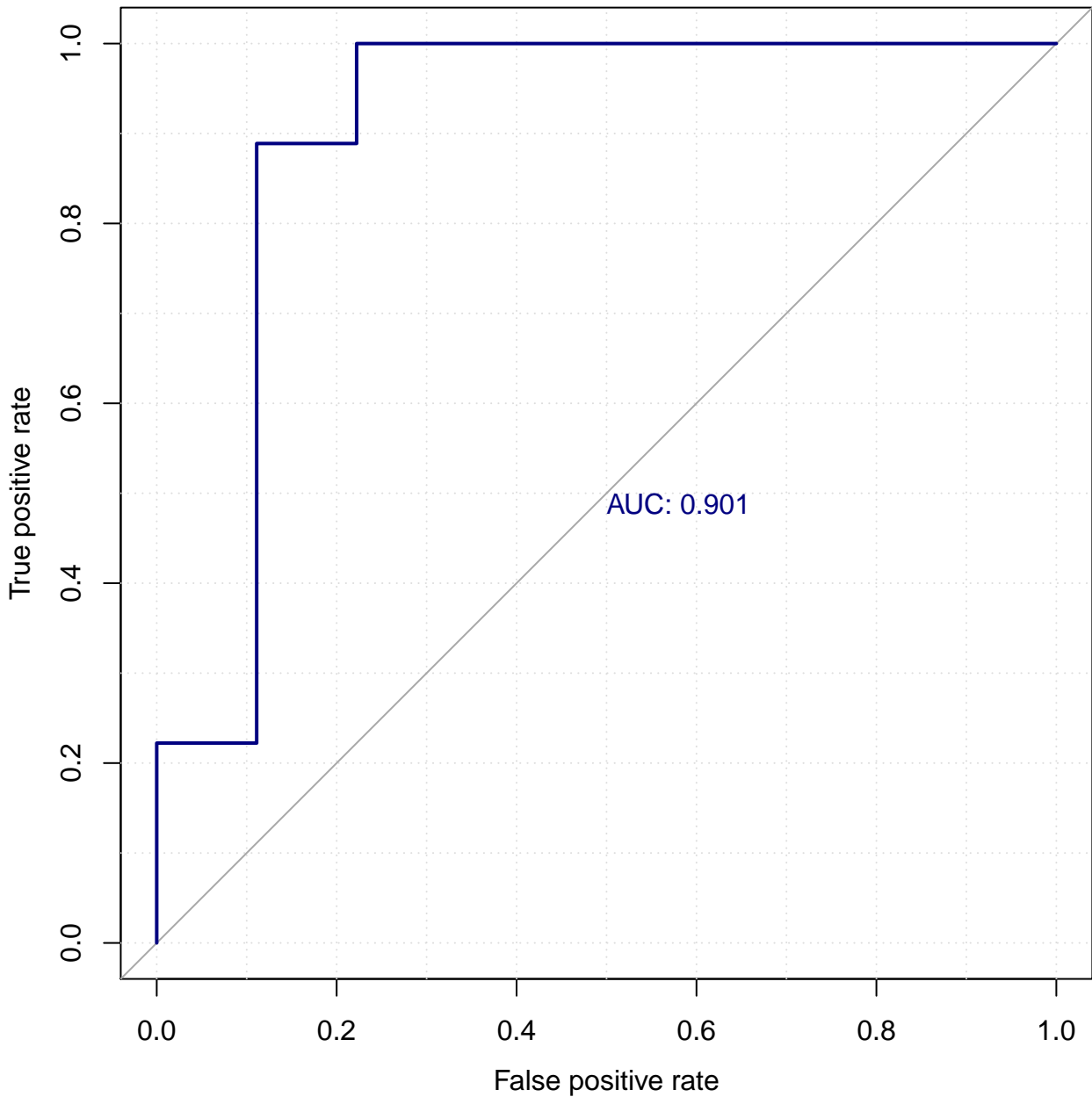

Supplement: Supplemental Information 2 [file peerj-11-15112-s002.zip › peerj-75361-Raw_data_result/Raw data/Result-X101SC21103966-Z01-J001-B1-42/4.MetDiffAnalysis/H.vs.NH/ROC_neg/Com_549_neg_ROC.pdf]

# H.vs.NH

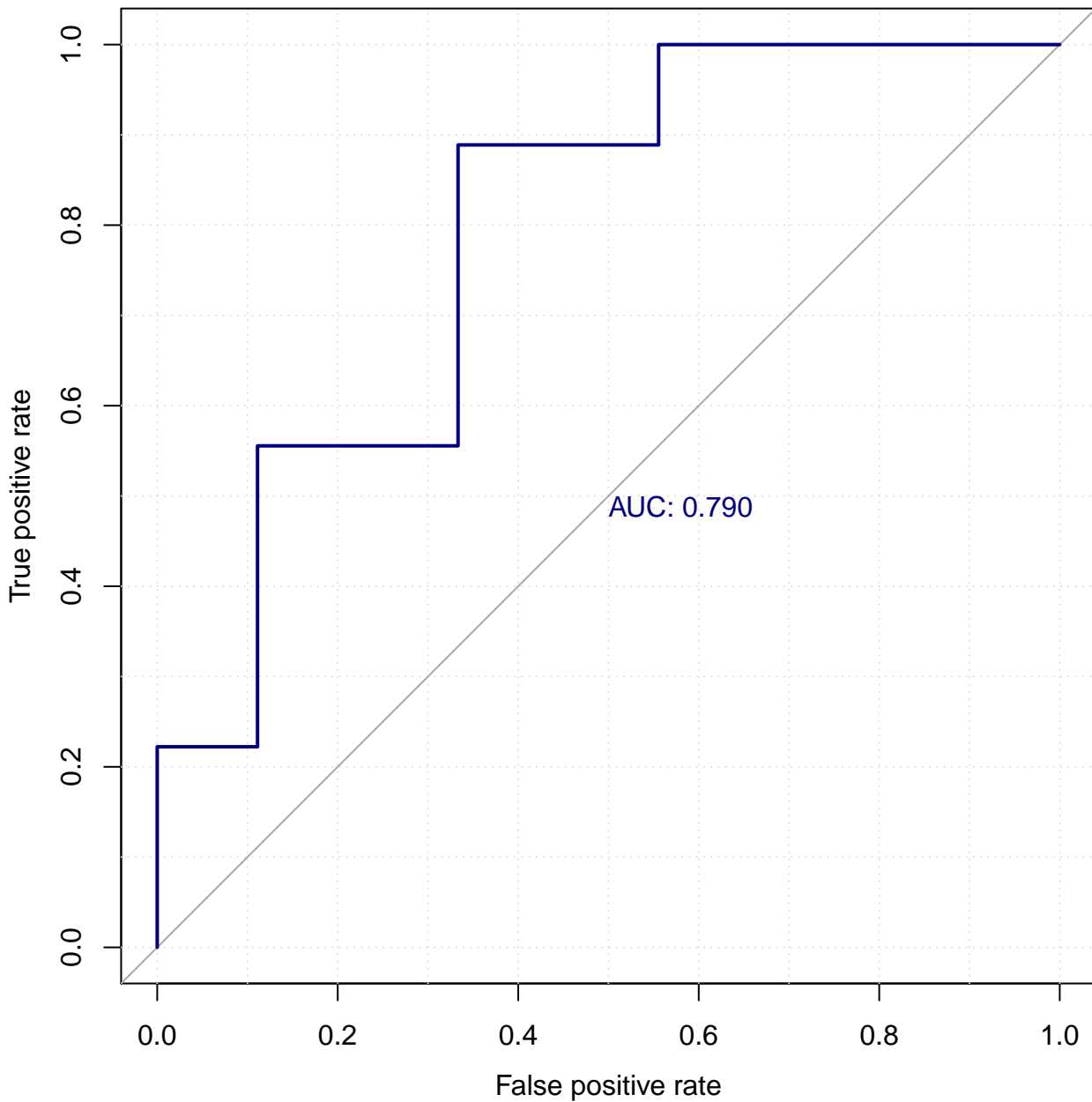

Supplement: Supplemental Information 2 [file peerj-11-15112-s002.zip › peerj-75361-Raw_data_result/Raw data/Result-X101SC21103966-Z01-J001-B1-42/4.MetDiffAnalysis/H.vs.NH/ROC_neg/Com_5747_neg_ROC.pdf]

H.vs.NH

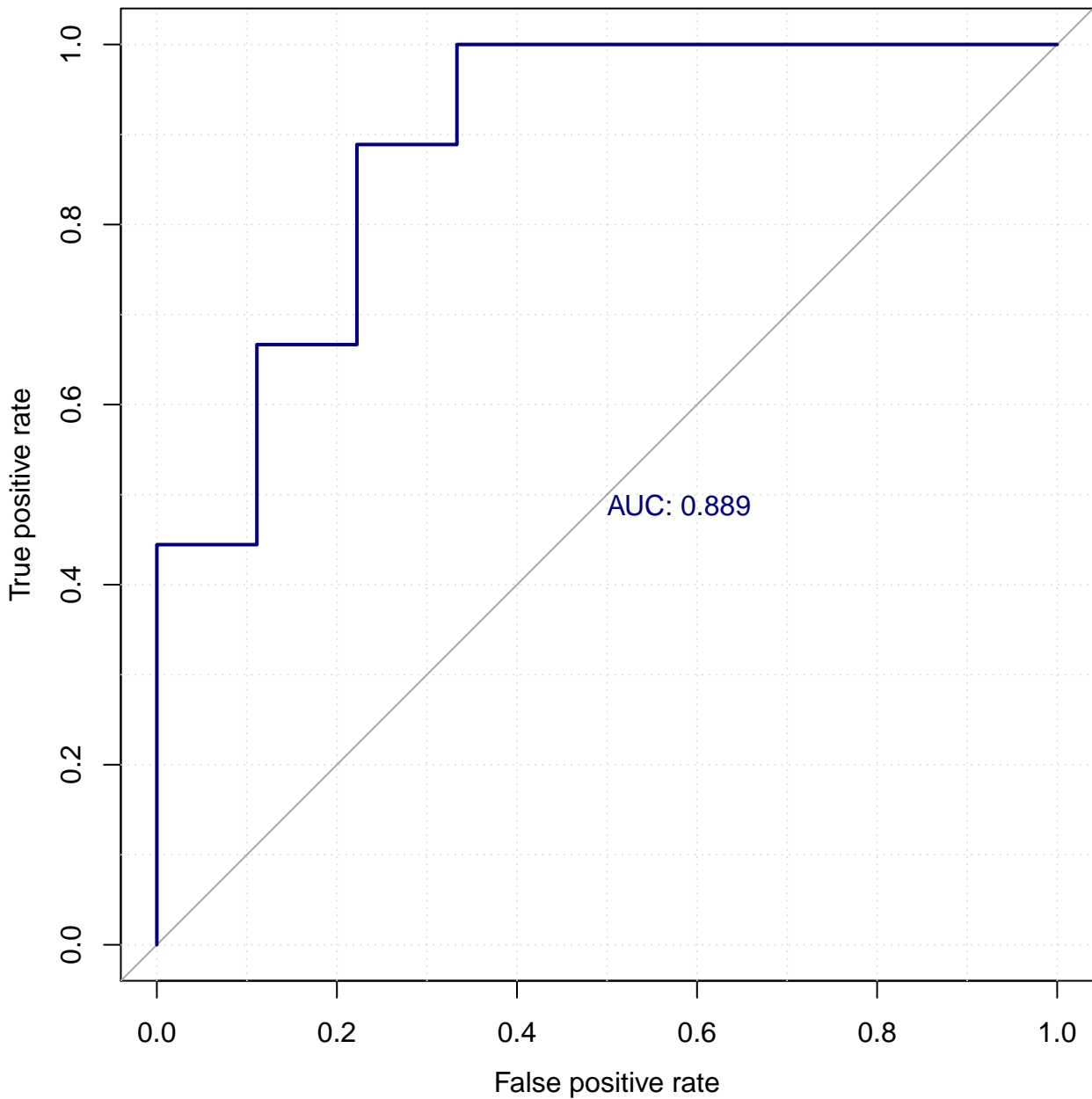

Supplement: Supplemental Information 2 [file peerj-11-15112-s002.zip › peerj-75361-Raw_data_result/Raw data/Result-X101SC21103966-Z01-J001-B1-42/4.MetDiffAnalysis/H.vs.NH/ROC_neg/Com_586_neg_ROC.pdf]

# H.vs.NH

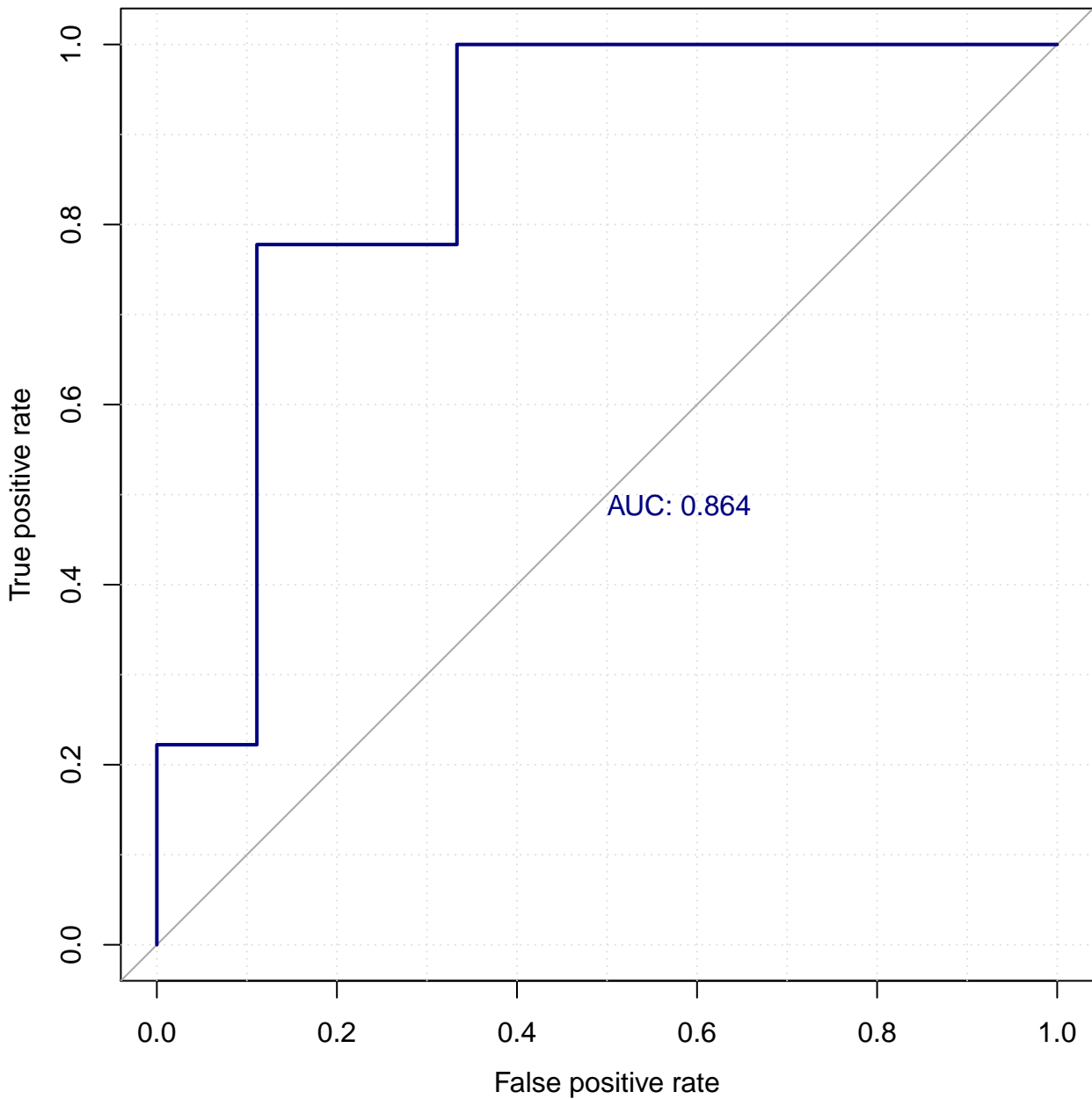

Supplement: Supplemental Information 2 [file peerj-11-15112-s002.zip › peerj-75361-Raw_data_result/Raw data/Result-X101SC21103966-Z01-J001-B1-42/4.MetDiffAnalysis/H.vs.NH/ROC_neg/Com_6699_neg_ROC.pdf]

# H.vs.NH

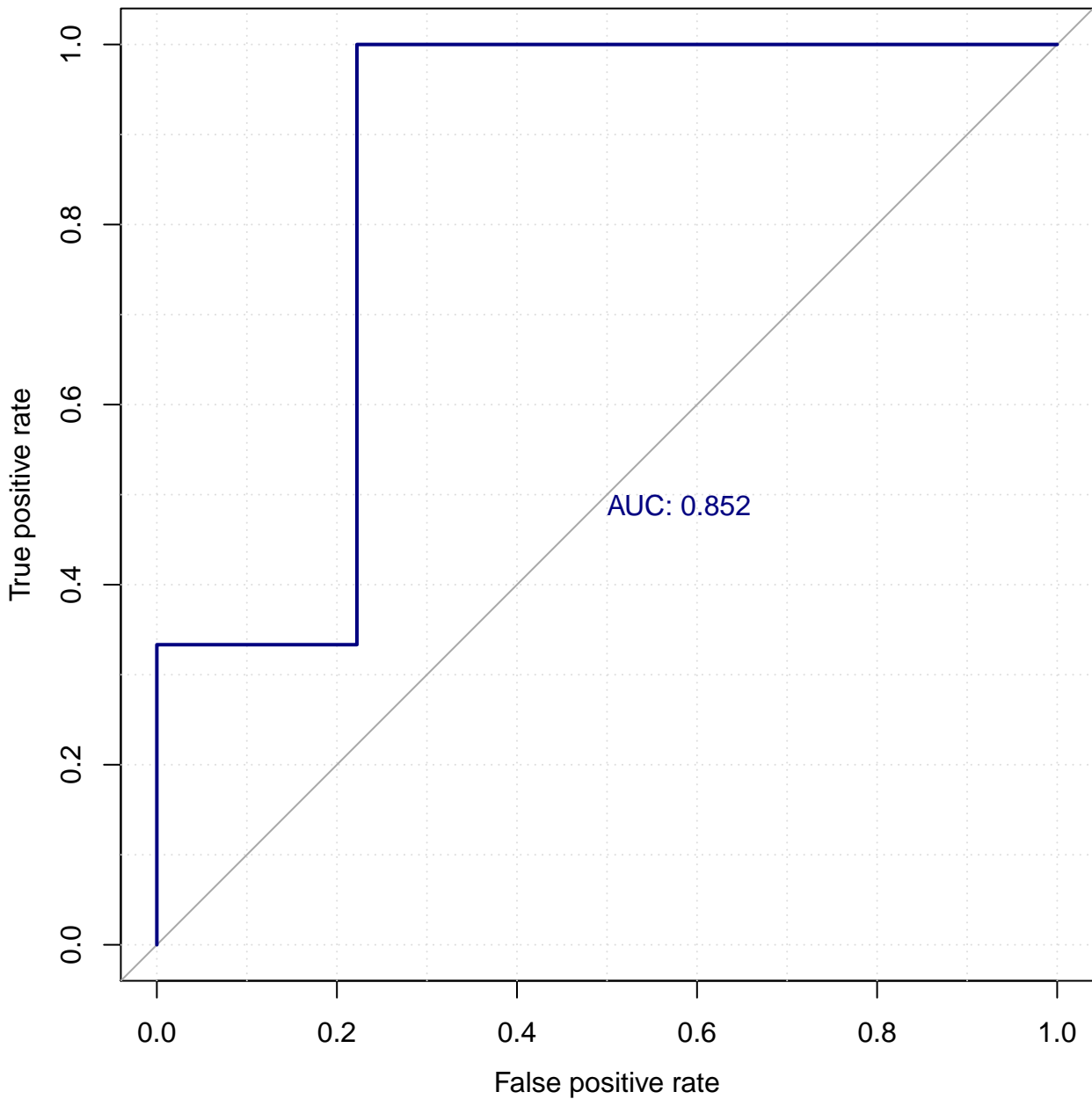

Supplement: Supplemental Information 2 [file peerj-11-15112-s002.zip › peerj-75361-Raw_data_result/Raw data/Result-X101SC21103966-Z01-J001-B1-42/4.MetDiffAnalysis/H.vs.NH/ROC_neg/Com_754_neg_ROC.pdf]

# H.vs.NH

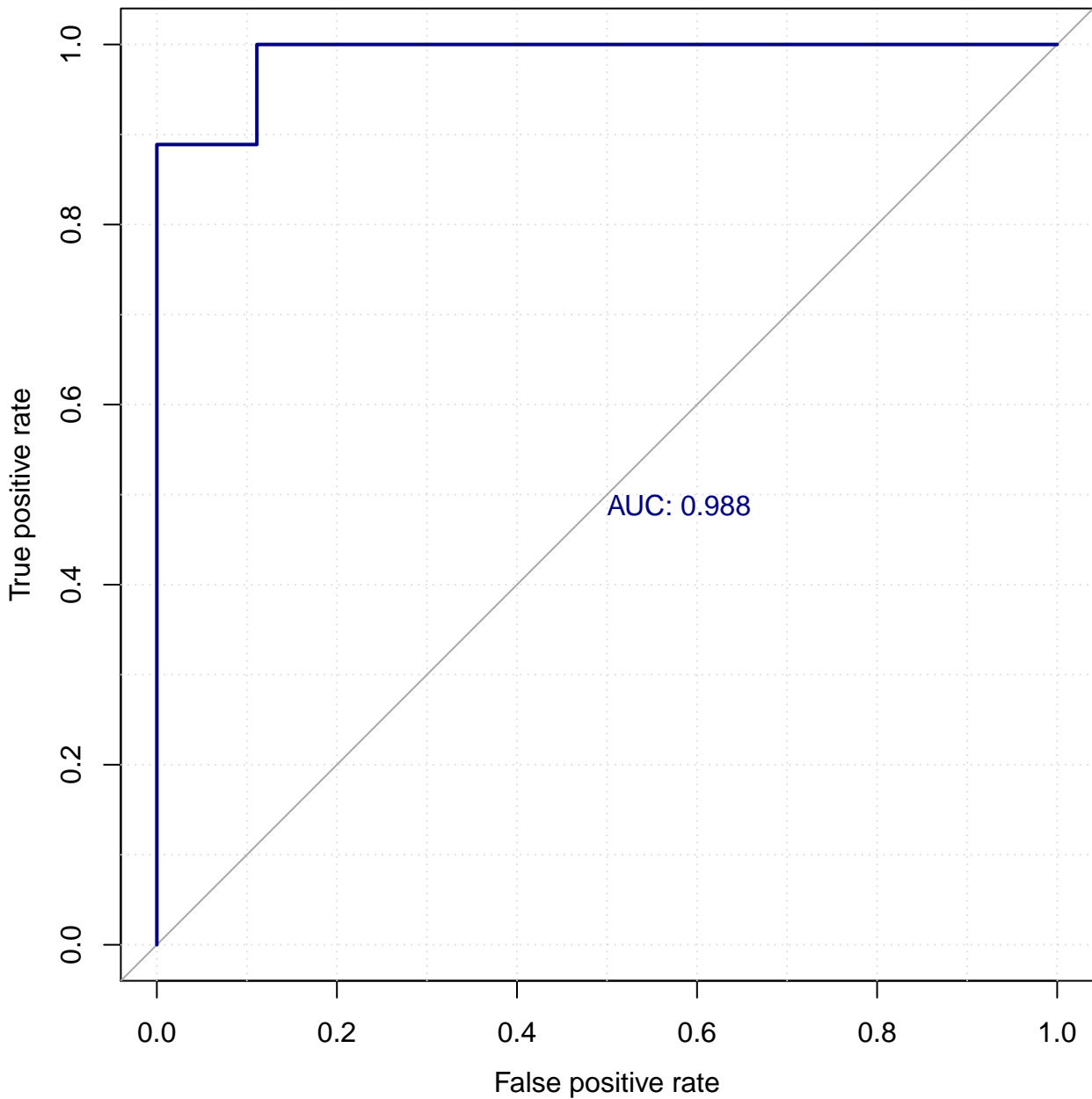

Supplement: Supplemental Information 2 [file peerj-11-15112-s002.zip › peerj-75361-Raw_data_result/Raw data/Result-X101SC21103966-Z01-J001-B1-42/4.MetDiffAnalysis/H.vs.NH/ROC_neg/Com_7747_neg_ROC.pdf]

# H.vs.NH

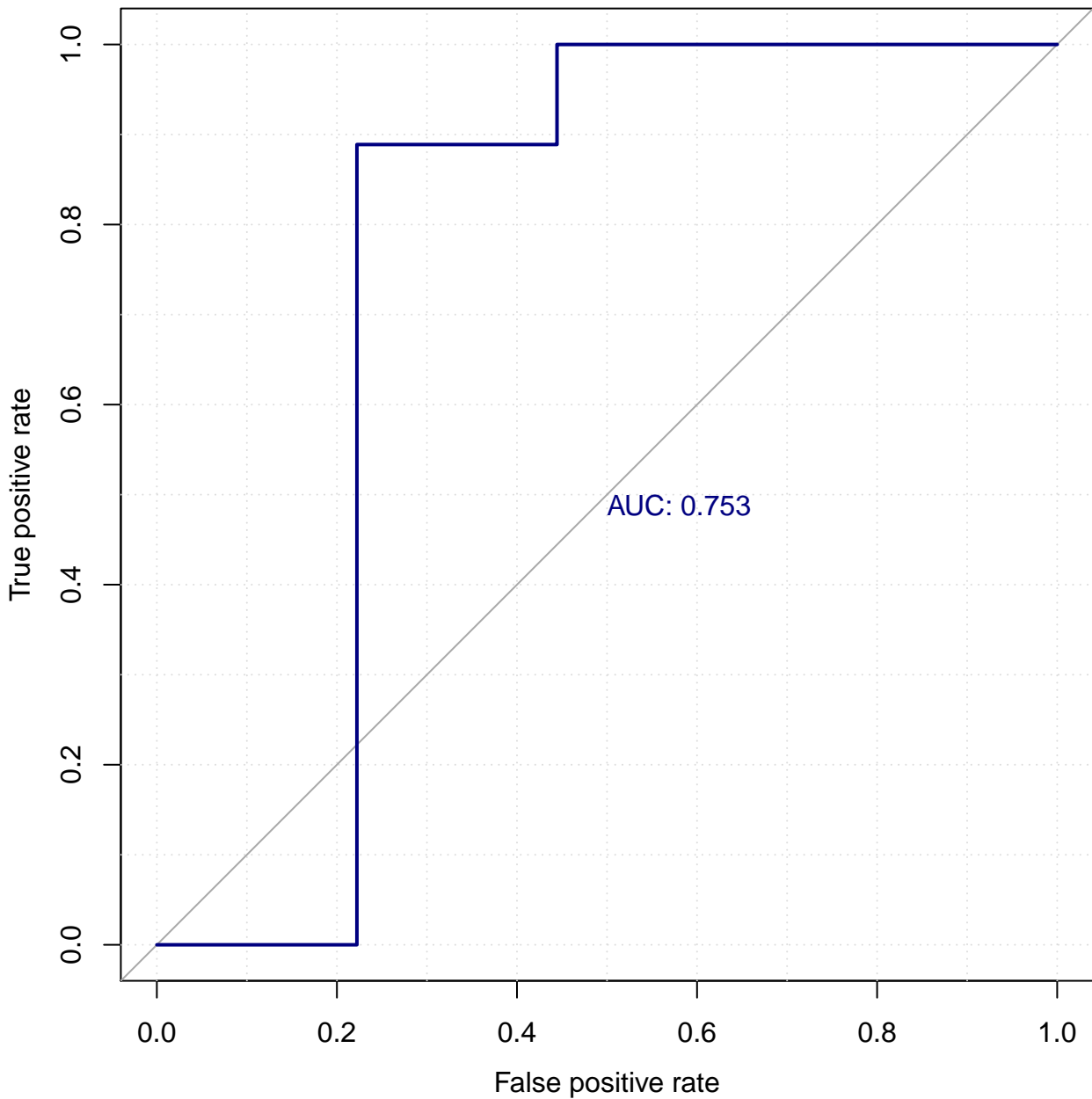

Supplement: Supplemental Information 2 [file peerj-11-15112-s002.zip › peerj-75361-Raw_data_result/Raw data/Result-X101SC21103966-Z01-J001-B1-42/4.MetDiffAnalysis/H.vs.NH/ROC_neg/Com_87_neg_ROC.pdf]

H.vs.NH

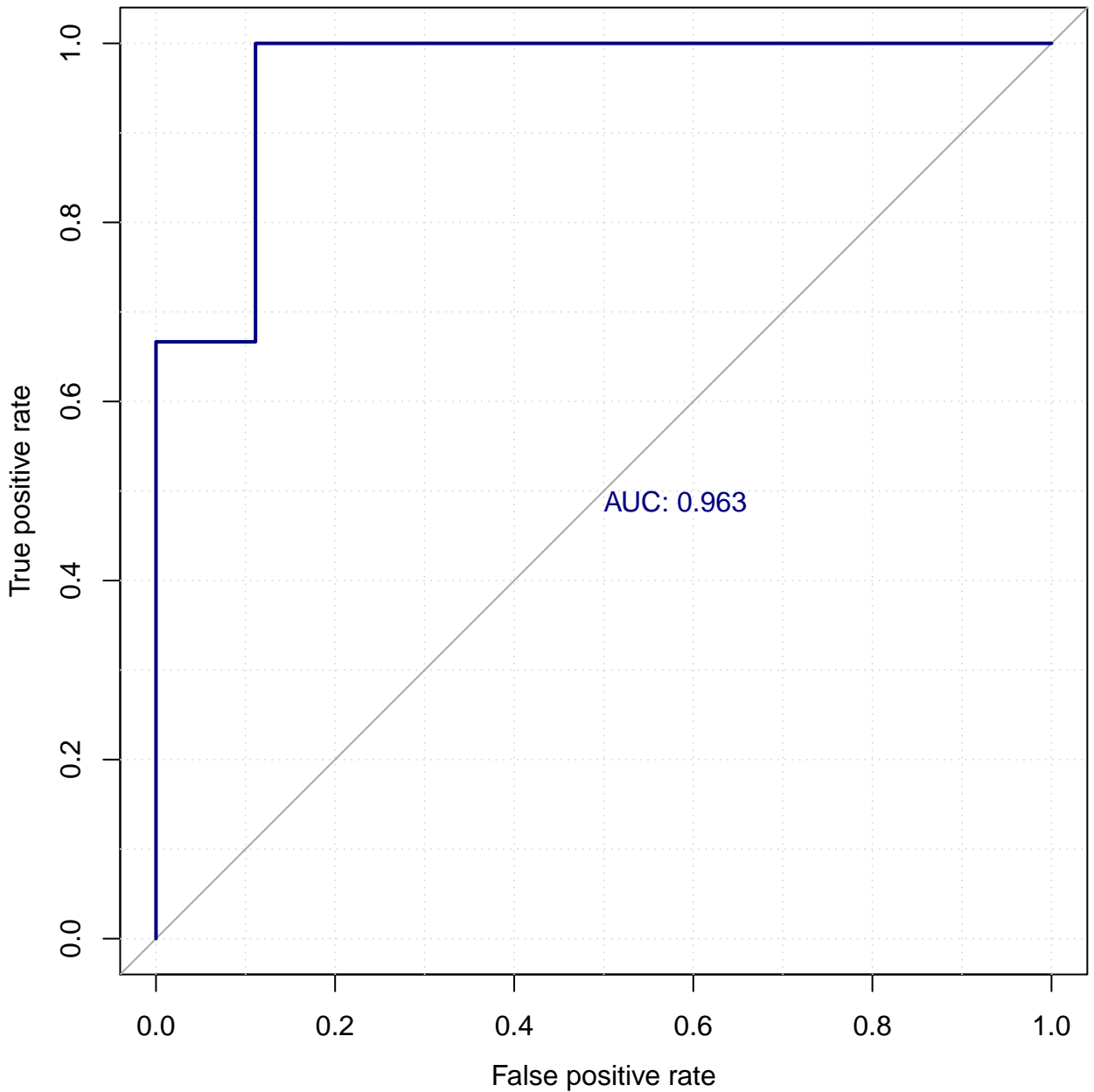

Supplement: Supplemental Information 2 [file peerj-11-15112-s002.zip › peerj-75361-Raw_data_result/Raw data/Result-X101SC21103966-Z01-J001-B1-42/4.MetDiffAnalysis/H.vs.NH/ROC_neg/Com_9816_neg_ROC.pdf]

# H.vs.NH

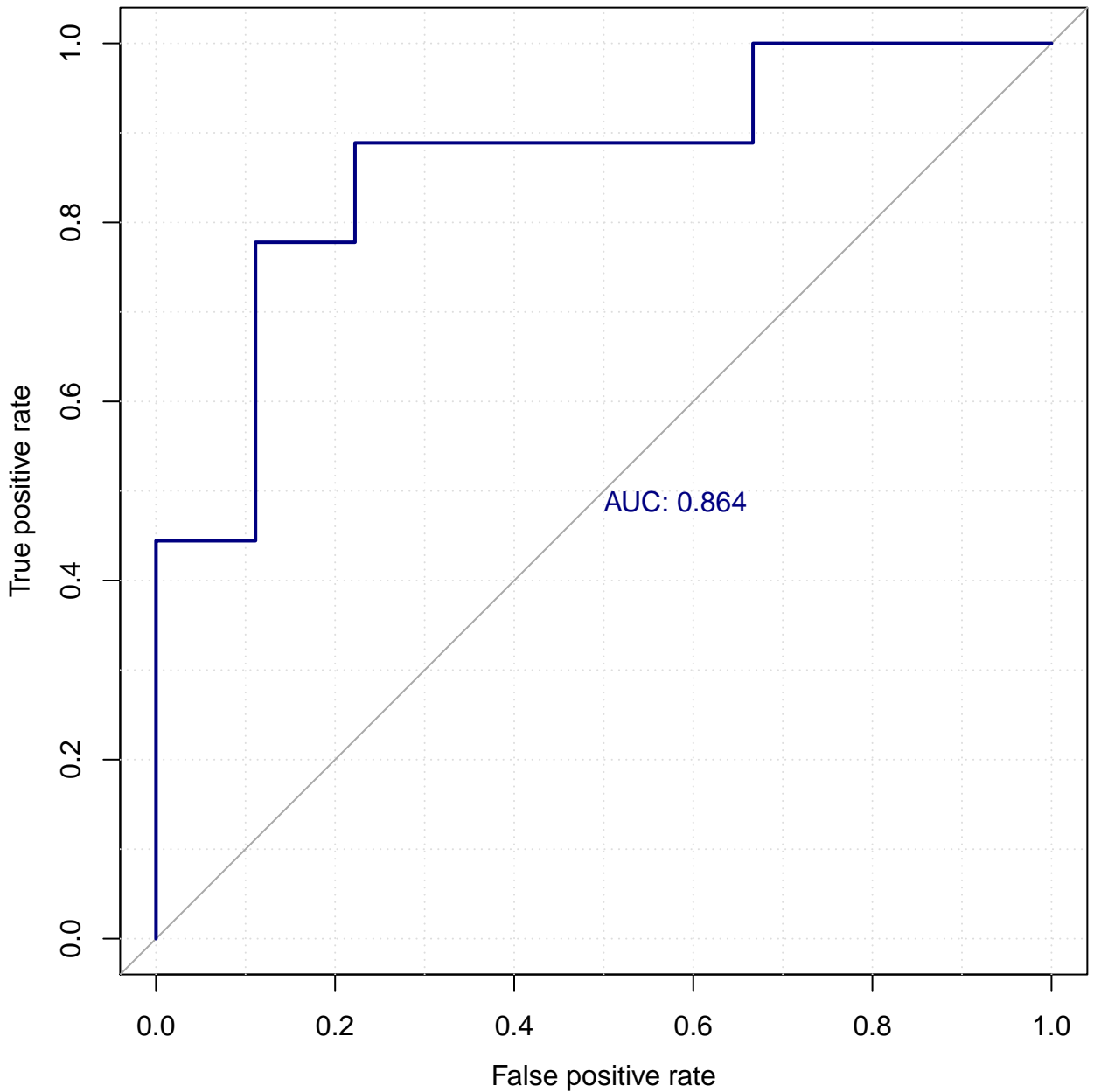

Supplement: Supplemental Information 2 [file peerj-11-15112-s002.zip › peerj-75361-Raw_data_result/Raw data/Result-X101SC21103966-Z01-J001-B1-42/4.MetDiffAnalysis/H.vs.NH/ROC_pos/Com_10042_pos_ROC.pdf]

# H.vs.NH

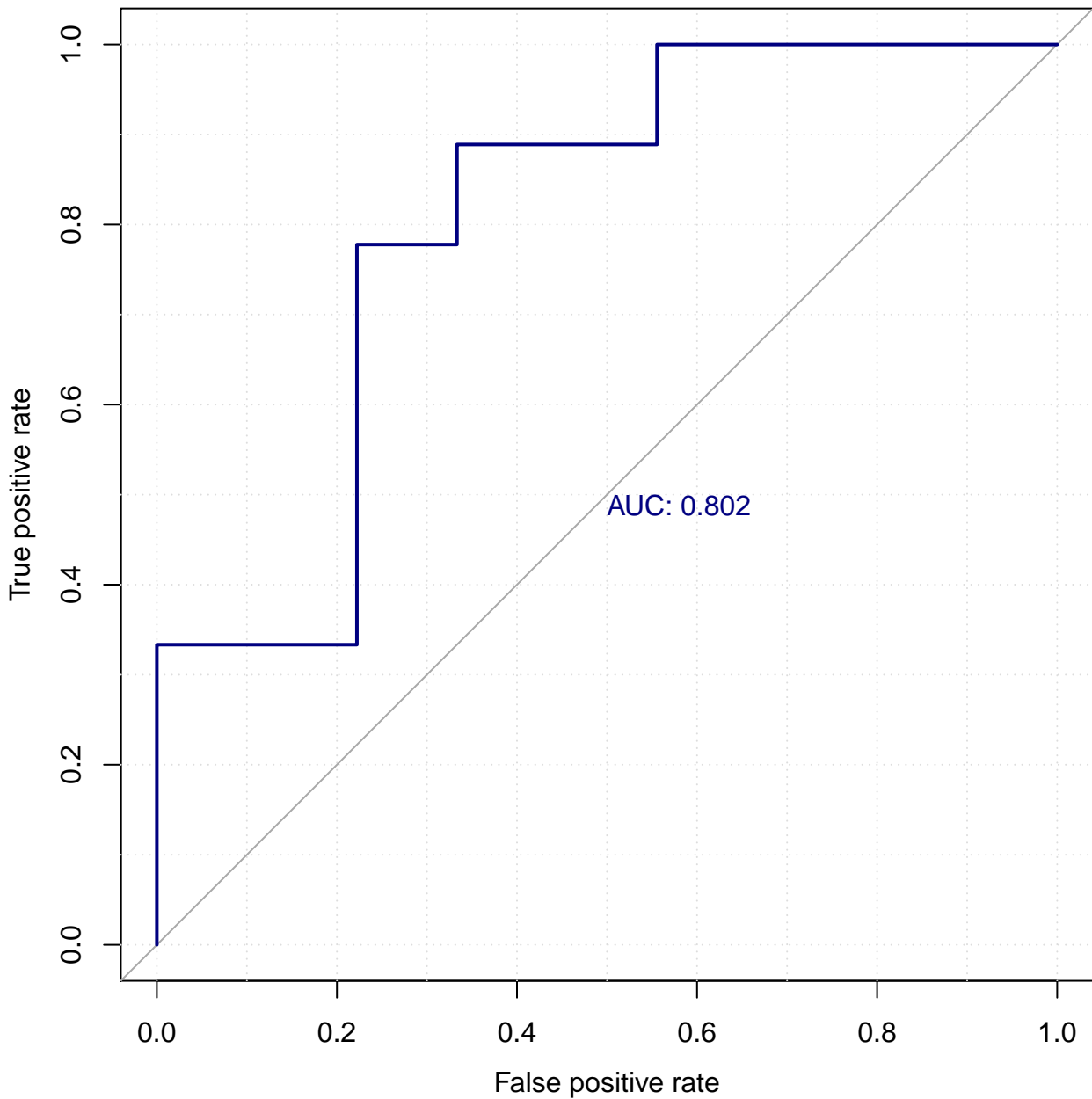

Supplement: Supplemental Information 2 [file peerj-11-15112-s002.zip › peerj-75361-Raw_data_result/Raw data/Result-X101SC21103966-Z01-J001-B1-42/4.MetDiffAnalysis/H.vs.NH/ROC_pos/Com_10634_pos_ROC.pdf]

# H.vs.NH

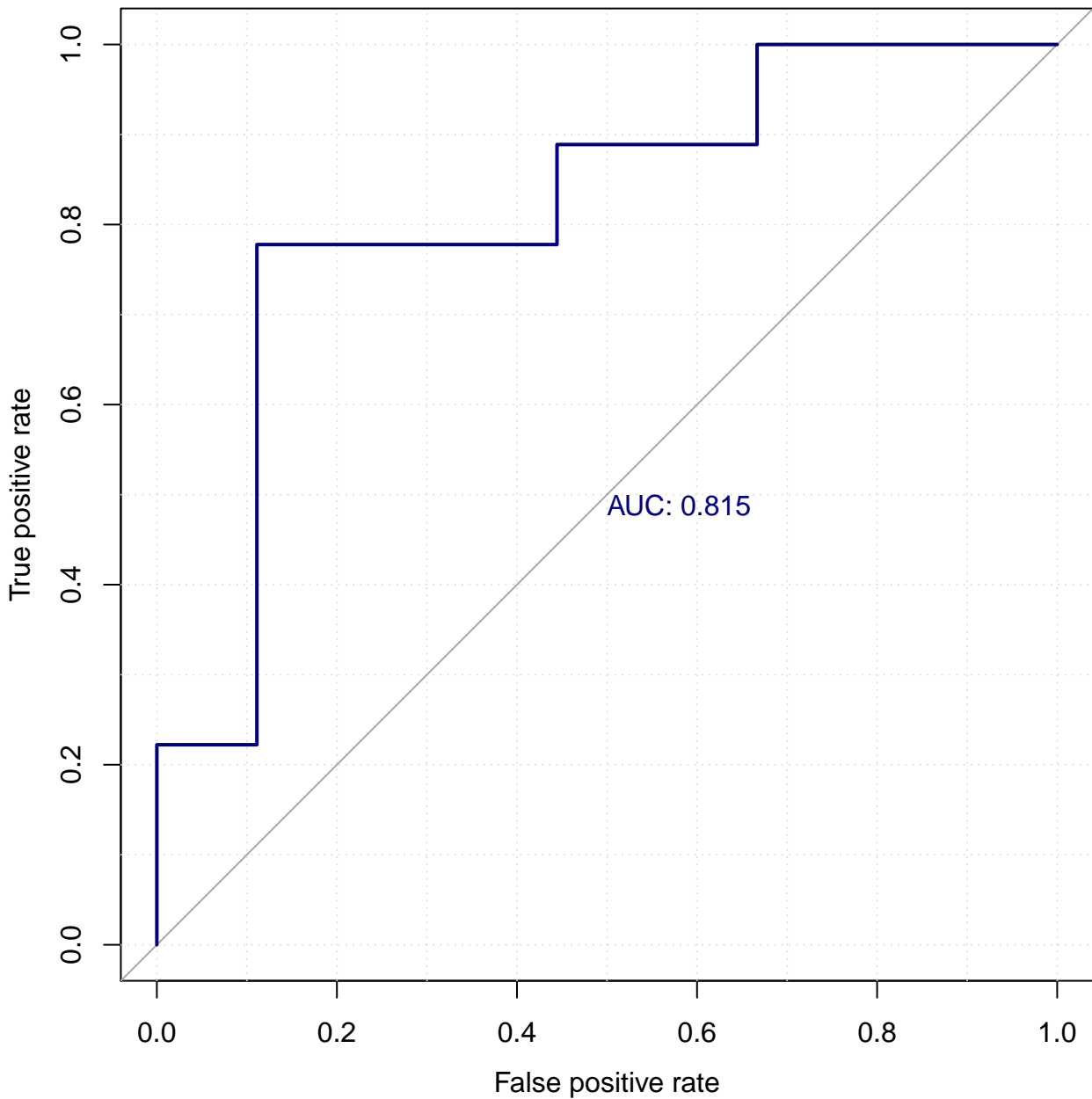

Supplement: Supplemental Information 2 [file peerj-11-15112-s002.zip › peerj-75361-Raw_data_result/Raw data/Result-X101SC21103966-Z01-J001-B1-42/4.MetDiffAnalysis/H.vs.NH/ROC_pos/Com_11056_pos_ROC.pdf]

H.vs.NH

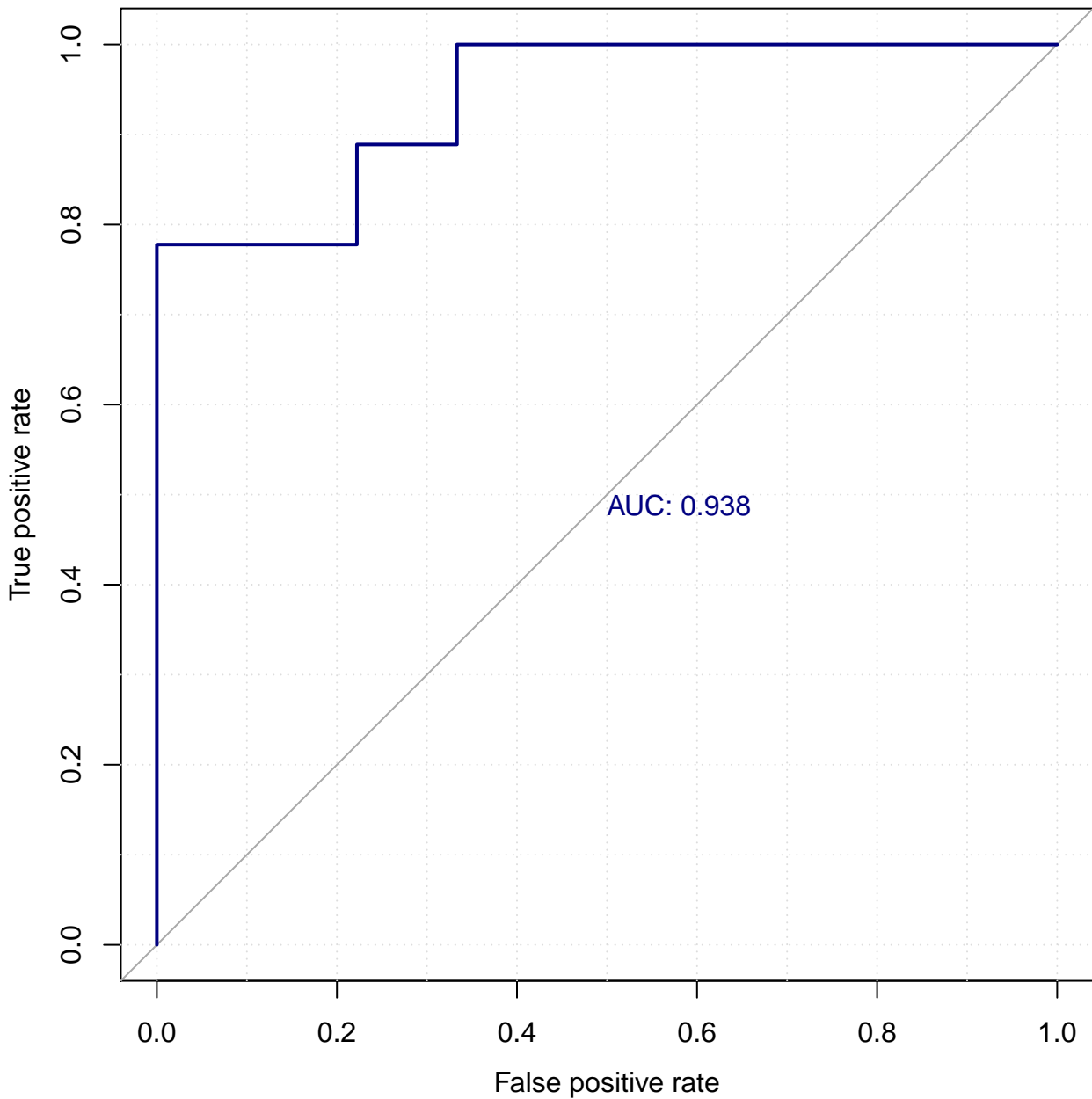

Supplement: Supplemental Information 2 [file peerj-11-15112-s002.zip › peerj-75361-Raw_data_result/Raw data/Result-X101SC21103966-Z01-J001-B1-42/4.MetDiffAnalysis/H.vs.NH/ROC_pos/Com_1138_pos_ROC.pdf]

H.vs.NH

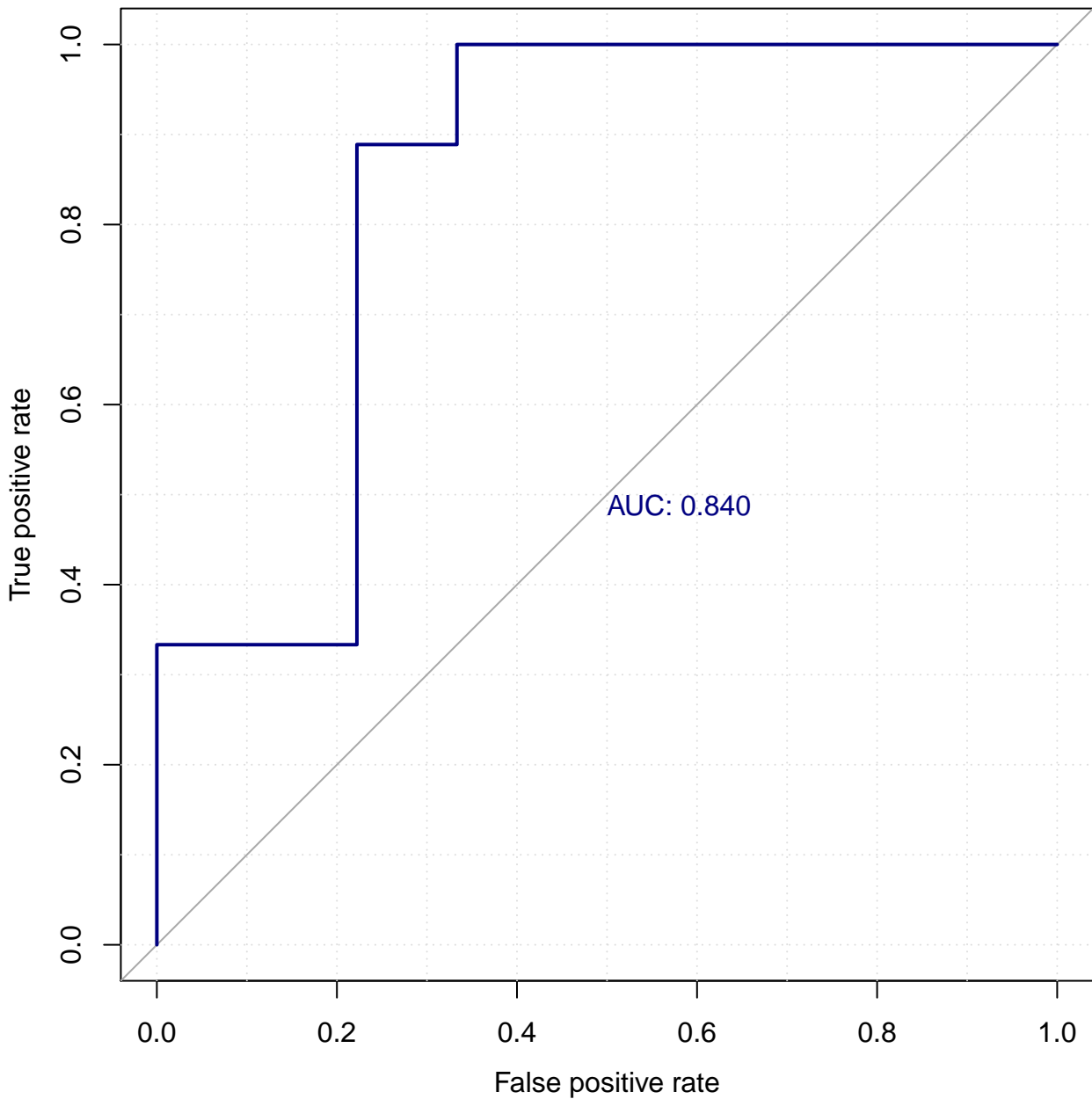

Supplement: Supplemental Information 2 [file peerj-11-15112-s002.zip › peerj-75361-Raw_data_result/Raw data/Result-X101SC21103966-Z01-J001-B1-42/4.MetDiffAnalysis/H.vs.NH/ROC_pos/Com_1426_pos_ROC.pdf]

# H.vs.NH

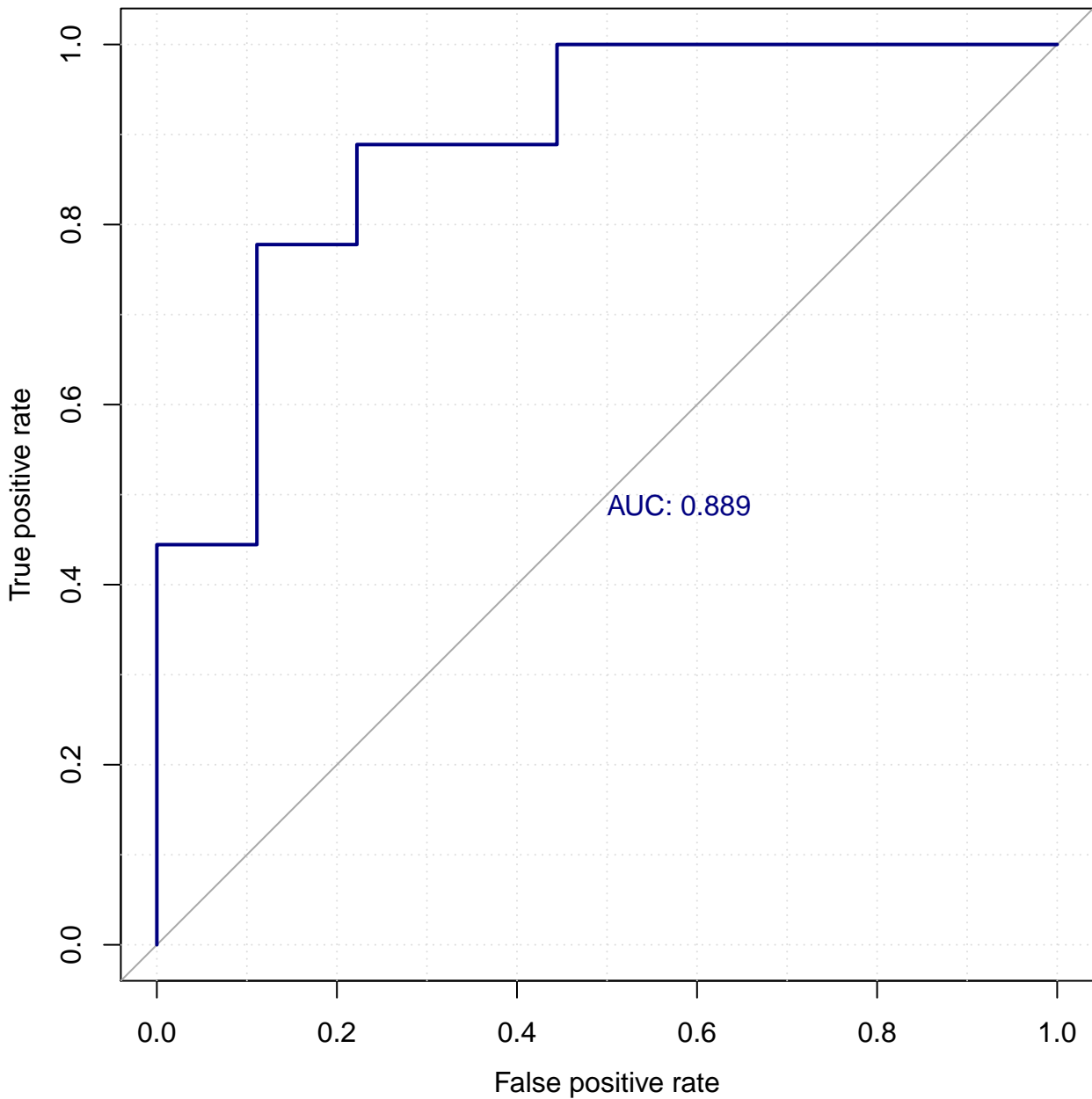

Supplement: Supplemental Information 2 [file peerj-11-15112-s002.zip › peerj-75361-Raw_data_result/Raw data/Result-X101SC21103966-Z01-J001-B1-42/4.MetDiffAnalysis/H.vs.NH/ROC_pos/Com_156_pos_ROC.pdf]

H.vs.NH

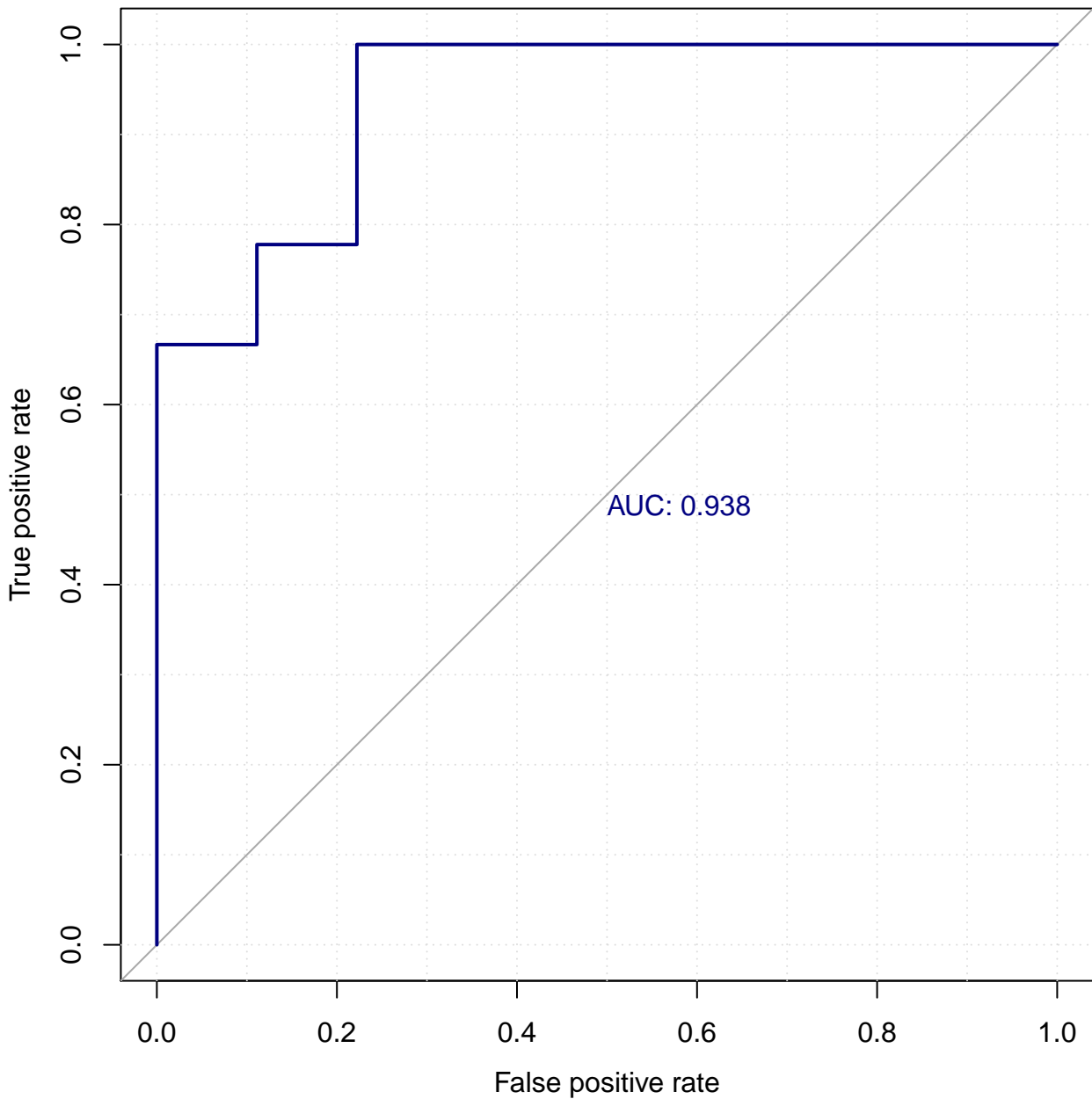

Supplement: Supplemental Information 2 [file peerj-11-15112-s002.zip › peerj-75361-Raw_data_result/Raw data/Result-X101SC21103966-Z01-J001-B1-42/4.MetDiffAnalysis/H.vs.NH/ROC_pos/Com_1621_pos_ROC.pdf]

# H.vs.NH

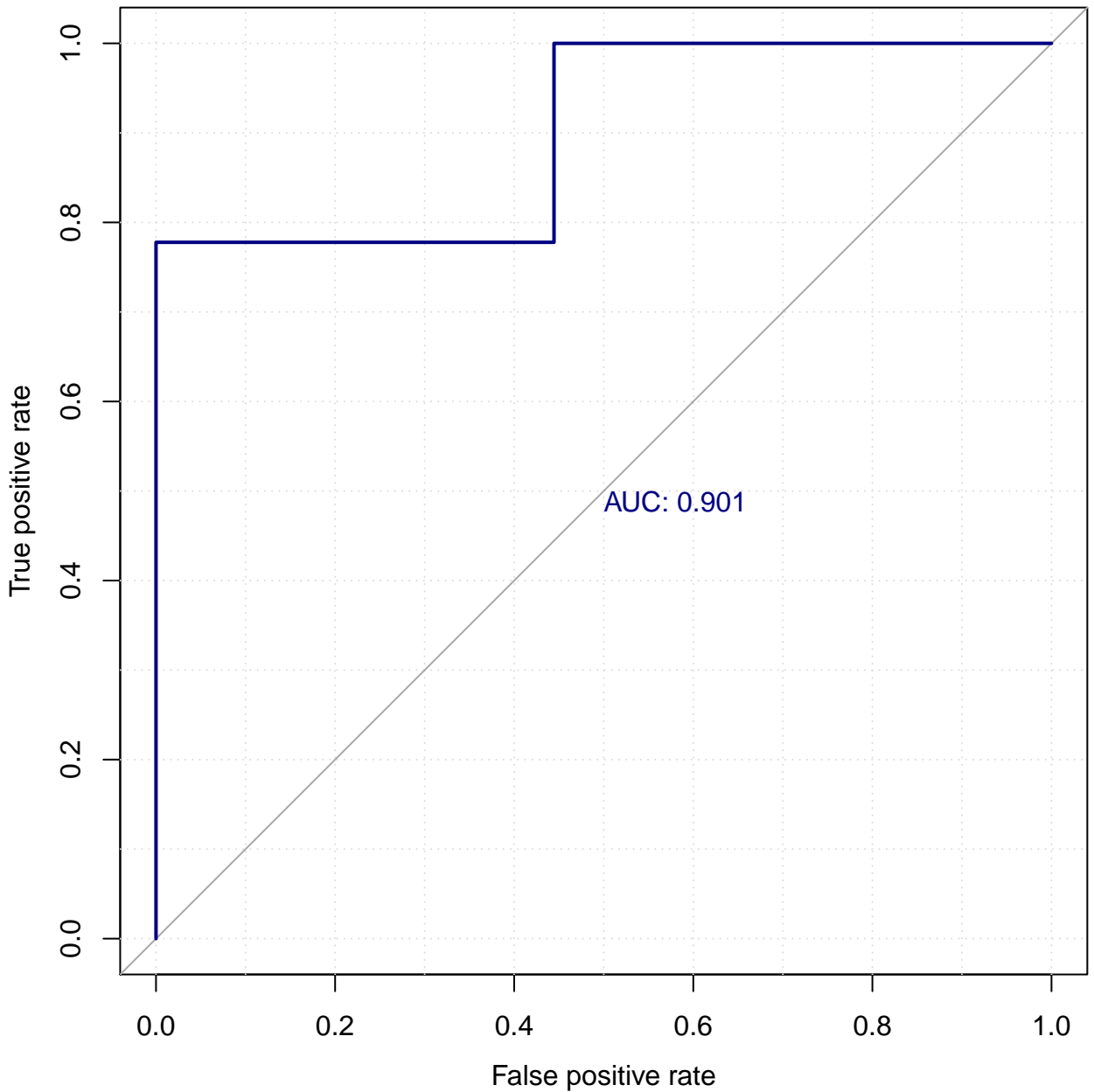

Supplement: Supplemental Information 2 [file peerj-11-15112-s002.zip › peerj-75361-Raw_data_result/Raw data/Result-X101SC21103966-Z01-J001-B1-42/4.MetDiffAnalysis/H.vs.NH/ROC_pos/Com_1664_pos_ROC.pdf]

H.vs.NH

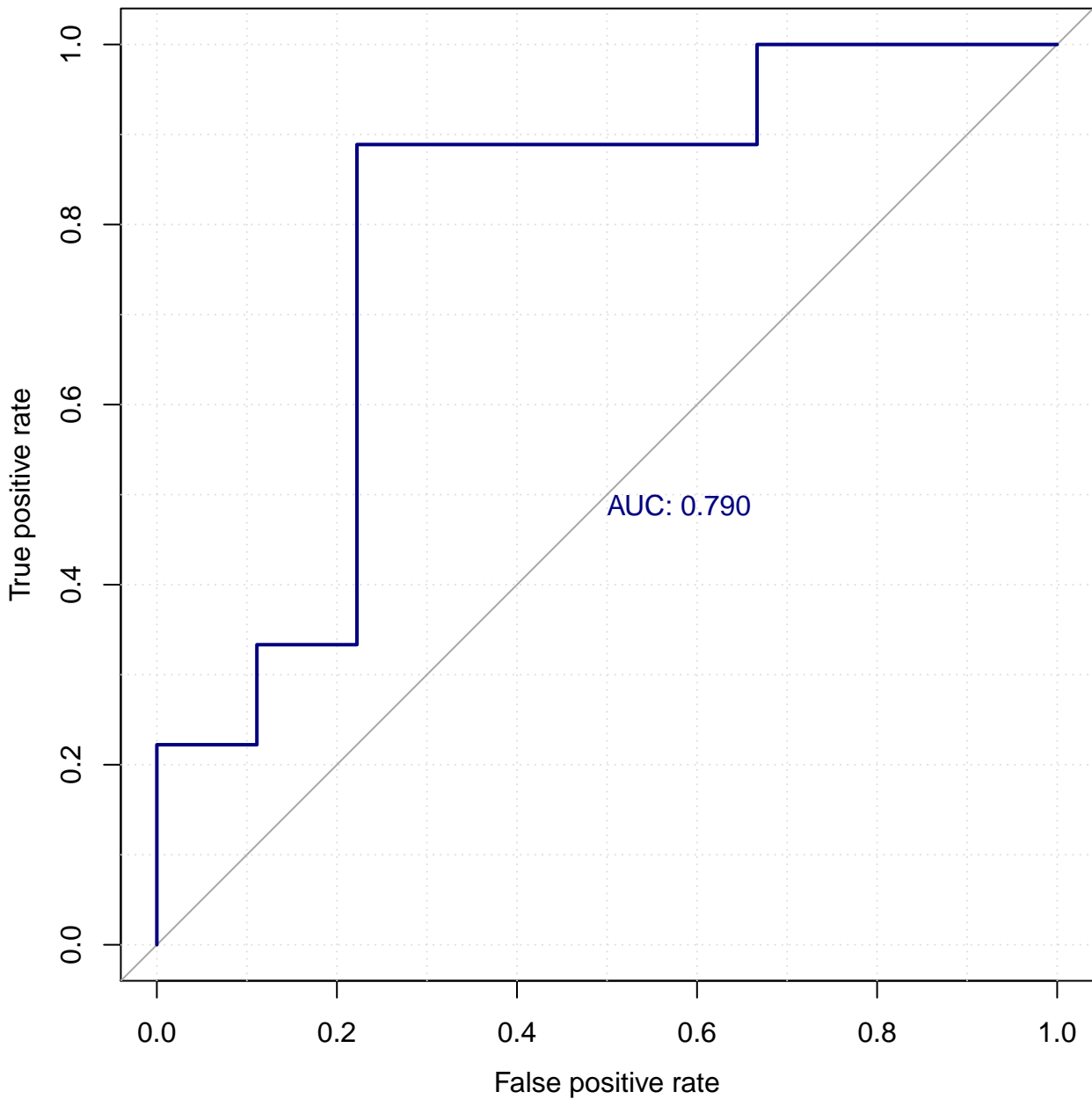

Supplement: Supplemental Information 2 [file peerj-11-15112-s002.zip › peerj-75361-Raw_data_result/Raw data/Result-X101SC21103966-Z01-J001-B1-42/4.MetDiffAnalysis/H.vs.NH/ROC_pos/Com_1852_pos_ROC.pdf]

# H.vs.NH

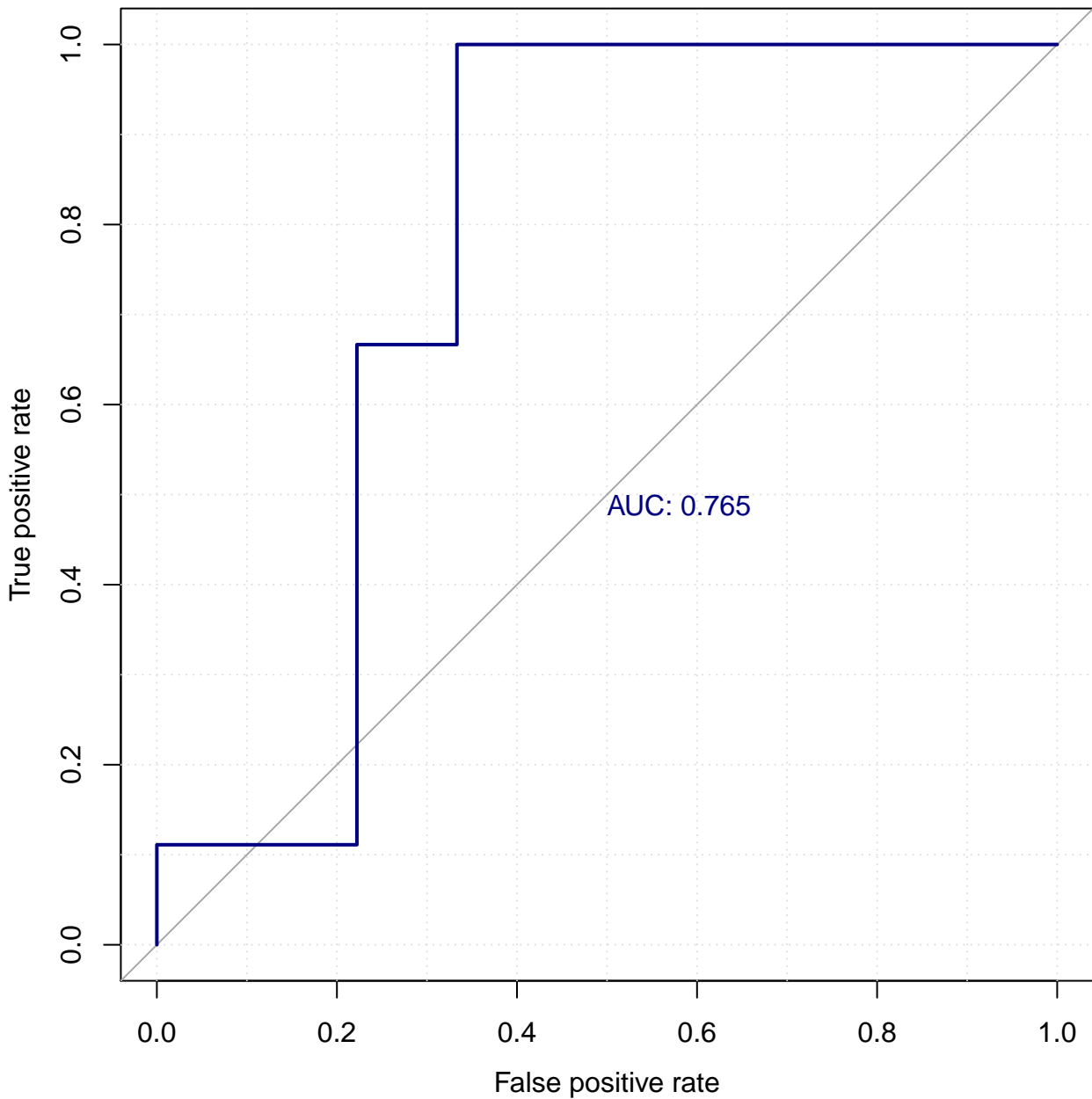

Supplement: Supplemental Information 2 [file peerj-11-15112-s002.zip › peerj-75361-Raw_data_result/Raw data/Result-X101SC21103966-Z01-J001-B1-42/4.MetDiffAnalysis/H.vs.NH/ROC_pos/Com_1894_pos_ROC.pdf]

# H.vs.NH

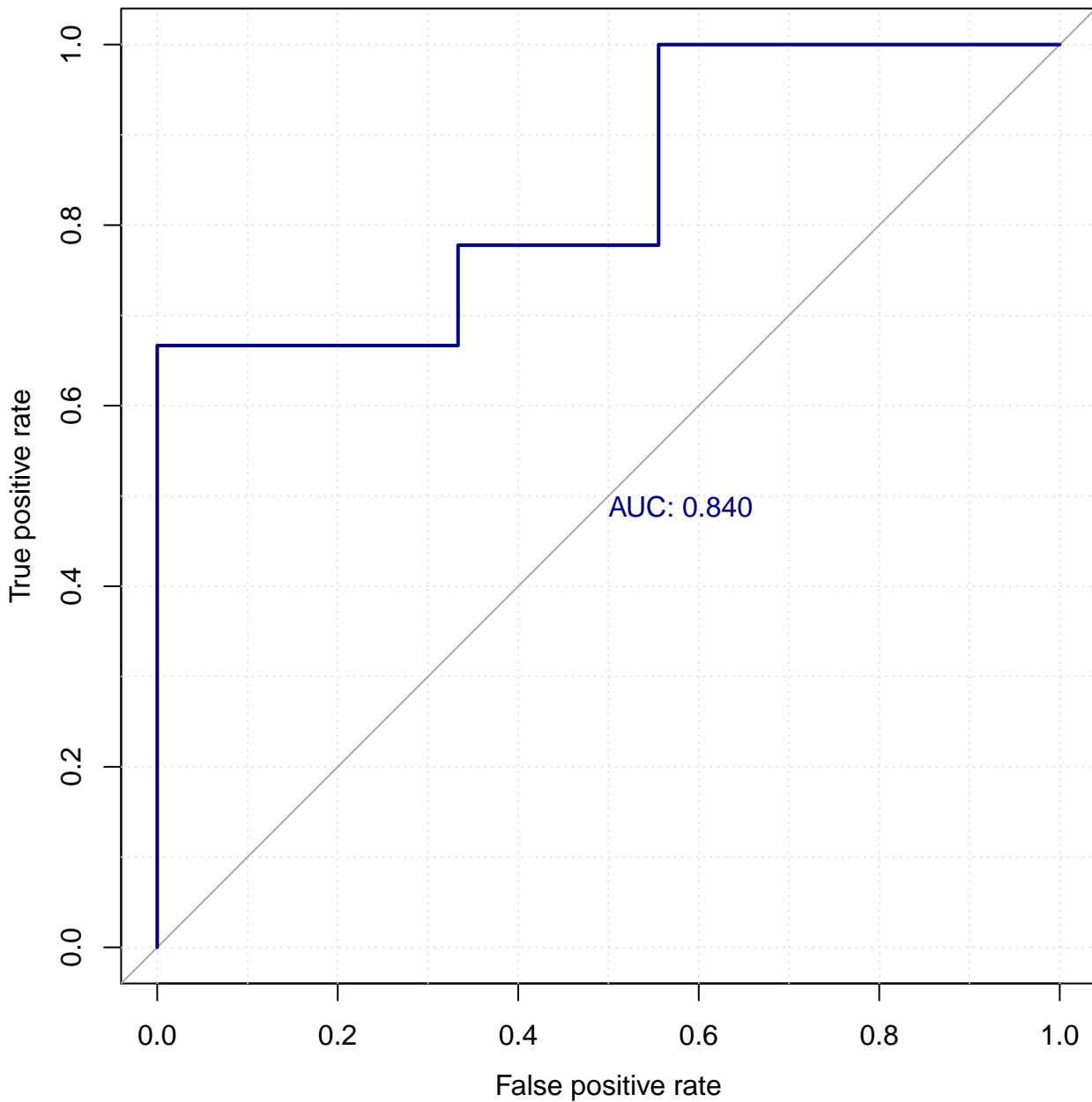

Supplement: Supplemental Information 2 [file peerj-11-15112-s002.zip › peerj-75361-Raw_data_result/Raw data/Result-X101SC21103966-Z01-J001-B1-42/4.MetDiffAnalysis/H.vs.NH/ROC_pos/Com_201_pos_ROC.pdf]

# H.vs.NH

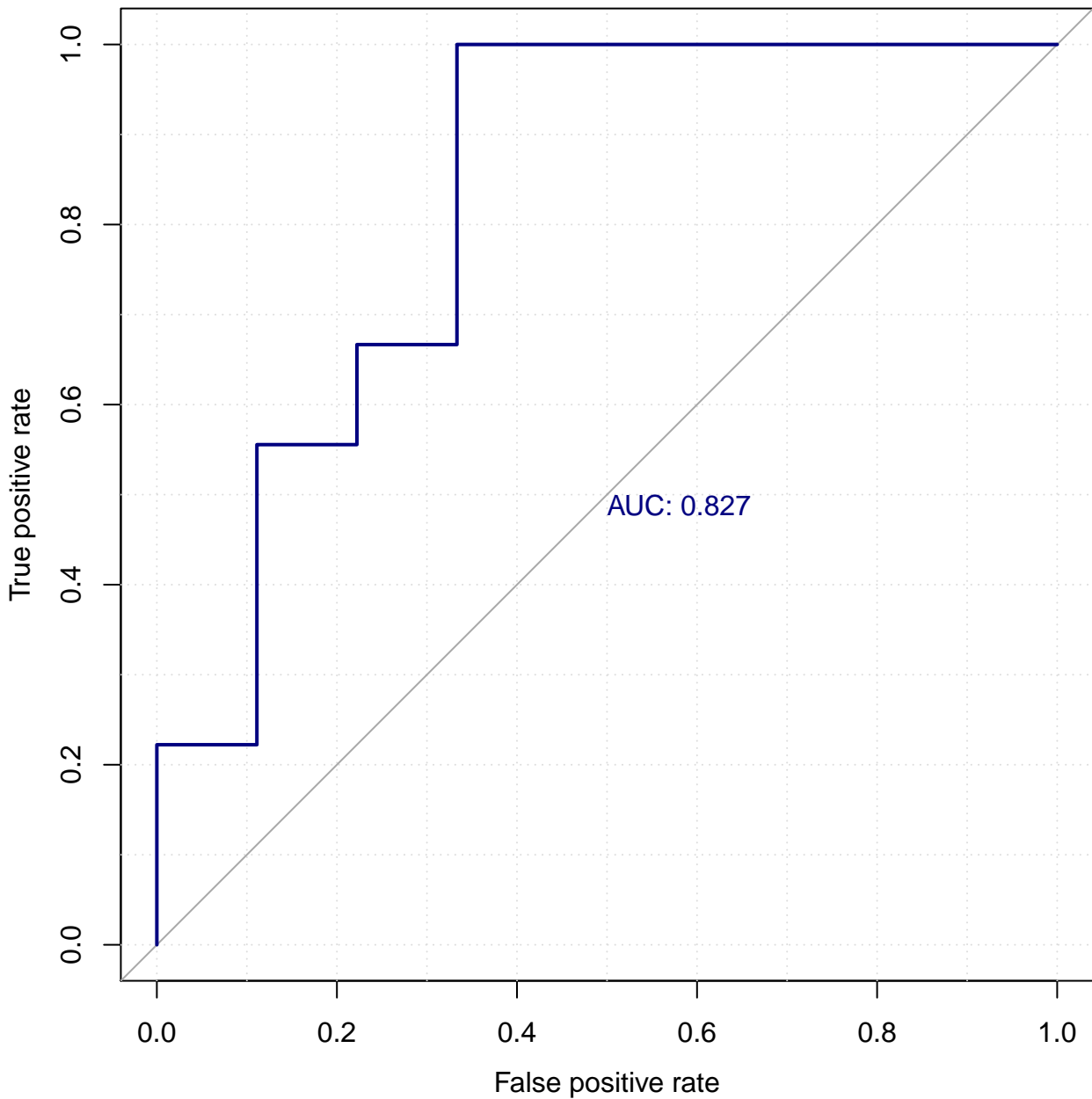

Supplement: Supplemental Information 2 [file peerj-11-15112-s002.zip › peerj-75361-Raw_data_result/Raw data/Result-X101SC21103966-Z01-J001-B1-42/4.MetDiffAnalysis/H.vs.NH/ROC_pos/Com_216_pos_ROC.pdf]

# H.vs.NH

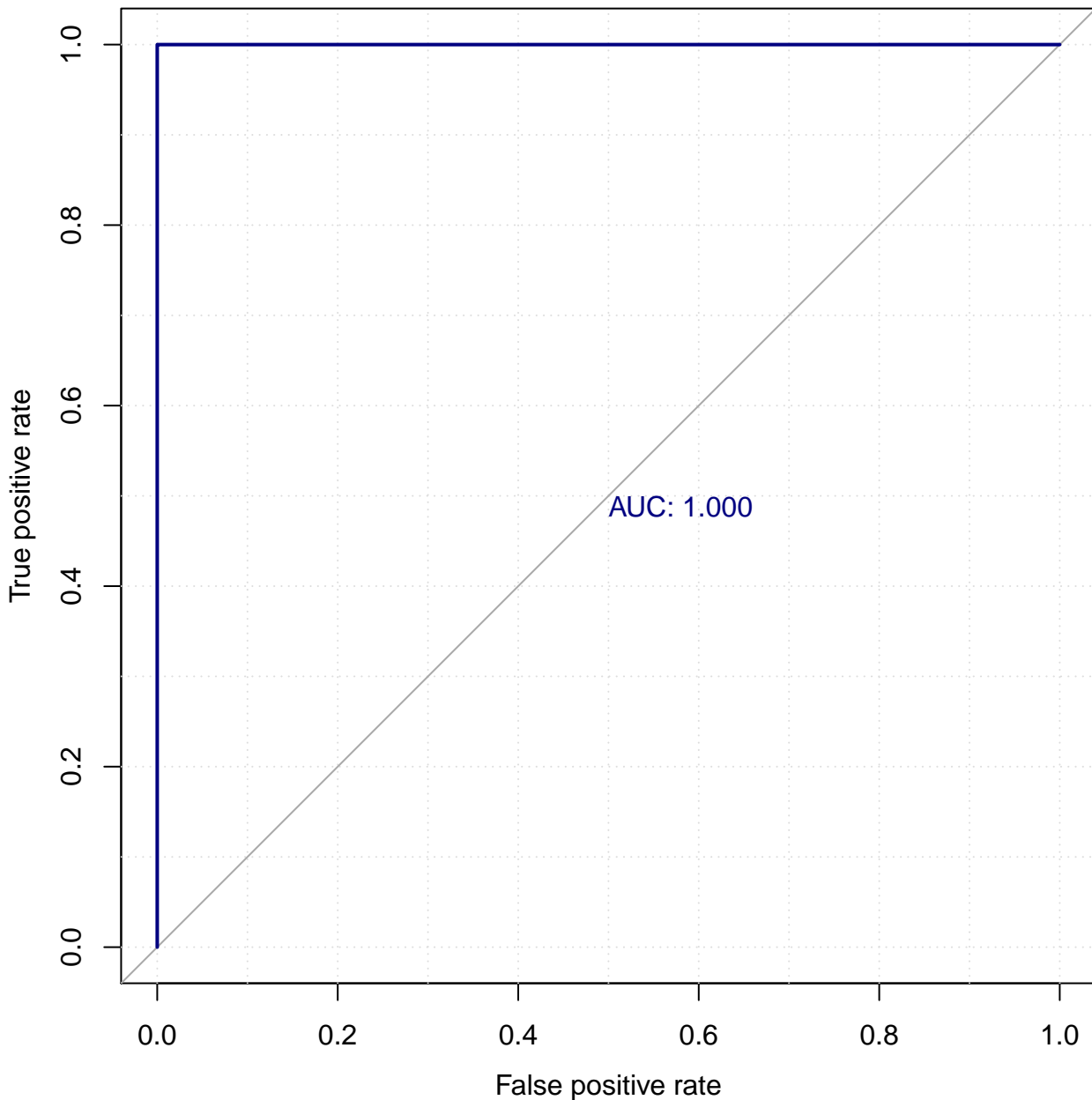

Supplement: Supplemental Information 2 [file peerj-11-15112-s002.zip › peerj-75361-Raw_data_result/Raw data/Result-X101SC21103966-Z01-J001-B1-42/4.MetDiffAnalysis/H.vs.NH/ROC_pos/Com_2406_pos_ROC.pdf]

# H.vs.NH

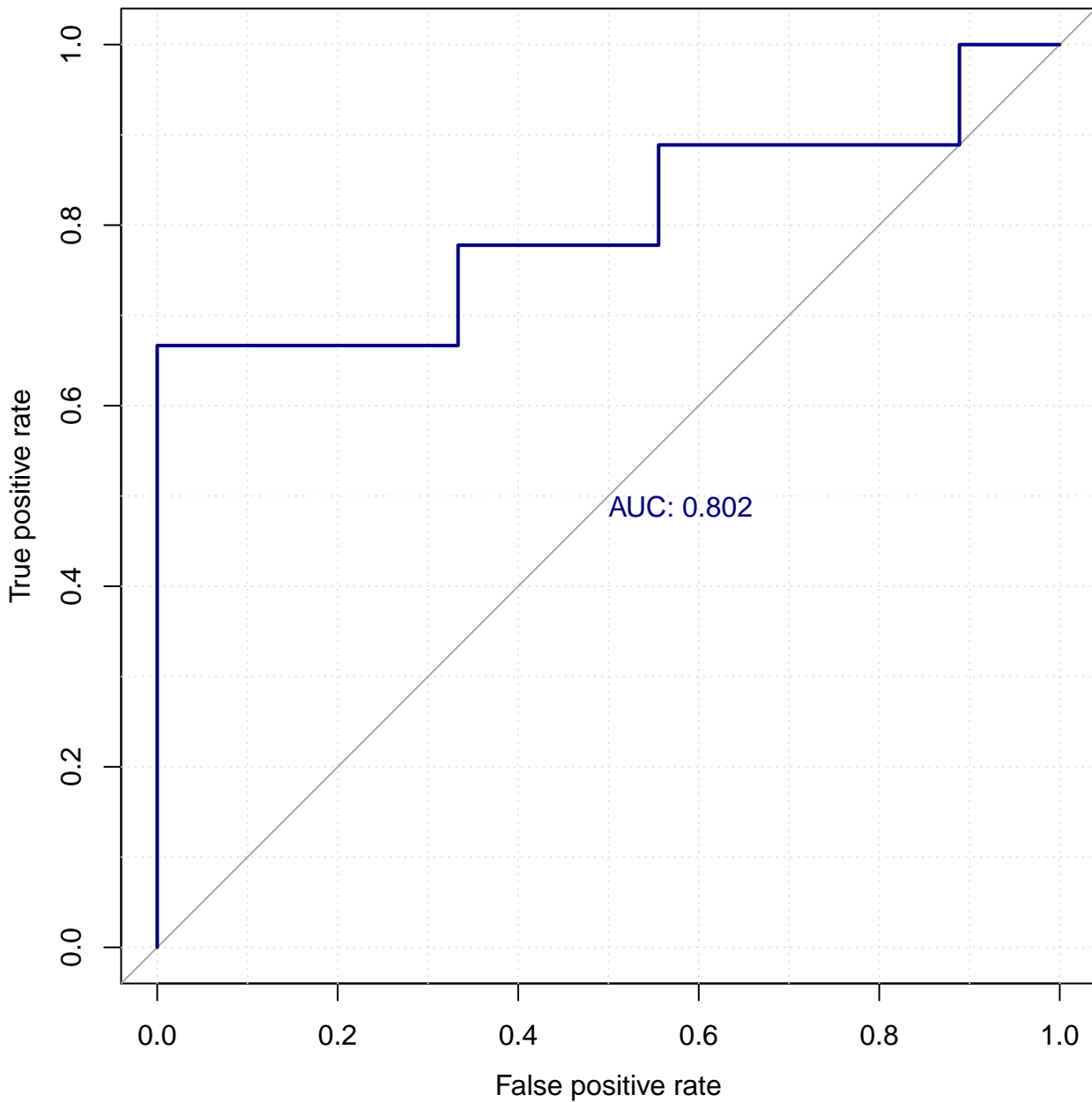

Supplement: Supplemental Information 2 [file peerj-11-15112-s002.zip › peerj-75361-Raw_data_result/Raw data/Result-X101SC21103966-Z01-J001-B1-42/4.MetDiffAnalysis/H.vs.NH/ROC_pos/Com_2544_pos_ROC.pdf]

# H.vs.NH

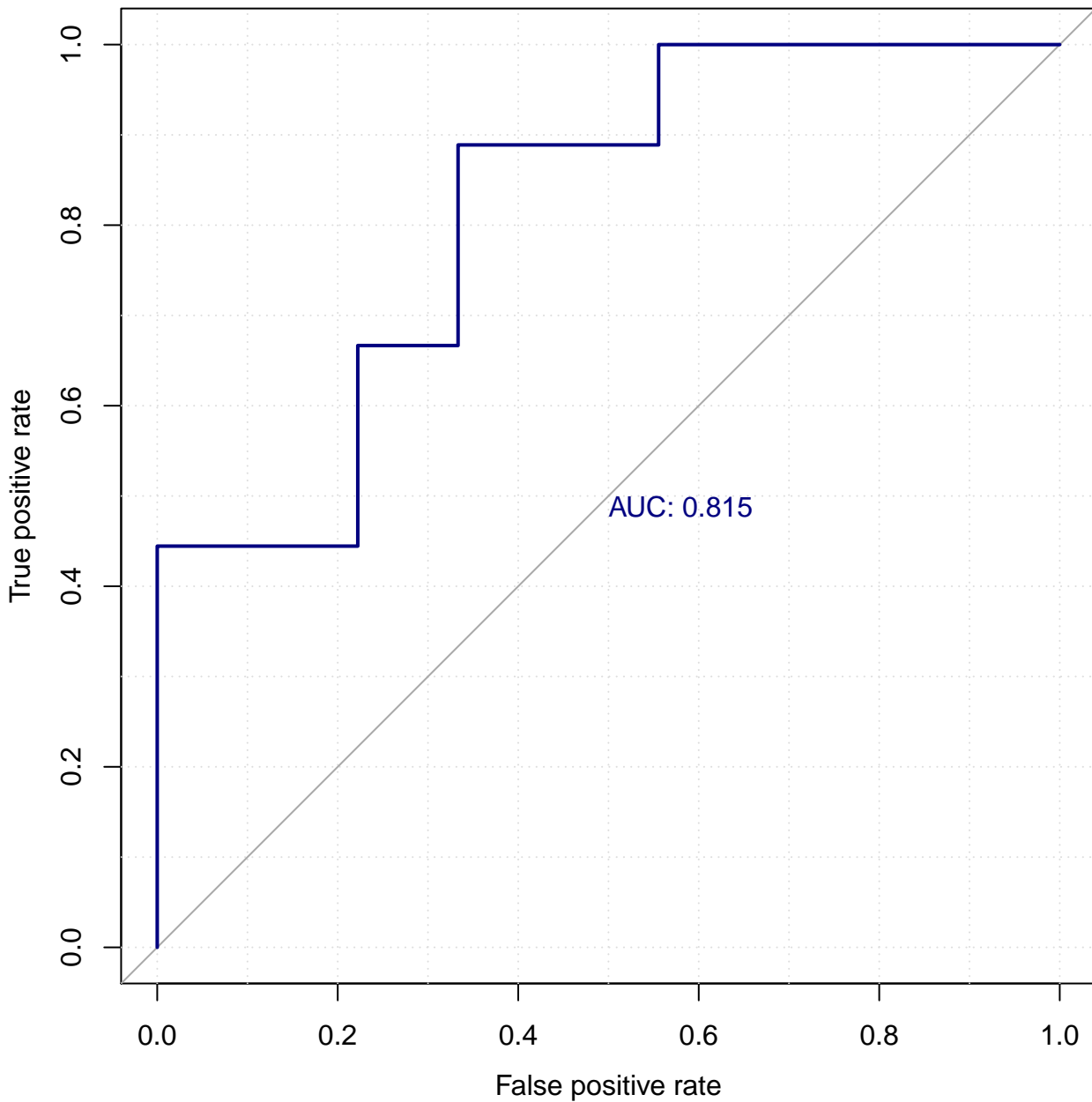

Supplement: Supplemental Information 2 [file peerj-11-15112-s002.zip › peerj-75361-Raw_data_result/Raw data/Result-X101SC21103966-Z01-J001-B1-42/4.MetDiffAnalysis/H.vs.NH/ROC_pos/Com_261_pos_ROC.pdf]

H.vs.NH

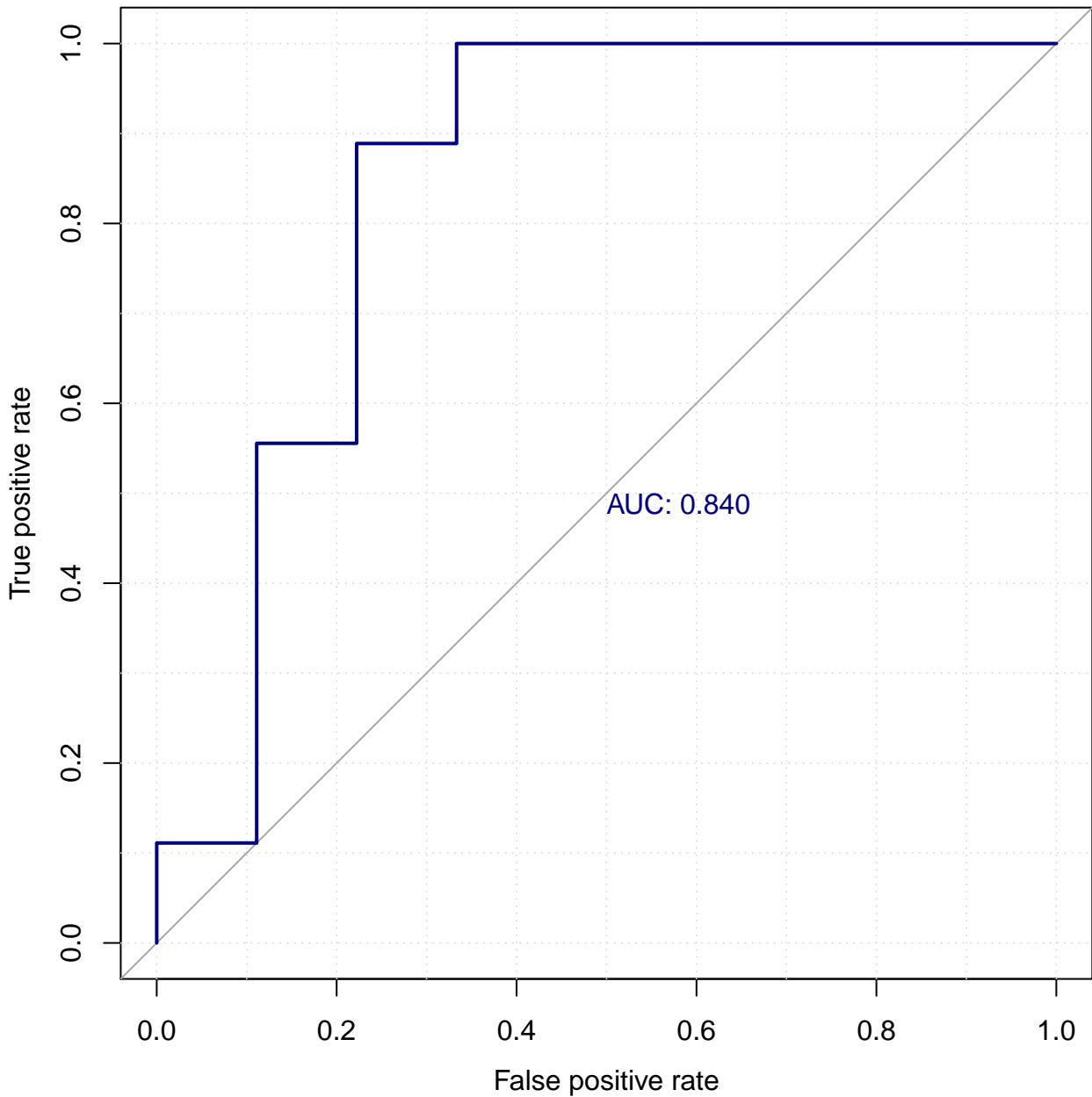

Supplement: Supplemental Information 2 [file peerj-11-15112-s002.zip › peerj-75361-Raw_data_result/Raw data/Result-X101SC21103966-Z01-J001-B1-42/4.MetDiffAnalysis/H.vs.NH/ROC_pos/Com_280_pos_ROC.pdf]

# H.vs.NH

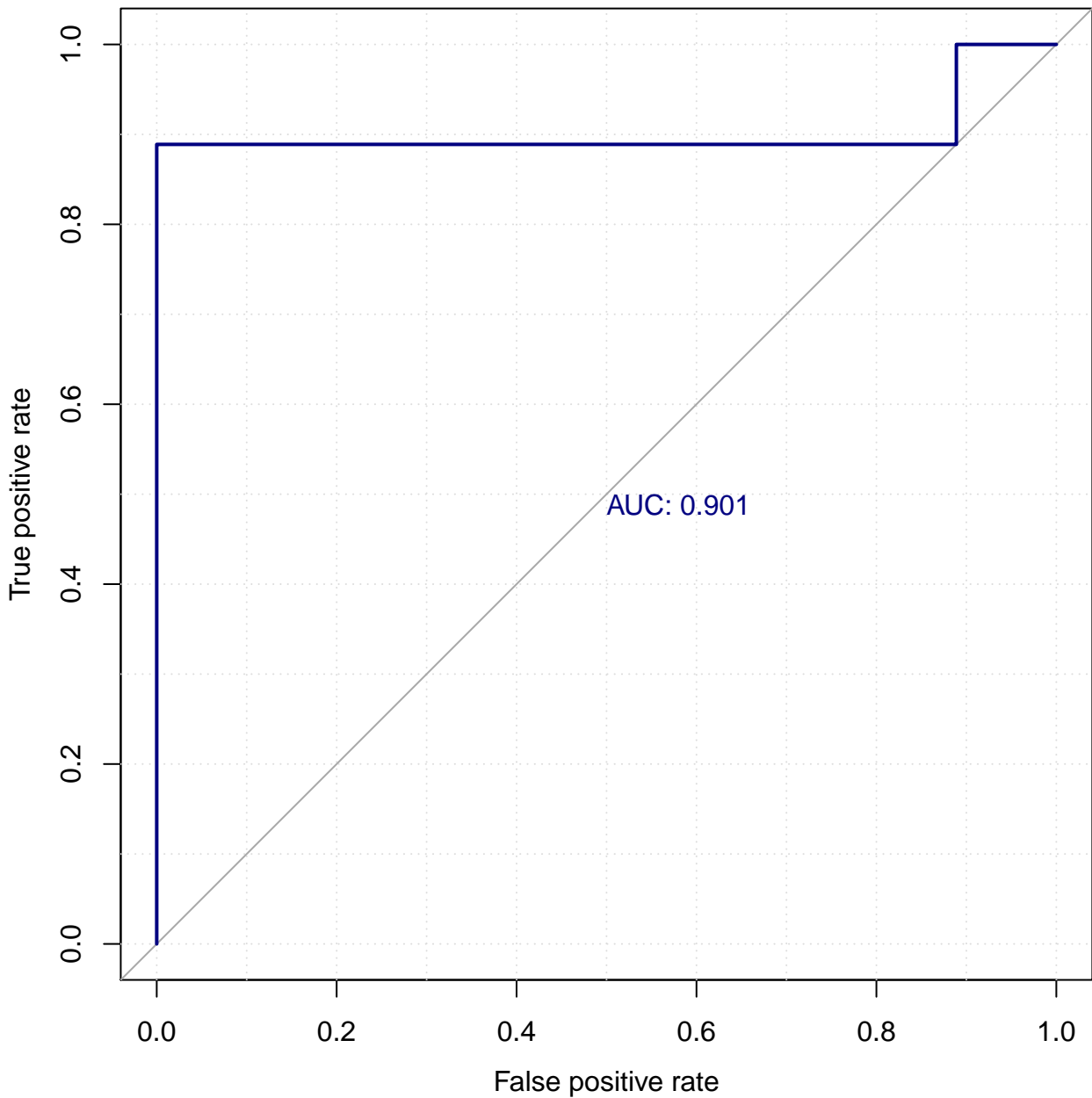

Supplement: Supplemental Information 2 [file peerj-11-15112-s002.zip › peerj-75361-Raw_data_result/Raw data/Result-X101SC21103966-Z01-J001-B1-42/4.MetDiffAnalysis/H.vs.NH/ROC_pos/Com_2820_pos_ROC.pdf]

H.vs.NH

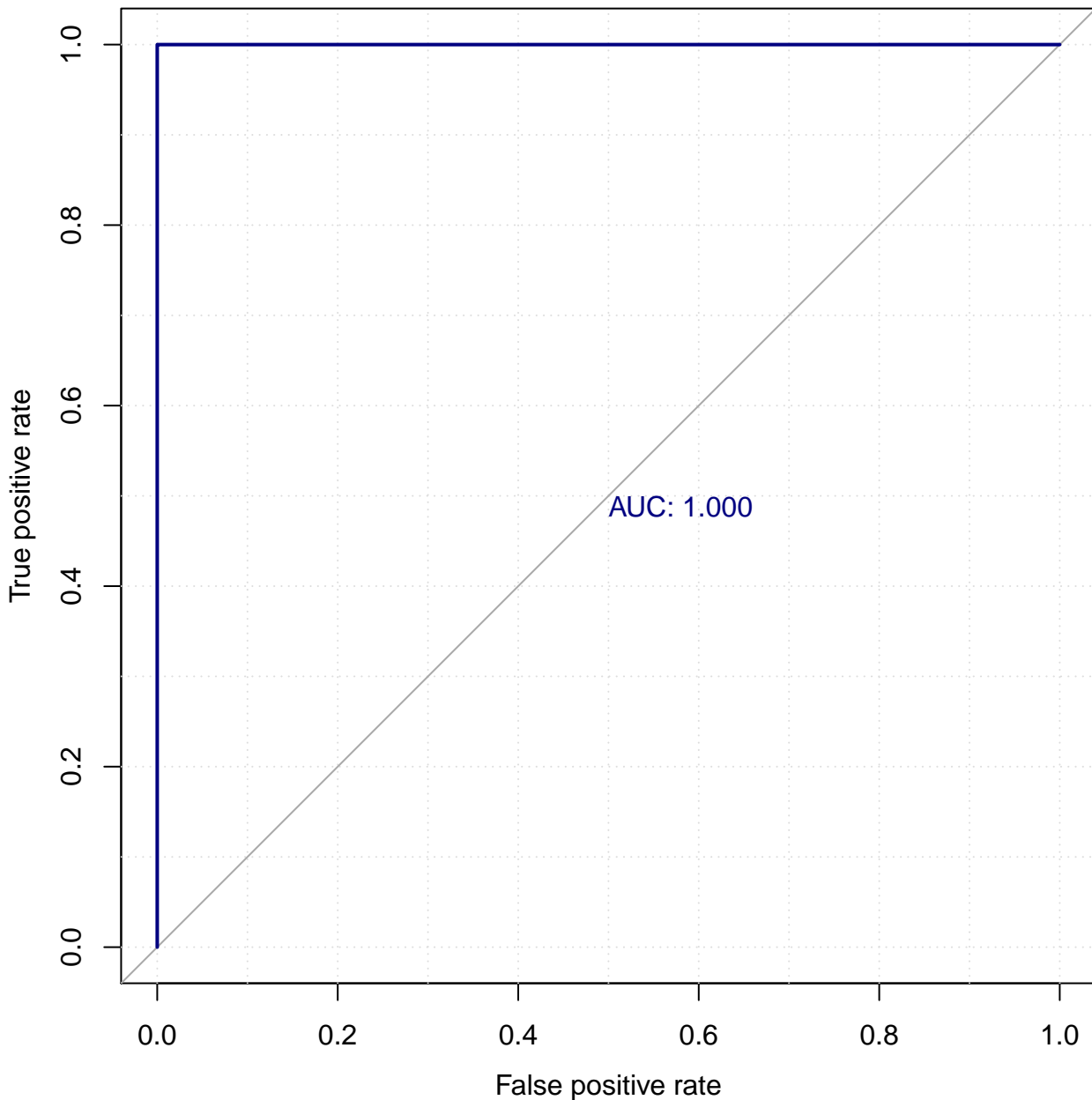

Supplement: Supplemental Information 2 [file peerj-11-15112-s002.zip › peerj-75361-Raw_data_result/Raw data/Result-X101SC21103966-Z01-J001-B1-42/4.MetDiffAnalysis/H.vs.NH/ROC_pos/Com_2912_pos_ROC.pdf]

# H.vs.NH

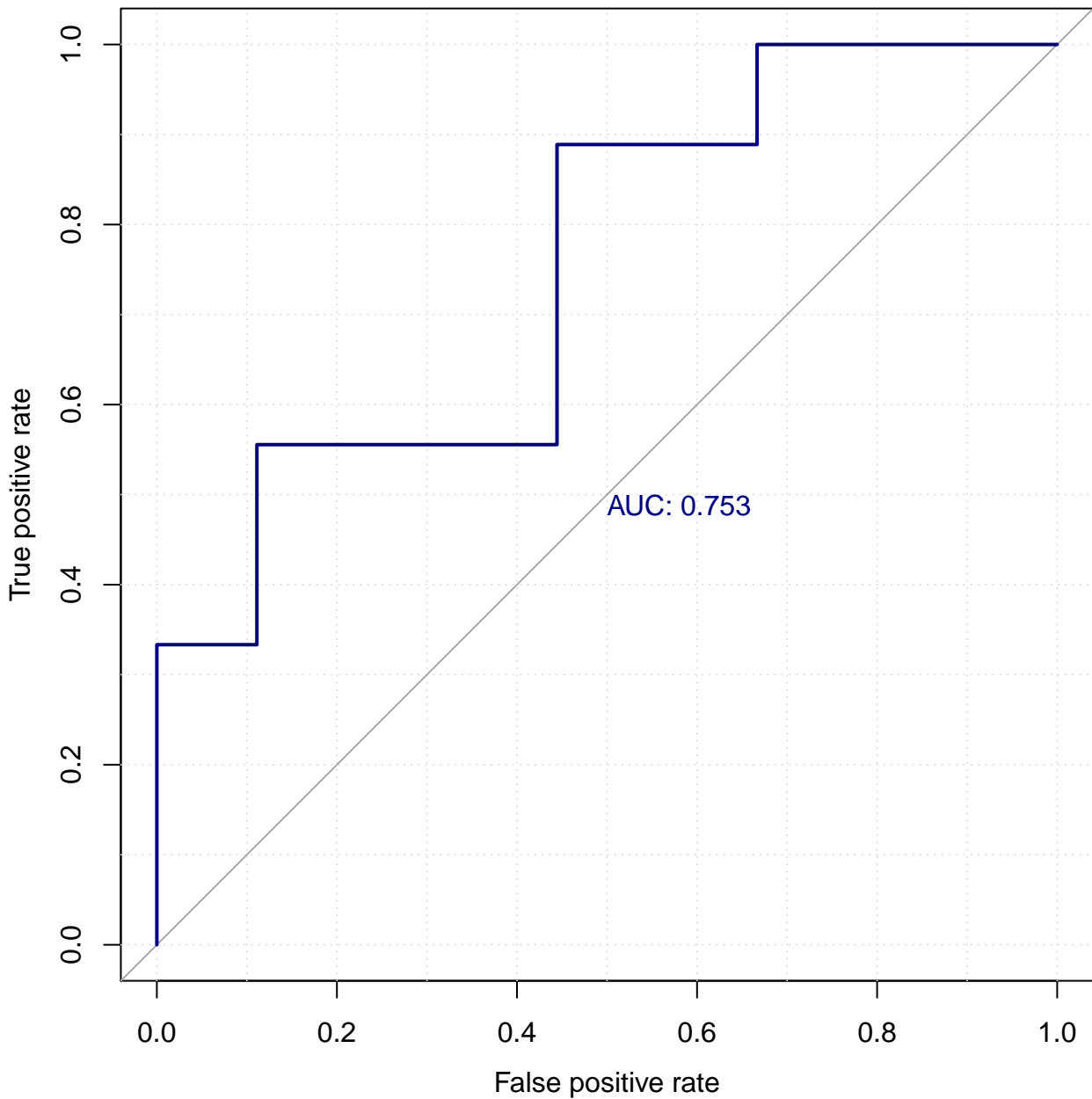

Supplement: Supplemental Information 2 [file peerj-11-15112-s002.zip › peerj-75361-Raw_data_result/Raw data/Result-X101SC21103966-Z01-J001-B1-42/4.MetDiffAnalysis/H.vs.NH/ROC_pos/Com_2936_pos_ROC.pdf]

# H.vs.NH

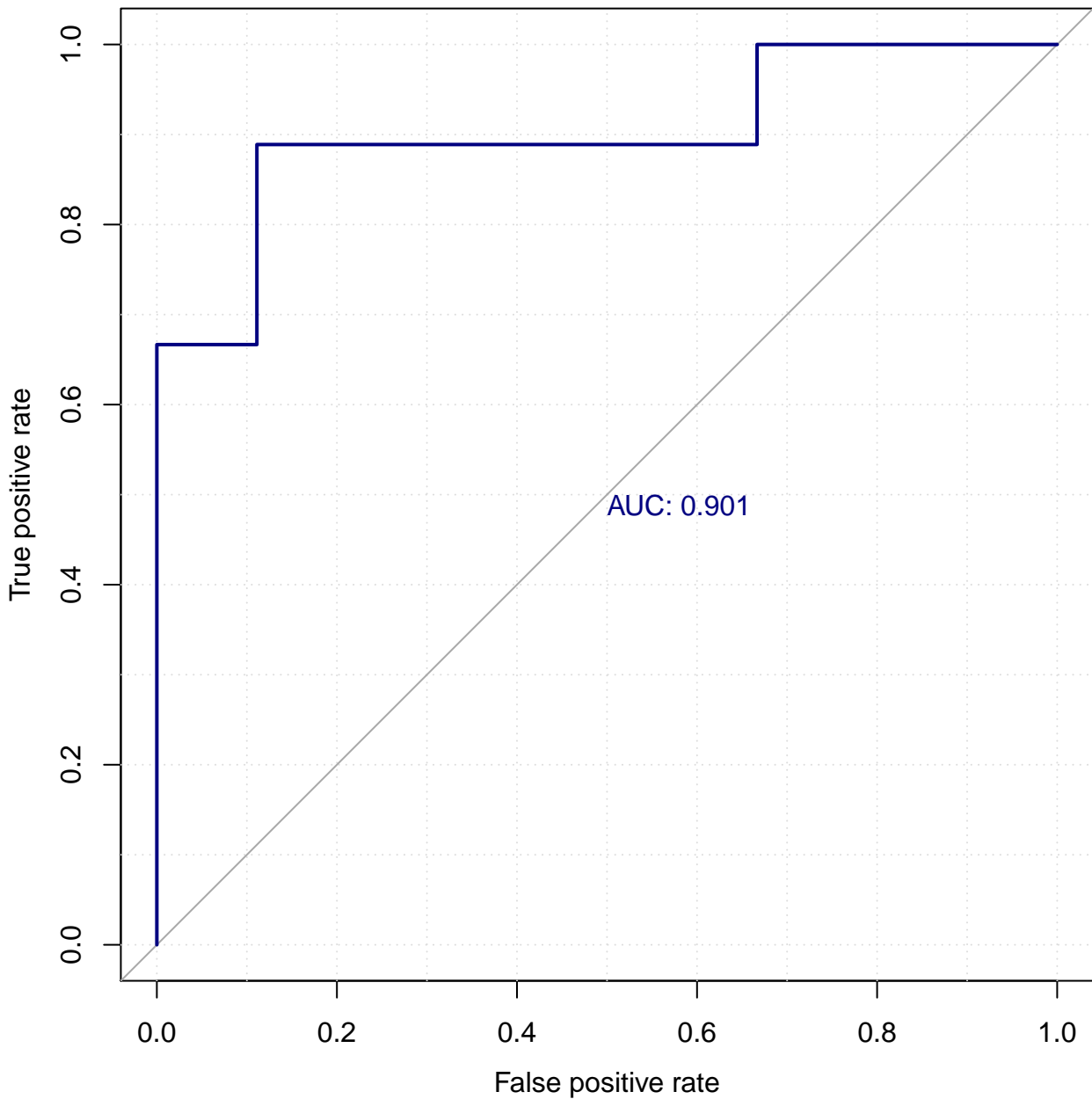

Supplement: Supplemental Information 2 [file peerj-11-15112-s002.zip › peerj-75361-Raw_data_result/Raw data/Result-X101SC21103966-Z01-J001-B1-42/4.MetDiffAnalysis/H.vs.NH/ROC_pos/Com_2982_pos_ROC.pdf]

H.vs.NH

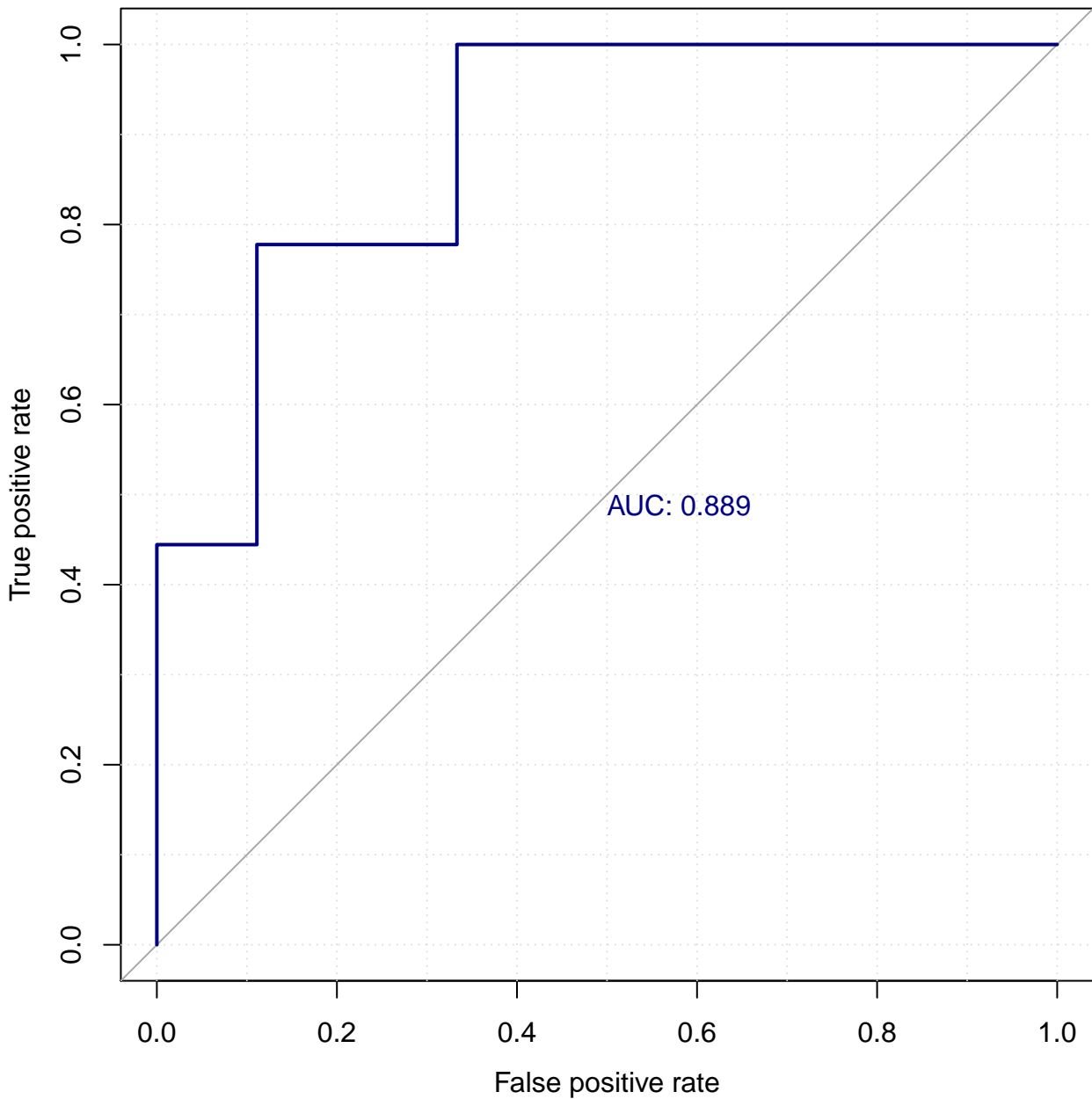

Supplement: Supplemental Information 2 [file peerj-11-15112-s002.zip › peerj-75361-Raw_data_result/Raw data/Result-X101SC21103966-Z01-J001-B1-42/4.MetDiffAnalysis/H.vs.NH/ROC_pos/Com_3220_pos_ROC.pdf]

# H.vs.NH

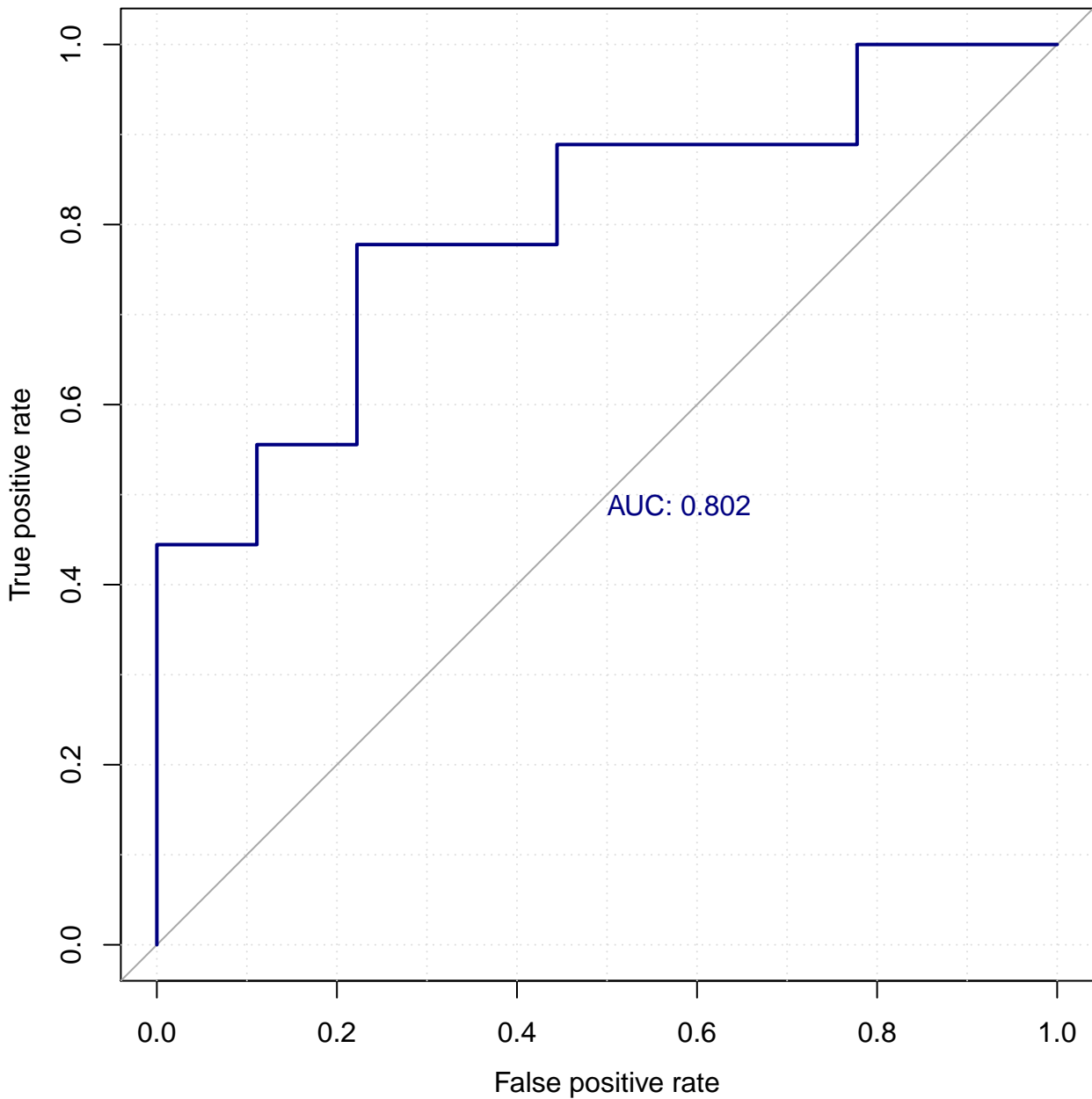

Supplement: Supplemental Information 2 [file peerj-11-15112-s002.zip › peerj-75361-Raw_data_result/Raw data/Result-X101SC21103966-Z01-J001-B1-42/4.MetDiffAnalysis/H.vs.NH/ROC_pos/Com_3343_pos_ROC.pdf]

H.vs.NH

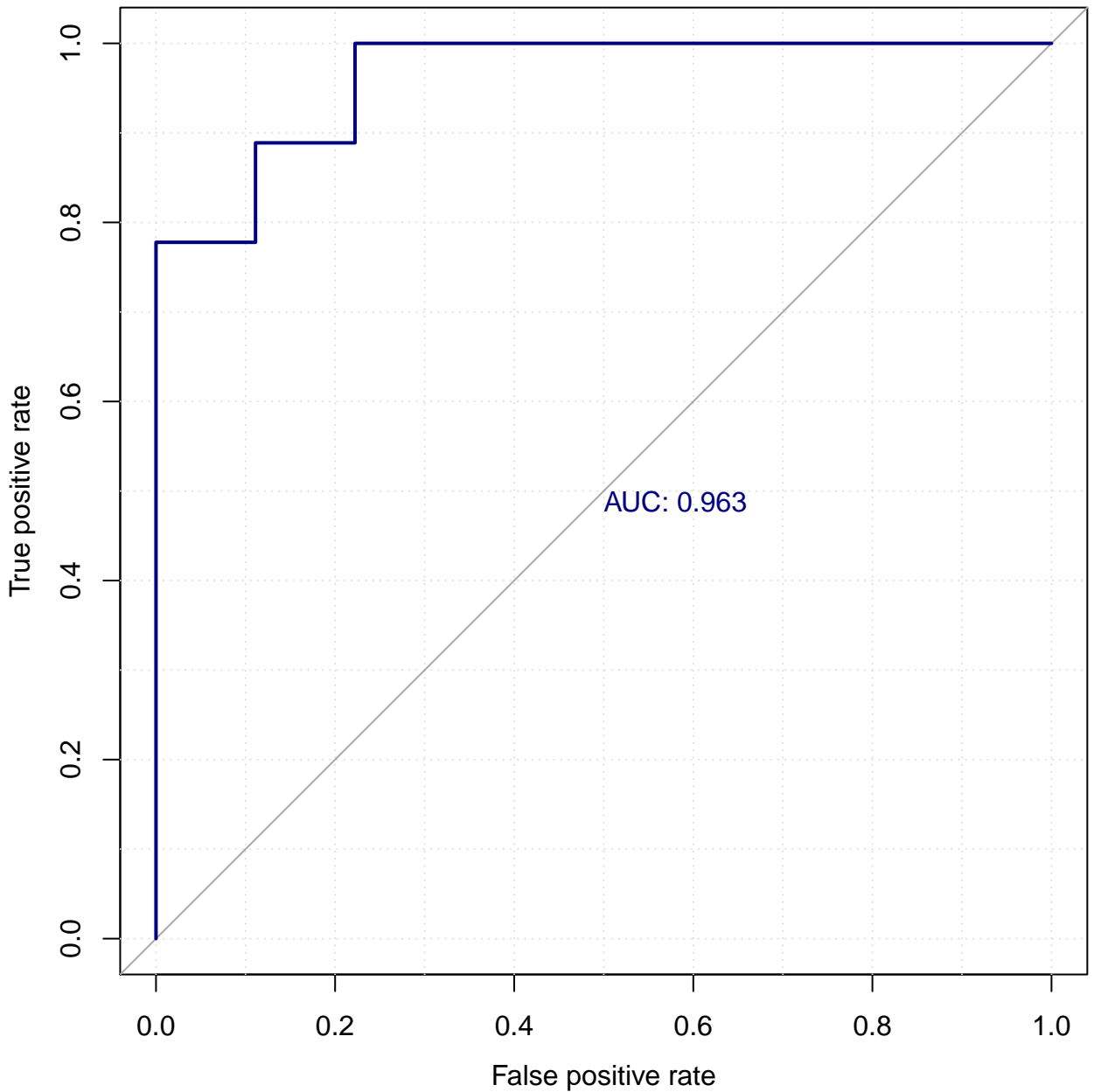

Supplement: Supplemental Information 2 [file peerj-11-15112-s002.zip › peerj-75361-Raw_data_result/Raw data/Result-X101SC21103966-Z01-J001-B1-42/4.MetDiffAnalysis/H.vs.NH/ROC_pos/Com_3399_pos_ROC.pdf]

# H.vs.NH

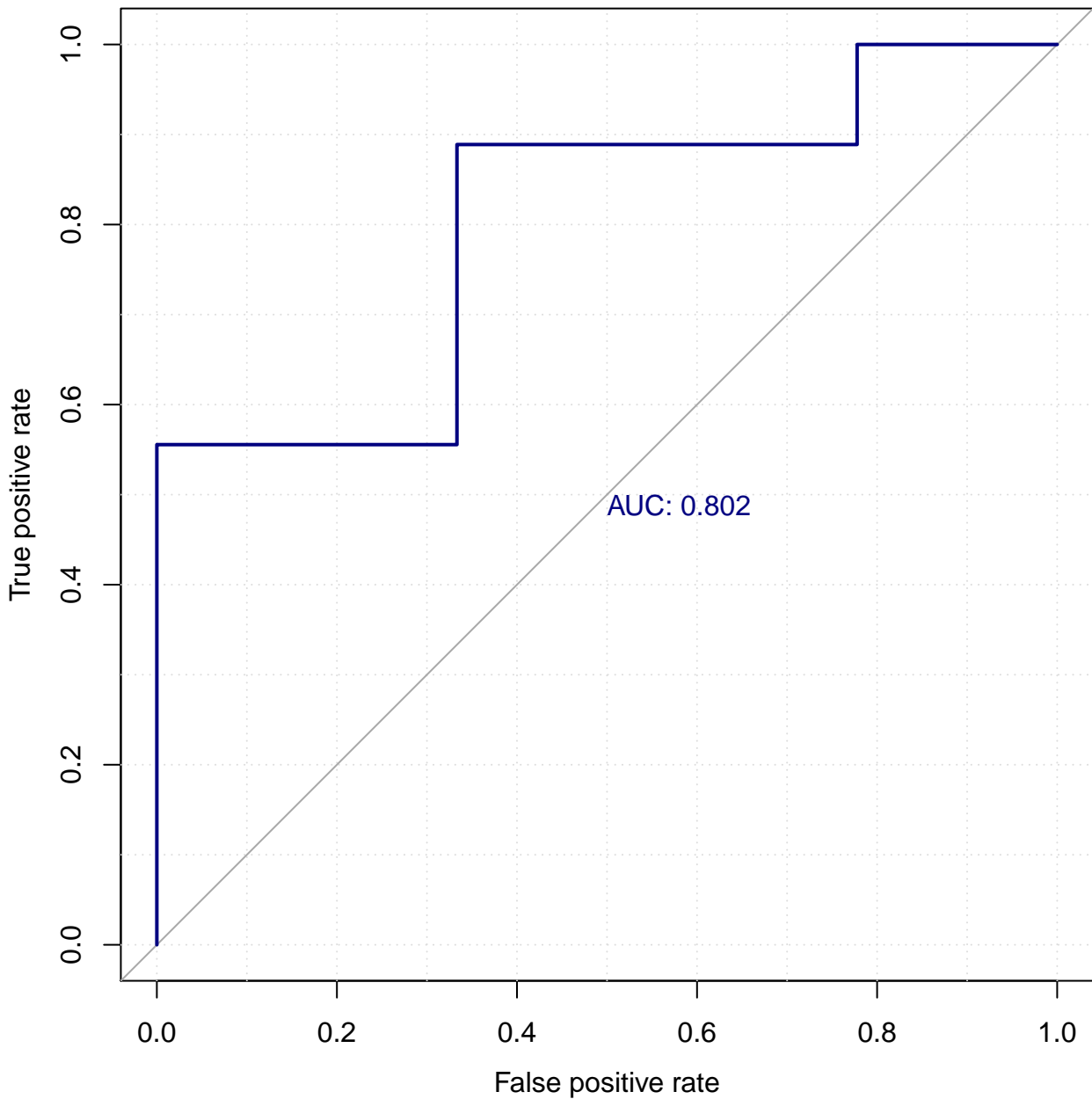

Supplement: Supplemental Information 2 [file peerj-11-15112-s002.zip › peerj-75361-Raw_data_result/Raw data/Result-X101SC21103966-Z01-J001-B1-42/4.MetDiffAnalysis/H.vs.NH/ROC_pos/Com_358_pos_ROC.pdf]

# H.vs.NH

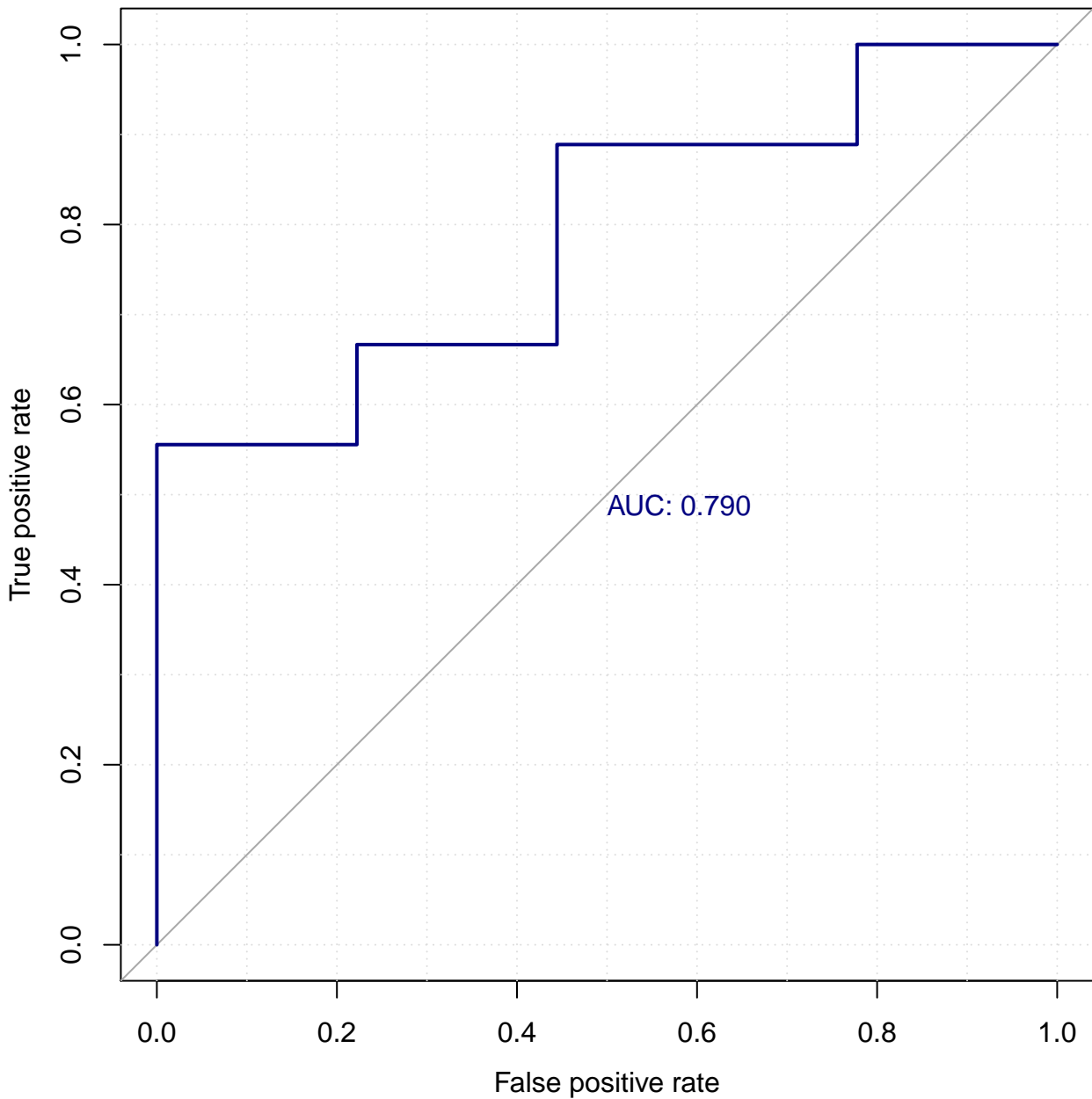

Supplement: Supplemental Information 2 [file peerj-11-15112-s002.zip › peerj-75361-Raw_data_result/Raw data/Result-X101SC21103966-Z01-J001-B1-42/4.MetDiffAnalysis/H.vs.NH/ROC_pos/Com_376_pos_ROC.pdf]

# H.vs.NH

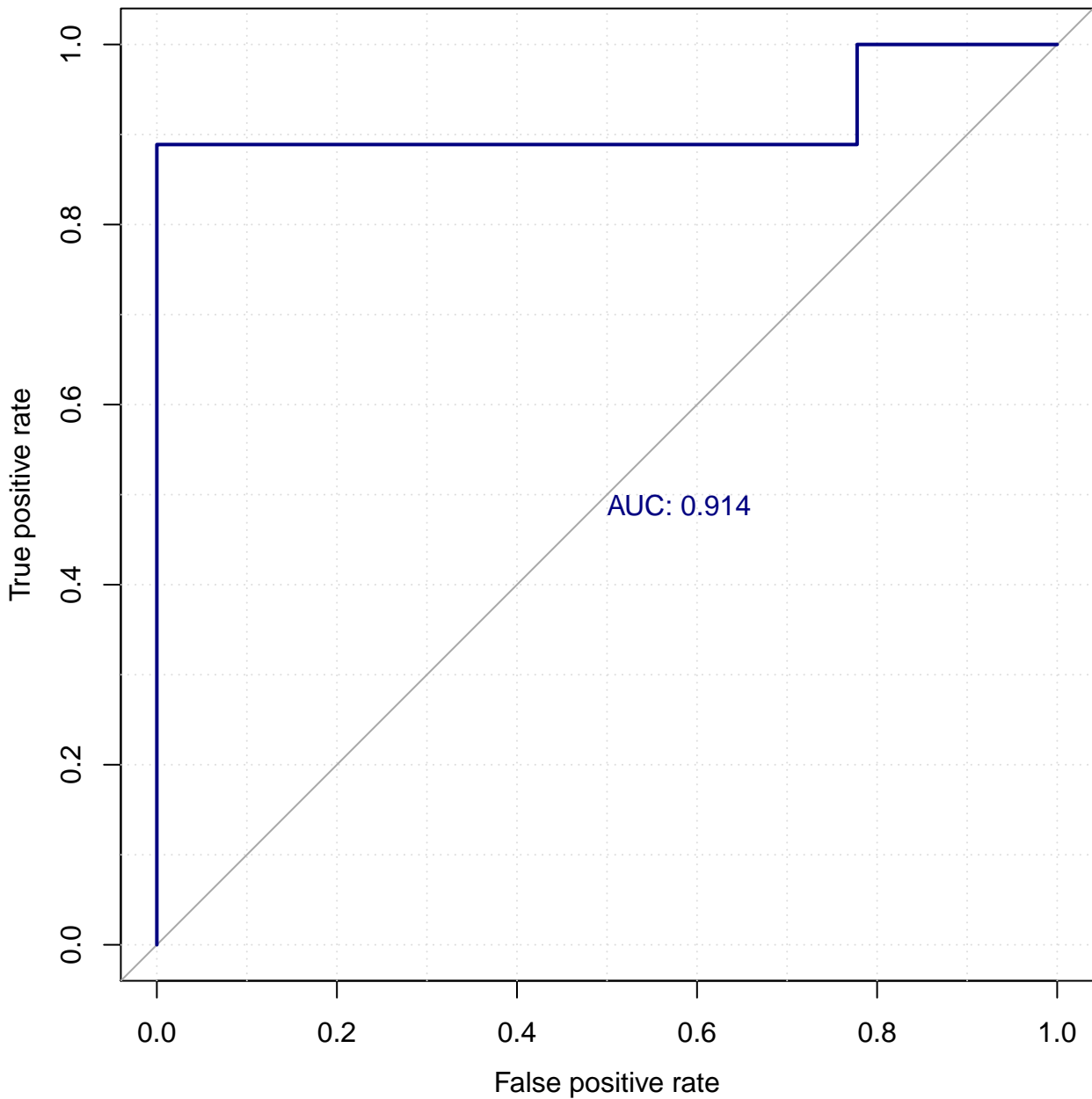

Supplement: Supplemental Information 2 [file peerj-11-15112-s002.zip › peerj-75361-Raw_data_result/Raw data/Result-X101SC21103966-Z01-J001-B1-42/4.MetDiffAnalysis/H.vs.NH/ROC_pos/Com_3932_pos_ROC.pdf]
