# Supplementary figures and images for: Semen quality and seminal plasma metabolites in male rabbits (Oryctolagus cuniculus) under heat stress (part 1 of 2)
Source: PeerJ. 2023 Apr 7;11:e15112. doi: 10.7717/peerj.15112 (PMC10103697; doi:10.7717/peerj.15112)

Pearson correlation between neg QC samples

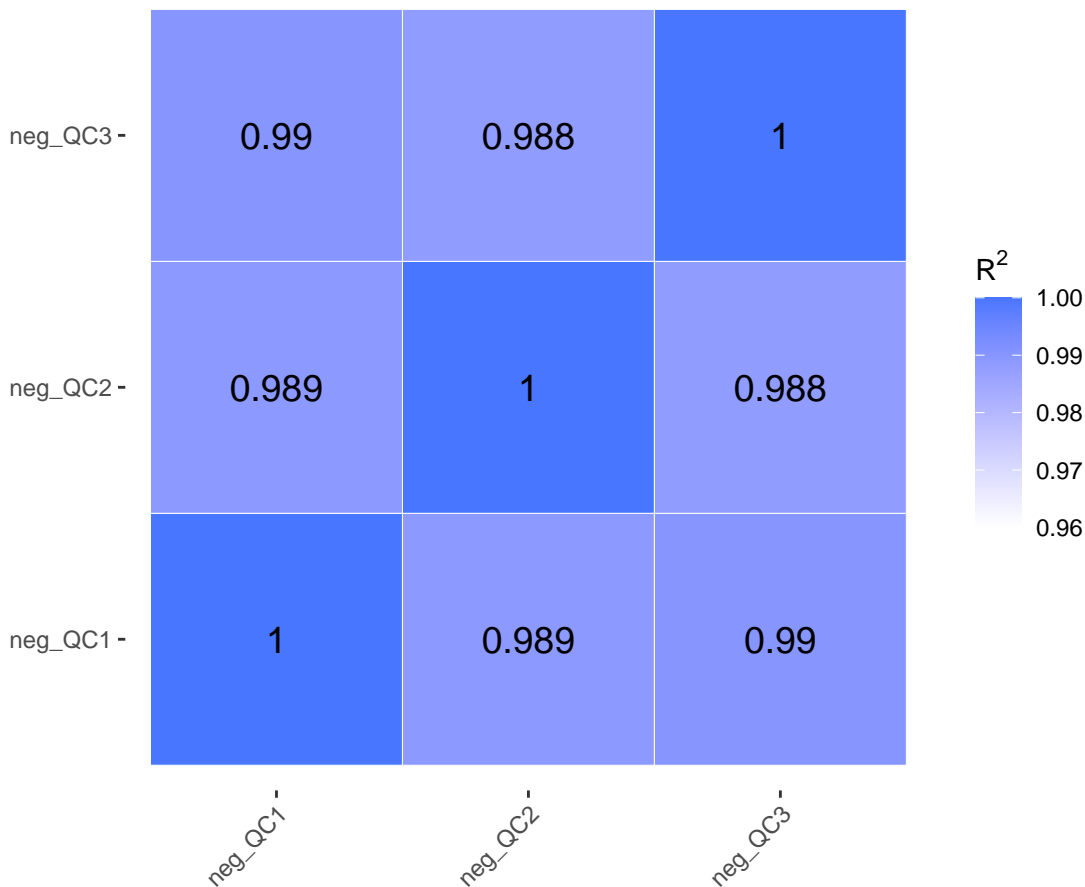

Supplement: Supplemental Information 2 [file peerj-11-15112-s002.zip › peerj-75361-Raw_data_result/Raw data/Result-X101SC21103966-Z01-J001-B1-42/1.MetQuant-QC/Correlation/cor_pearson_neg.pdf]

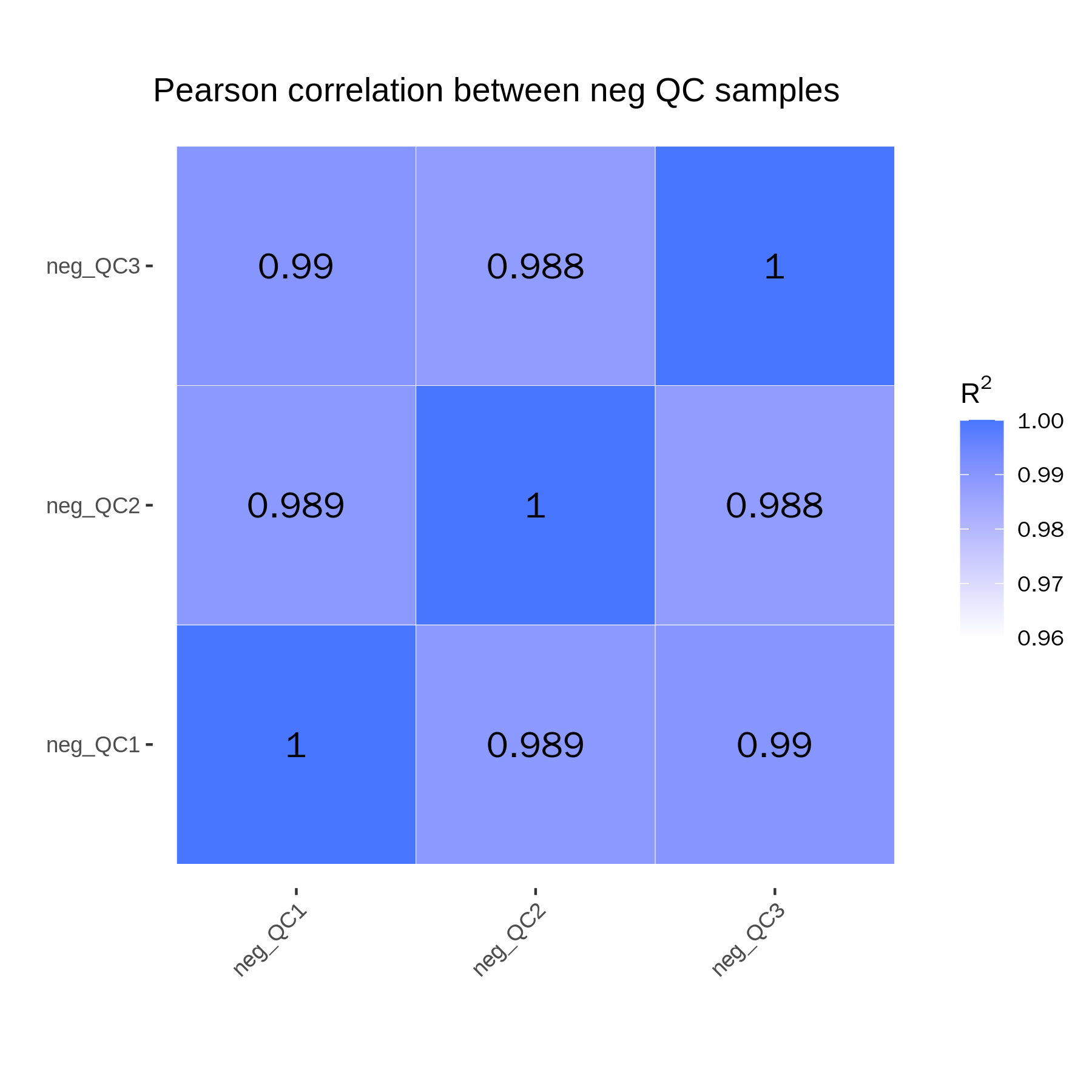

Supplement: Supplemental Information 2 [file peerj-11-15112-s002.zip › peerj-75361-Raw_data_result/Raw data/Result-X101SC21103966-Z01-J001-B1-42/1.MetQuant-QC/Correlation/cor_pearson_neg.png]

Pearson correlation between pos QC samples

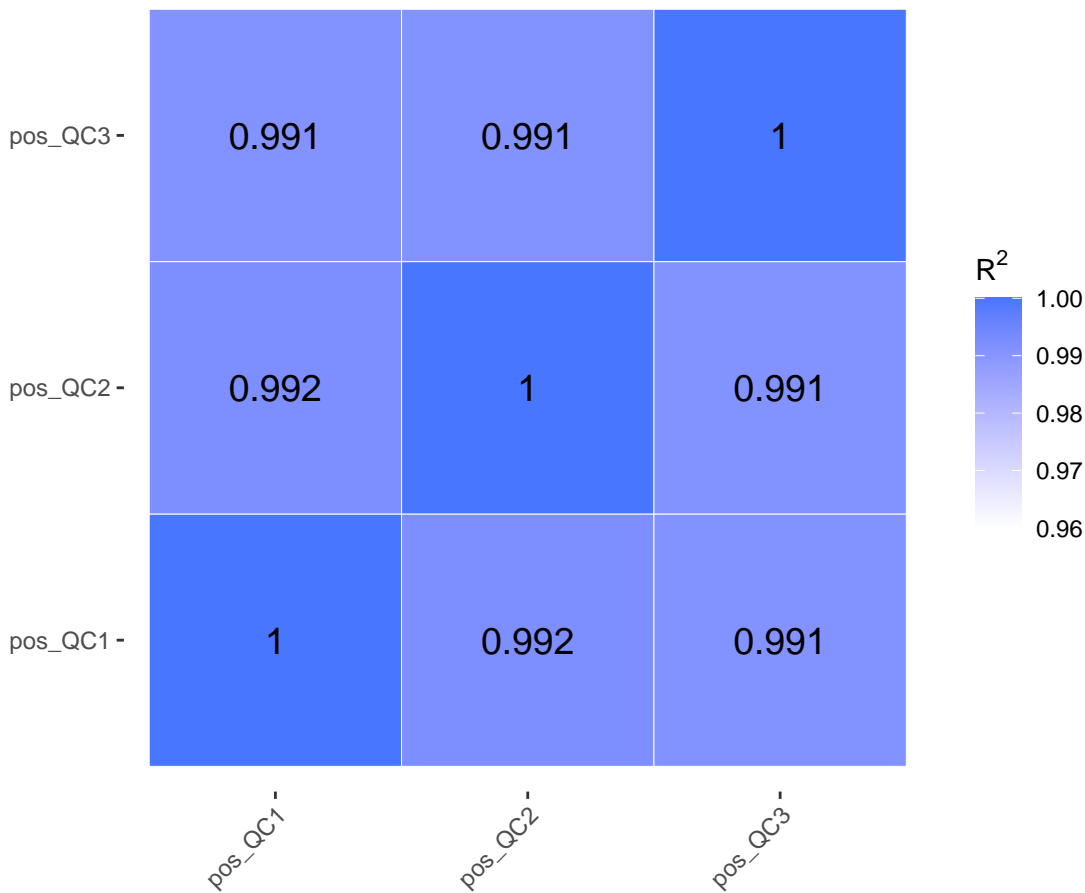

Supplement: Supplemental Information 2 [file peerj-11-15112-s002.zip › peerj-75361-Raw_data_result/Raw data/Result-X101SC21103966-Z01-J001-B1-42/1.MetQuant-QC/Correlation/cor_pearson_pos.pdf]

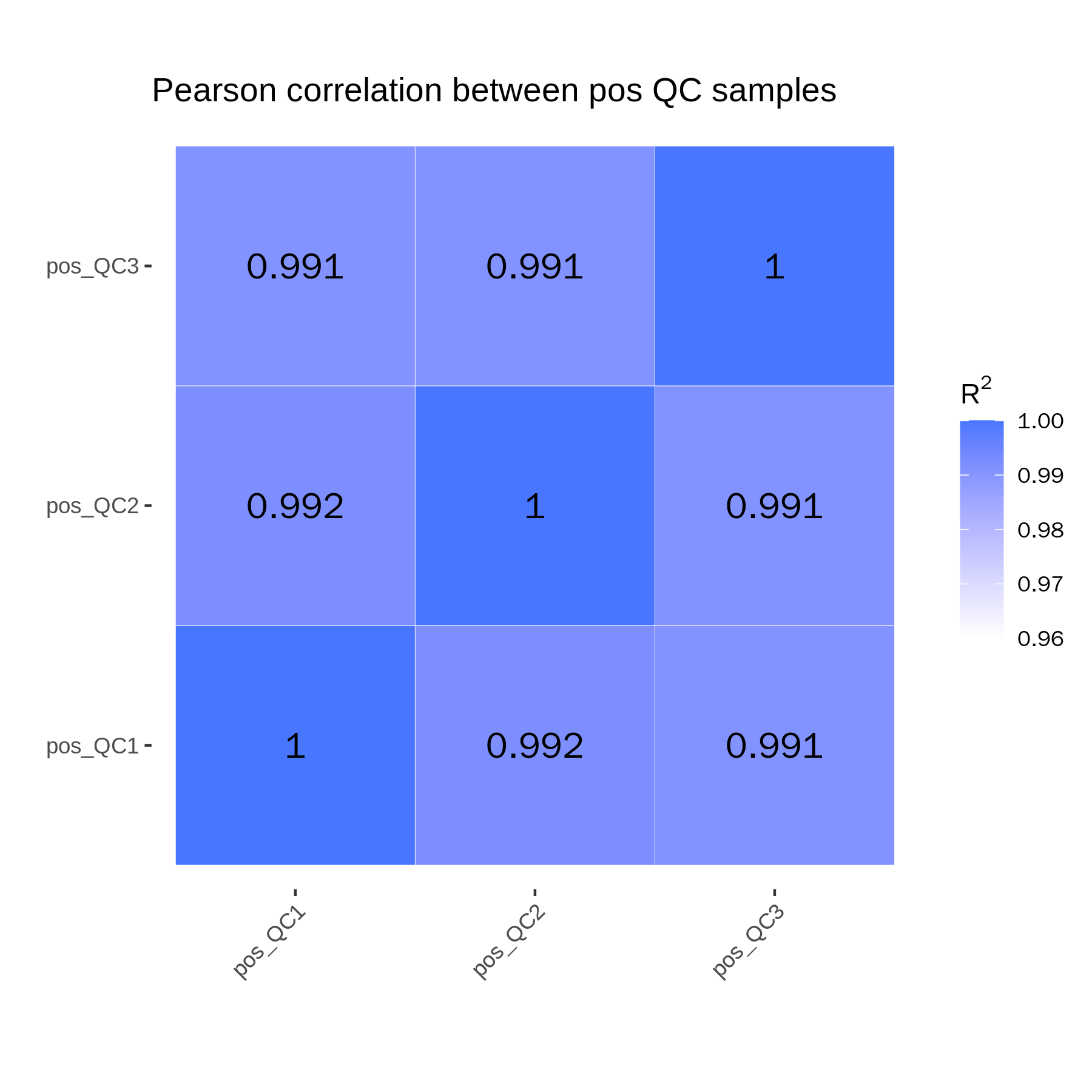

Supplement: Supplemental Information 2 [file peerj-11-15112-s002.zip › peerj-75361-Raw_data_result/Raw data/Result-X101SC21103966-Z01-J001-B1-42/1.MetQuant-QC/Correlation/cor_pearson_pos.png]

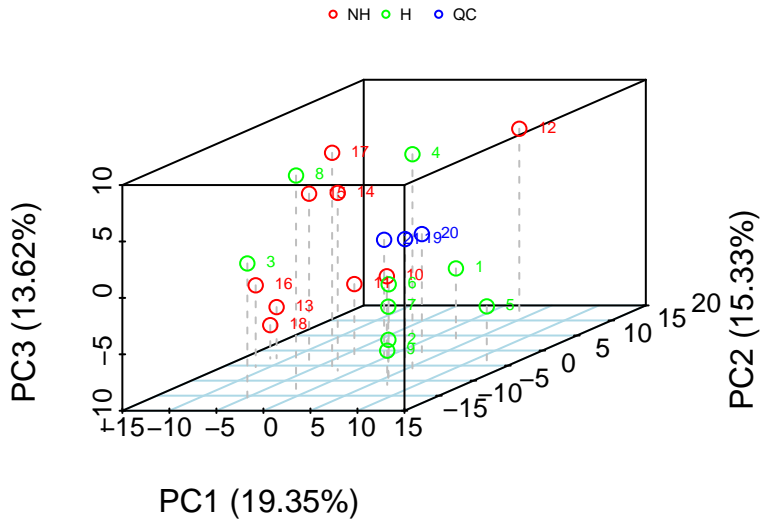

Supplement: Supplemental Information 2 [file peerj-11-15112-s002.zip › peerj-75361-Raw_data_result/Raw data/Result-X101SC21103966-Z01-J001-B1-42/1.MetQuant-QC/Samples_QC_neg-PCA.3D.pdf]

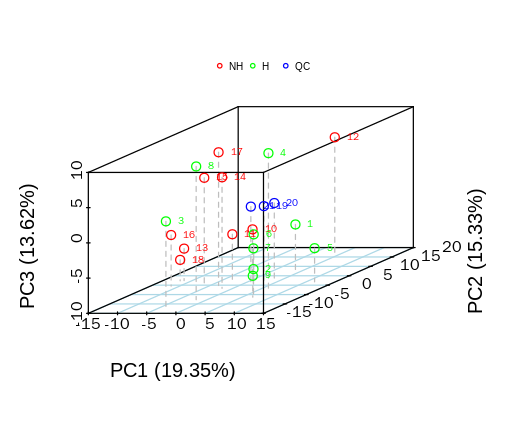

Supplement: Supplemental Information 2 [file peerj-11-15112-s002.zip › peerj-75361-Raw_data_result/Raw data/Result-X101SC21103966-Z01-J001-B1-42/1.MetQuant-QC/Samples_QC_neg-PCA.3D.png]

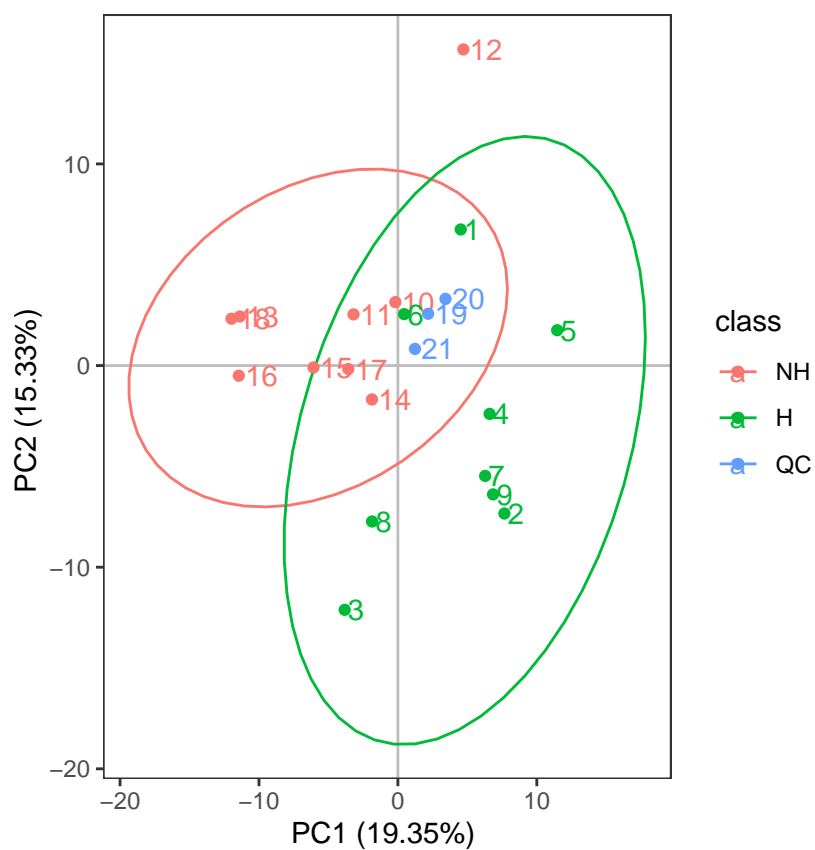

Supplement: Supplemental Information 2 [file peerj-11-15112-s002.zip › peerj-75361-Raw_data_result/Raw data/Result-X101SC21103966-Z01-J001-B1-42/1.MetQuant-QC/Samples_QC_neg-PCA.pdf]

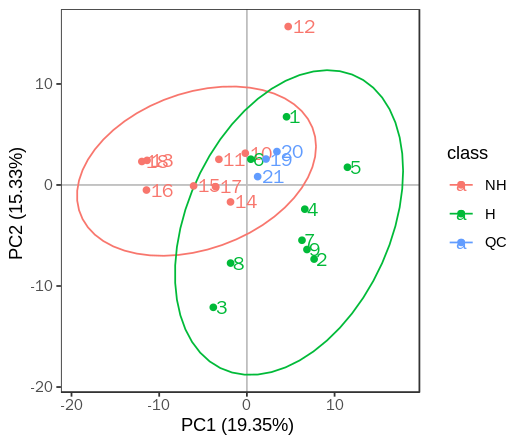

Supplement: Supplemental Information 2 [file peerj-11-15112-s002.zip › peerj-75361-Raw_data_result/Raw data/Result-X101SC21103966-Z01-J001-B1-42/1.MetQuant-QC/Samples_QC_neg-PCA.png]

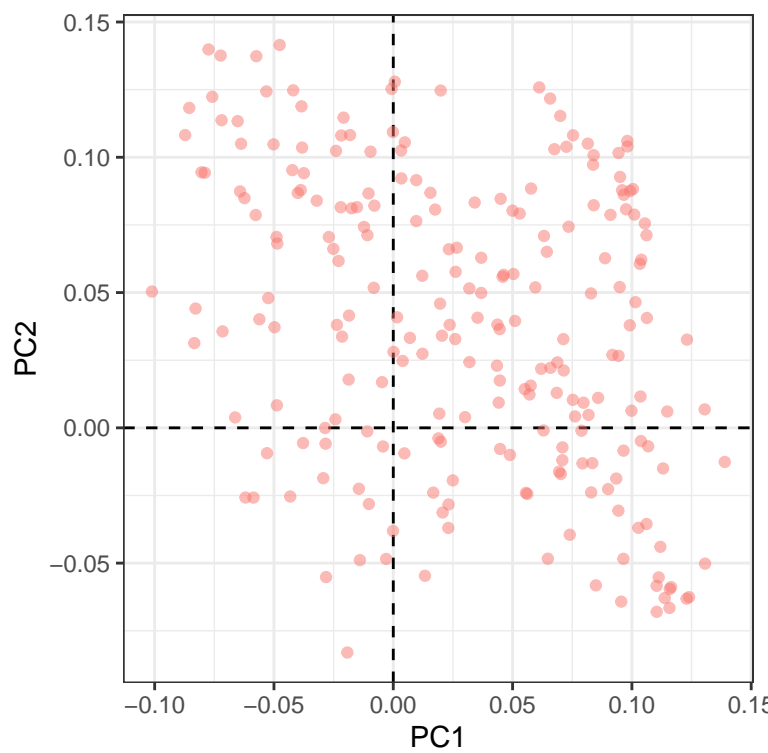

Supplement: Supplemental Information 2 [file peerj-11-15112-s002.zip › peerj-75361-Raw_data_result/Raw data/Result-X101SC21103966-Z01-J001-B1-42/1.MetQuant-QC/Samples_QC_neg-pcaloading.pdf]

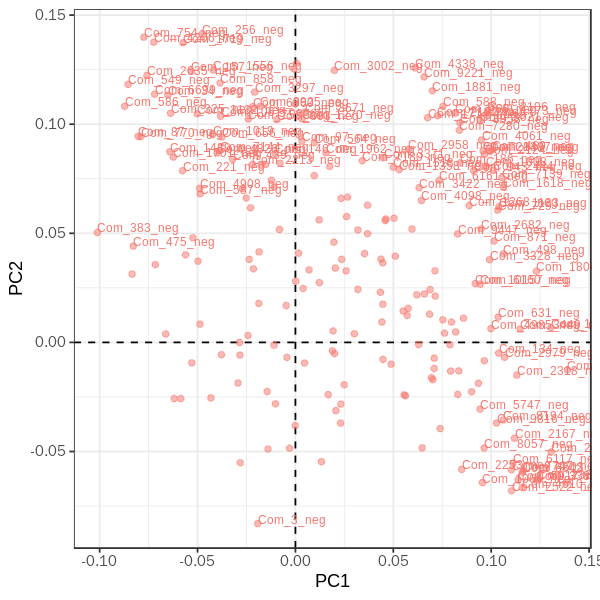

Supplement: Supplemental Information 2 [file peerj-11-15112-s002.zip › peerj-75361-Raw_data_result/Raw data/Result-X101SC21103966-Z01-J001-B1-42/1.MetQuant-QC/Samples_QC_neg-pcaloading.png]

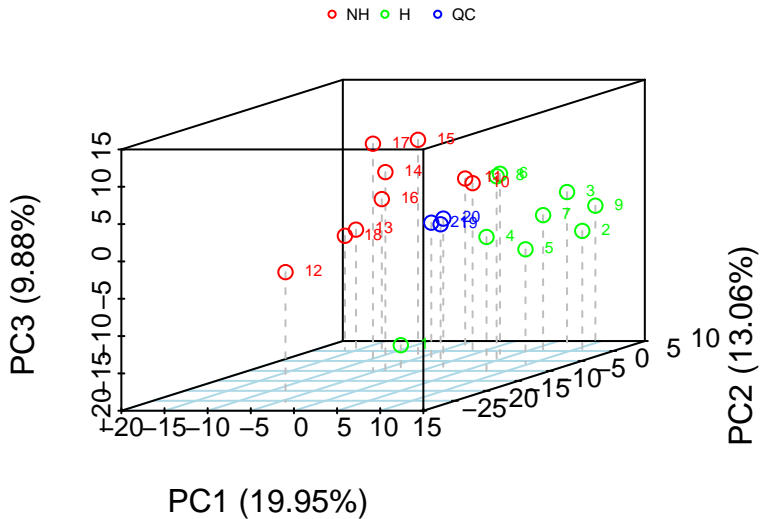

Supplement: Supplemental Information 2 [file peerj-11-15112-s002.zip › peerj-75361-Raw_data_result/Raw data/Result-X101SC21103966-Z01-J001-B1-42/1.MetQuant-QC/Samples_QC_pos-PCA.3D.pdf]

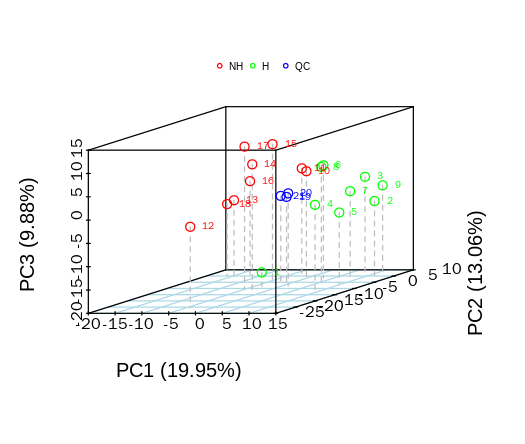

Supplement: Supplemental Information 2 [file peerj-11-15112-s002.zip › peerj-75361-Raw_data_result/Raw data/Result-X101SC21103966-Z01-J001-B1-42/1.MetQuant-QC/Samples_QC_pos-PCA.3D.png]

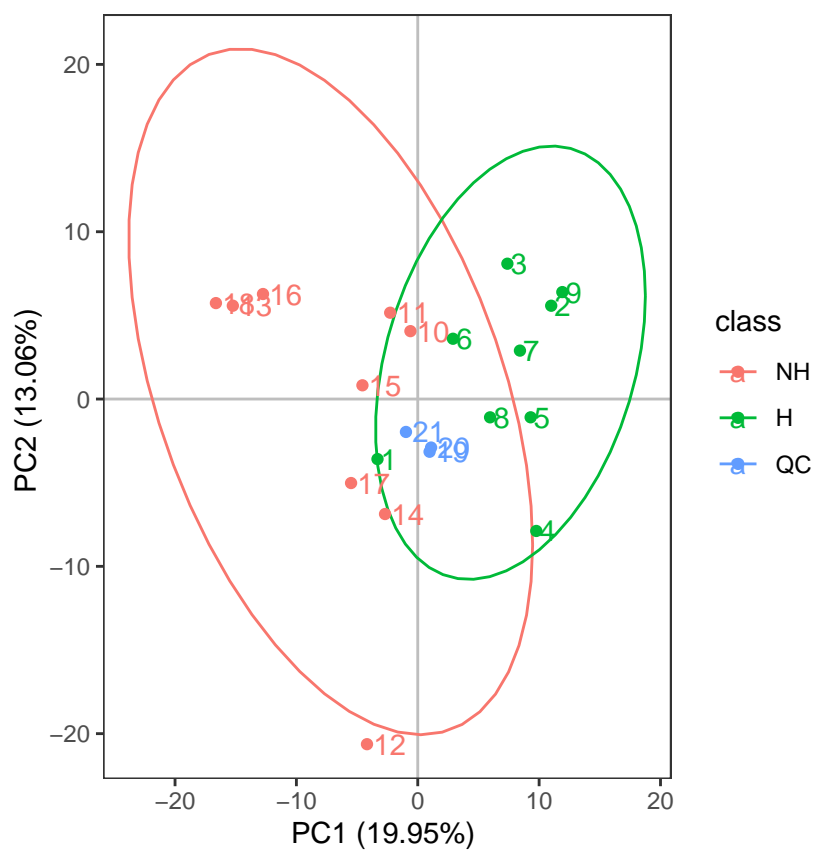

Supplement: Supplemental Information 2 [file peerj-11-15112-s002.zip › peerj-75361-Raw_data_result/Raw data/Result-X101SC21103966-Z01-J001-B1-42/1.MetQuant-QC/Samples_QC_pos-PCA.pdf]

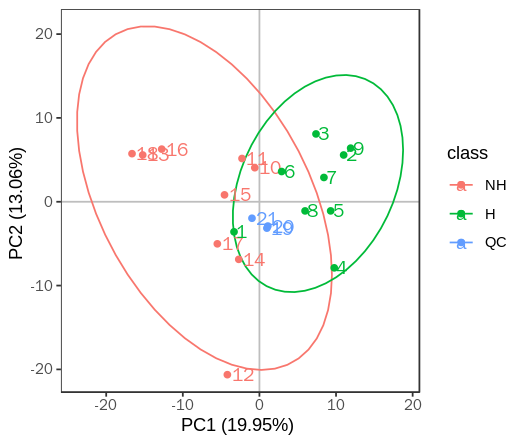

Supplement: Supplemental Information 2 [file peerj-11-15112-s002.zip › peerj-75361-Raw_data_result/Raw data/Result-X101SC21103966-Z01-J001-B1-42/1.MetQuant-QC/Samples_QC_pos-PCA.png]

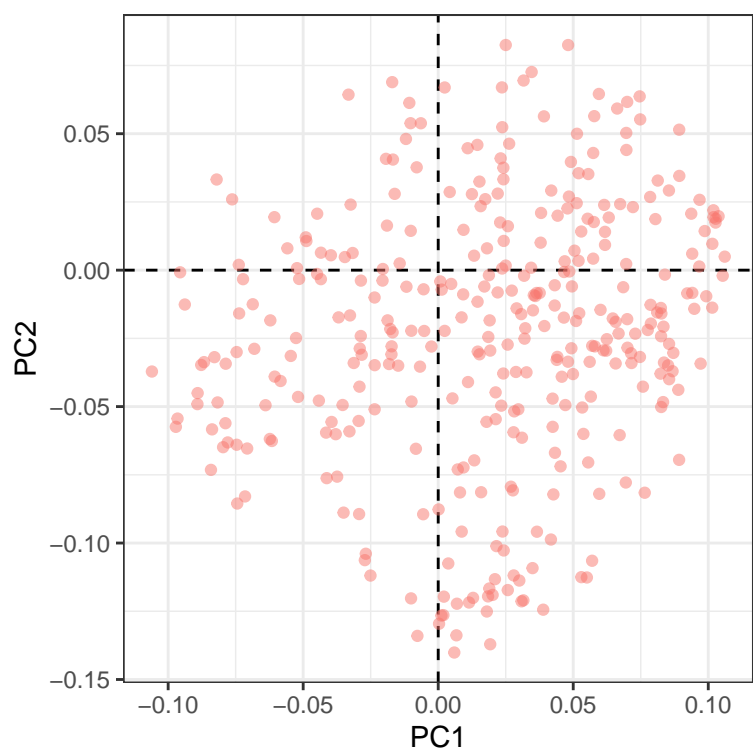

Supplement: Supplemental Information 2 [file peerj-11-15112-s002.zip › peerj-75361-Raw_data_result/Raw data/Result-X101SC21103966-Z01-J001-B1-42/1.MetQuant-QC/Samples_QC_pos-pcaloading.pdf]

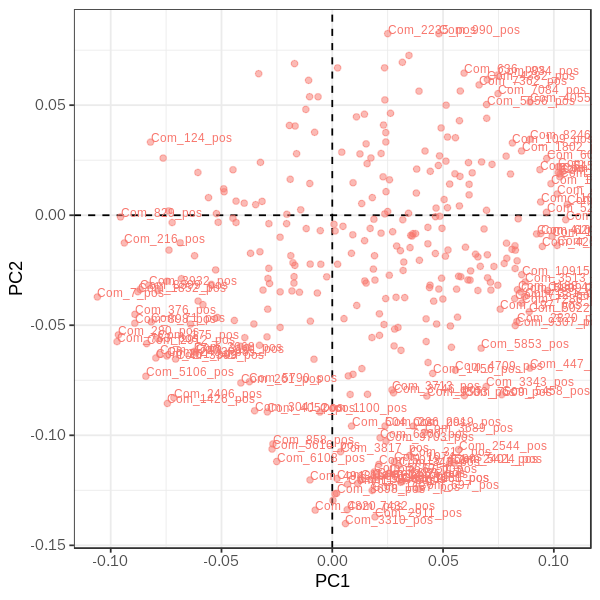

Supplement: Supplemental Information 2 [file peerj-11-15112-s002.zip › peerj-75361-Raw_data_result/Raw data/Result-X101SC21103966-Z01-J001-B1-42/1.MetQuant-QC/Samples_QC_pos-pcaloading.png]

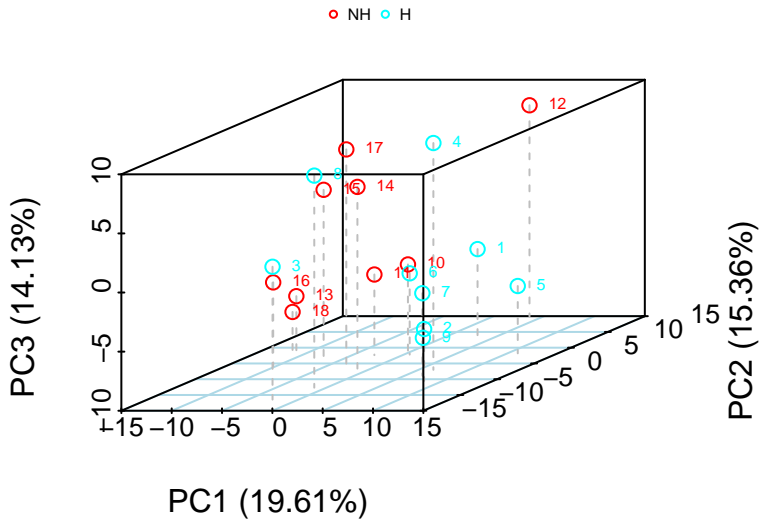

Supplement: Supplemental Information 2 [file peerj-11-15112-s002.zip › peerj-75361-Raw_data_result/Raw data/Result-X101SC21103966-Z01-J001-B1-42/1.MetQuant-QC/Samples_neg-PCA.3D.pdf]

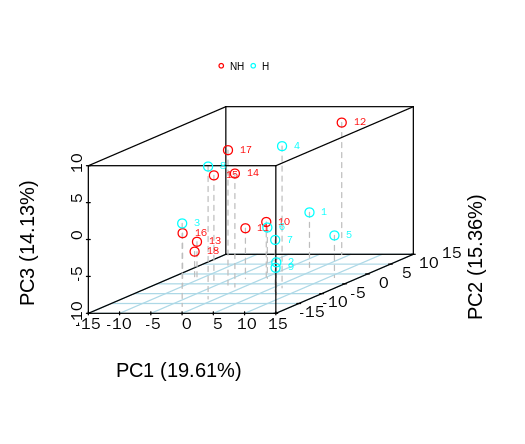

Supplement: Supplemental Information 2 [file peerj-11-15112-s002.zip › peerj-75361-Raw_data_result/Raw data/Result-X101SC21103966-Z01-J001-B1-42/1.MetQuant-QC/Samples_neg-PCA.3D.png]

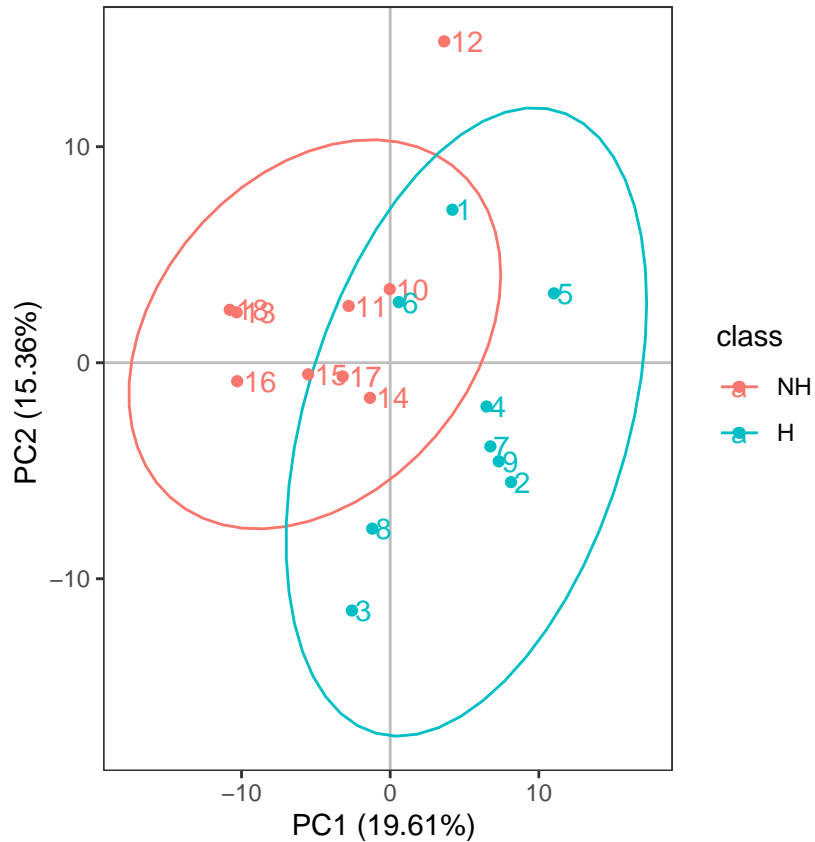

Supplement: Supplemental Information 2 [file peerj-11-15112-s002.zip › peerj-75361-Raw_data_result/Raw data/Result-X101SC21103966-Z01-J001-B1-42/1.MetQuant-QC/Samples_neg-PCA.pdf]

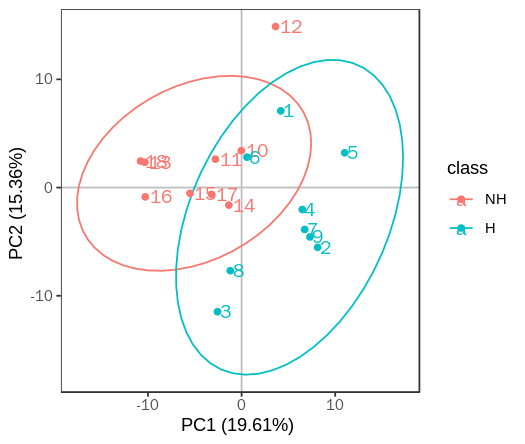

Supplement: Supplemental Information 2 [file peerj-11-15112-s002.zip › peerj-75361-Raw_data_result/Raw data/Result-X101SC21103966-Z01-J001-B1-42/1.MetQuant-QC/Samples_neg-PCA.png]

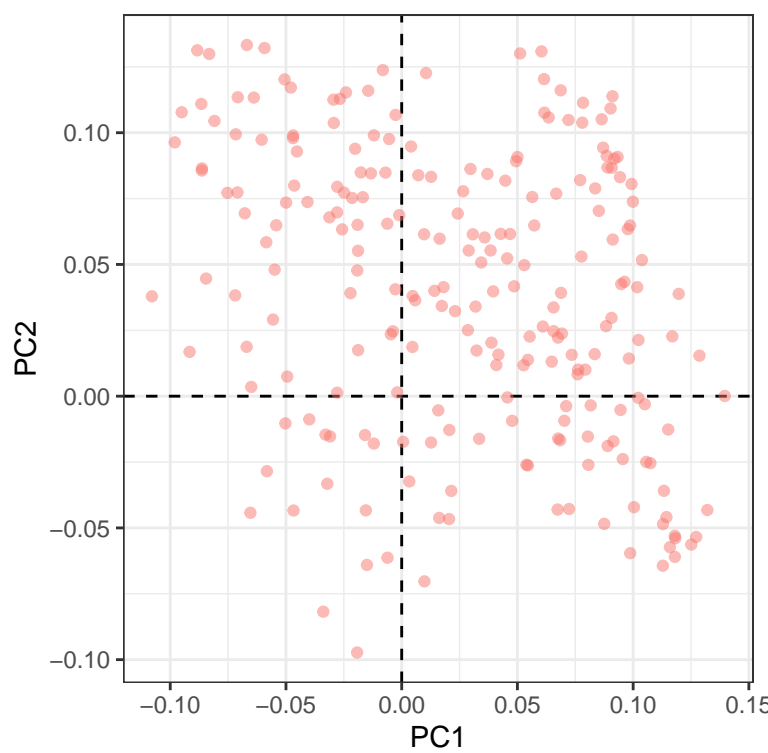

Supplement: Supplemental Information 2 [file peerj-11-15112-s002.zip › peerj-75361-Raw_data_result/Raw data/Result-X101SC21103966-Z01-J001-B1-42/1.MetQuant-QC/Samples_neg-pcaloading.pdf]

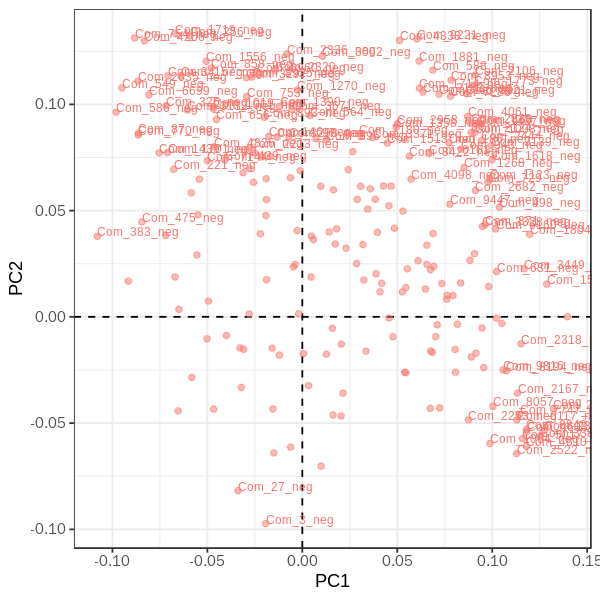

Supplement: Supplemental Information 2 [file peerj-11-15112-s002.zip › peerj-75361-Raw_data_result/Raw data/Result-X101SC21103966-Z01-J001-B1-42/1.MetQuant-QC/Samples_neg-pcaloading.png]

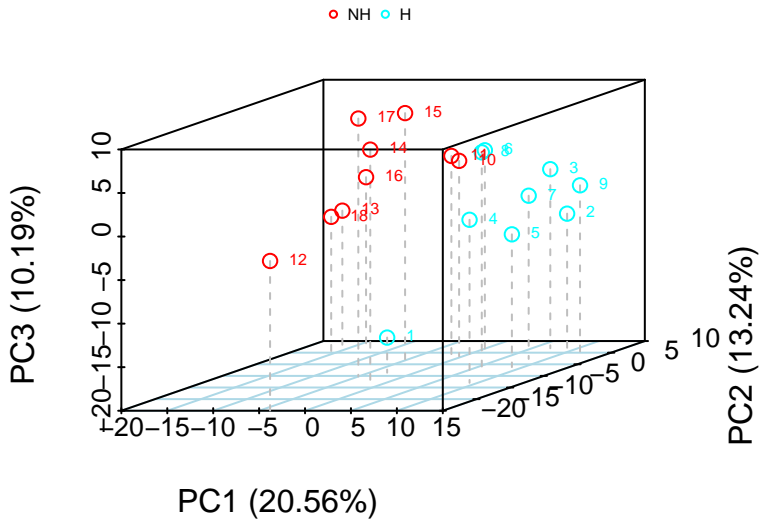

Supplement: Supplemental Information 2 [file peerj-11-15112-s002.zip › peerj-75361-Raw_data_result/Raw data/Result-X101SC21103966-Z01-J001-B1-42/1.MetQuant-QC/Samples_pos-PCA.3D.pdf]

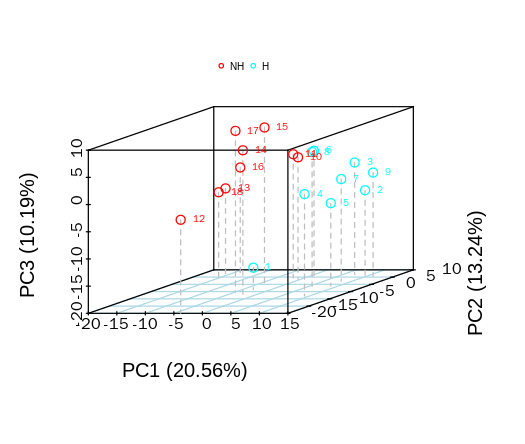

Supplement: Supplemental Information 2 [file peerj-11-15112-s002.zip › peerj-75361-Raw_data_result/Raw data/Result-X101SC21103966-Z01-J001-B1-42/1.MetQuant-QC/Samples_pos-PCA.3D.png]

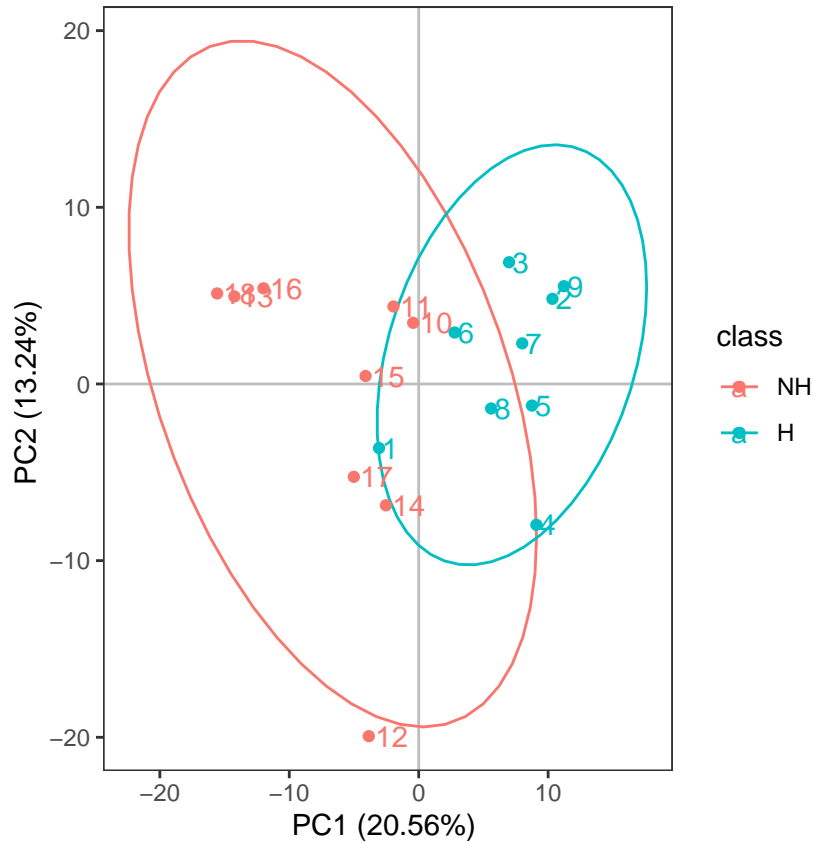

Supplement: Supplemental Information 2 [file peerj-11-15112-s002.zip › peerj-75361-Raw_data_result/Raw data/Result-X101SC21103966-Z01-J001-B1-42/1.MetQuant-QC/Samples_pos-PCA.pdf]

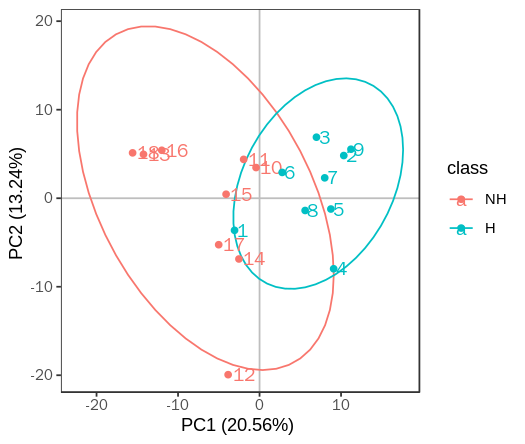

Supplement: Supplemental Information 2 [file peerj-11-15112-s002.zip › peerj-75361-Raw_data_result/Raw data/Result-X101SC21103966-Z01-J001-B1-42/1.MetQuant-QC/Samples_pos-PCA.png]

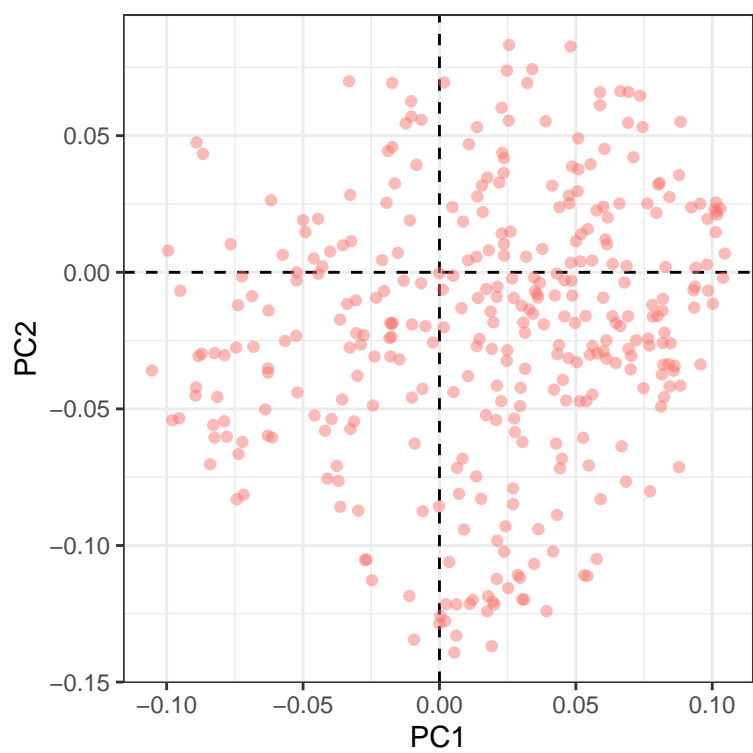

Supplement: Supplemental Information 2 [file peerj-11-15112-s002.zip › peerj-75361-Raw_data_result/Raw data/Result-X101SC21103966-Z01-J001-B1-42/1.MetQuant-QC/Samples_pos-pcaloading.pdf]

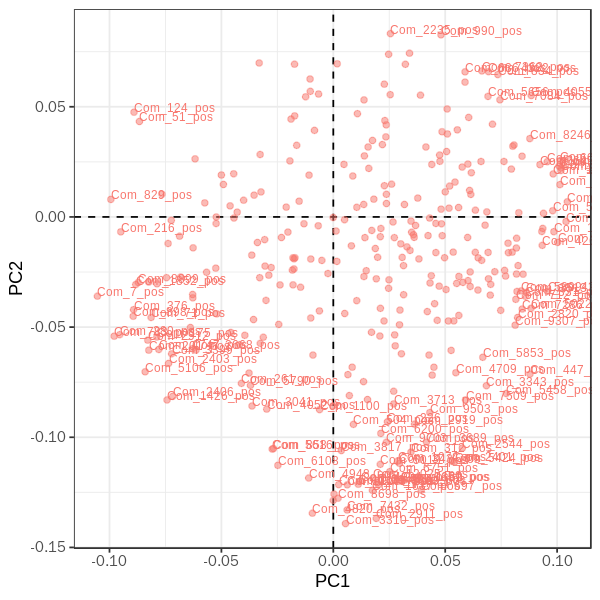

Supplement: Supplemental Information 2 [file peerj-11-15112-s002.zip › peerj-75361-Raw_data_result/Raw data/Result-X101SC21103966-Z01-J001-B1-42/1.MetQuant-QC/Samples_pos-pcaloading.png]

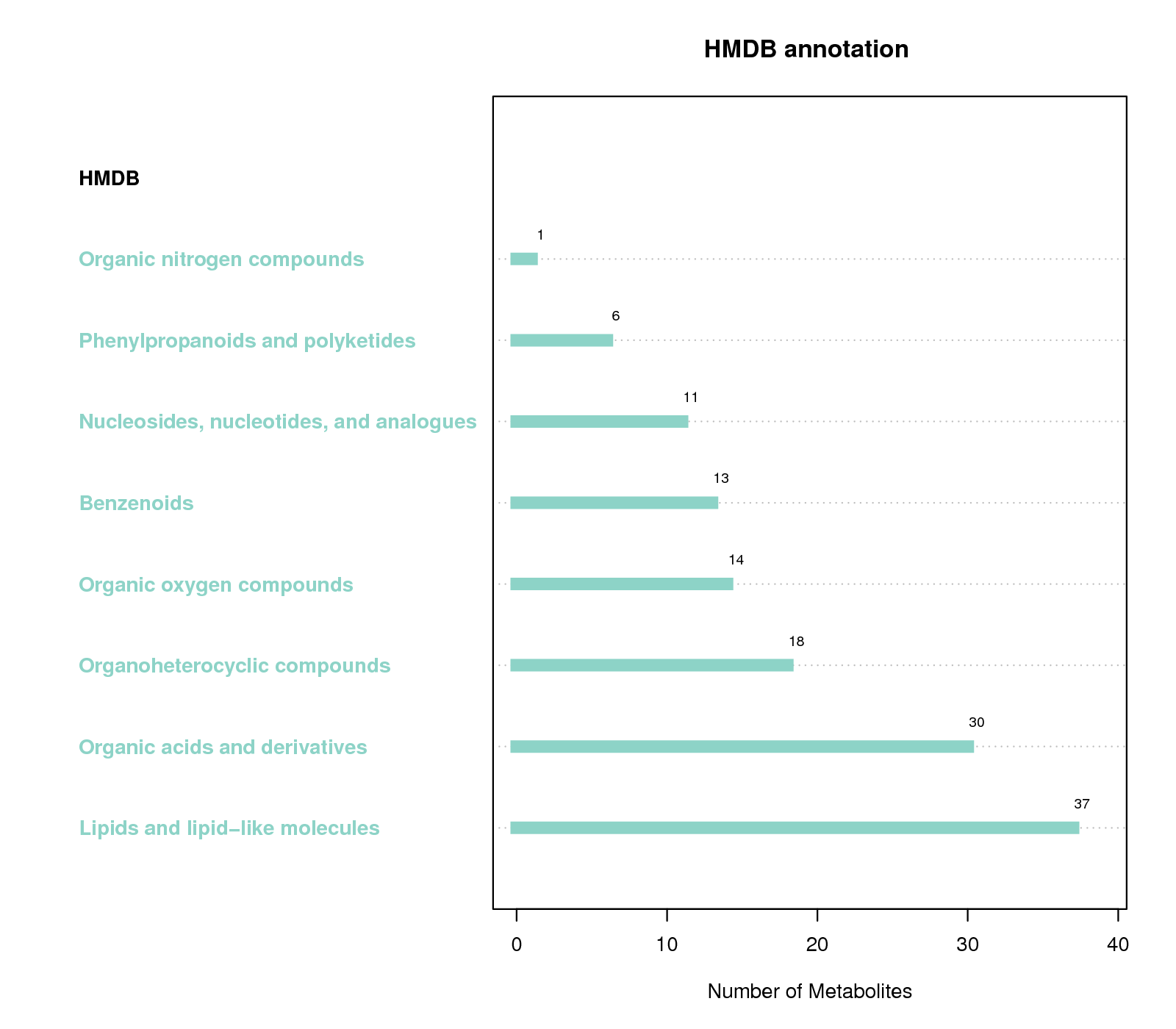

Supplement: Supplemental Information 2 [file peerj-11-15112-s002.zip › peerj-75361-Raw_data_result/Raw data/Result-X101SC21103966-Z01-J001-B1-42/2.MetAnnotation/HMDB/meta_neg.HMDB.Anno.png]

## HMDB annotation

HMDB

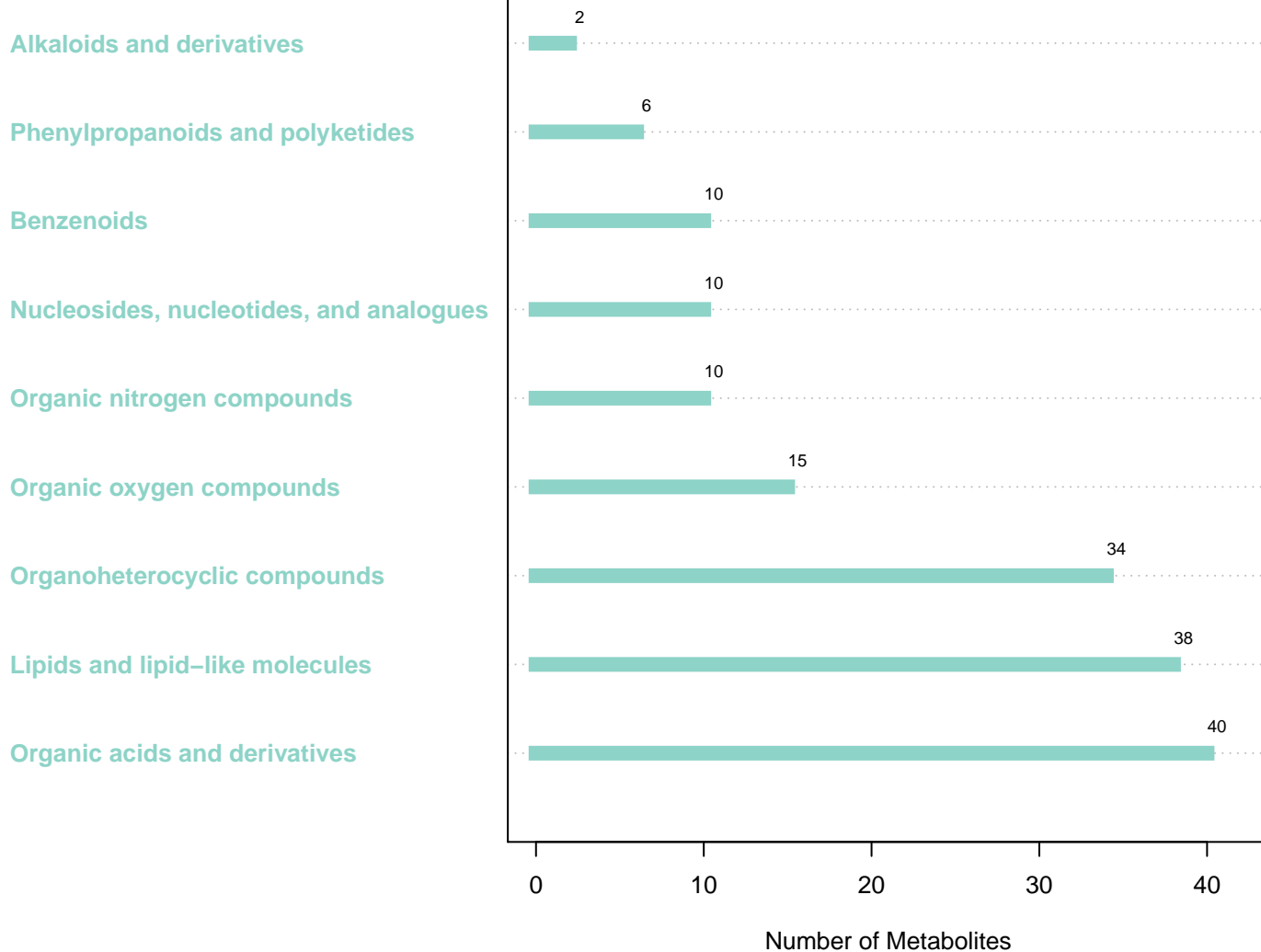

Supplement: Supplemental Information 2 [file peerj-11-15112-s002.zip › peerj-75361-Raw_data_result/Raw data/Result-X101SC21103966-Z01-J001-B1-42/2.MetAnnotation/HMDB/meta_pos.HMDB.Anno.pdf]

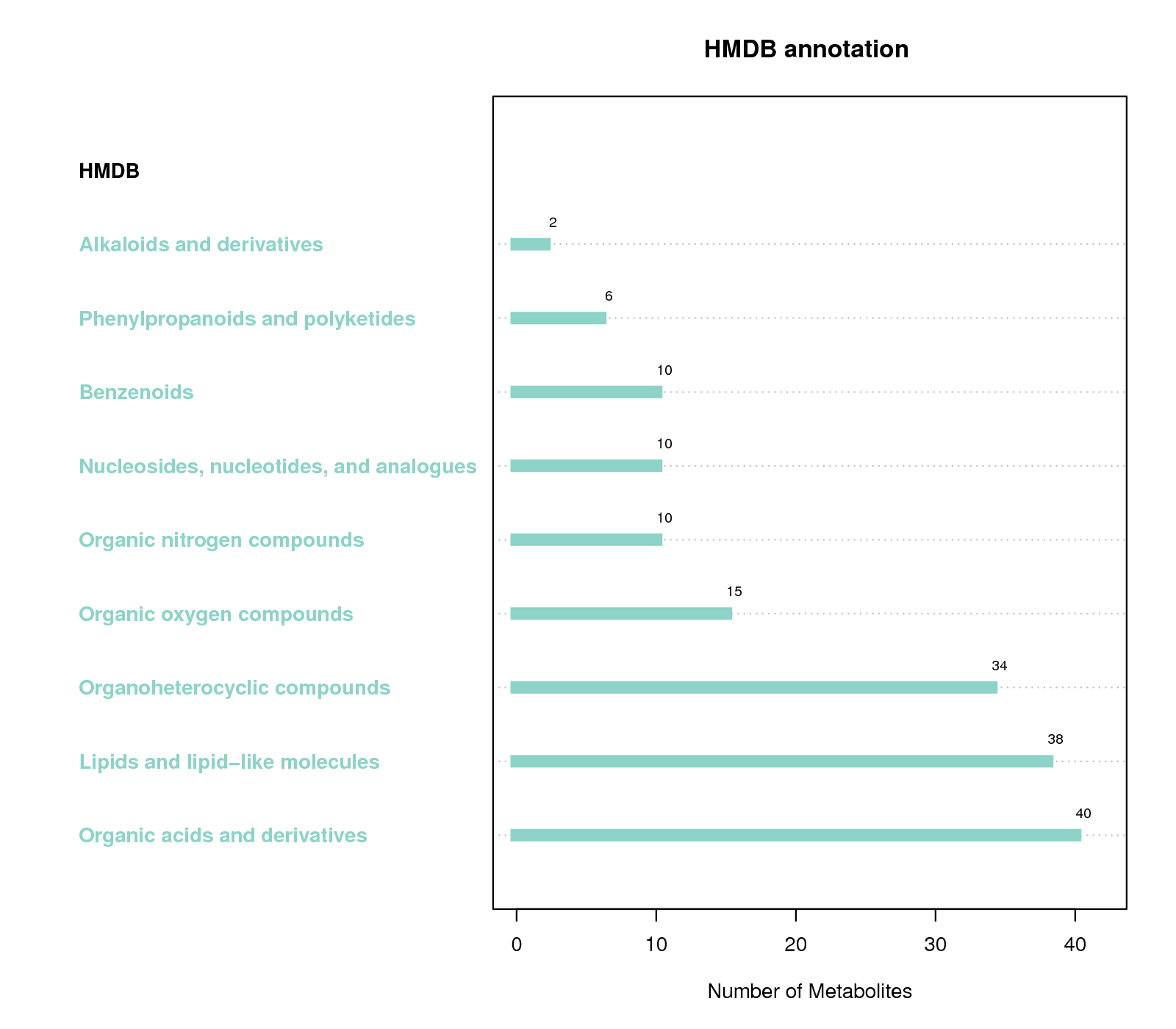

Supplement: Supplemental Information 2 [file peerj-11-15112-s002.zip › peerj-75361-Raw_data_result/Raw data/Result-X101SC21103966-Z01-J001-B1-42/2.MetAnnotation/HMDB/meta_pos.HMDB.Anno.png]

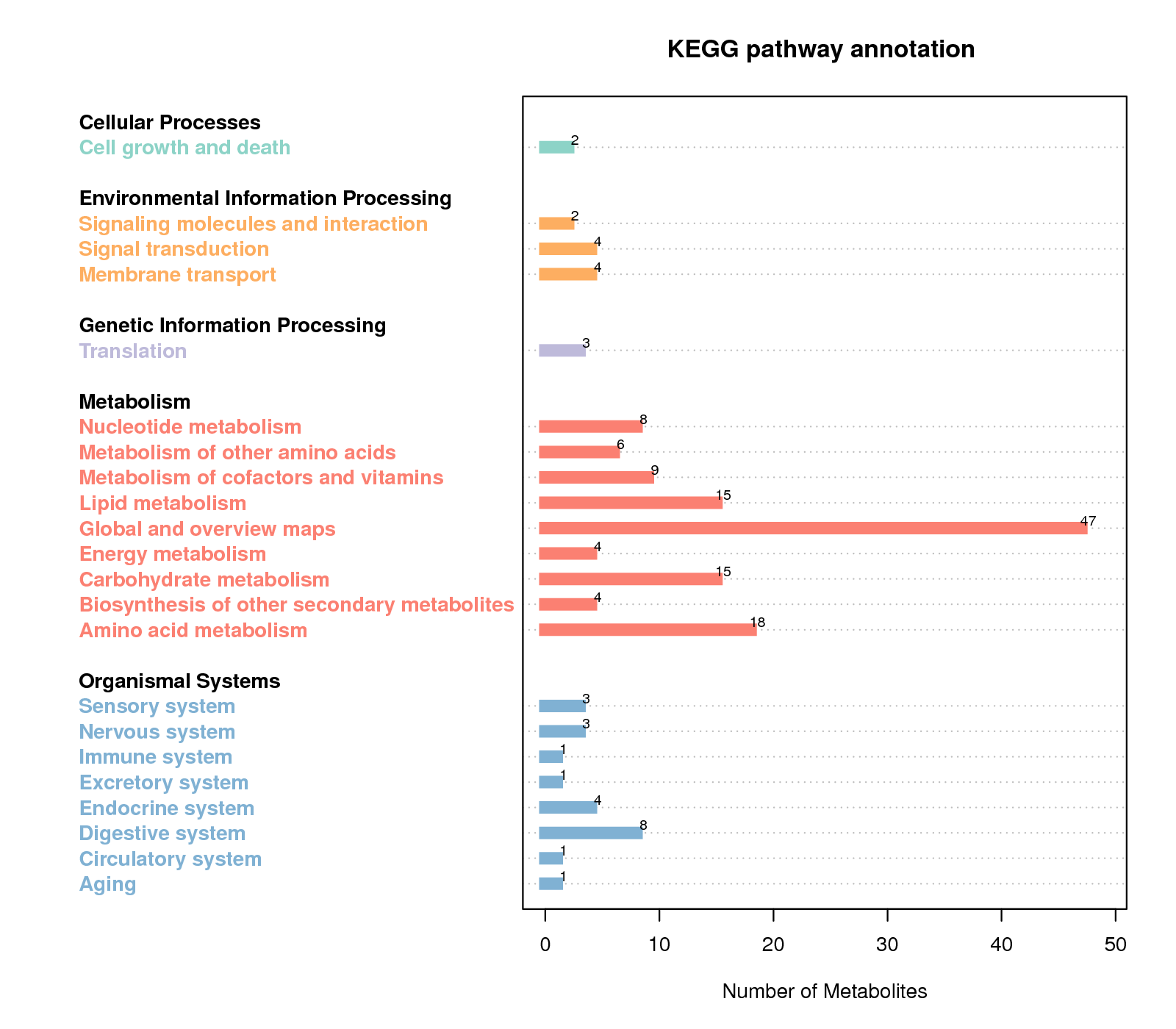

Supplement: Supplemental Information 2 [file peerj-11-15112-s002.zip › peerj-75361-Raw_data_result/Raw data/Result-X101SC21103966-Z01-J001-B1-42/2.MetAnnotation/KEGG/meta_neg.KEGG.Anno.png]

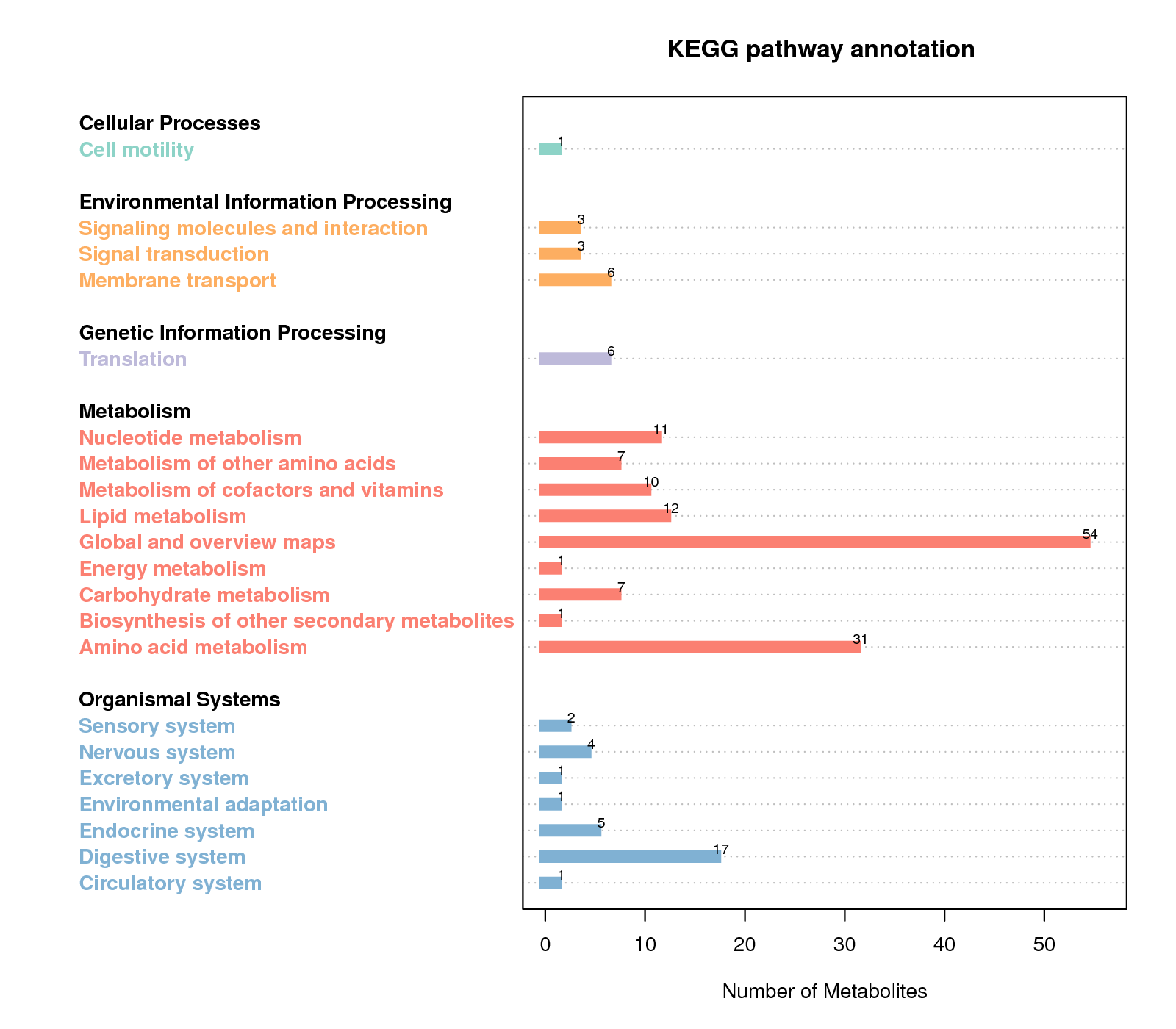

Supplement: Supplemental Information 2 [file peerj-11-15112-s002.zip › peerj-75361-Raw_data_result/Raw data/Result-X101SC21103966-Z01-J001-B1-42/2.MetAnnotation/KEGG/meta_pos.KEGG.Anno.png]

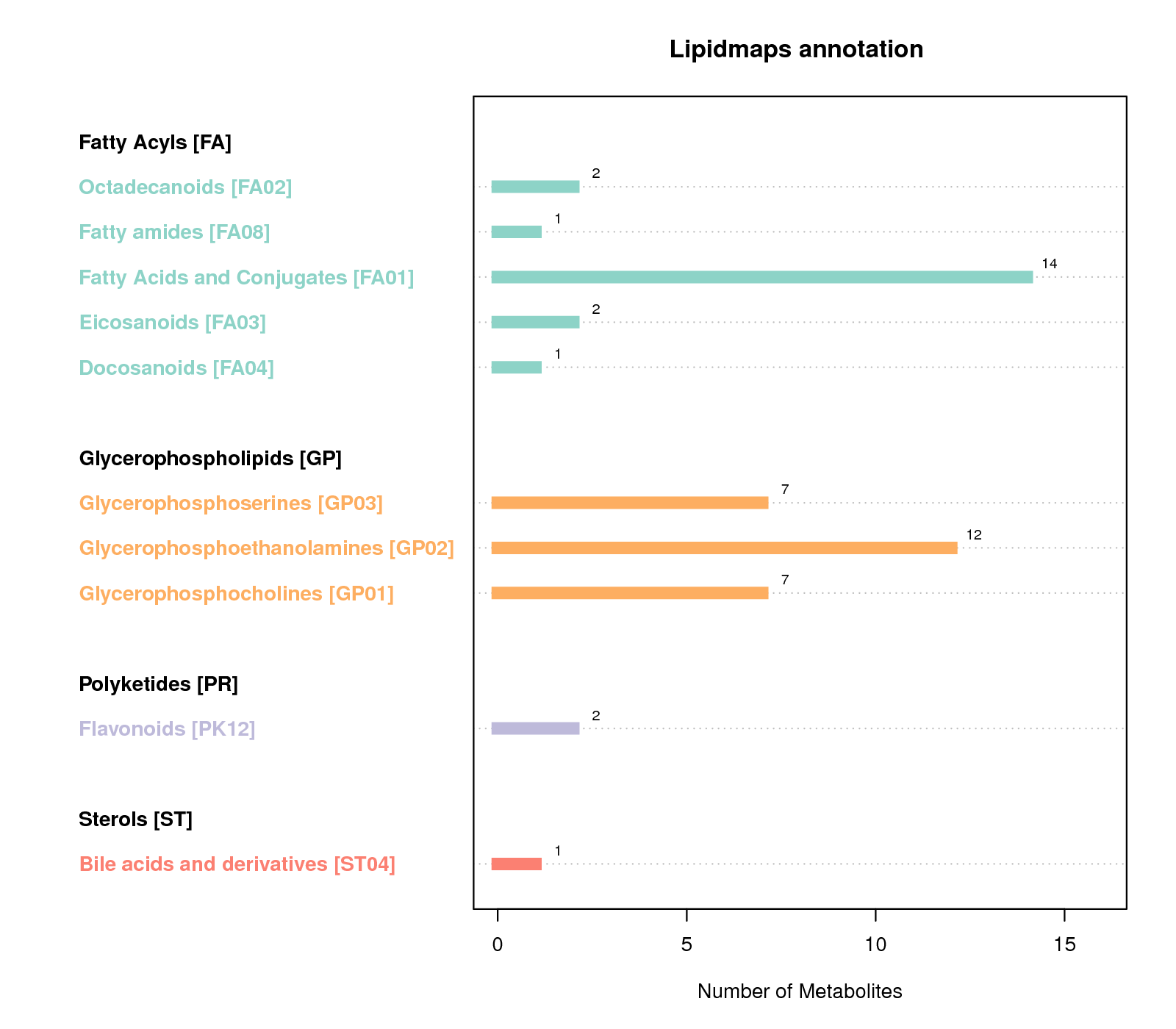

Supplement: Supplemental Information 2 [file peerj-11-15112-s002.zip › peerj-75361-Raw_data_result/Raw data/Result-X101SC21103966-Z01-J001-B1-42/2.MetAnnotation/Lipidmaps/meta_neg.Lipidmaps.Anno.png]

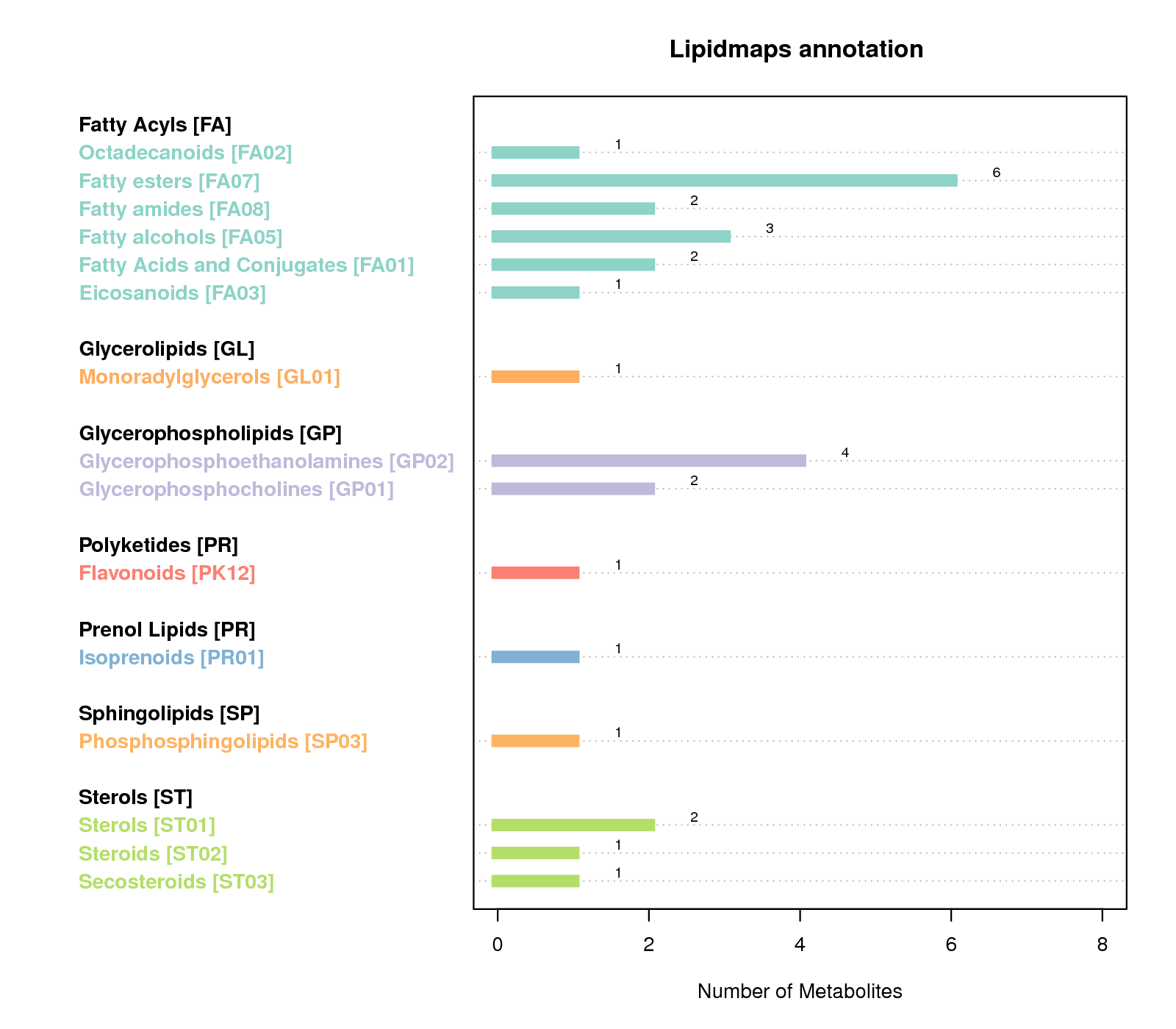

Supplement: Supplemental Information 2 [file peerj-11-15112-s002.zip › peerj-75361-Raw_data_result/Raw data/Result-X101SC21103966-Z01-J001-B1-42/2.MetAnnotation/Lipidmaps/meta_pos.Lipidmaps.Anno.png]

H.vs.NH

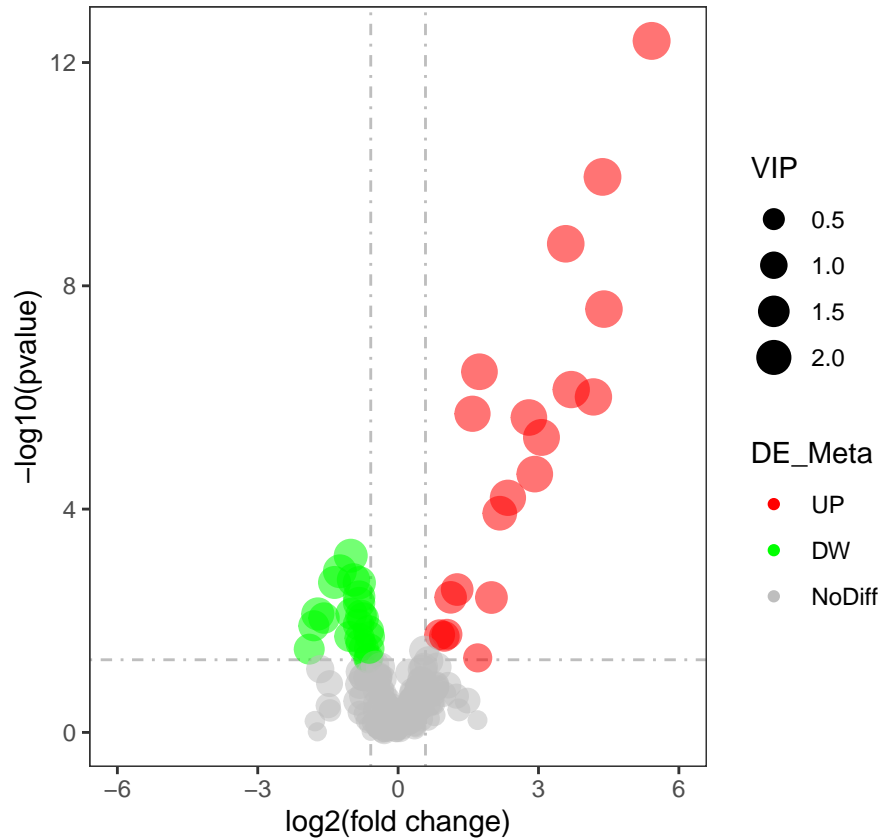

Supplement: Supplemental Information 2 [file peerj-11-15112-s002.zip › peerj-75361-Raw_data_result/Raw data/Result-X101SC21103966-Z01-J001-B1-42/3.MetDiffScreening/H.vs.NH/H.vs.NH_neg.xls.volcano.pdf]

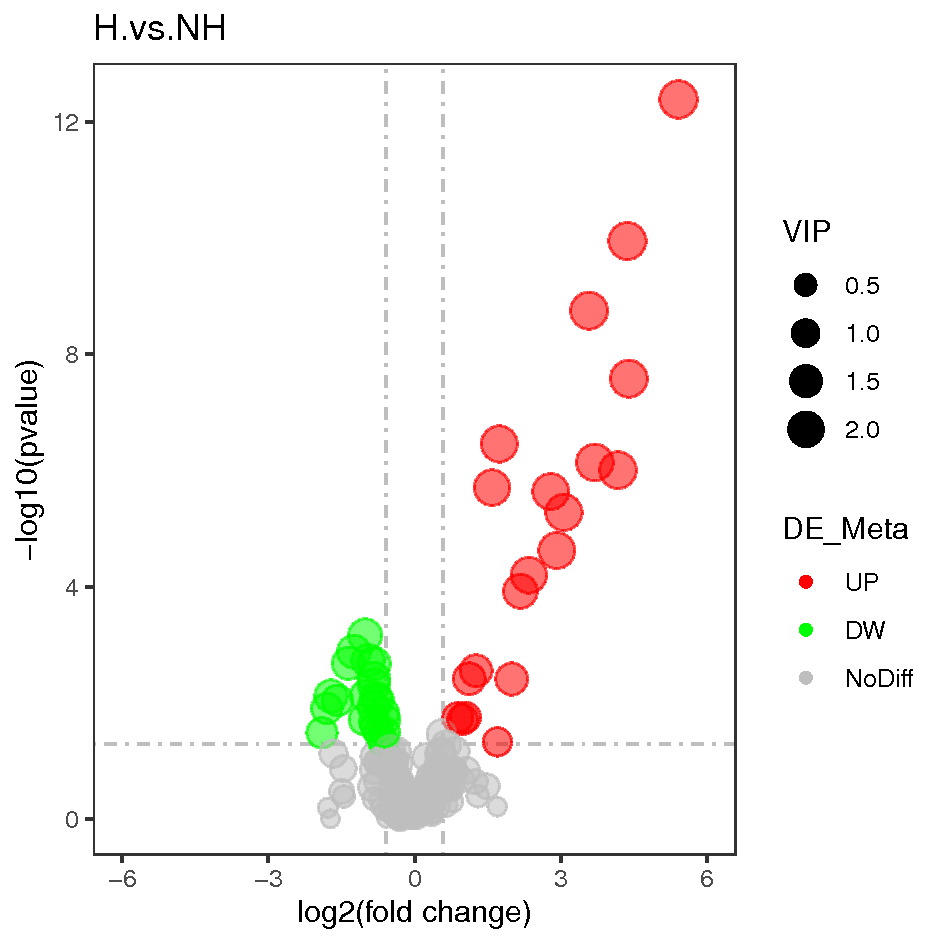

Supplement: Supplemental Information 2 [file peerj-11-15112-s002.zip › peerj-75361-Raw_data_result/Raw data/Result-X101SC21103966-Z01-J001-B1-42/3.MetDiffScreening/H.vs.NH/H.vs.NH_neg.xls.volcano.png]

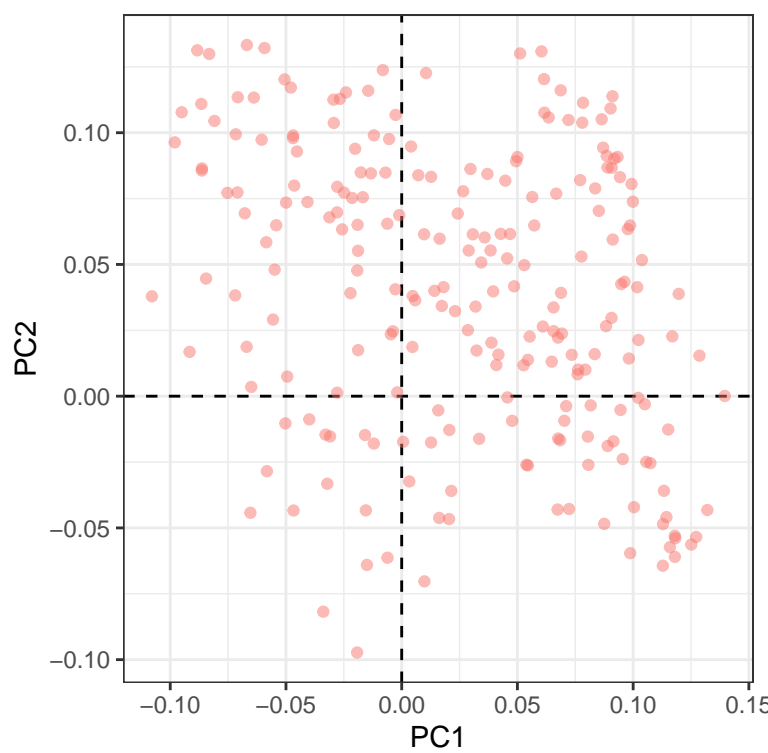

Supplement: Supplemental Information 2 [file peerj-11-15112-s002.zip › peerj-75361-Raw_data_result/Raw data/Result-X101SC21103966-Z01-J001-B1-42/3.MetDiffScreening/H.vs.NH/H.vs.NH_neg_PCA-pcaloading.pdf]

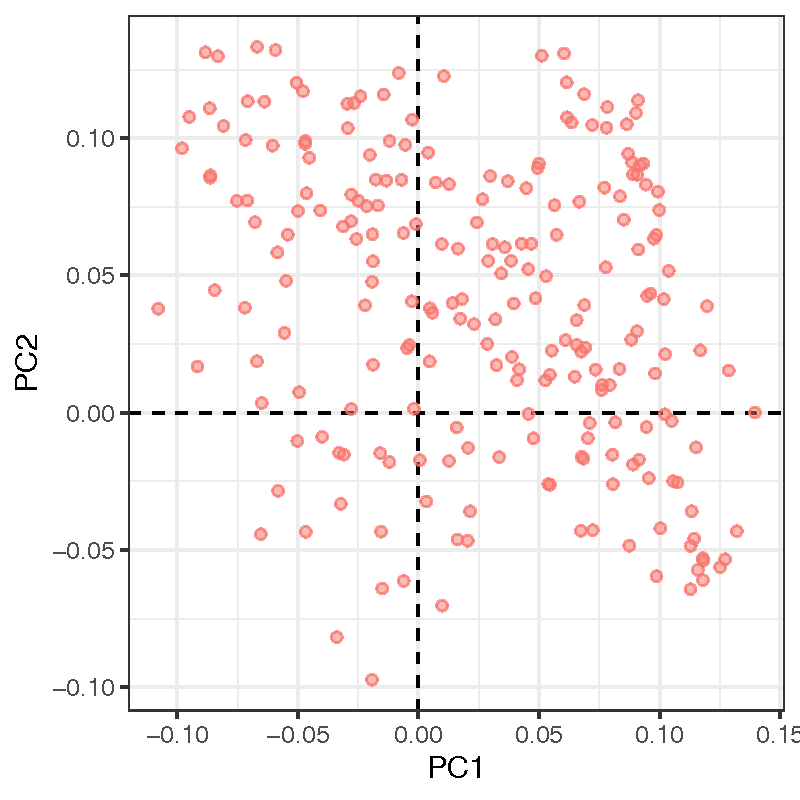

Supplement: Supplemental Information 2 [file peerj-11-15112-s002.zip › peerj-75361-Raw_data_result/Raw data/Result-X101SC21103966-Z01-J001-B1-42/3.MetDiffScreening/H.vs.NH/H.vs.NH_neg_PCA-pcaloading.png]

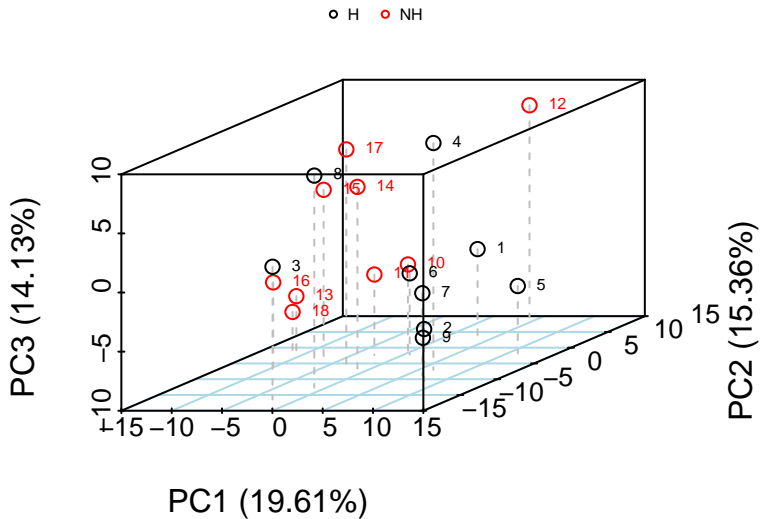

Supplement: Supplemental Information 2 [file peerj-11-15112-s002.zip › peerj-75361-Raw_data_result/Raw data/Result-X101SC21103966-Z01-J001-B1-42/3.MetDiffScreening/H.vs.NH/H.vs.NH_neg_PCA.3D.pdf]

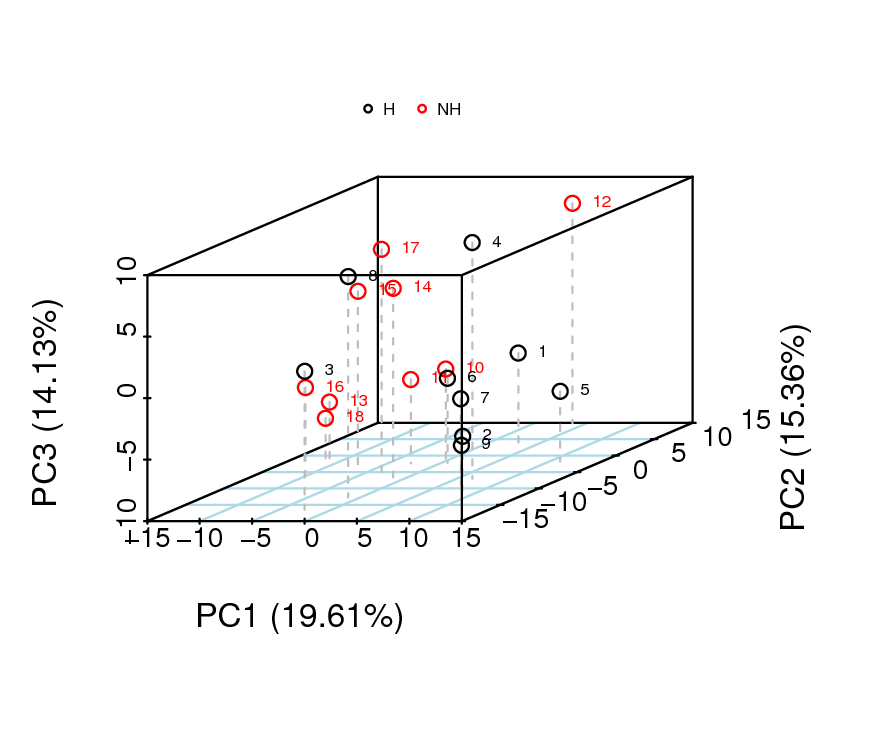

Supplement: Supplemental Information 2 [file peerj-11-15112-s002.zip › peerj-75361-Raw_data_result/Raw data/Result-X101SC21103966-Z01-J001-B1-42/3.MetDiffScreening/H.vs.NH/H.vs.NH_neg_PCA.3D.png]

class    —●— H    —●— NH

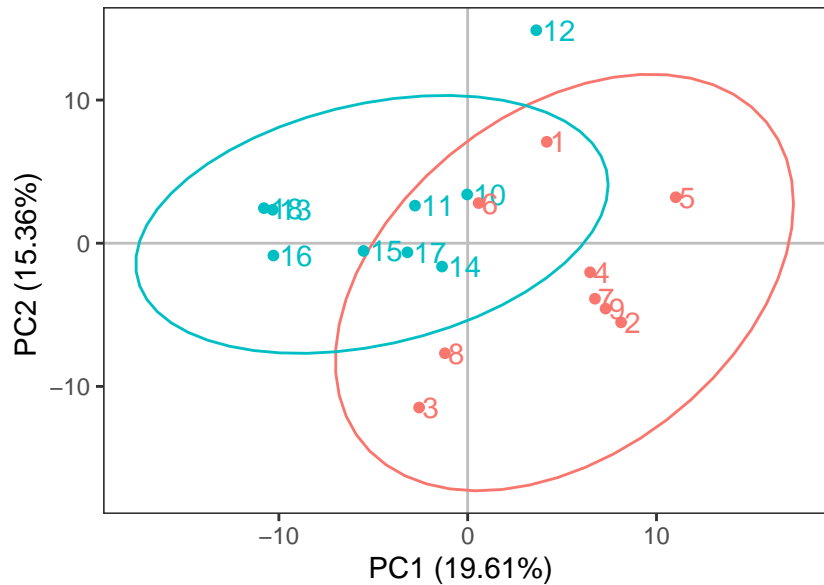

Supplement: Supplemental Information 2 [file peerj-11-15112-s002.zip › peerj-75361-Raw_data_result/Raw data/Result-X101SC21103966-Z01-J001-B1-42/3.MetDiffScreening/H.vs.NH/H.vs.NH_neg_PCA.pdf]

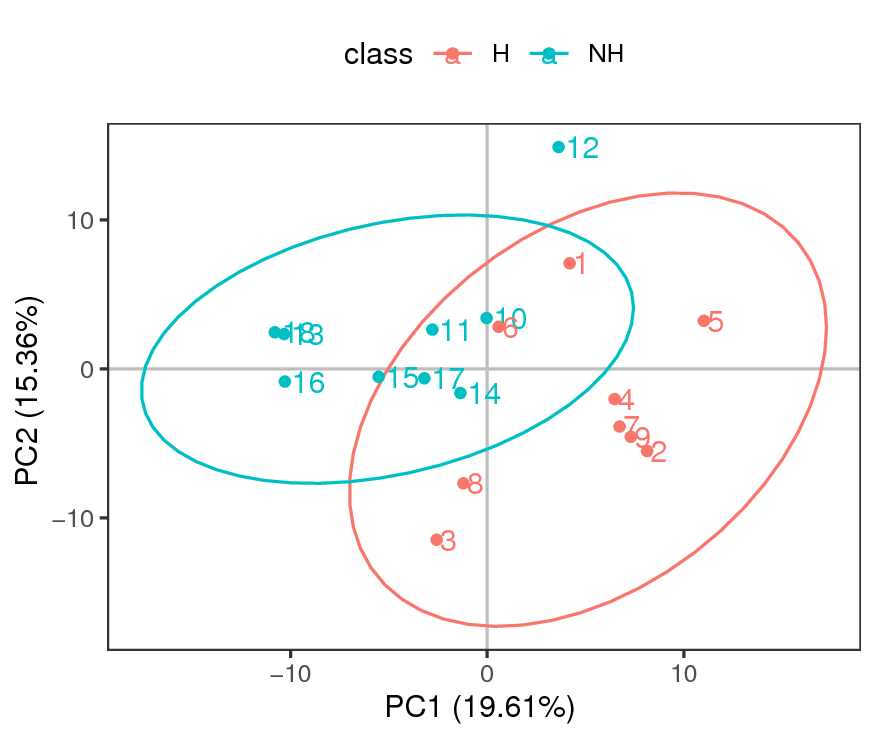

Supplement: Supplemental Information 2 [file peerj-11-15112-s002.zip › peerj-75361-Raw_data_result/Raw data/Result-X101SC21103966-Z01-J001-B1-42/3.MetDiffScreening/H.vs.NH/H.vs.NH_neg_PCA.png]

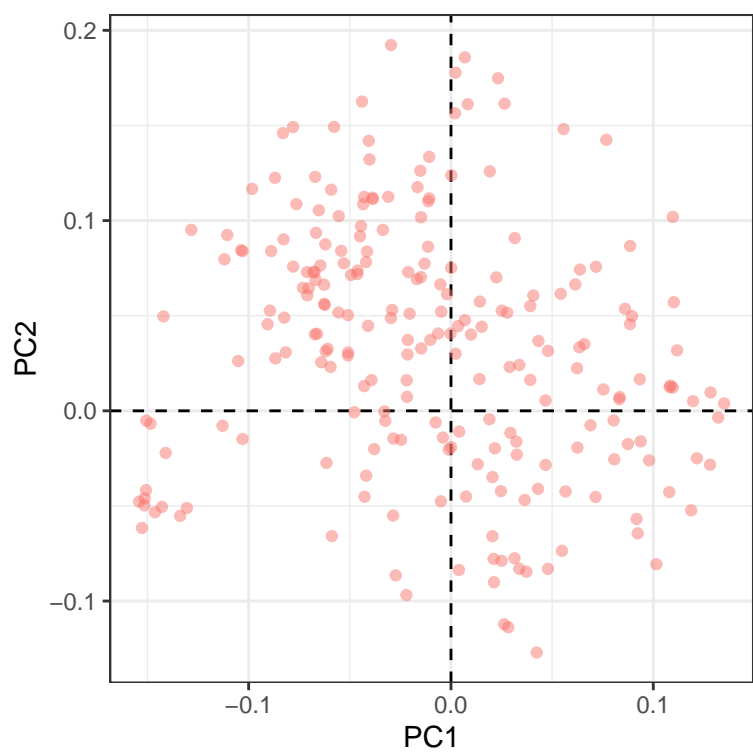

Supplement: Supplemental Information 2 [file peerj-11-15112-s002.zip › peerj-75361-Raw_data_result/Raw data/Result-X101SC21103966-Z01-J001-B1-42/3.MetDiffScreening/H.vs.NH/H.vs.NH_neg_PLSDA-loading.pdf]

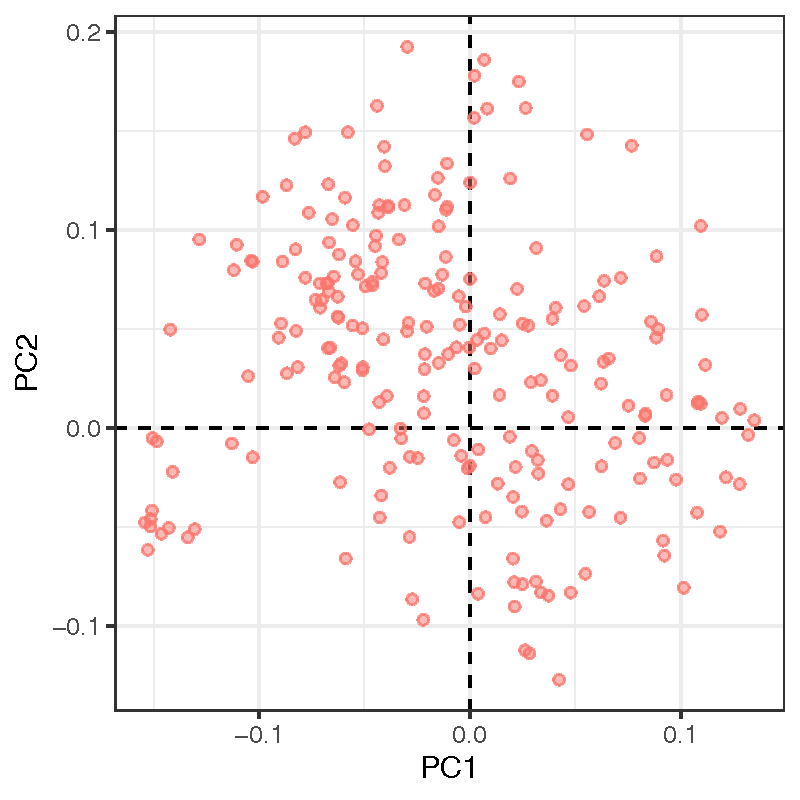

Supplement: Supplemental Information 2 [file peerj-11-15112-s002.zip › peerj-75361-Raw_data_result/Raw data/Result-X101SC21103966-Z01-J001-B1-42/3.MetDiffScreening/H.vs.NH/H.vs.NH_neg_PLSDA-loading.png]

class    —●— H    —●— NH

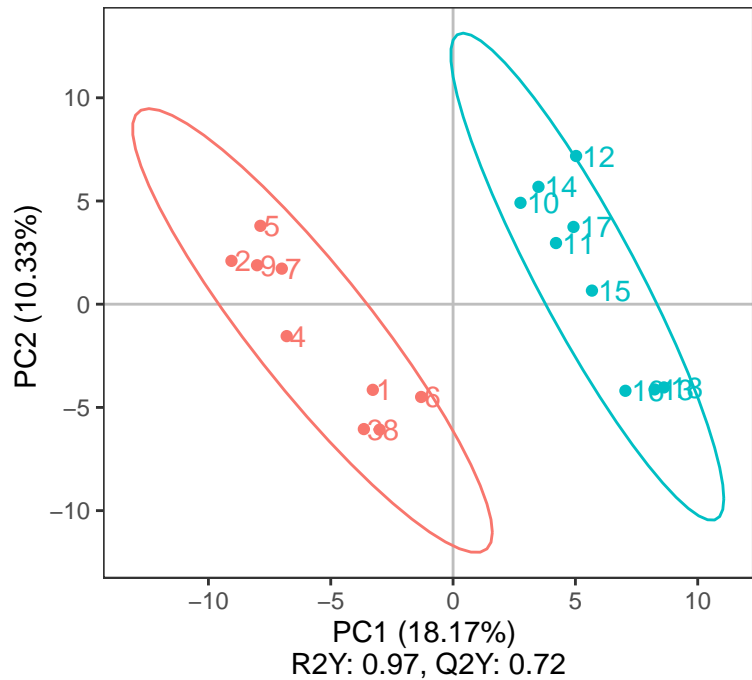

Supplement: Supplemental Information 2 [file peerj-11-15112-s002.zip › peerj-75361-Raw_data_result/Raw data/Result-X101SC21103966-Z01-J001-B1-42/3.MetDiffScreening/H.vs.NH/H.vs.NH_neg_PLSDA-score.pdf]

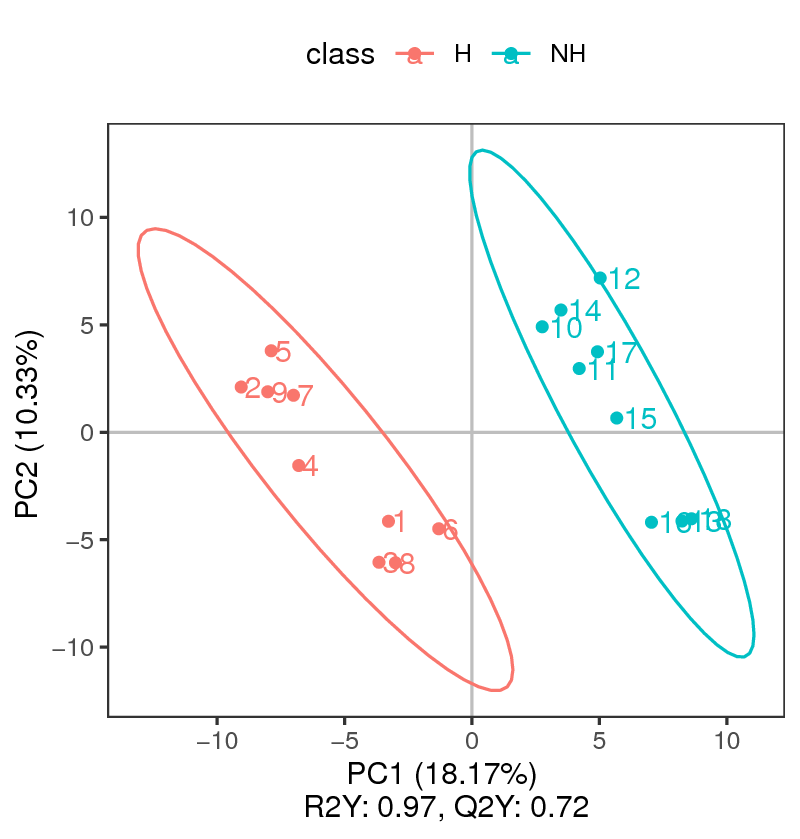

Supplement: Supplemental Information 2 [file peerj-11-15112-s002.zip › peerj-75361-Raw_data_result/Raw data/Result-X101SC21103966-Z01-J001-B1-42/3.MetDiffScreening/H.vs.NH/H.vs.NH_neg_PLSDA-score.png]

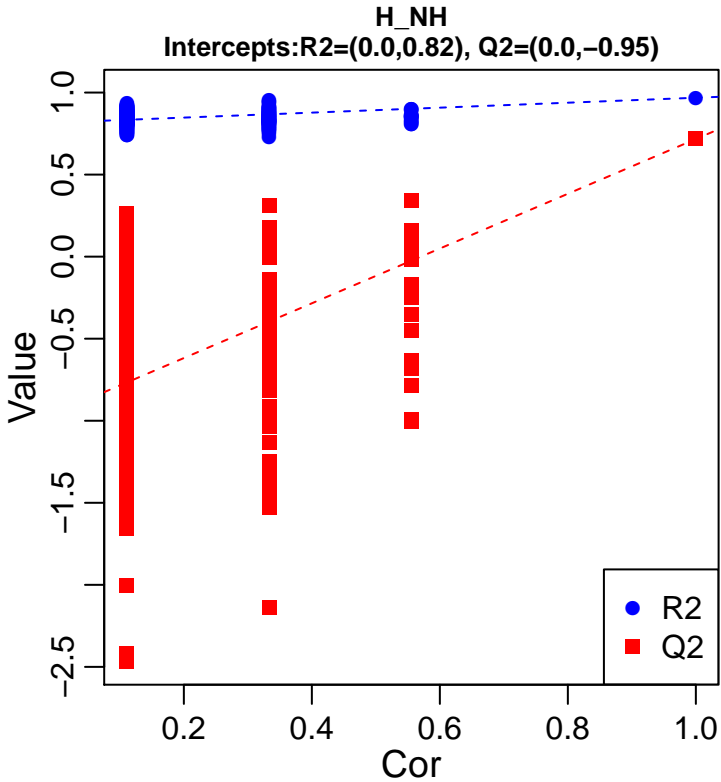

Supplement: Supplemental Information 2 [file peerj-11-15112-s002.zip › peerj-75361-Raw_data_result/Raw data/Result-X101SC21103966-Z01-J001-B1-42/3.MetDiffScreening/H.vs.NH/H.vs.NH_neg_PLSDA-valid.pdf]

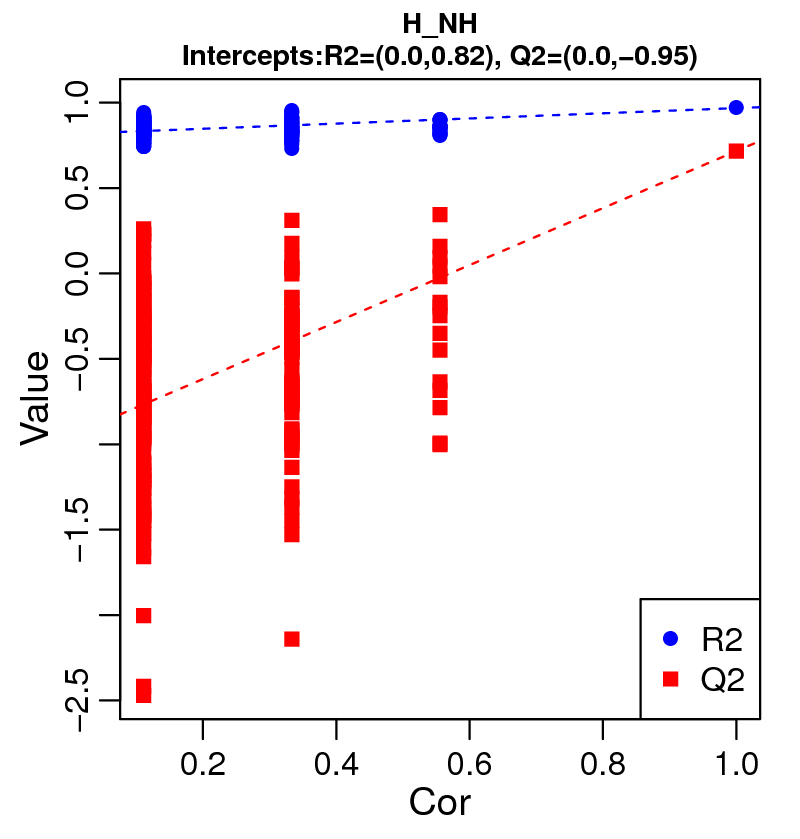

Supplement: Supplemental Information 2 [file peerj-11-15112-s002.zip › peerj-75361-Raw_data_result/Raw data/Result-X101SC21103966-Z01-J001-B1-42/3.MetDiffScreening/H.vs.NH/H.vs.NH_neg_PLSDA-valid.png]

H.vs.NH

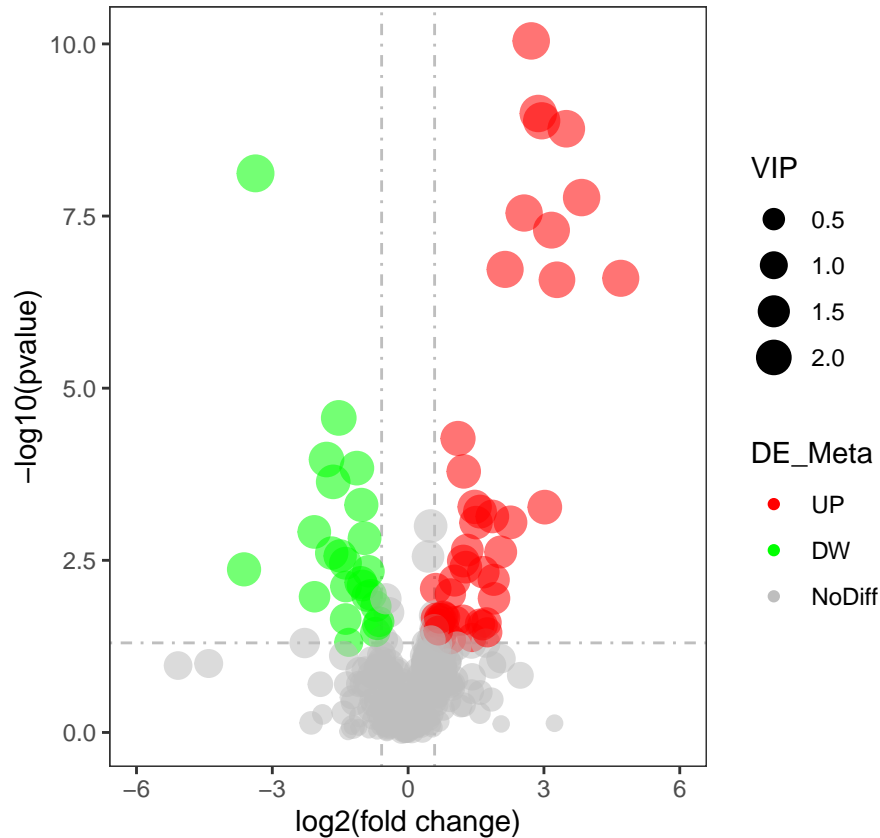

Supplement: Supplemental Information 2 [file peerj-11-15112-s002.zip › peerj-75361-Raw_data_result/Raw data/Result-X101SC21103966-Z01-J001-B1-42/3.MetDiffScreening/H.vs.NH/H.vs.NH_pos.xls.volcano.pdf]

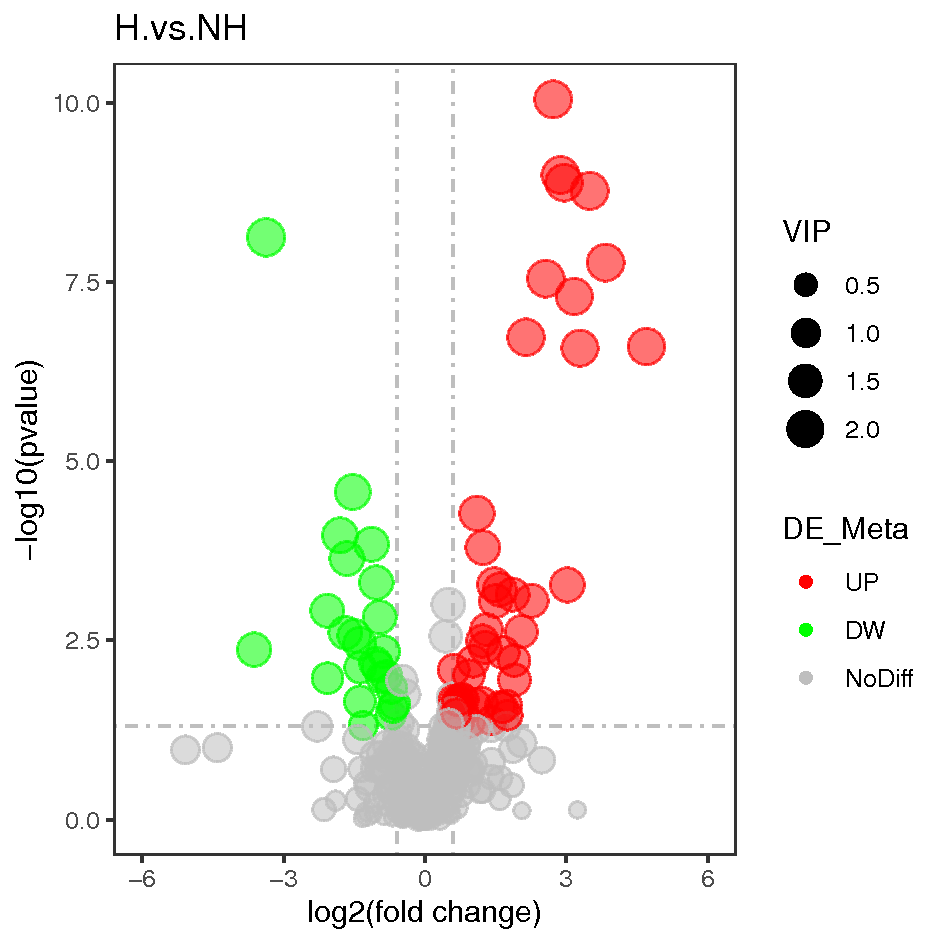

Supplement: Supplemental Information 2 [file peerj-11-15112-s002.zip › peerj-75361-Raw_data_result/Raw data/Result-X101SC21103966-Z01-J001-B1-42/3.MetDiffScreening/H.vs.NH/H.vs.NH_pos.xls.volcano.png]

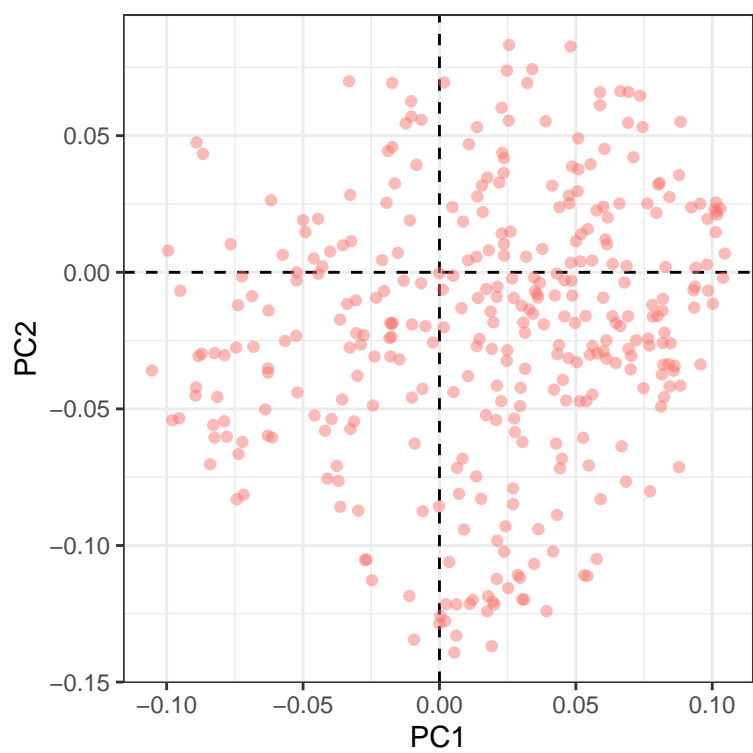

Supplement: Supplemental Information 2 [file peerj-11-15112-s002.zip › peerj-75361-Raw_data_result/Raw data/Result-X101SC21103966-Z01-J001-B1-42/3.MetDiffScreening/H.vs.NH/H.vs.NH_pos_PCA-pcaloading.pdf]

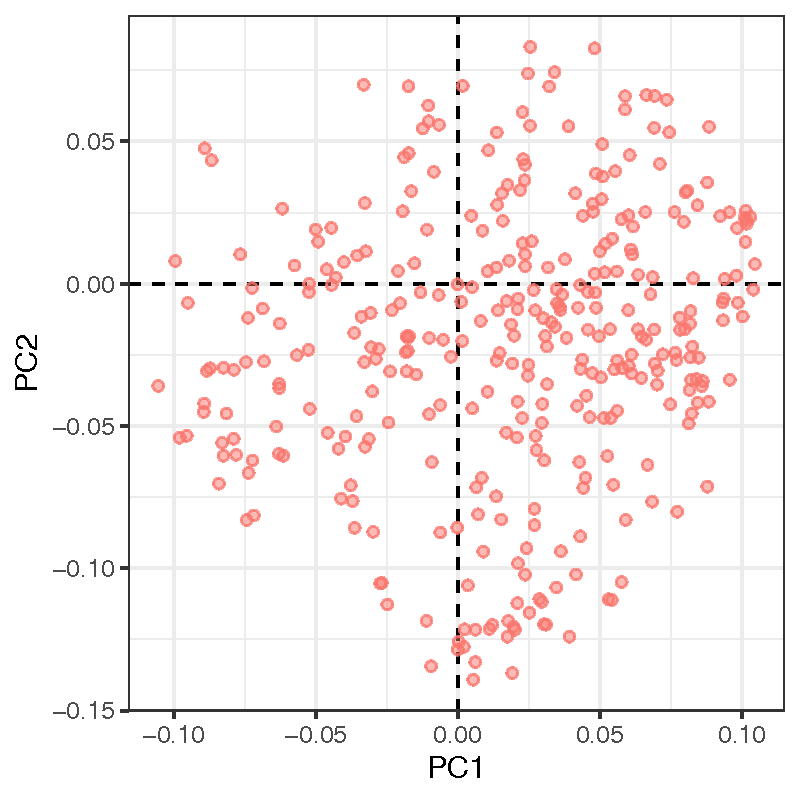

Supplement: Supplemental Information 2 [file peerj-11-15112-s002.zip › peerj-75361-Raw_data_result/Raw data/Result-X101SC21103966-Z01-J001-B1-42/3.MetDiffScreening/H.vs.NH/H.vs.NH_pos_PCA-pcaloading.png]

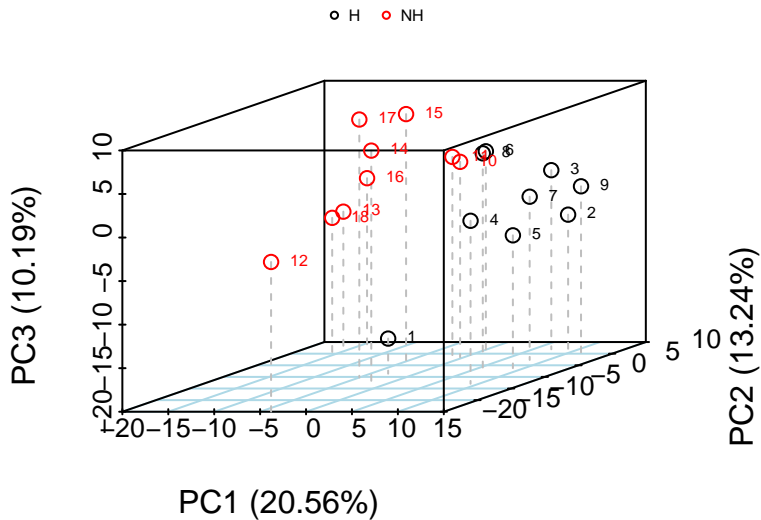

Supplement: Supplemental Information 2 [file peerj-11-15112-s002.zip › peerj-75361-Raw_data_result/Raw data/Result-X101SC21103966-Z01-J001-B1-42/3.MetDiffScreening/H.vs.NH/H.vs.NH_pos_PCA.3D.pdf]

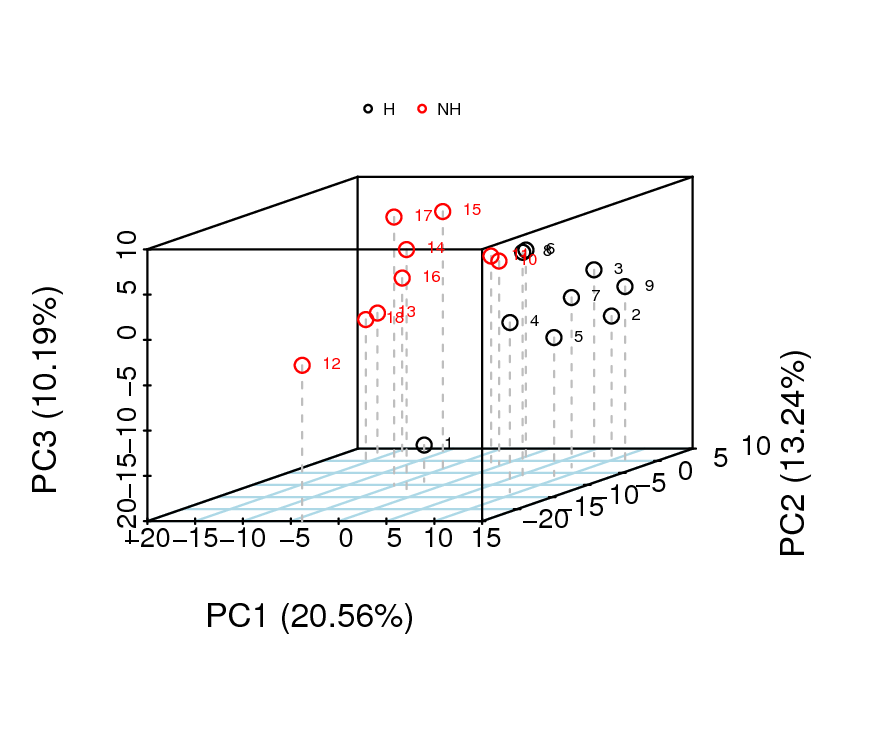

Supplement: Supplemental Information 2 [file peerj-11-15112-s002.zip › peerj-75361-Raw_data_result/Raw data/Result-X101SC21103966-Z01-J001-B1-42/3.MetDiffScreening/H.vs.NH/H.vs.NH_pos_PCA.3D.png]

class    ● H    ● NH

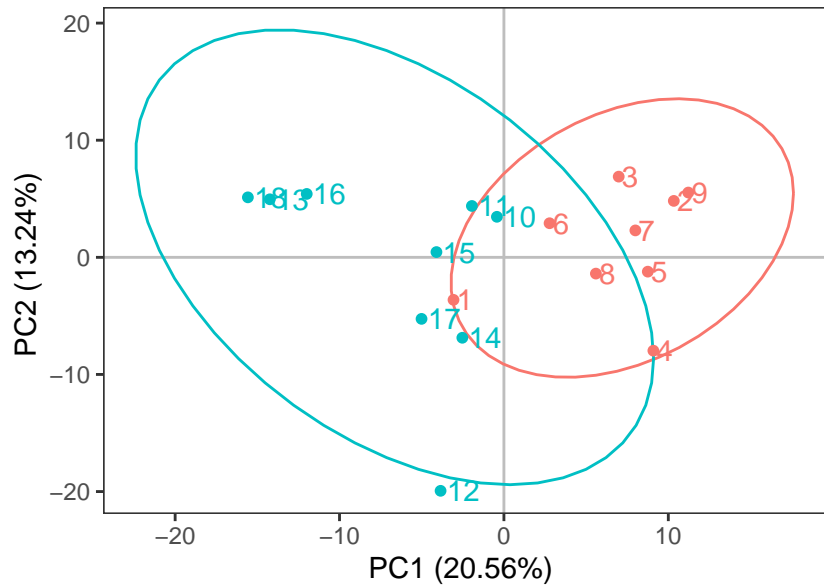

Supplement: Supplemental Information 2 [file peerj-11-15112-s002.zip › peerj-75361-Raw_data_result/Raw data/Result-X101SC21103966-Z01-J001-B1-42/3.MetDiffScreening/H.vs.NH/H.vs.NH_pos_PCA.pdf]

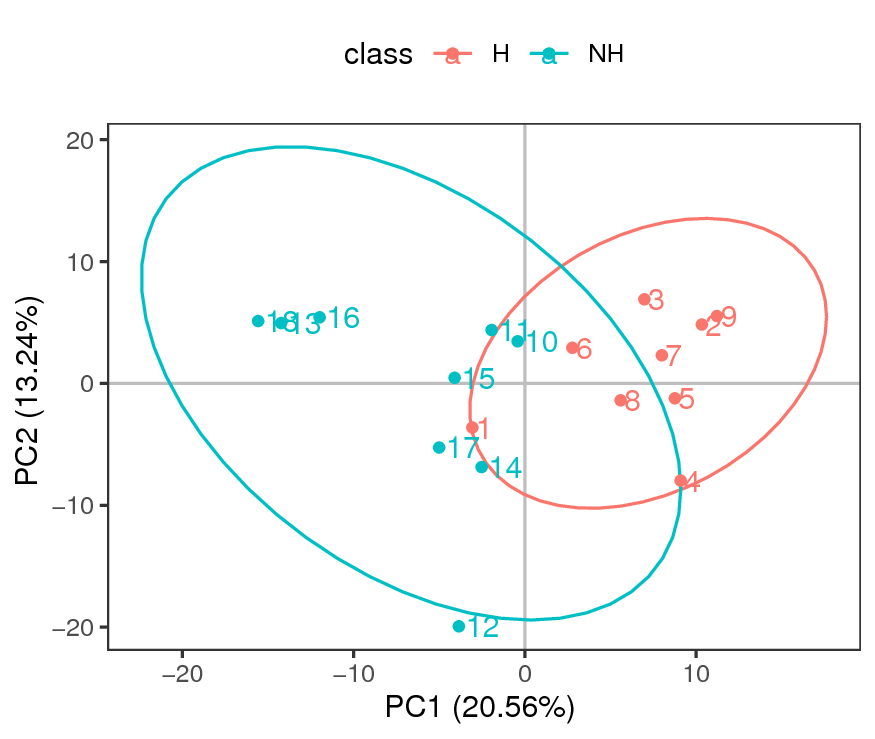

Supplement: Supplemental Information 2 [file peerj-11-15112-s002.zip › peerj-75361-Raw_data_result/Raw data/Result-X101SC21103966-Z01-J001-B1-42/3.MetDiffScreening/H.vs.NH/H.vs.NH_pos_PCA.png]

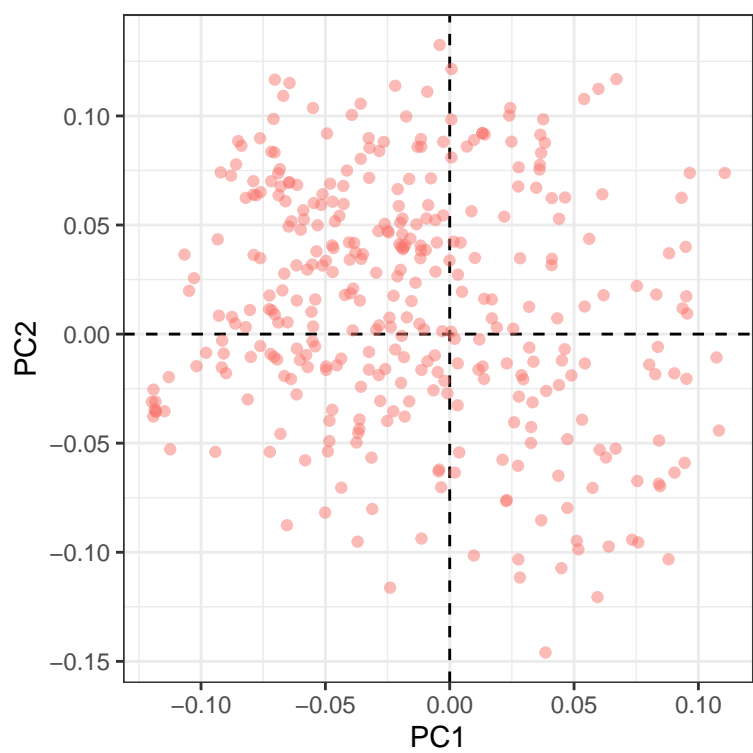

Supplement: Supplemental Information 2 [file peerj-11-15112-s002.zip › peerj-75361-Raw_data_result/Raw data/Result-X101SC21103966-Z01-J001-B1-42/3.MetDiffScreening/H.vs.NH/H.vs.NH_pos_PLSDA-loading.pdf]

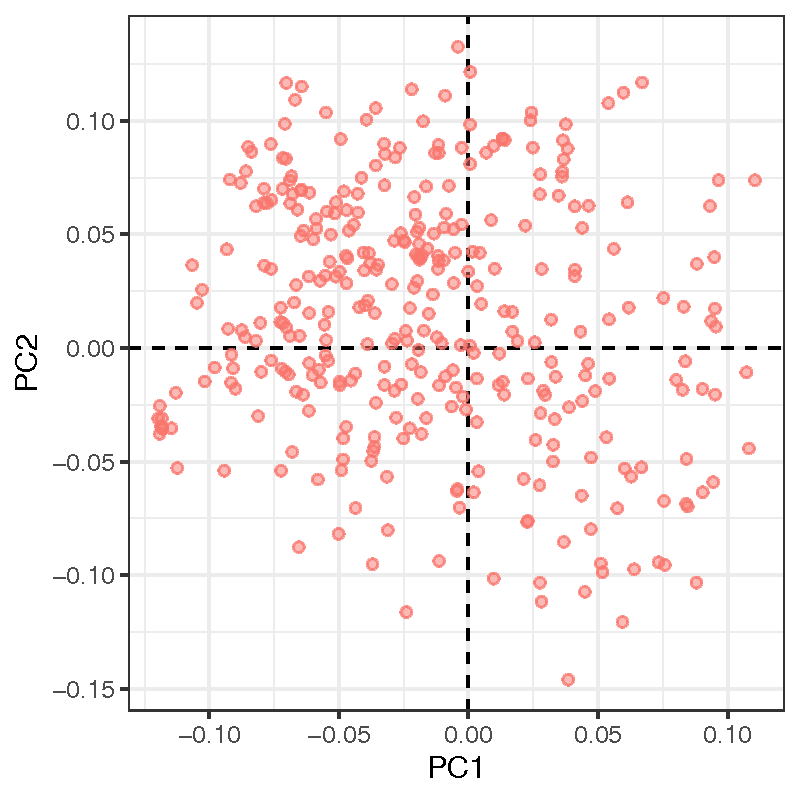

Supplement: Supplemental Information 2 [file peerj-11-15112-s002.zip › peerj-75361-Raw_data_result/Raw data/Result-X101SC21103966-Z01-J001-B1-42/3.MetDiffScreening/H.vs.NH/H.vs.NH_pos_PLSDA-loading.png]

class    ● H    ● NH

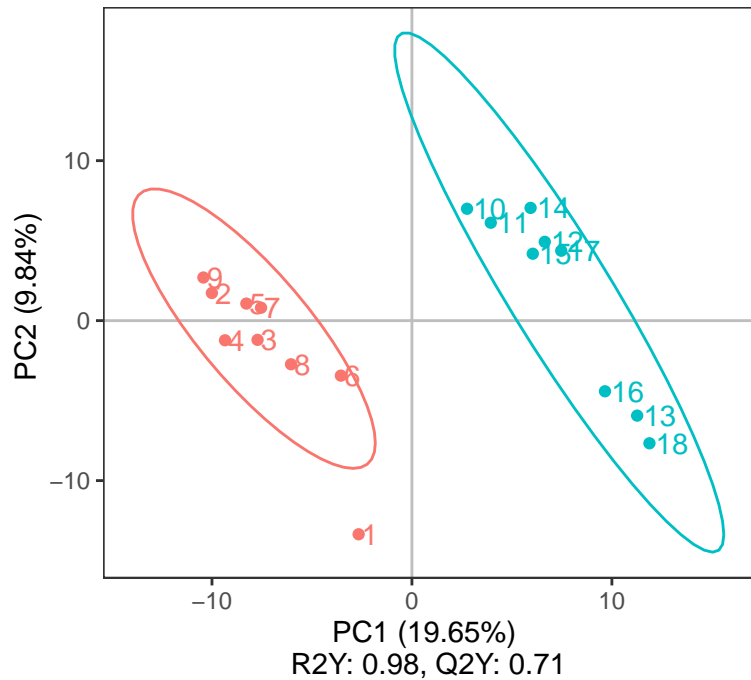

Supplement: Supplemental Information 2 [file peerj-11-15112-s002.zip › peerj-75361-Raw_data_result/Raw data/Result-X101SC21103966-Z01-J001-B1-42/3.MetDiffScreening/H.vs.NH/H.vs.NH_pos_PLSDA-score.pdf]

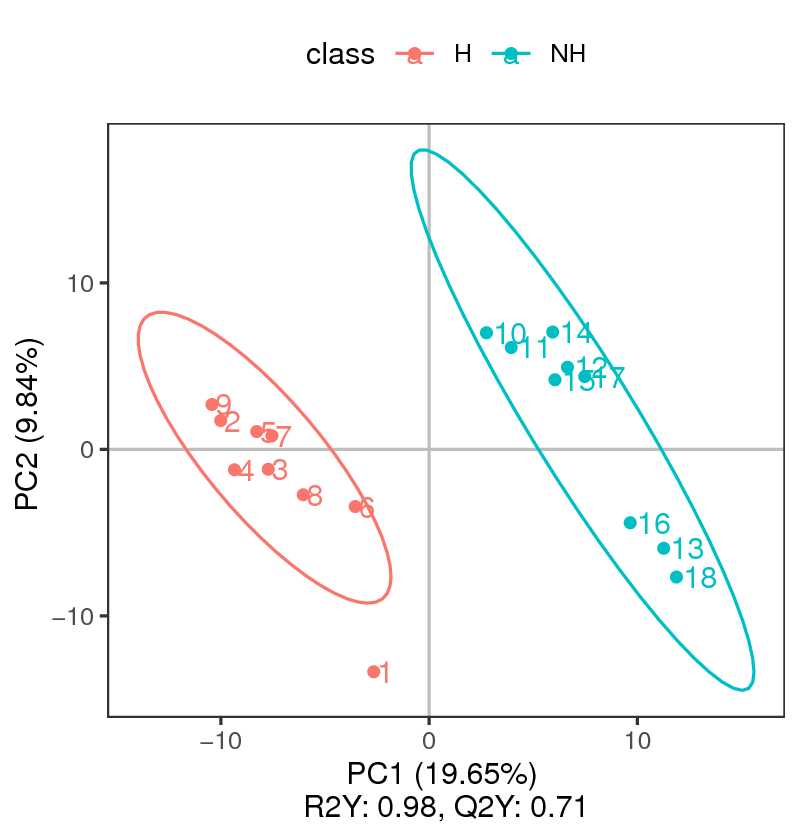

Supplement: Supplemental Information 2 [file peerj-11-15112-s002.zip › peerj-75361-Raw_data_result/Raw data/Result-X101SC21103966-Z01-J001-B1-42/3.MetDiffScreening/H.vs.NH/H.vs.NH_pos_PLSDA-score.png]

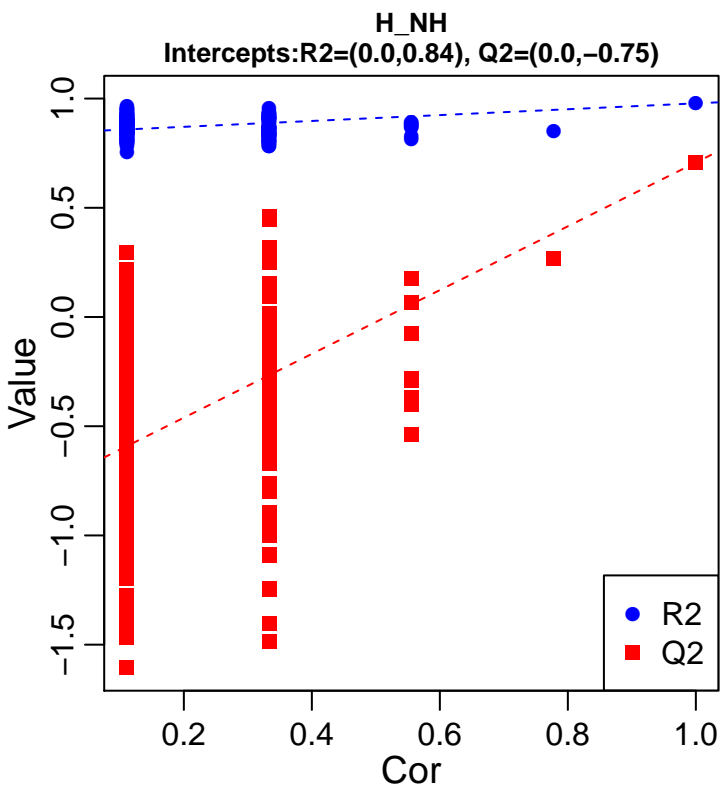

Supplement: Supplemental Information 2 [file peerj-11-15112-s002.zip › peerj-75361-Raw_data_result/Raw data/Result-X101SC21103966-Z01-J001-B1-42/3.MetDiffScreening/H.vs.NH/H.vs.NH_pos_PLSDA-valid.pdf]

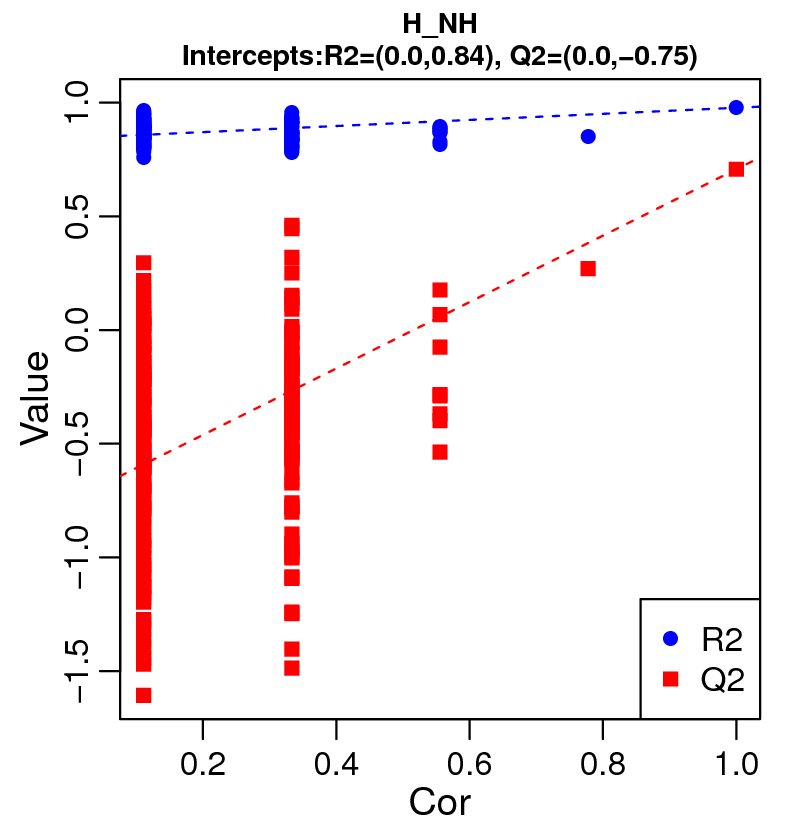

Supplement: Supplemental Information 2 [file peerj-11-15112-s002.zip › peerj-75361-Raw_data_result/Raw data/Result-X101SC21103966-Z01-J001-B1-42/3.MetDiffScreening/H.vs.NH/H.vs.NH_pos_PLSDA-valid.png]

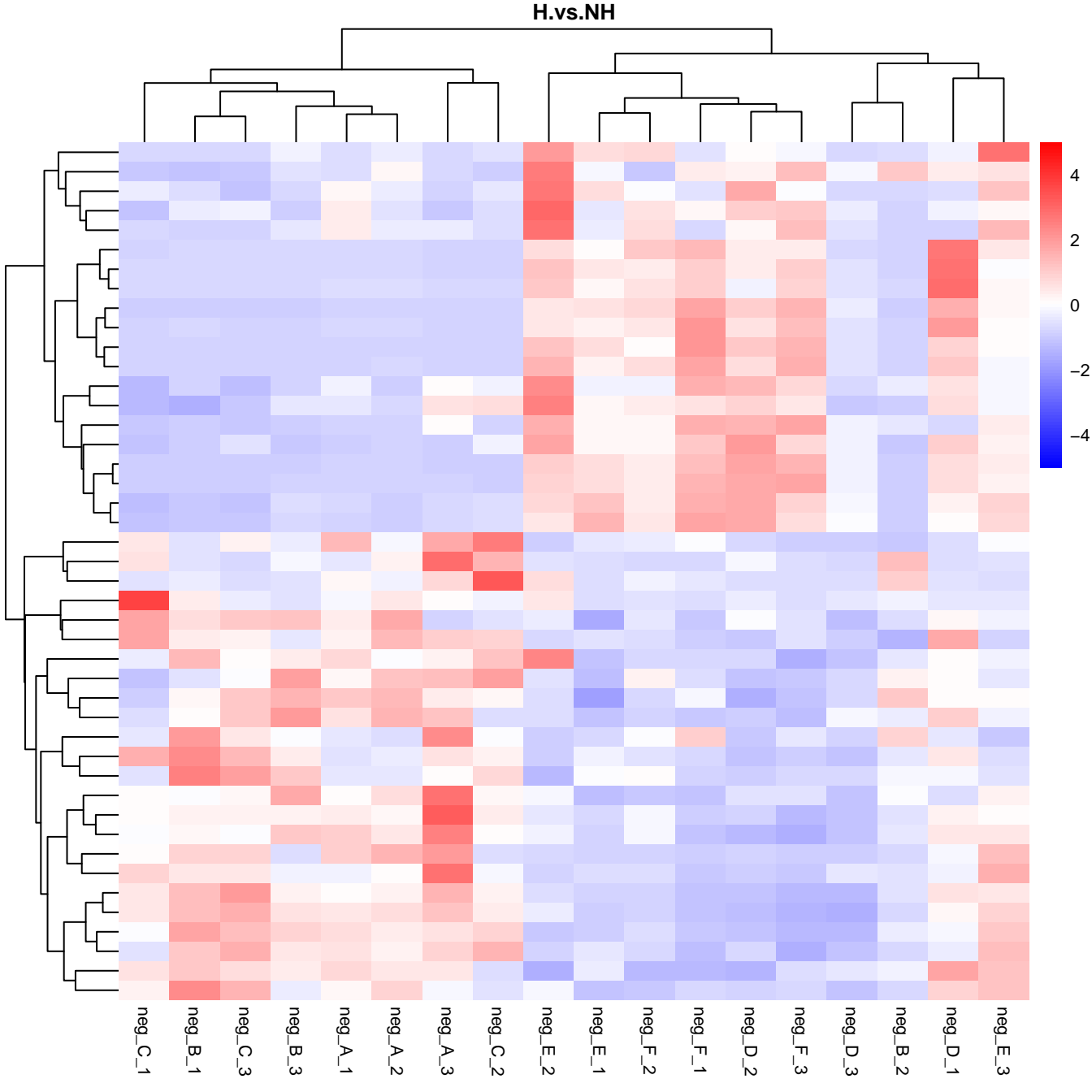

Supplement: Supplemental Information 2 [file peerj-11-15112-s002.zip › peerj-75361-Raw_data_result/Raw data/Result-X101SC21103966-Z01-J001-B1-42/4.MetDiffAnalysis/H.vs.NH/H.vs.NH_neg_cluster_heatmap.pdf]

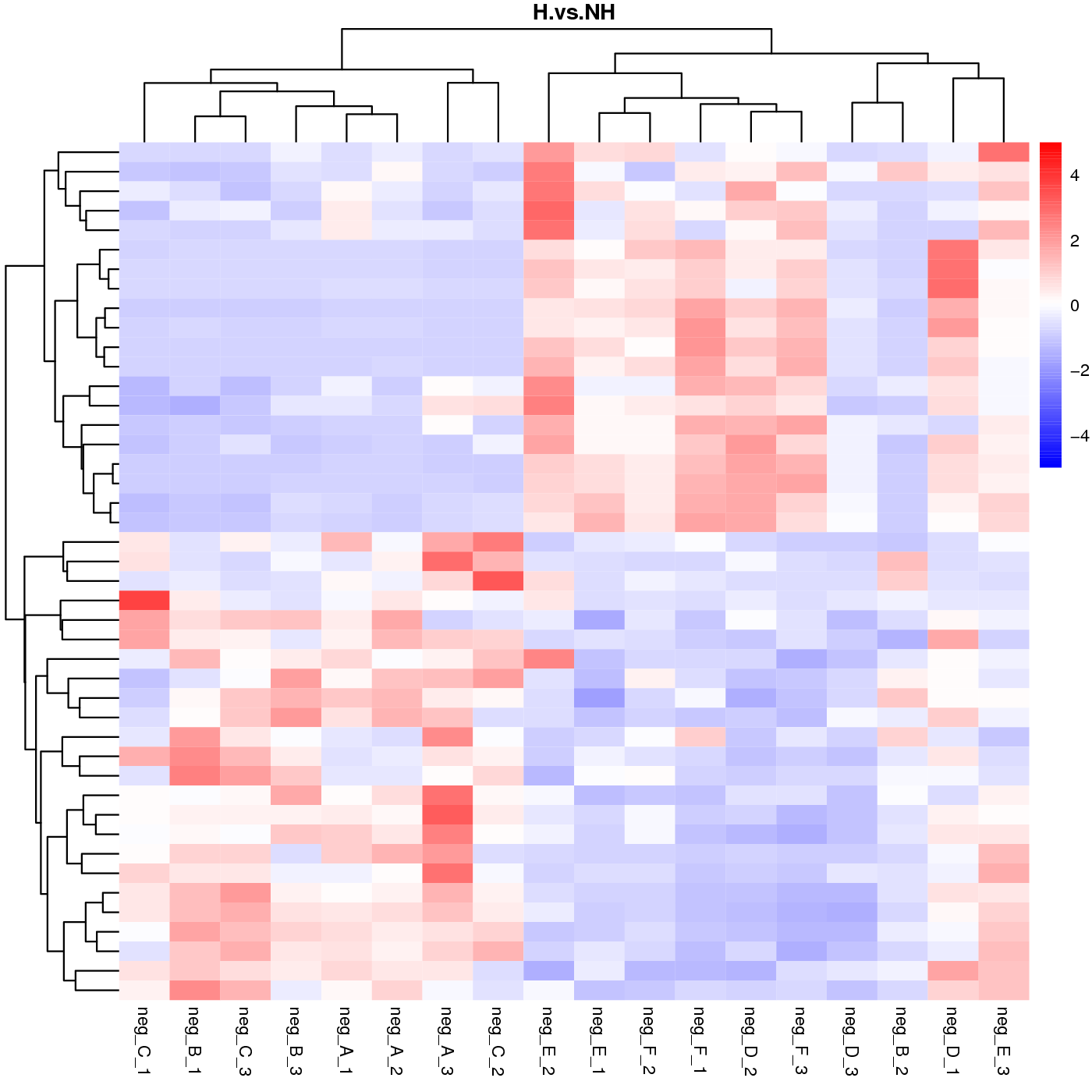

Supplement: Supplemental Information 2 [file peerj-11-15112-s002.zip › peerj-75361-Raw_data_result/Raw data/Result-X101SC21103966-Z01-J001-B1-42/4.MetDiffAnalysis/H.vs.NH/H.vs.NH_neg_cluster_heatmap.png]

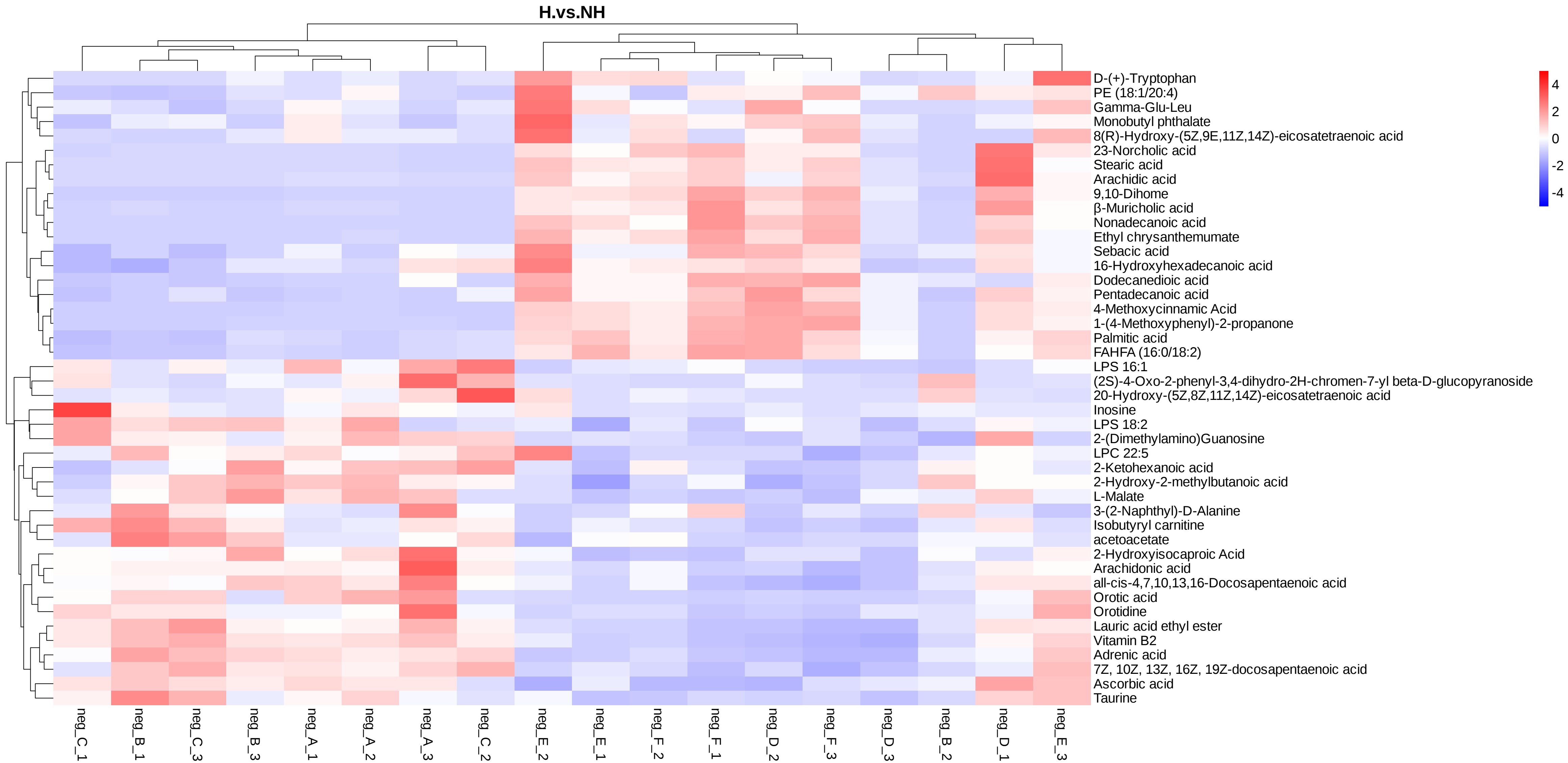

Supplement: Supplemental Information 2 [file peerj-11-15112-s002.zip › peerj-75361-Raw_data_result/Raw data/Result-X101SC21103966-Z01-J001-B1-42/4.MetDiffAnalysis/H.vs.NH/H.vs.NH_neg_cluster_heatmap_detail.pdf]

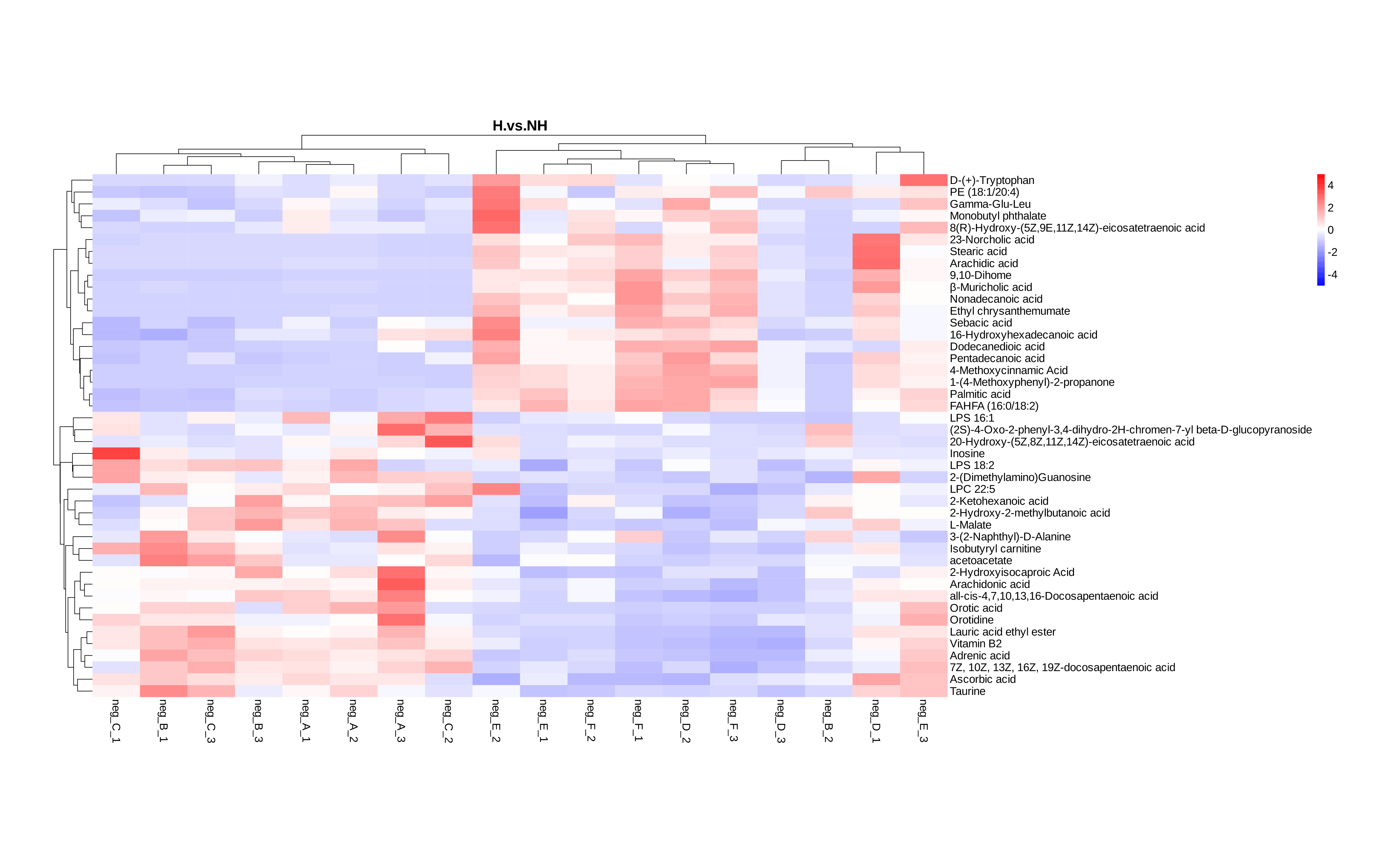

Supplement: Supplemental Information 2 [file peerj-11-15112-s002.zip › peerj-75361-Raw_data_result/Raw data/Result-X101SC21103966-Z01-J001-B1-42/4.MetDiffAnalysis/H.vs.NH/H.vs.NH_neg_cluster_heatmap_detail.png]

## H.vs.NH

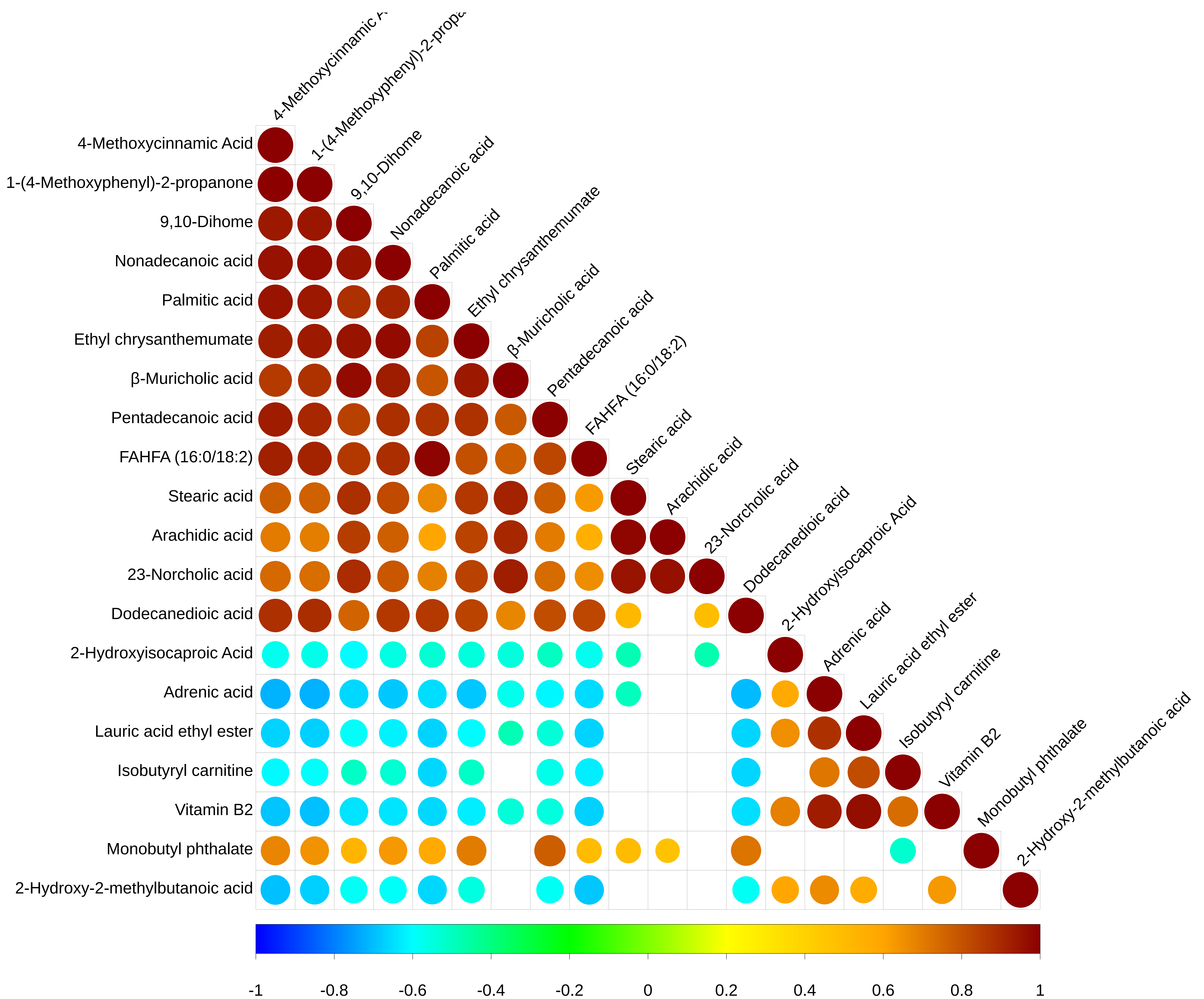

Supplement: Supplemental Information 2 [file peerj-11-15112-s002.zip › peerj-75361-Raw_data_result/Raw data/Result-X101SC21103966-Z01-J001-B1-42/4.MetDiffAnalysis/H.vs.NH/H.vs.NH_neg_corr.pdf]

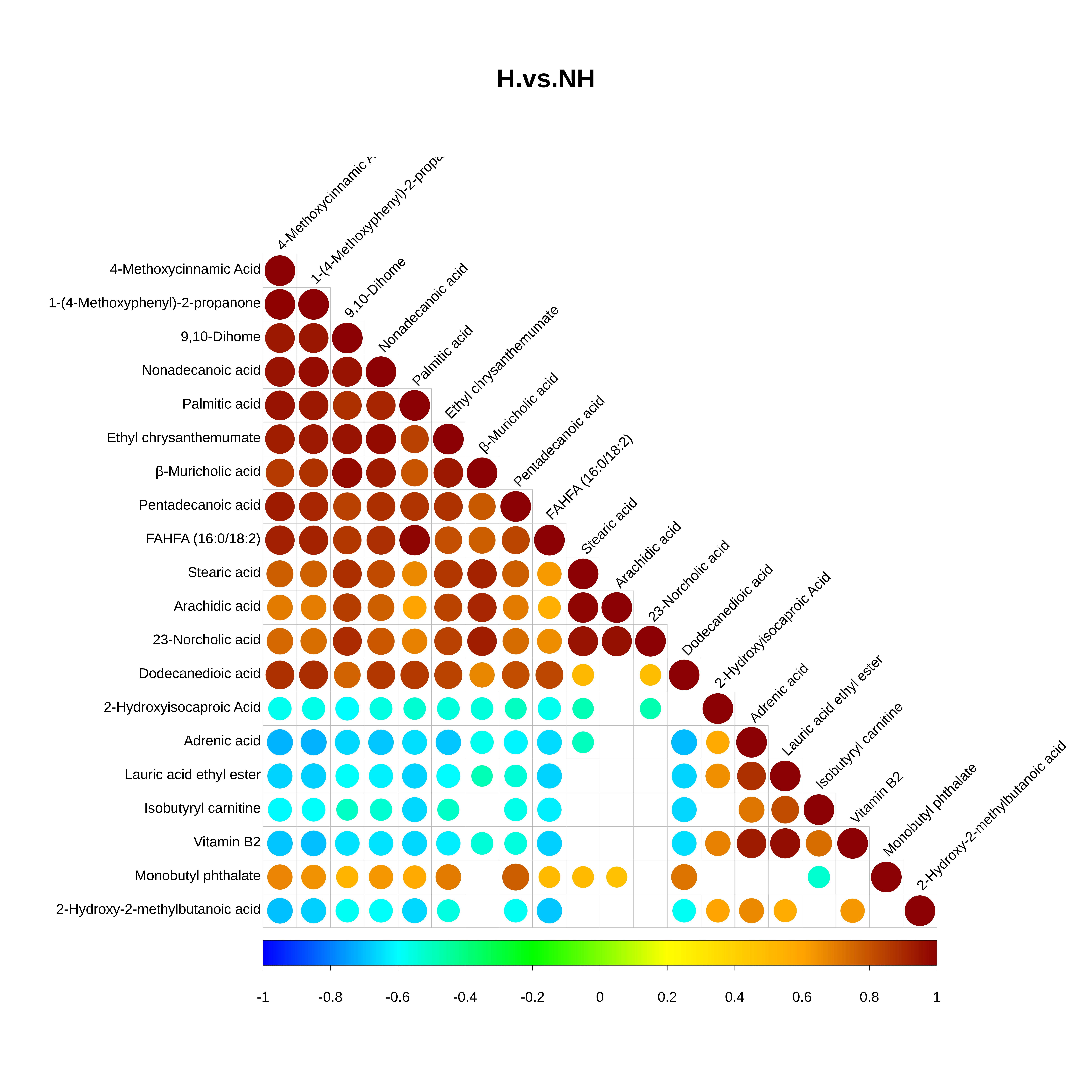

Supplement: Supplemental Information 2 [file peerj-11-15112-s002.zip › peerj-75361-Raw_data_result/Raw data/Result-X101SC21103966-Z01-J001-B1-42/4.MetDiffAnalysis/H.vs.NH/H.vs.NH_neg_corr.png]

## H.vs.NH

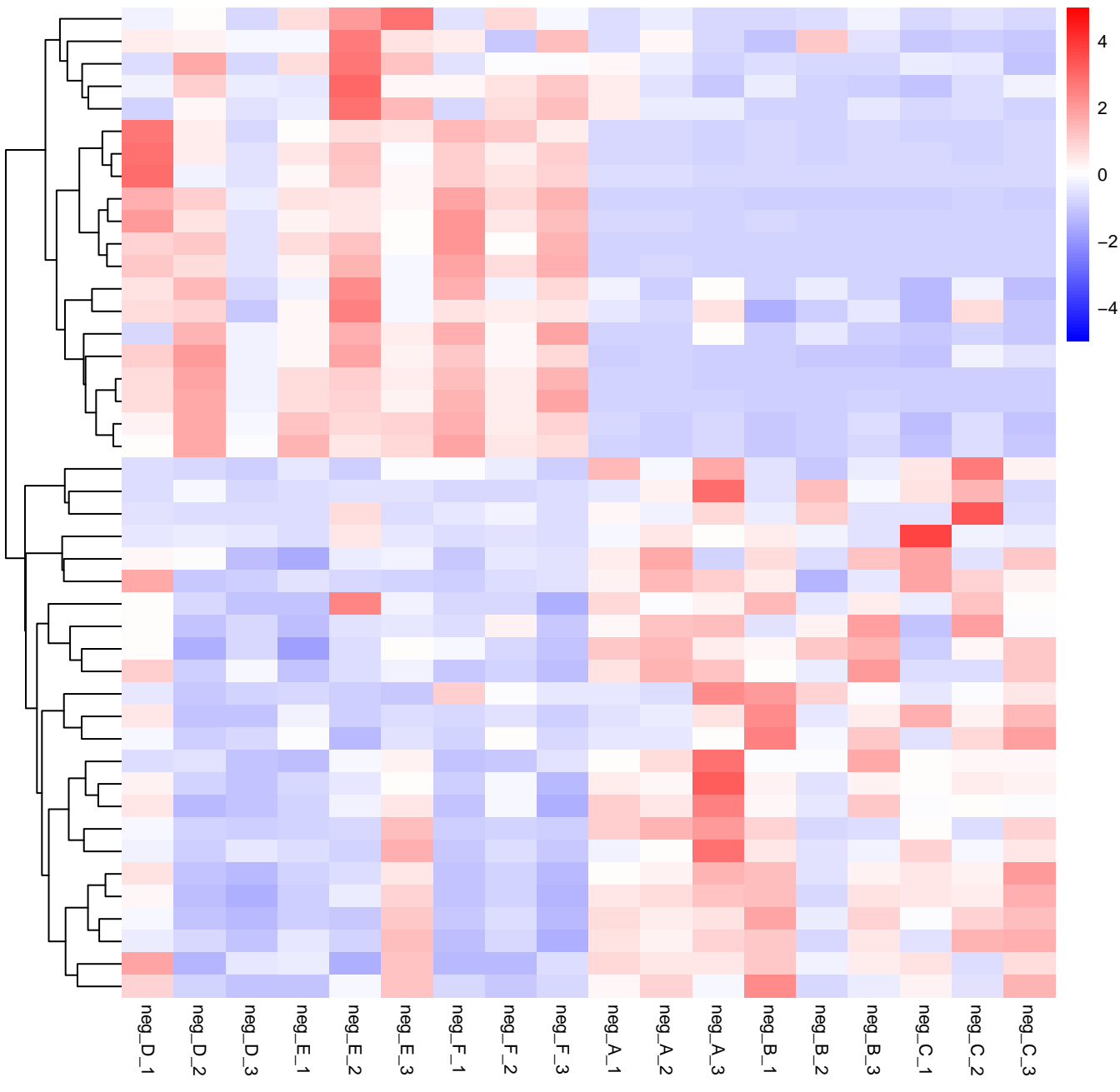

Supplement: Supplemental Information 2 [file peerj-11-15112-s002.zip › peerj-75361-Raw_data_result/Raw data/Result-X101SC21103966-Z01-J001-B1-42/4.MetDiffAnalysis/H.vs.NH/H.vs.NH_neg_heatmap.pdf]

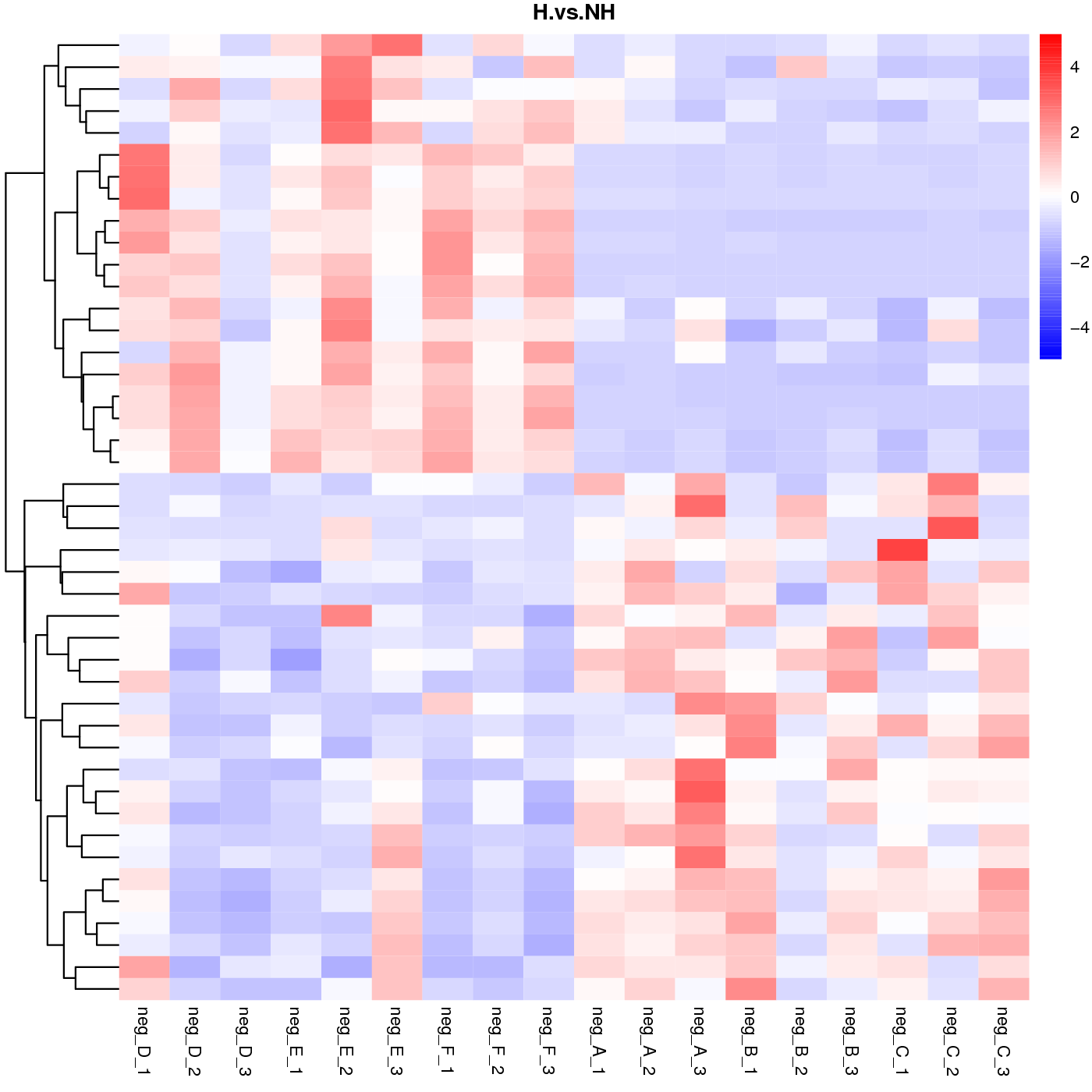

Supplement: Supplemental Information 2 [file peerj-11-15112-s002.zip › peerj-75361-Raw_data_result/Raw data/Result-X101SC21103966-Z01-J001-B1-42/4.MetDiffAnalysis/H.vs.NH/H.vs.NH_neg_heatmap.png]

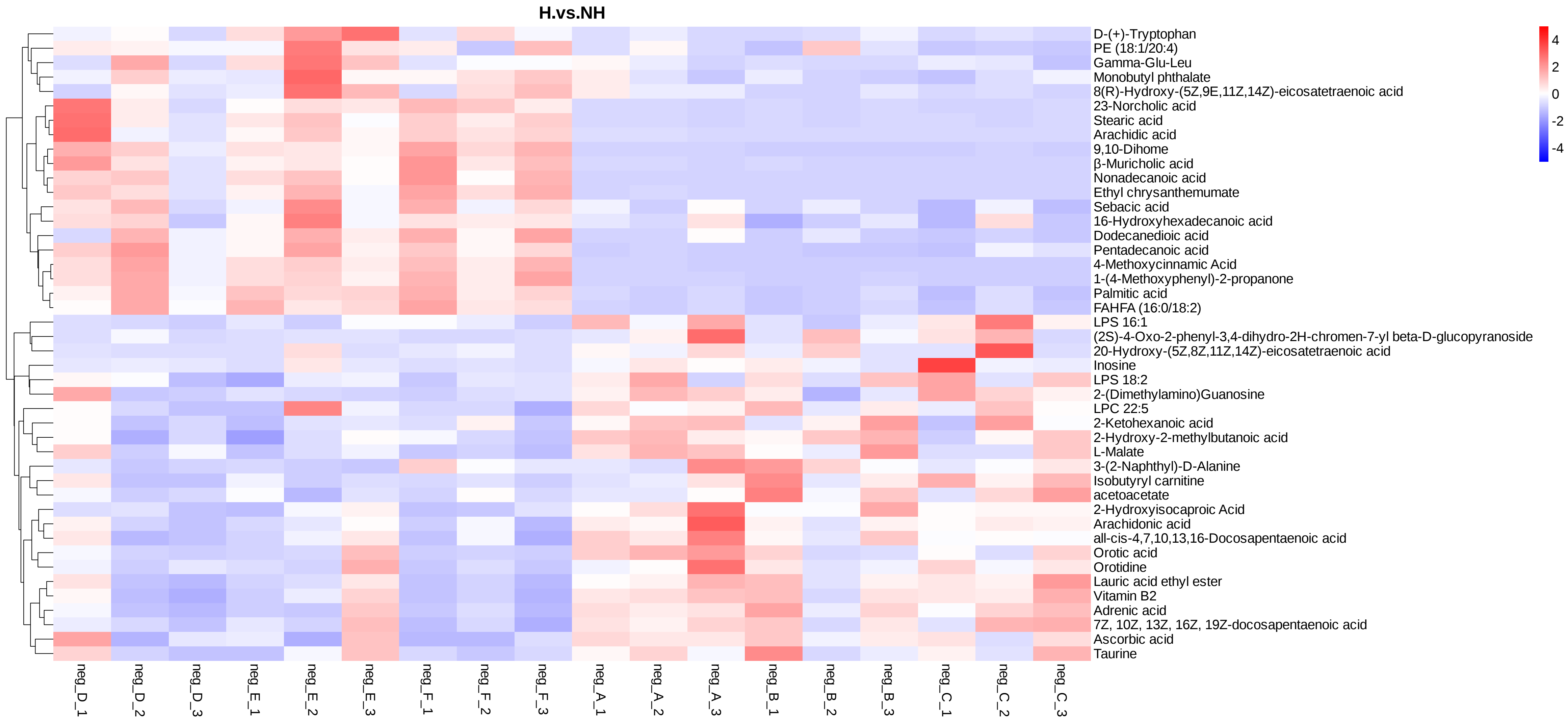

Supplement: Supplemental Information 2 [file peerj-11-15112-s002.zip › peerj-75361-Raw_data_result/Raw data/Result-X101SC21103966-Z01-J001-B1-42/4.MetDiffAnalysis/H.vs.NH/H.vs.NH_neg_heatmap_detail.pdf]

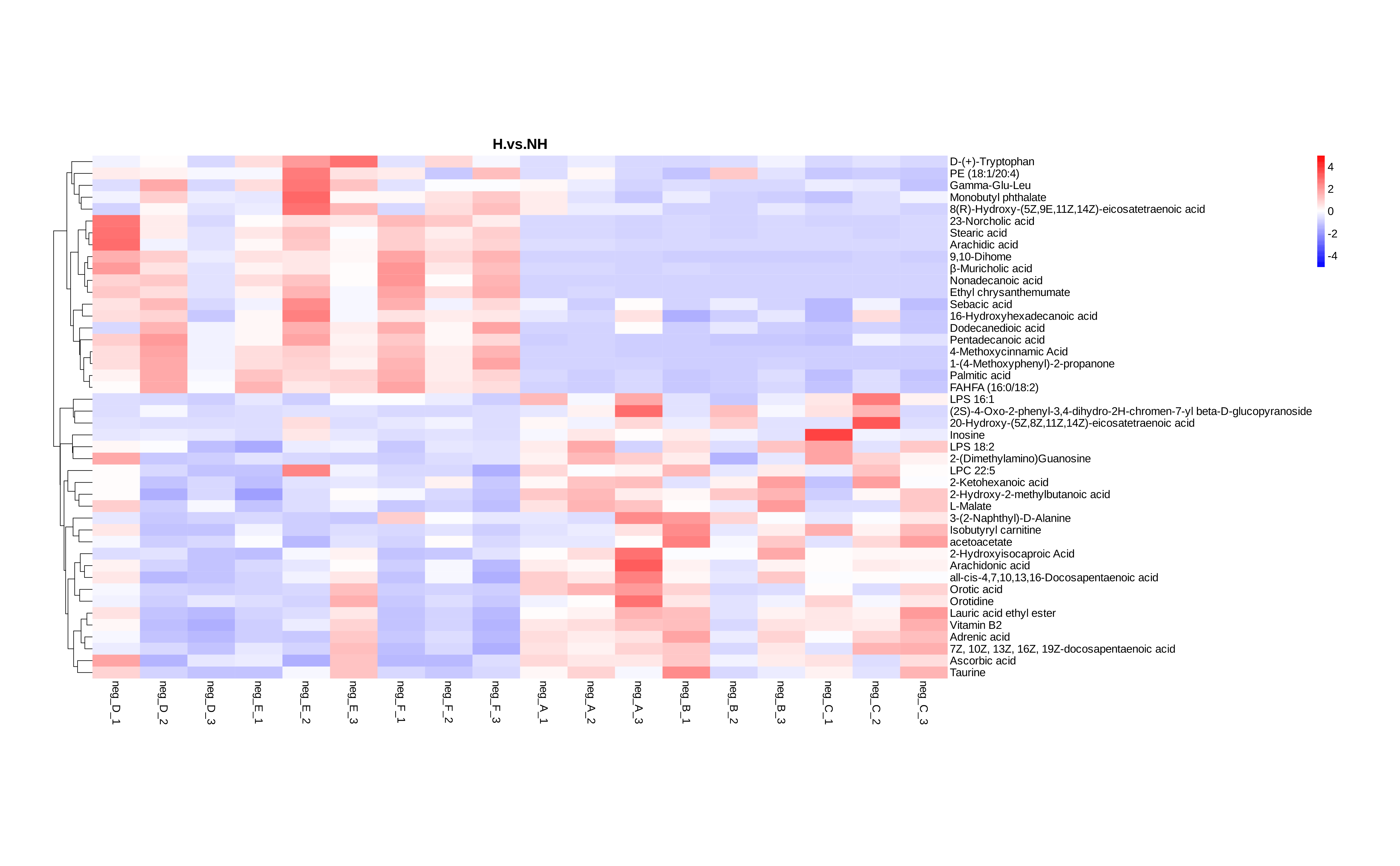

Supplement: Supplemental Information 2 [file peerj-11-15112-s002.zip › peerj-75361-Raw_data_result/Raw data/Result-X101SC21103966-Z01-J001-B1-42/4.MetDiffAnalysis/H.vs.NH/H.vs.NH_neg_heatmap_detail.png]

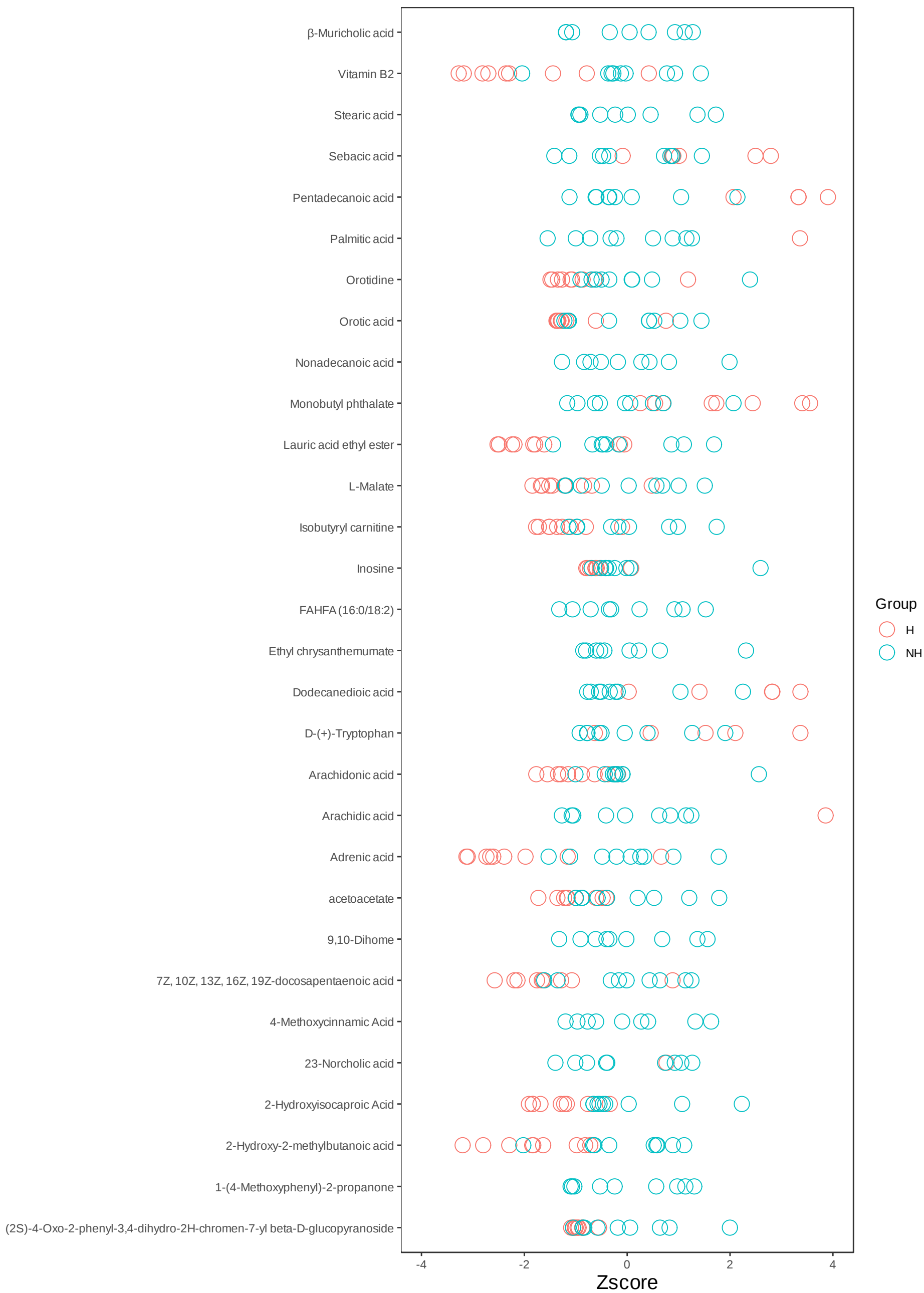

Supplement: Supplemental Information 2 [file peerj-11-15112-s002.zip › peerj-75361-Raw_data_result/Raw data/Result-X101SC21103966-Z01-J001-B1-42/4.MetDiffAnalysis/H.vs.NH/H.vs.NH_neg_zscore.pdf]

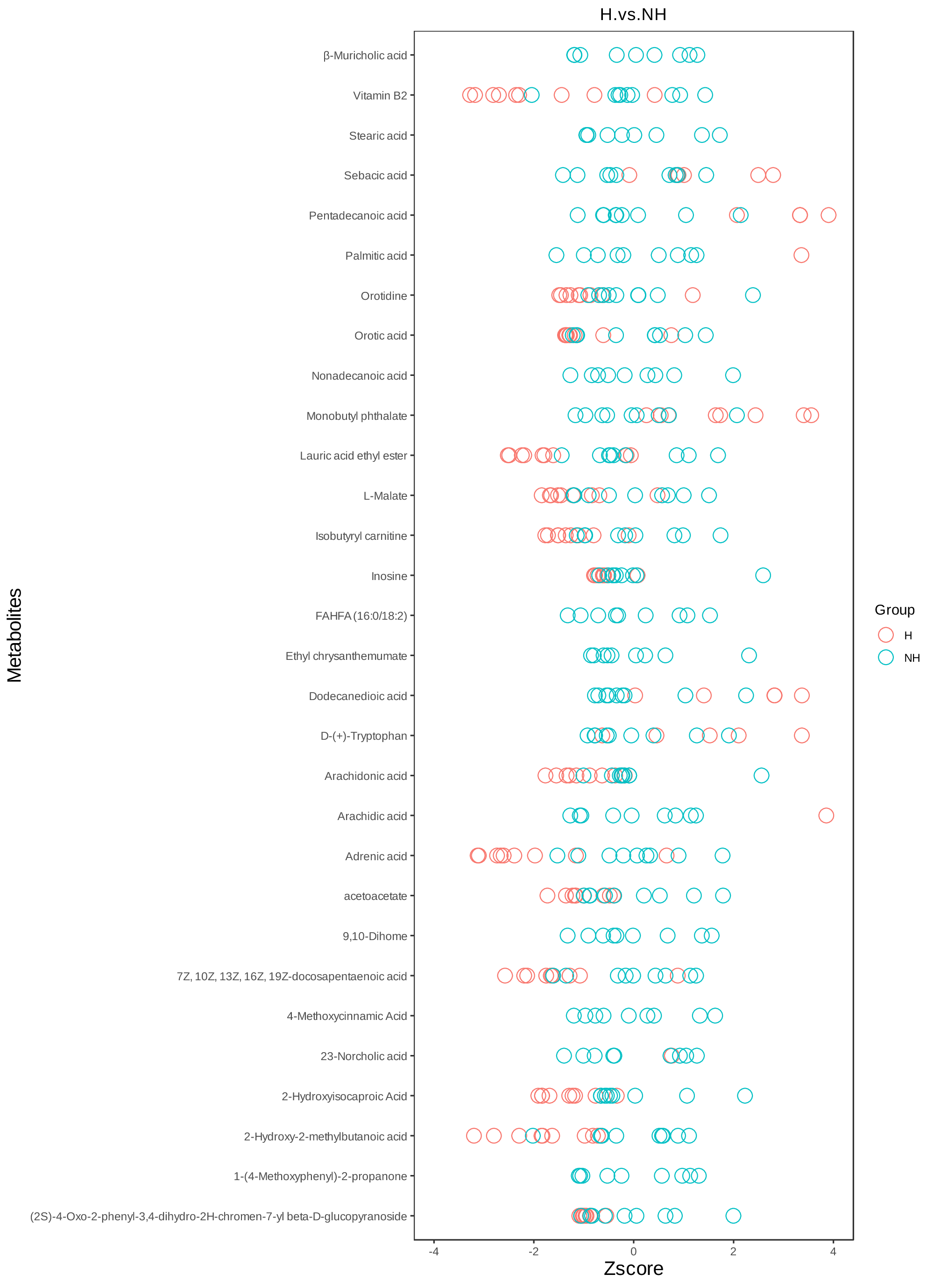

Supplement: Supplemental Information 2 [file peerj-11-15112-s002.zip › peerj-75361-Raw_data_result/Raw data/Result-X101SC21103966-Z01-J001-B1-42/4.MetDiffAnalysis/H.vs.NH/H.vs.NH_neg_zscore.png]

H.vs.NH

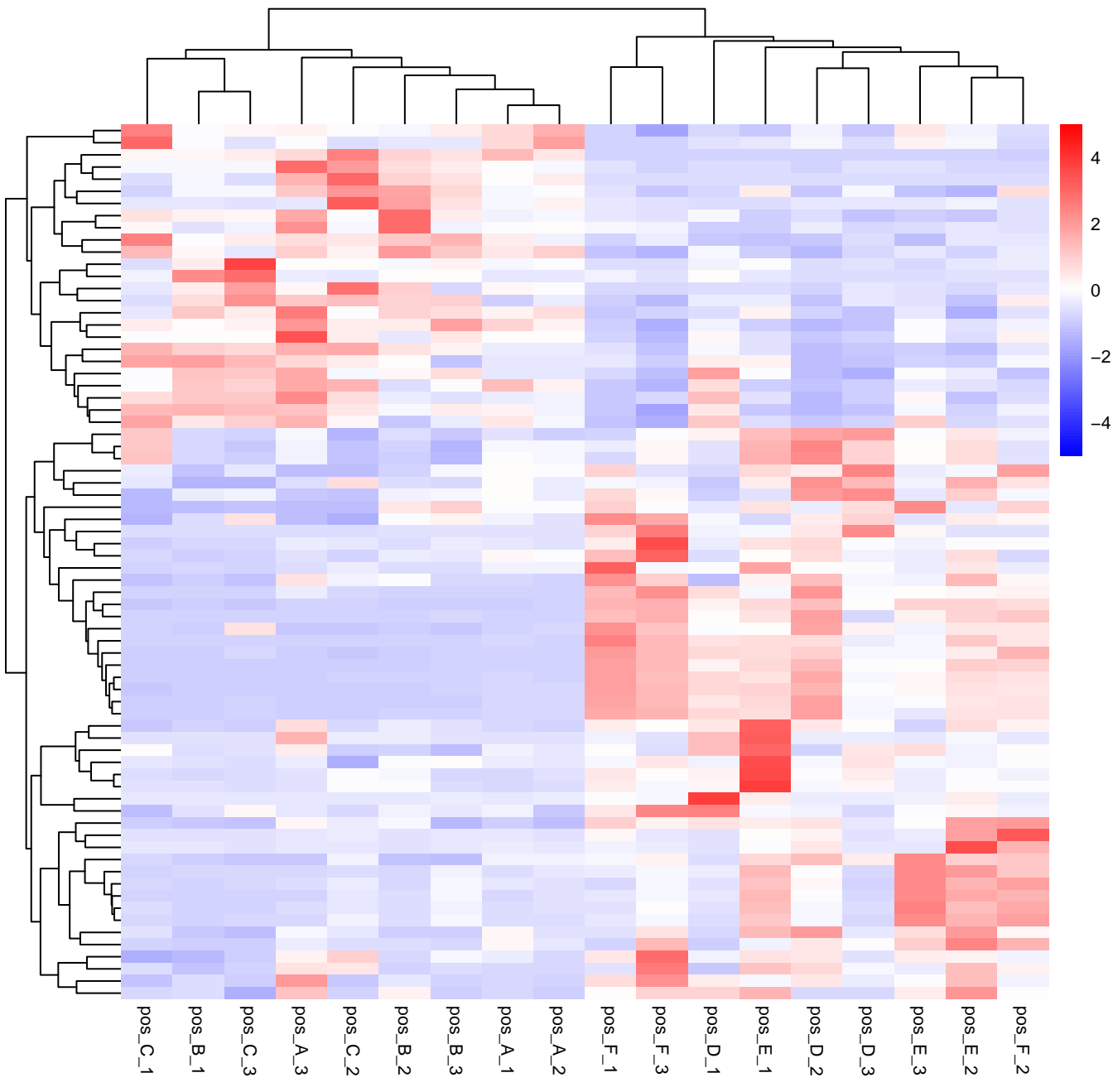

Supplement: Supplemental Information 2 [file peerj-11-15112-s002.zip › peerj-75361-Raw_data_result/Raw data/Result-X101SC21103966-Z01-J001-B1-42/4.MetDiffAnalysis/H.vs.NH/H.vs.NH_pos_cluster_heatmap.pdf]

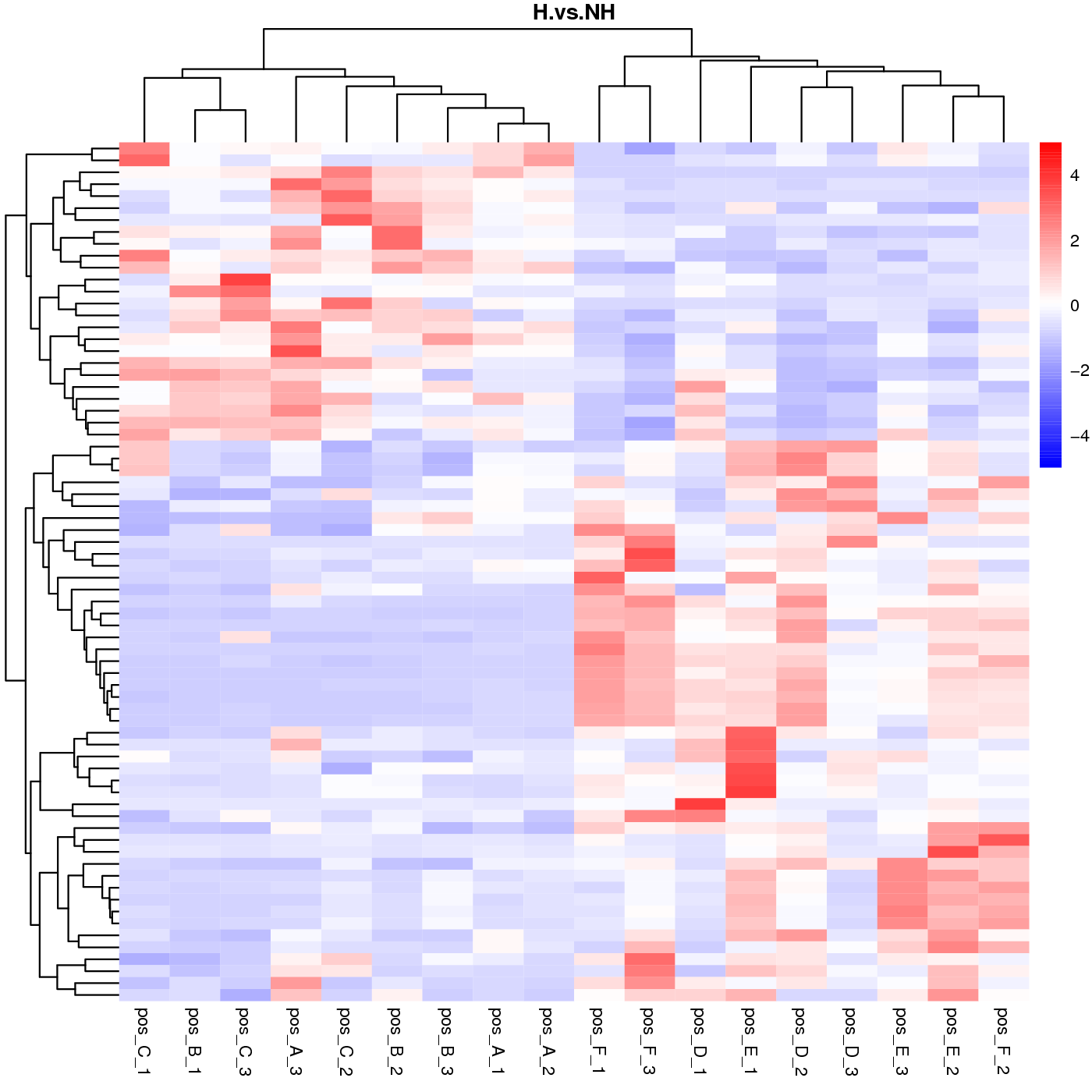

Supplement: Supplemental Information 2 [file peerj-11-15112-s002.zip › peerj-75361-Raw_data_result/Raw data/Result-X101SC21103966-Z01-J001-B1-42/4.MetDiffAnalysis/H.vs.NH/H.vs.NH_pos_cluster_heatmap.png]

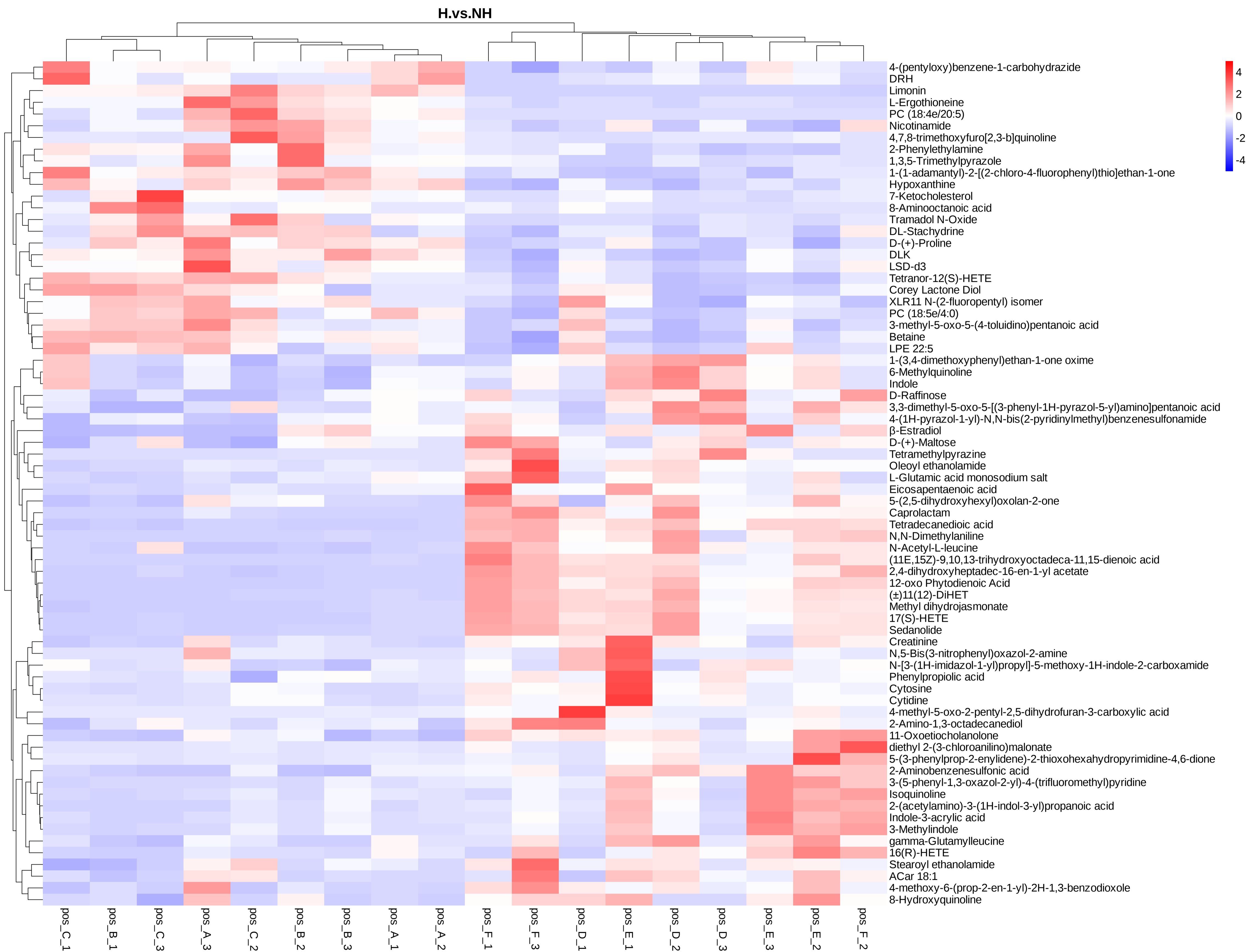

Supplement: Supplemental Information 2 [file peerj-11-15112-s002.zip › peerj-75361-Raw_data_result/Raw data/Result-X101SC21103966-Z01-J001-B1-42/4.MetDiffAnalysis/H.vs.NH/H.vs.NH_pos_cluster_heatmap_detail.pdf]

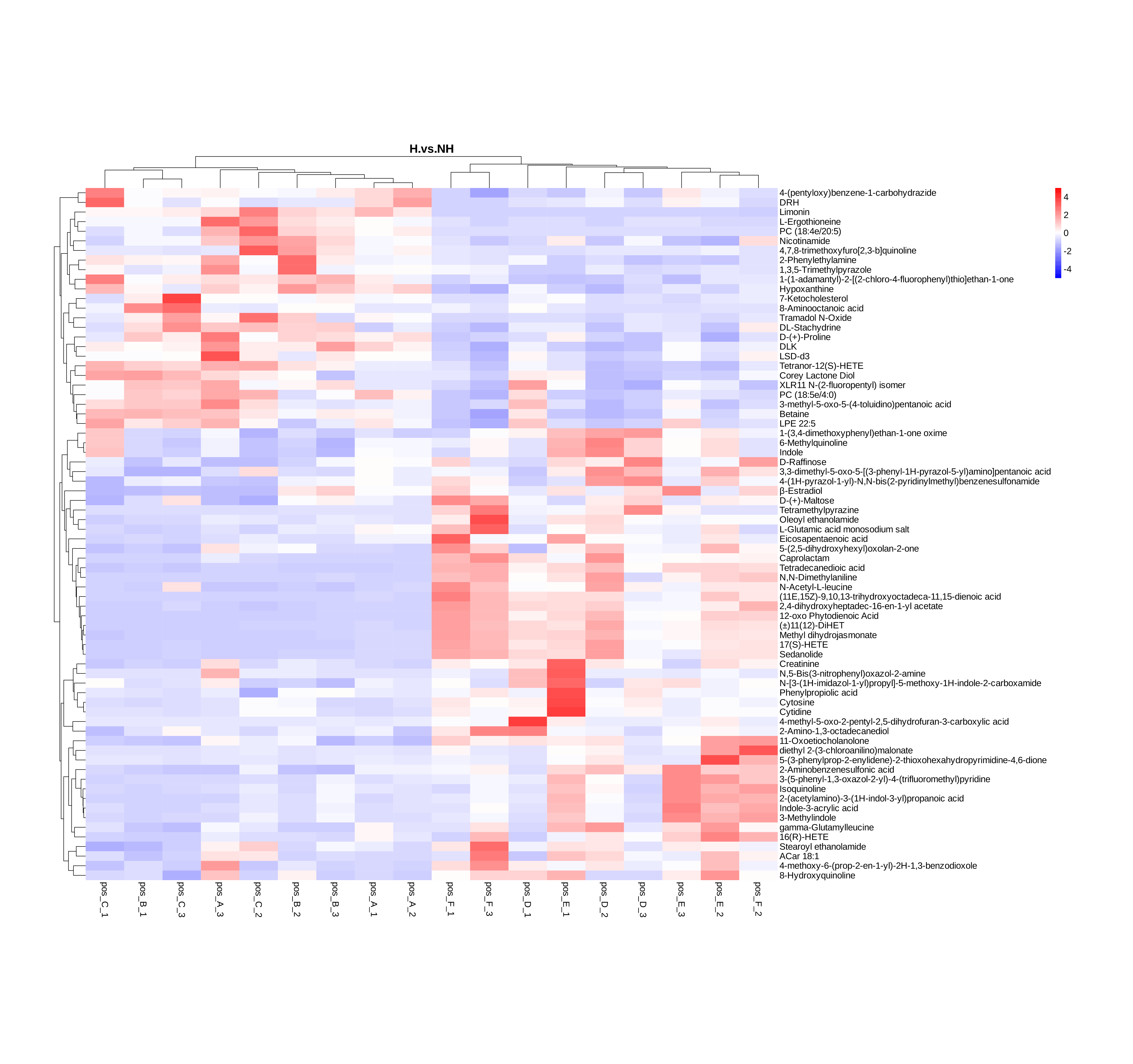

Supplement: Supplemental Information 2 [file peerj-11-15112-s002.zip › peerj-75361-Raw_data_result/Raw data/Result-X101SC21103966-Z01-J001-B1-42/4.MetDiffAnalysis/H.vs.NH/H.vs.NH_pos_cluster_heatmap_detail.png]

# H.vs.NH

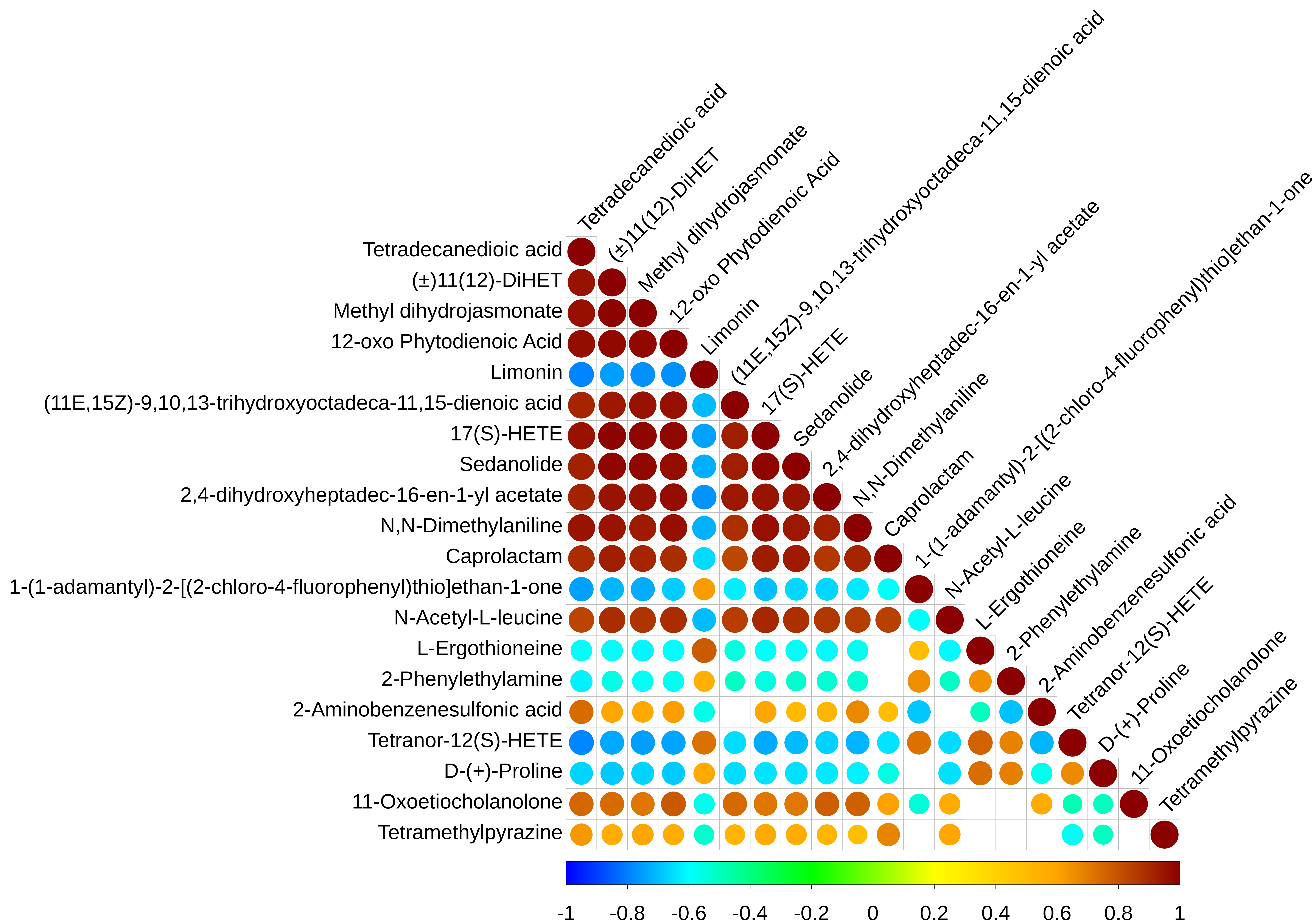

Supplement: Supplemental Information 2 [file peerj-11-15112-s002.zip › peerj-75361-Raw_data_result/Raw data/Result-X101SC21103966-Z01-J001-B1-42/4.MetDiffAnalysis/H.vs.NH/H.vs.NH_pos_corr.pdf]

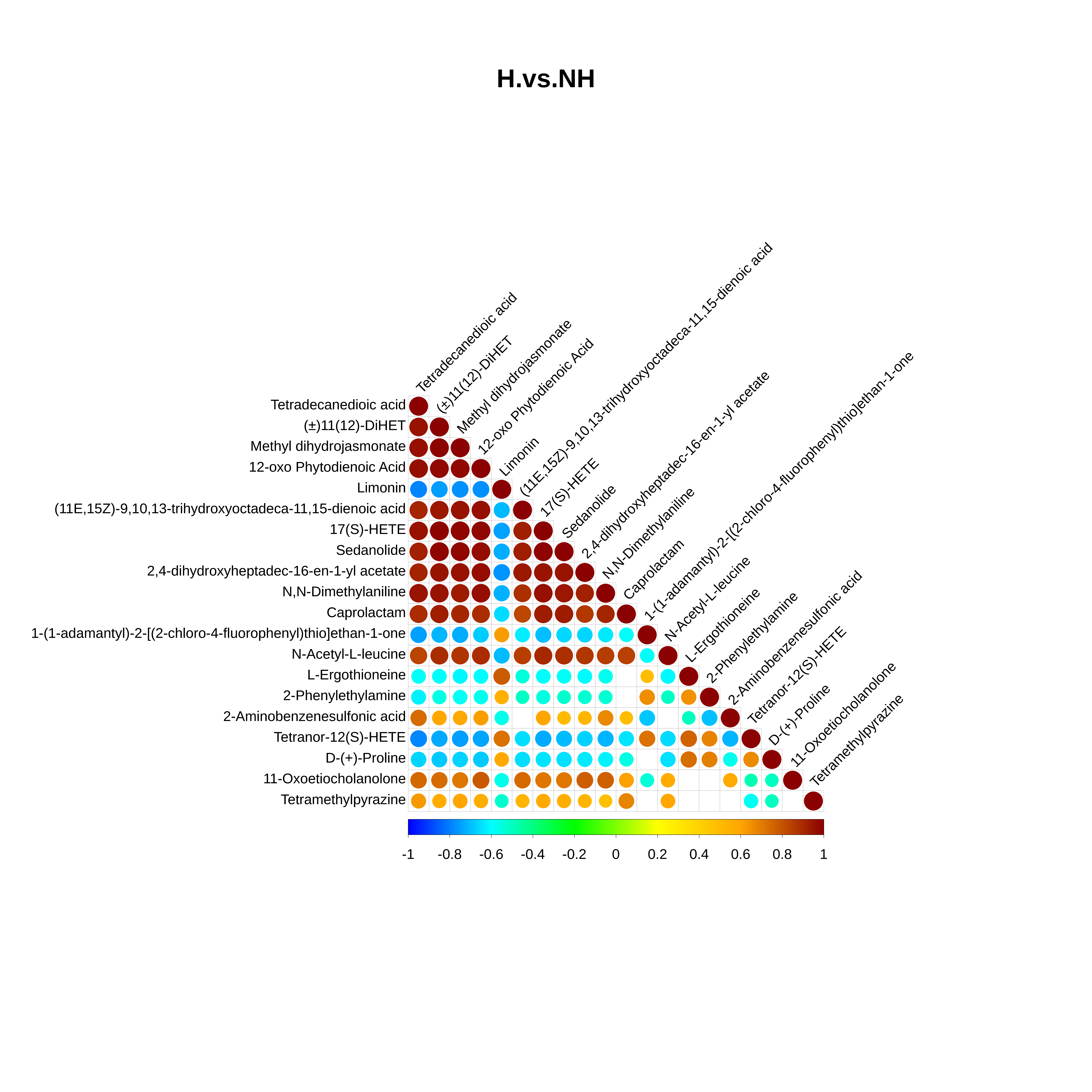

Supplement: Supplemental Information 2 [file peerj-11-15112-s002.zip › peerj-75361-Raw_data_result/Raw data/Result-X101SC21103966-Z01-J001-B1-42/4.MetDiffAnalysis/H.vs.NH/H.vs.NH_pos_corr.png]

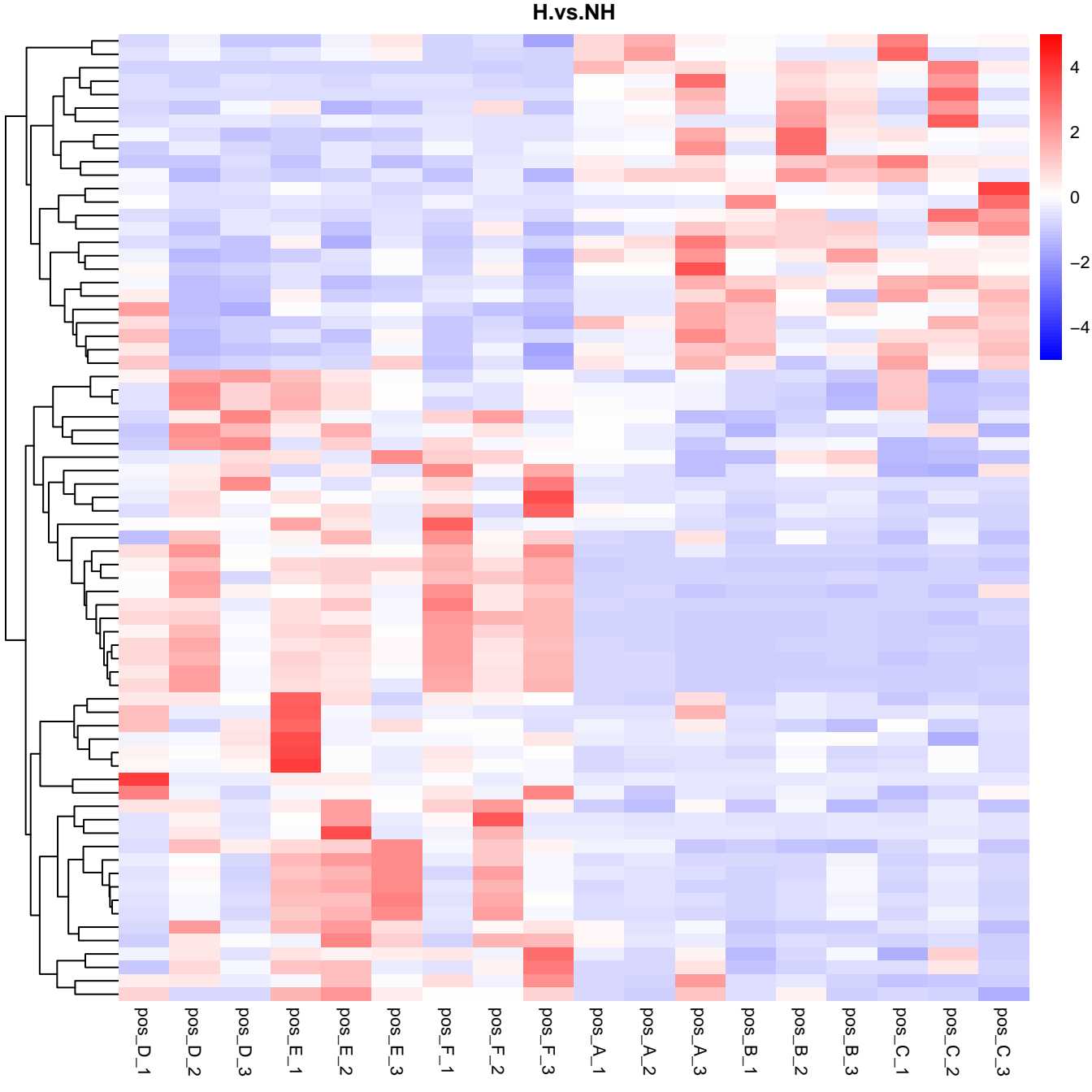

Supplement: Supplemental Information 2 [file peerj-11-15112-s002.zip › peerj-75361-Raw_data_result/Raw data/Result-X101SC21103966-Z01-J001-B1-42/4.MetDiffAnalysis/H.vs.NH/H.vs.NH_pos_heatmap.pdf]

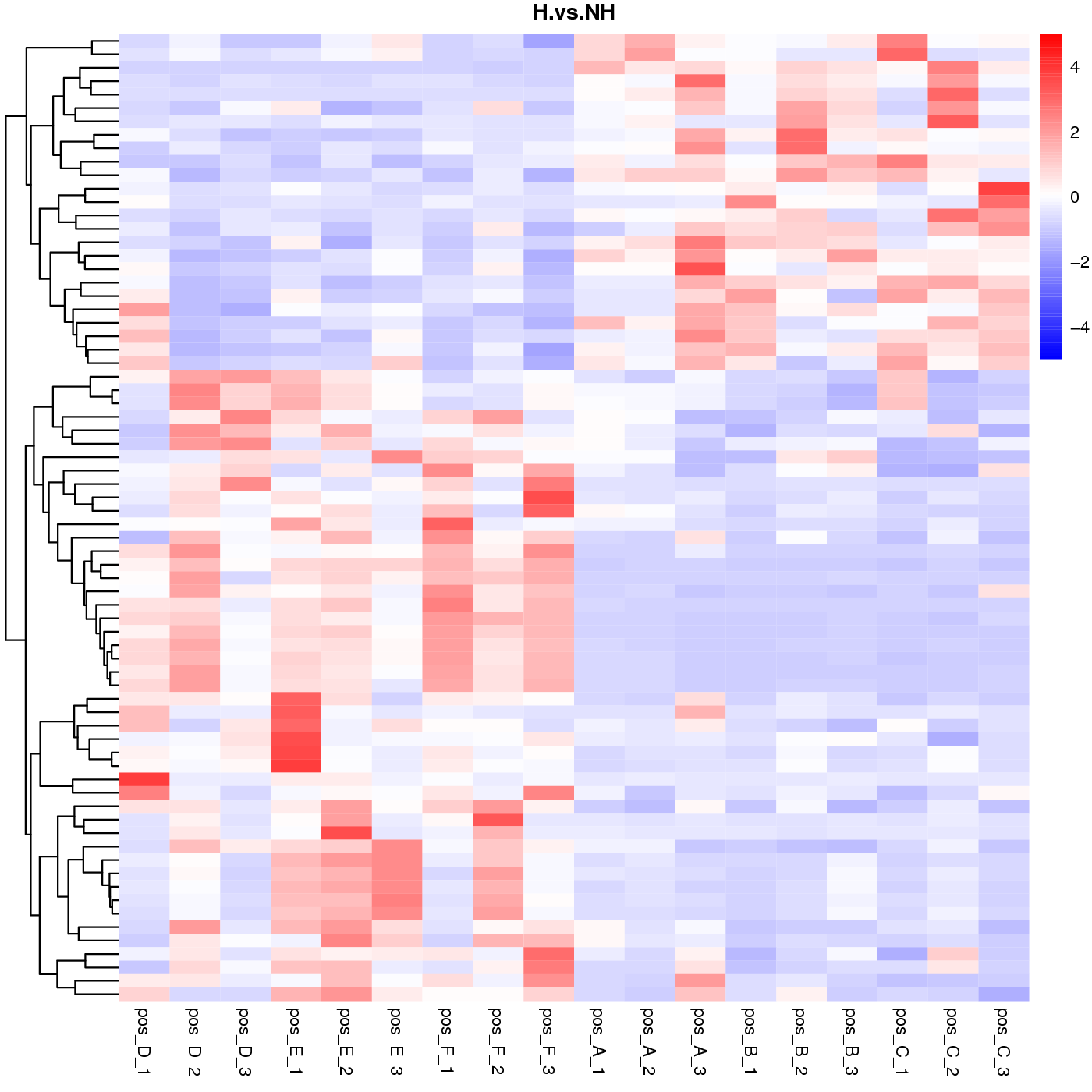

Supplement: Supplemental Information 2 [file peerj-11-15112-s002.zip › peerj-75361-Raw_data_result/Raw data/Result-X101SC21103966-Z01-J001-B1-42/4.MetDiffAnalysis/H.vs.NH/H.vs.NH_pos_heatmap.png]

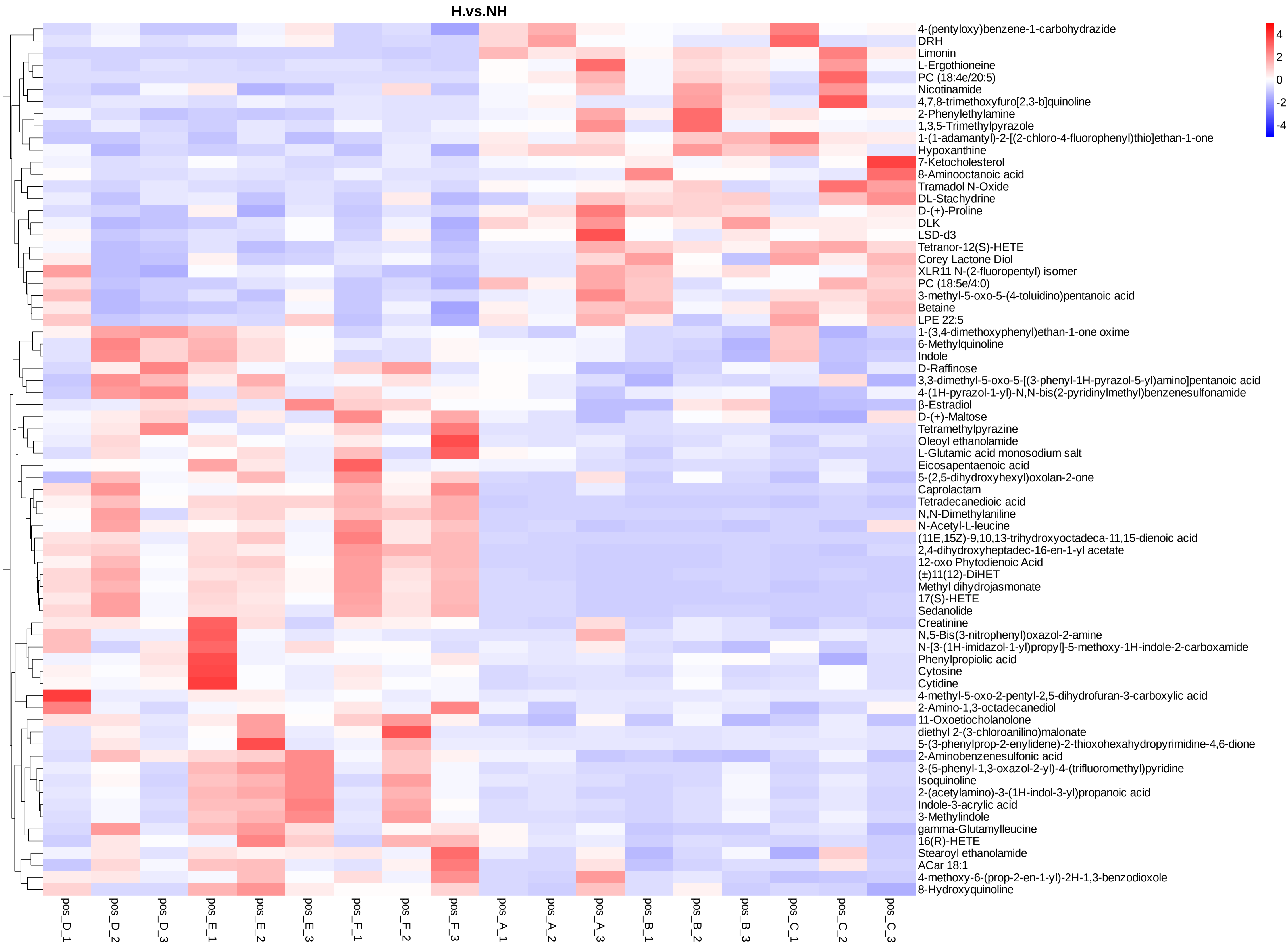

Supplement: Supplemental Information 2 [file peerj-11-15112-s002.zip › peerj-75361-Raw_data_result/Raw data/Result-X101SC21103966-Z01-J001-B1-42/4.MetDiffAnalysis/H.vs.NH/H.vs.NH_pos_heatmap_detail.pdf]

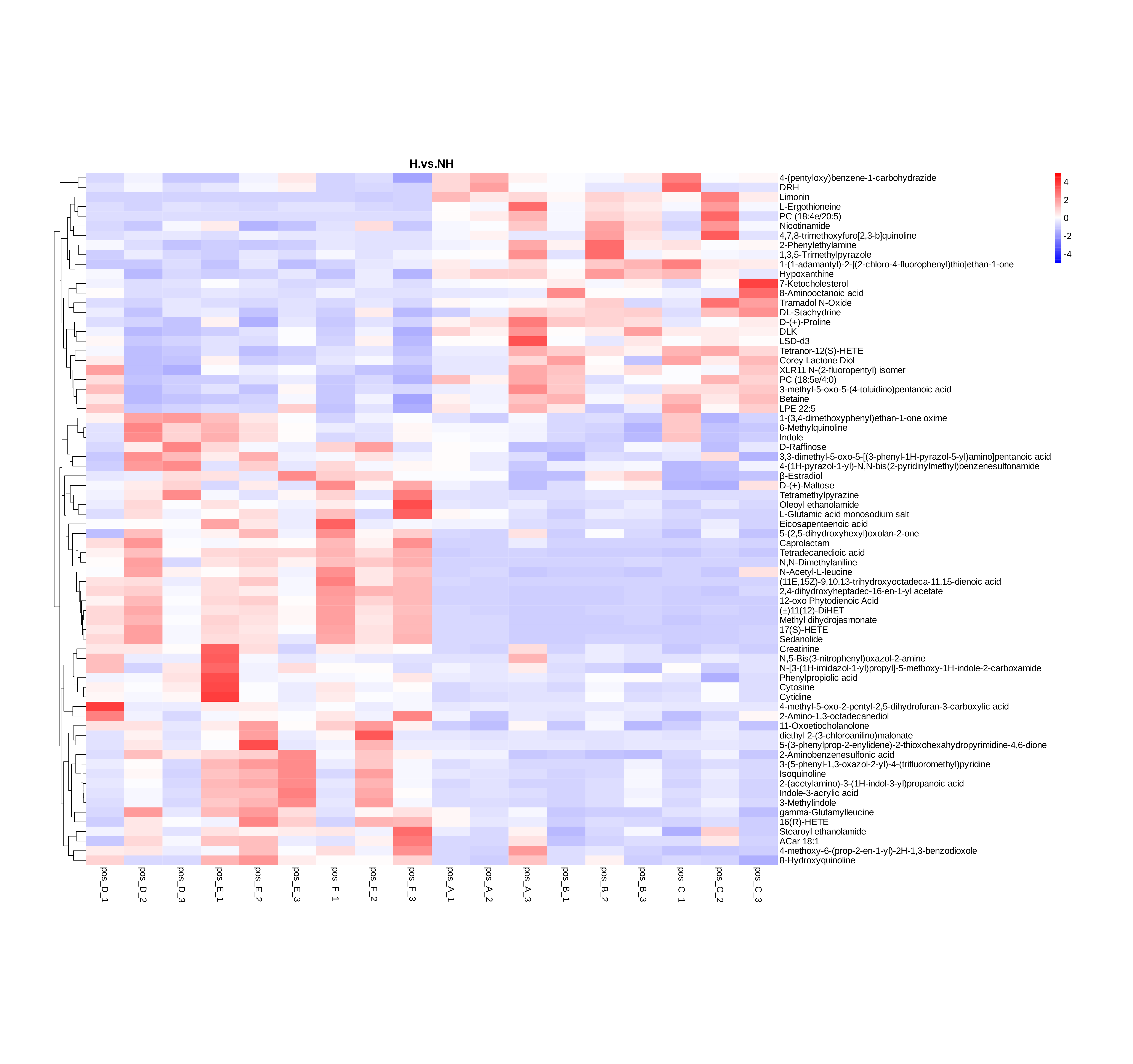

Supplement: Supplemental Information 2 [file peerj-11-15112-s002.zip › peerj-75361-Raw_data_result/Raw data/Result-X101SC21103966-Z01-J001-B1-42/4.MetDiffAnalysis/H.vs.NH/H.vs.NH_pos_heatmap_detail.png]

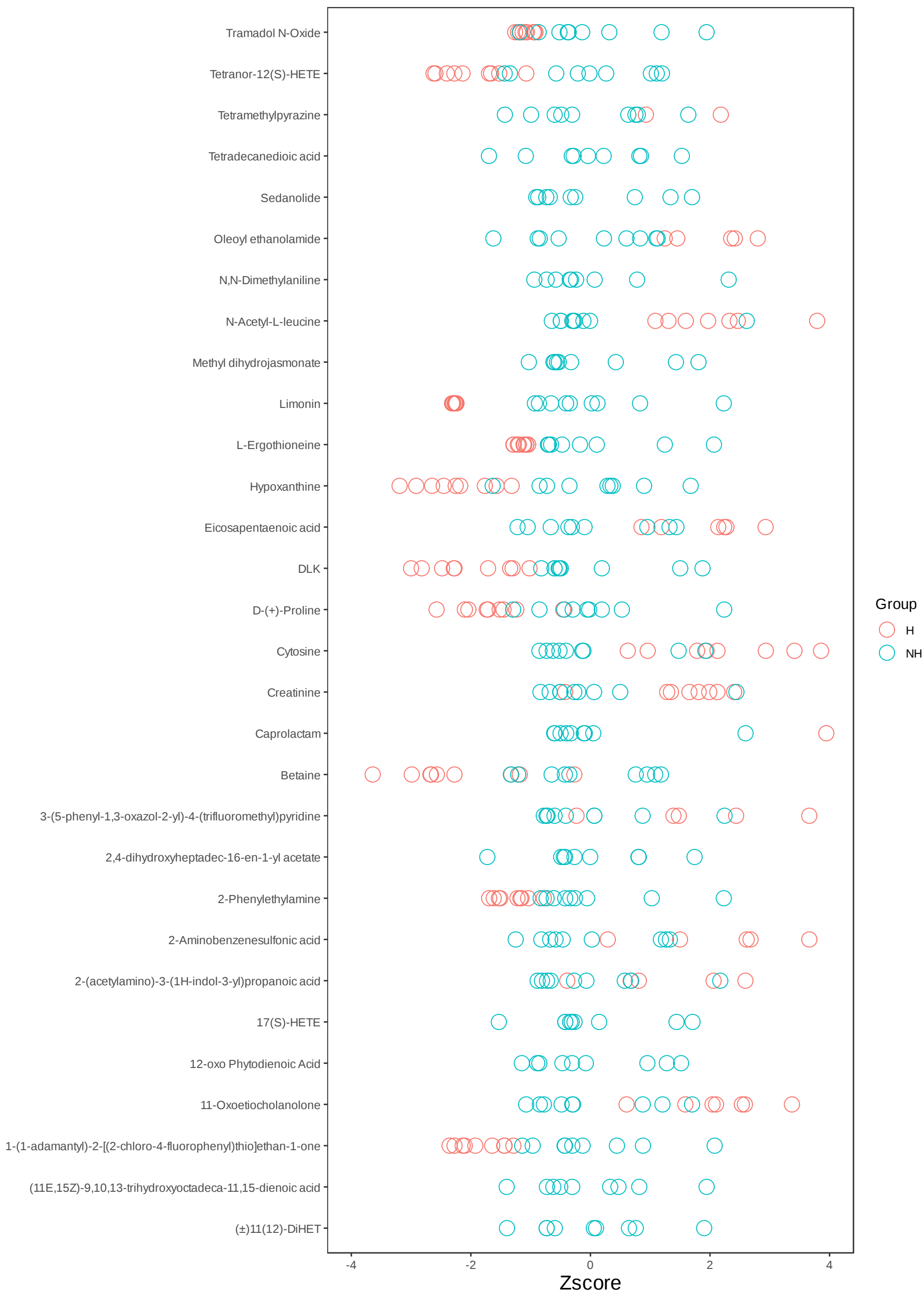

Supplement: Supplemental Information 2 [file peerj-11-15112-s002.zip › peerj-75361-Raw_data_result/Raw data/Result-X101SC21103966-Z01-J001-B1-42/4.MetDiffAnalysis/H.vs.NH/H.vs.NH_pos_zscore.pdf]

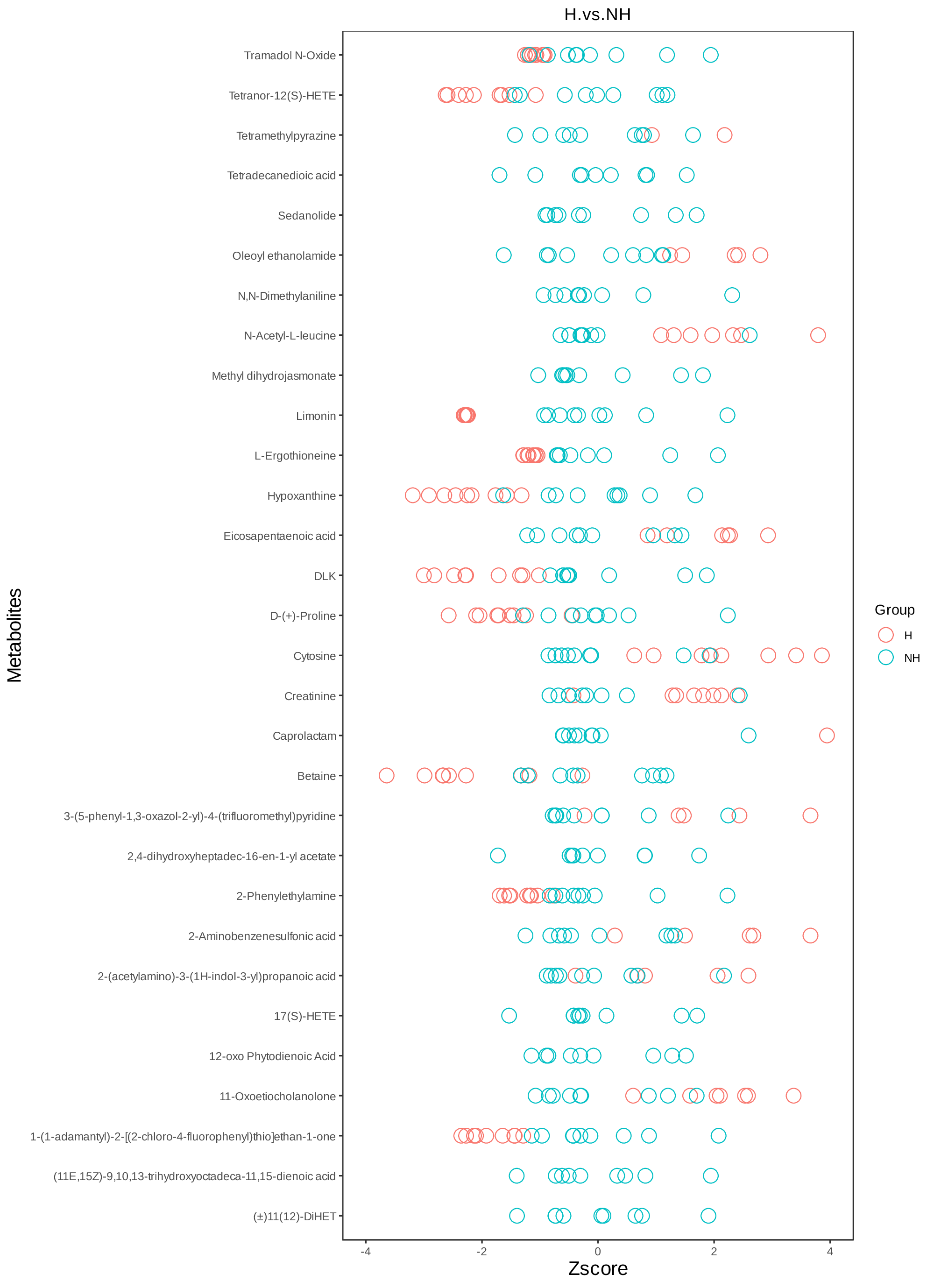

Supplement: Supplemental Information 2 [file peerj-11-15112-s002.zip › peerj-75361-Raw_data_result/Raw data/Result-X101SC21103966-Z01-J001-B1-42/4.MetDiffAnalysis/H.vs.NH/H.vs.NH_pos_zscore.png]

# H.vs.NH

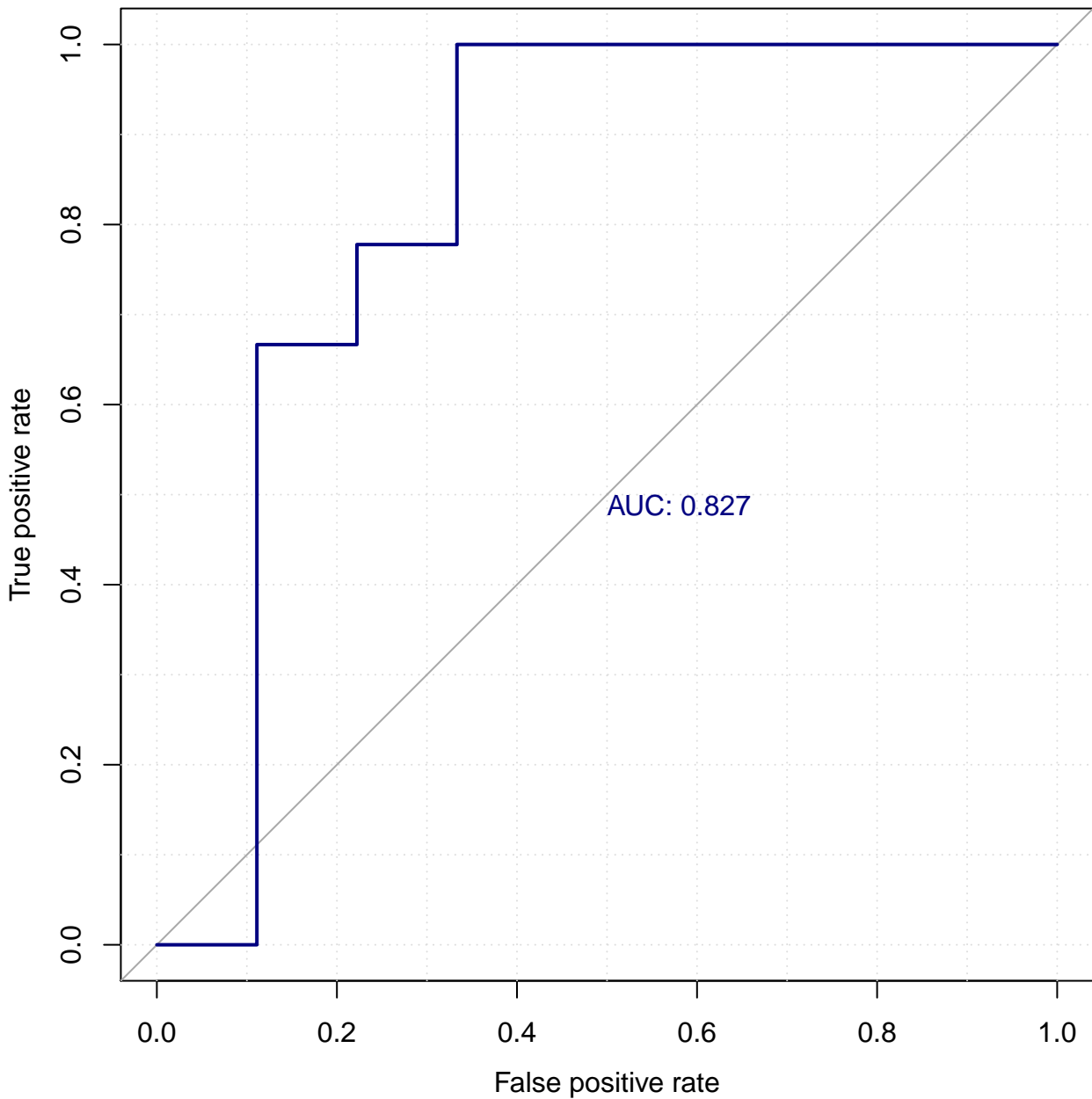

Supplement: Supplemental Information 2 [file peerj-11-15112-s002.zip › peerj-75361-Raw_data_result/Raw data/Result-X101SC21103966-Z01-J001-B1-42/4.MetDiffAnalysis/H.vs.NH/ROC_neg/Com_1061_neg_ROC.pdf]

H.vs.NH

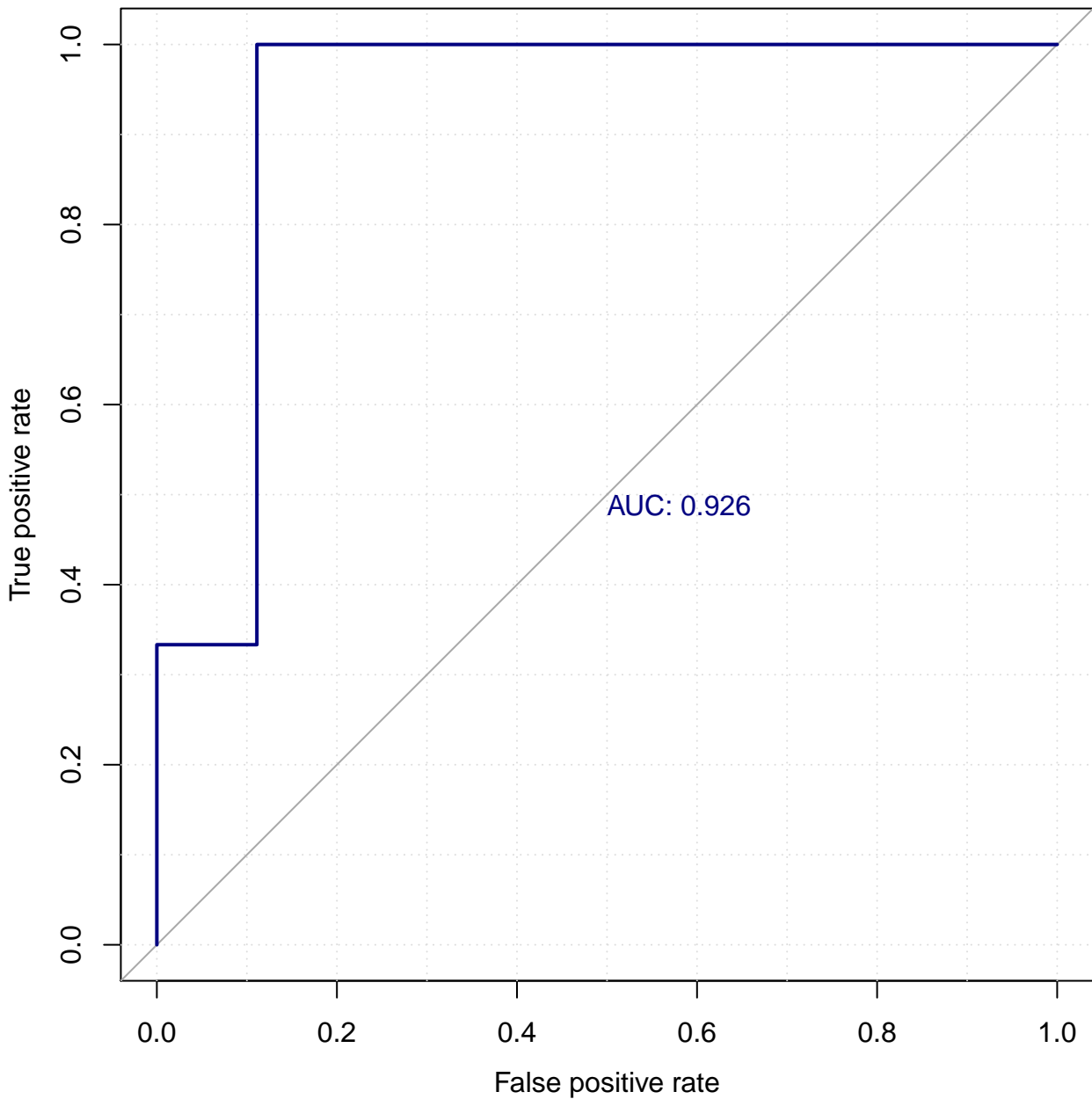

Supplement: Supplemental Information 2 [file peerj-11-15112-s002.zip › peerj-75361-Raw_data_result/Raw data/Result-X101SC21103966-Z01-J001-B1-42/4.MetDiffAnalysis/H.vs.NH/ROC_neg/Com_1556_neg_ROC.pdf]

# H.vs.NH

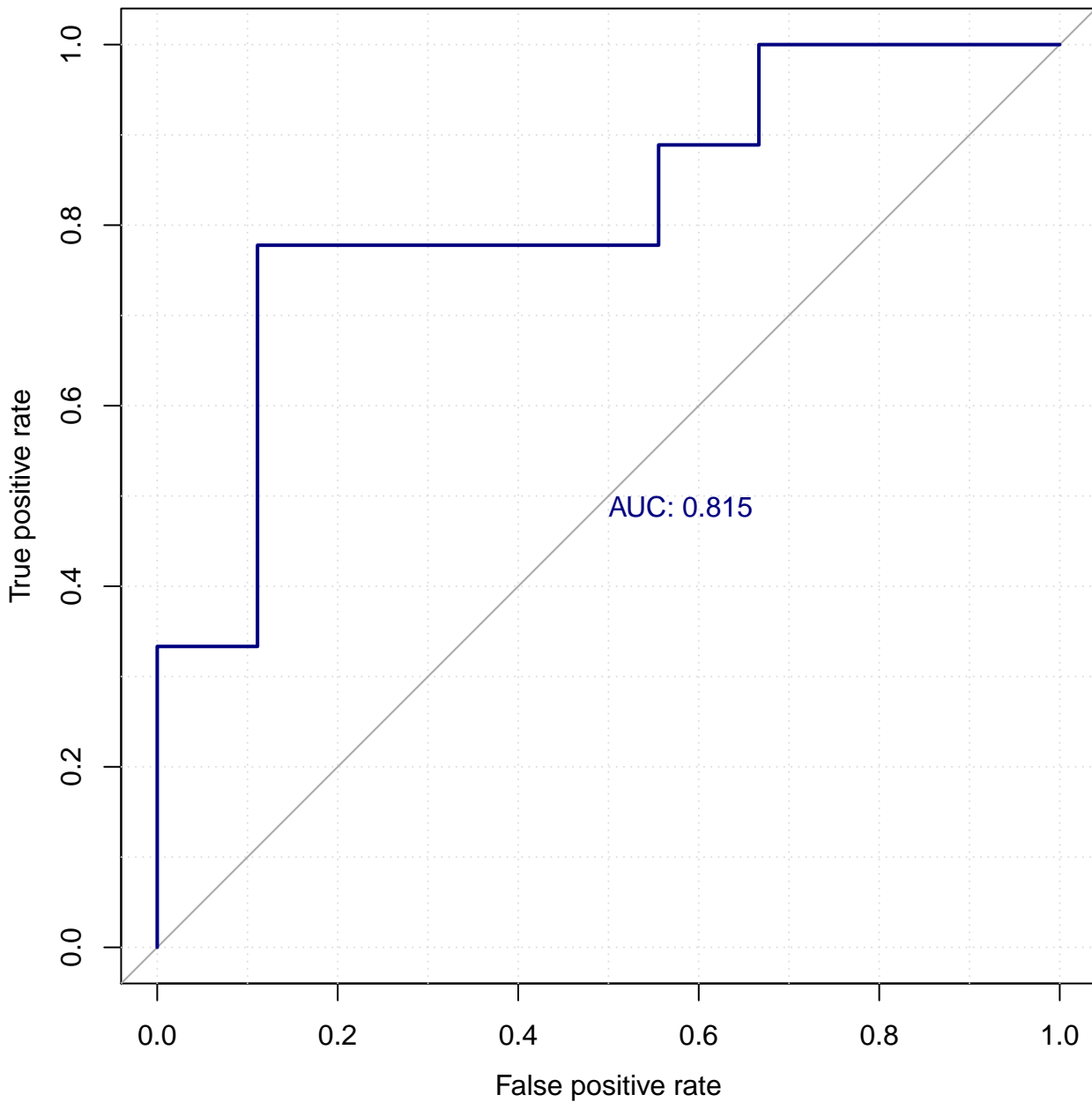

Supplement: Supplemental Information 2 [file peerj-11-15112-s002.zip › peerj-75361-Raw_data_result/Raw data/Result-X101SC21103966-Z01-J001-B1-42/4.MetDiffAnalysis/H.vs.NH/ROC_neg/Com_1570_neg_ROC.pdf]

# H.vs.NH

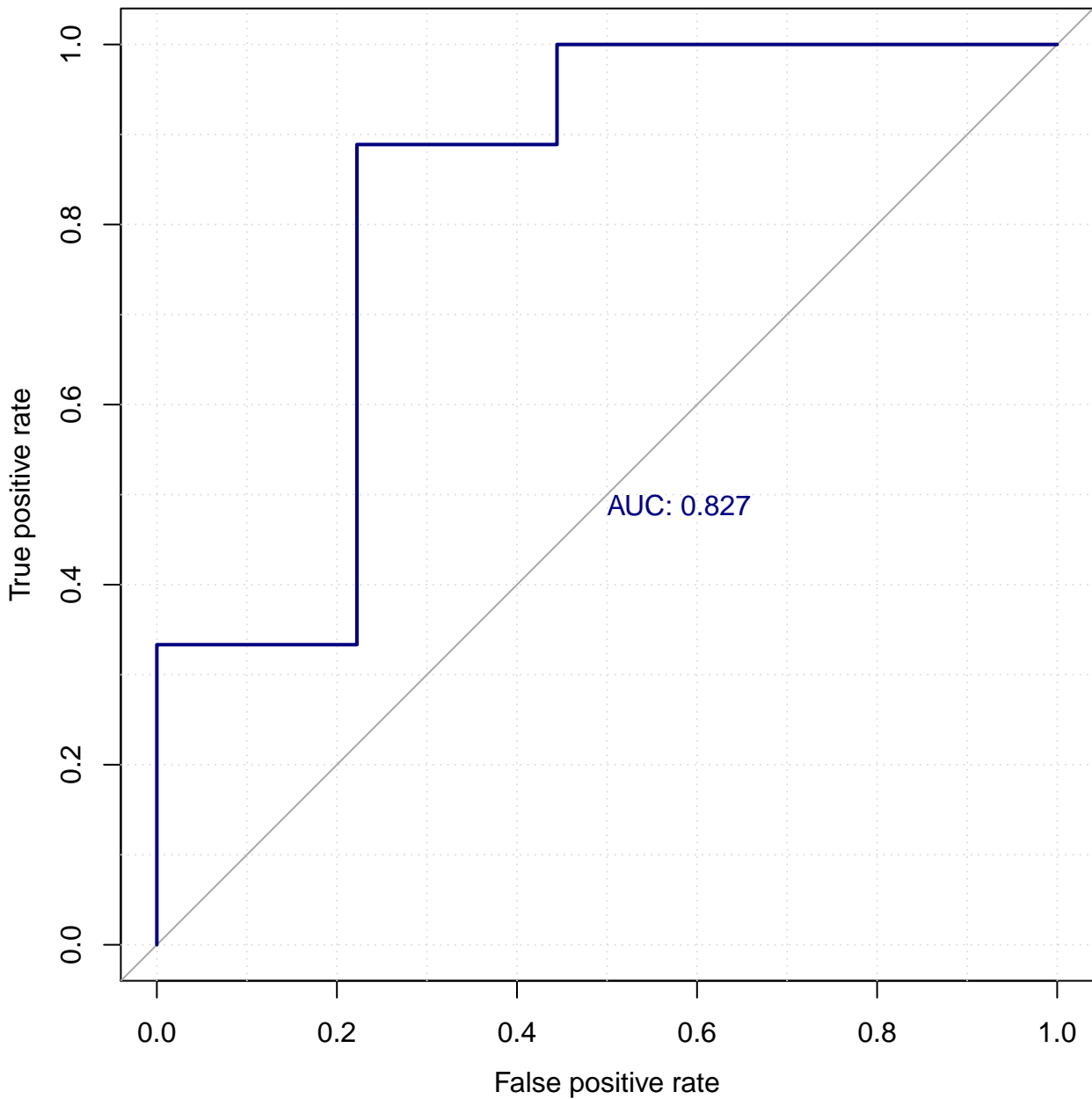

Supplement: Supplemental Information 2 [file peerj-11-15112-s002.zip › peerj-75361-Raw_data_result/Raw data/Result-X101SC21103966-Z01-J001-B1-42/4.MetDiffAnalysis/H.vs.NH/ROC_neg/Com_157_neg_ROC.pdf]

# H.vs.NH

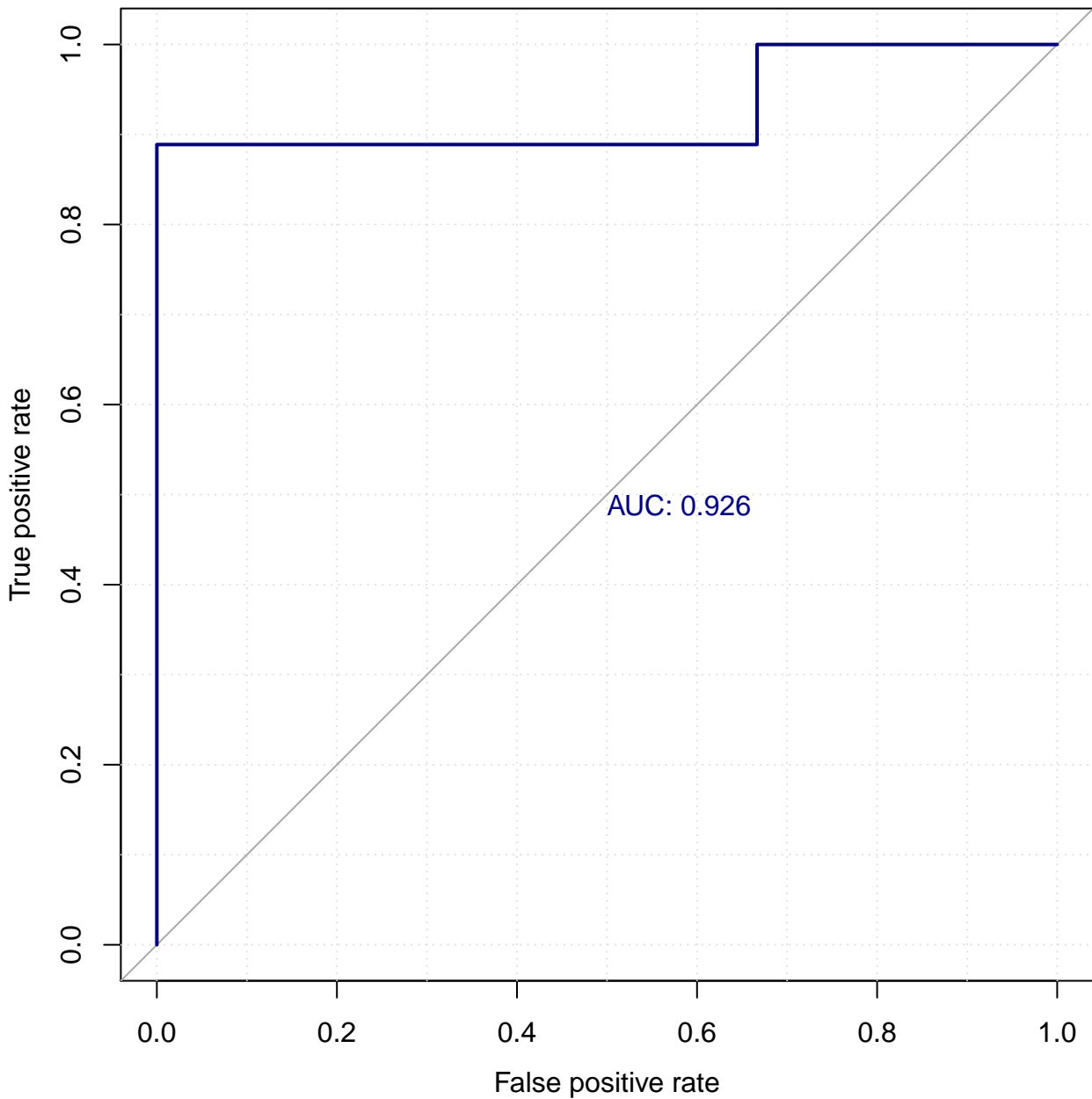

Supplement: Supplemental Information 2 [file peerj-11-15112-s002.zip › peerj-75361-Raw_data_result/Raw data/Result-X101SC21103966-Z01-J001-B1-42/4.MetDiffAnalysis/H.vs.NH/ROC_neg/Com_1701_neg_ROC.pdf]

H.vs.NH

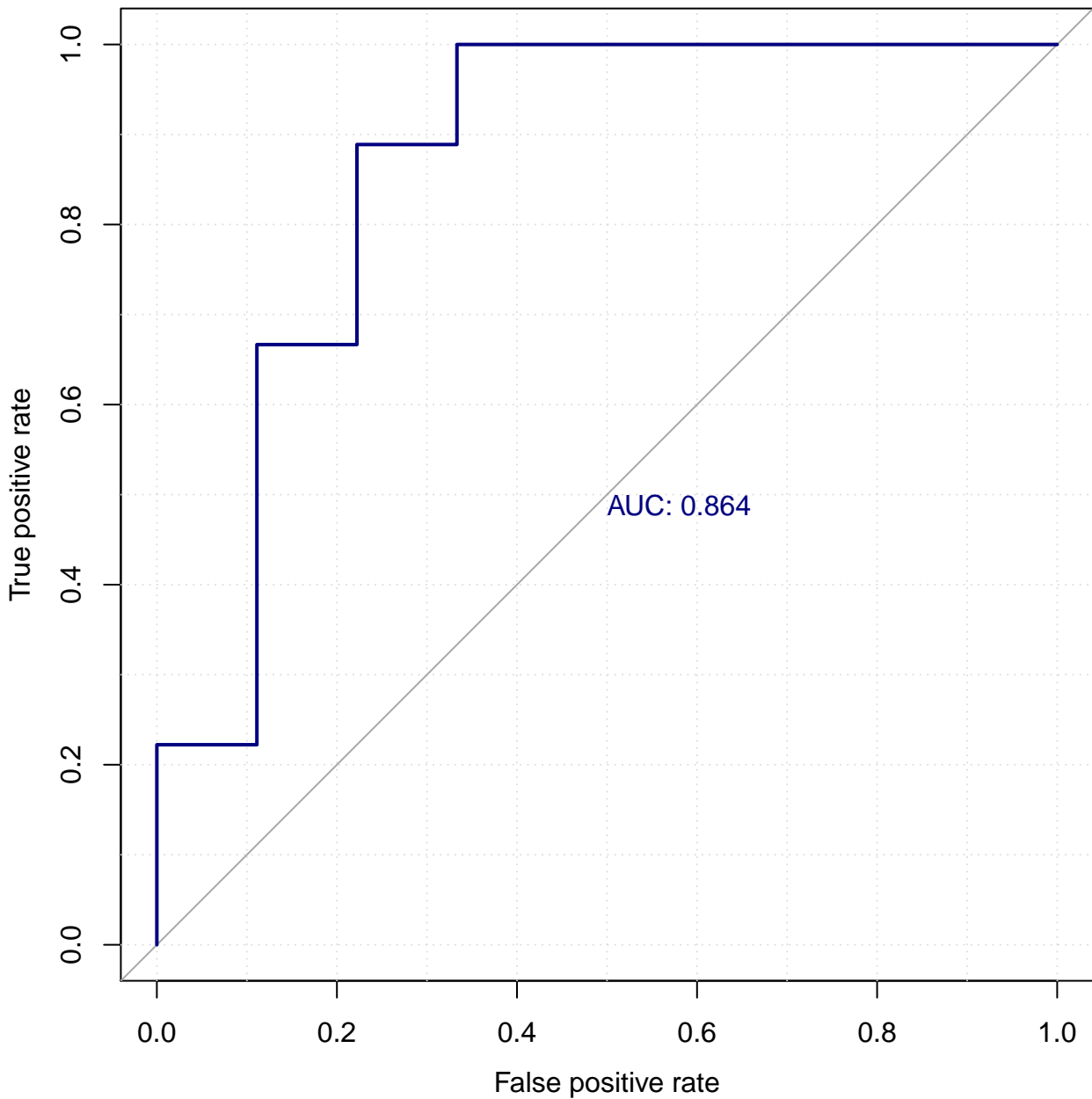

Supplement: Supplemental Information 2 [file peerj-11-15112-s002.zip › peerj-75361-Raw_data_result/Raw data/Result-X101SC21103966-Z01-J001-B1-42/4.MetDiffAnalysis/H.vs.NH/ROC_neg/Com_1719_neg_ROC.pdf]

H.vs.NH

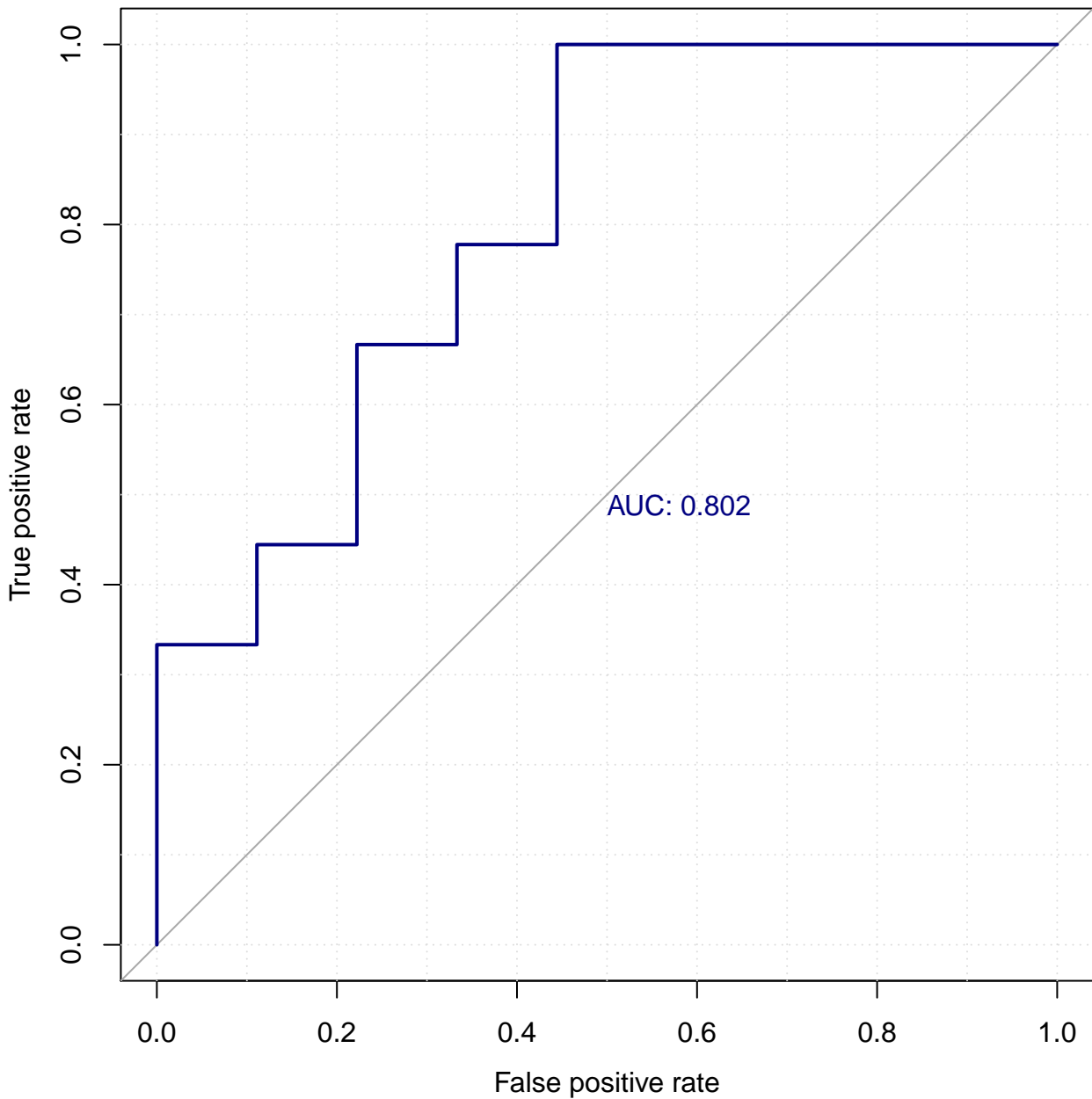

Supplement: Supplemental Information 2 [file peerj-11-15112-s002.zip › peerj-75361-Raw_data_result/Raw data/Result-X101SC21103966-Z01-J001-B1-42/4.MetDiffAnalysis/H.vs.NH/ROC_neg/Com_1870_neg_ROC.pdf]

H.vs.NH

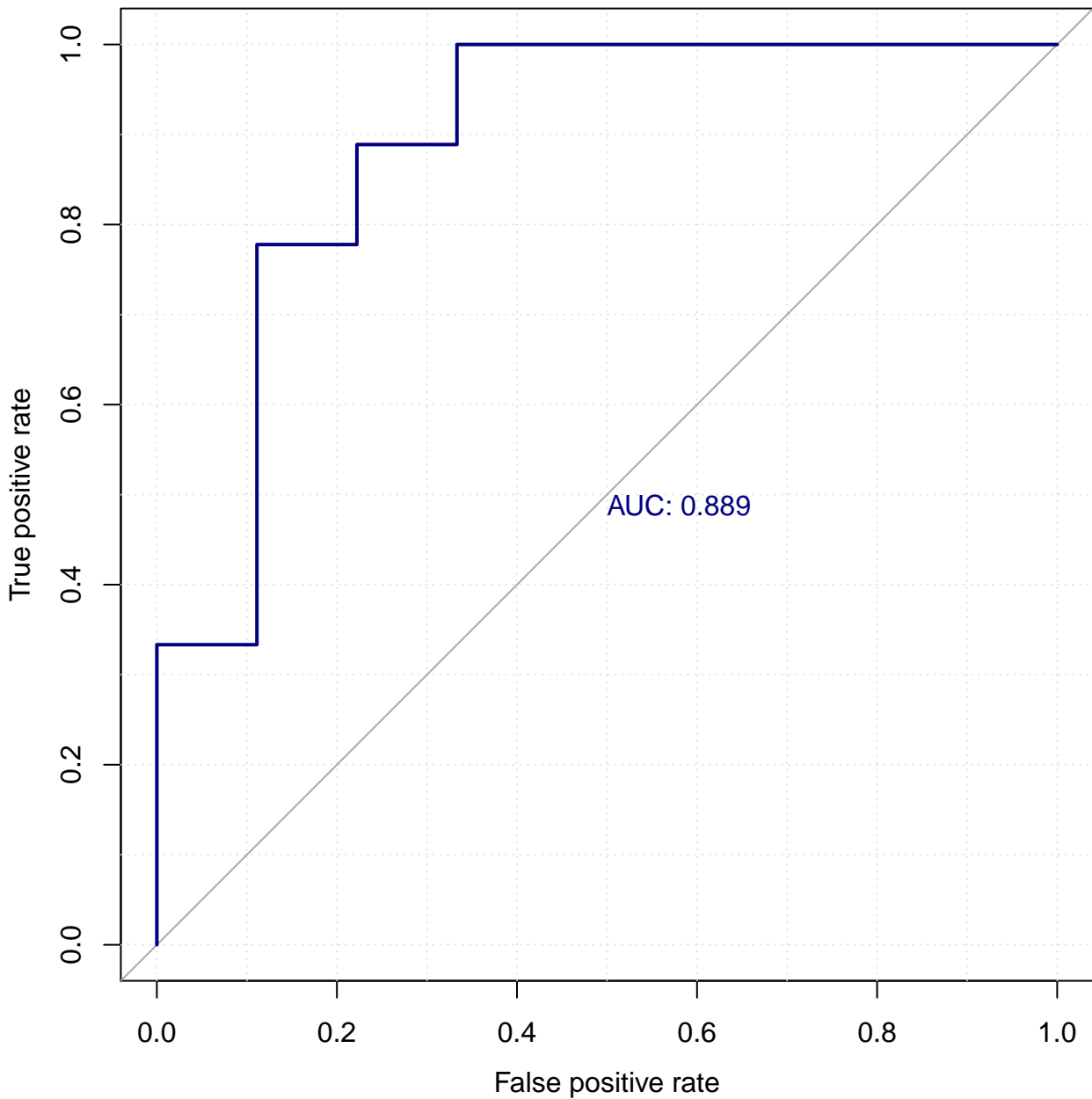

Supplement: Supplemental Information 2 [file peerj-11-15112-s002.zip › peerj-75361-Raw_data_result/Raw data/Result-X101SC21103966-Z01-J001-B1-42/4.MetDiffAnalysis/H.vs.NH/ROC_neg/Com_1951_neg_ROC.pdf]

H.vs.NH

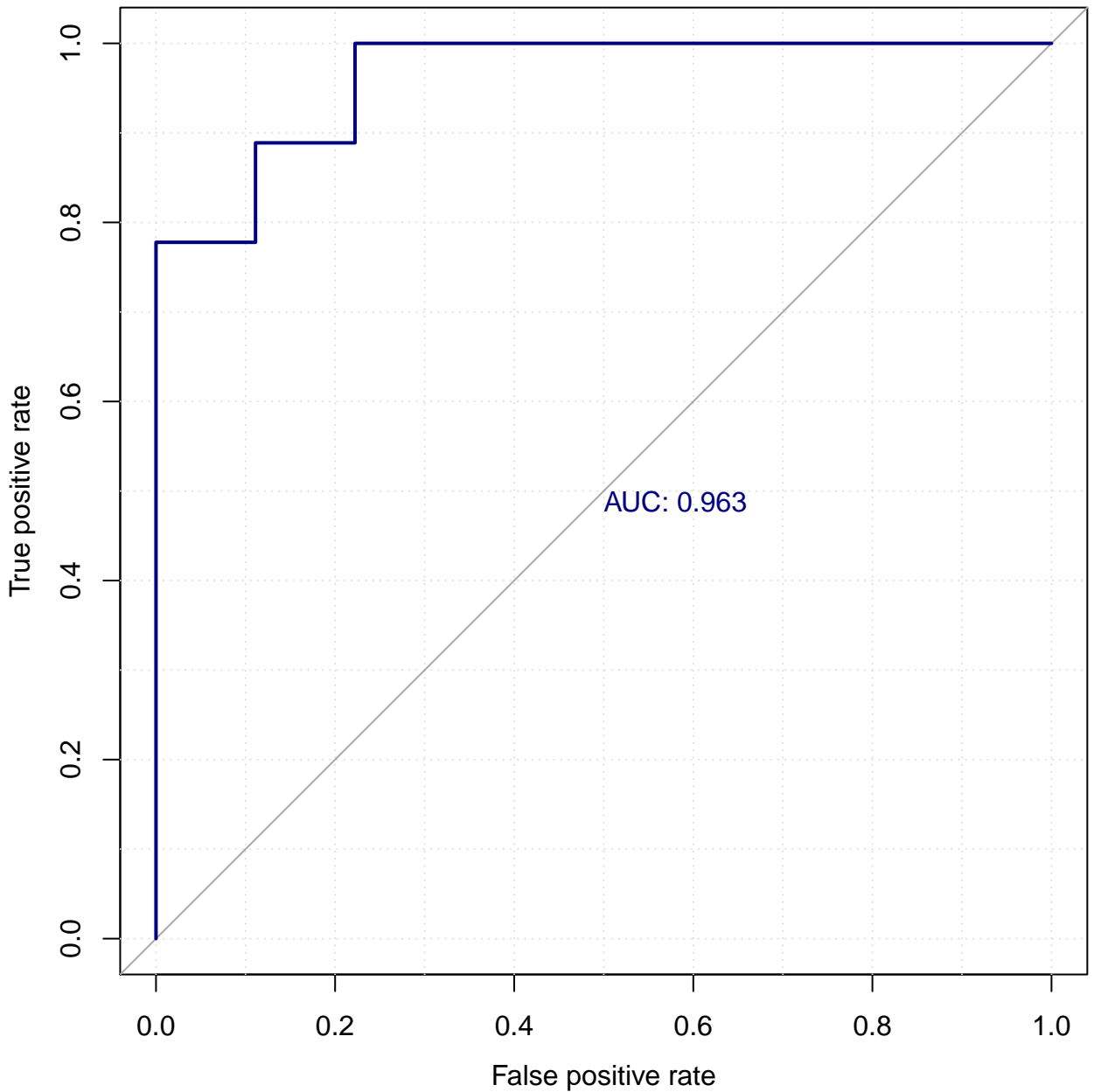

Supplement: Supplemental Information 2 [file peerj-11-15112-s002.zip › peerj-75361-Raw_data_result/Raw data/Result-X101SC21103966-Z01-J001-B1-42/4.MetDiffAnalysis/H.vs.NH/ROC_neg/Com_2001_neg_ROC.pdf]

# H.vs.NH

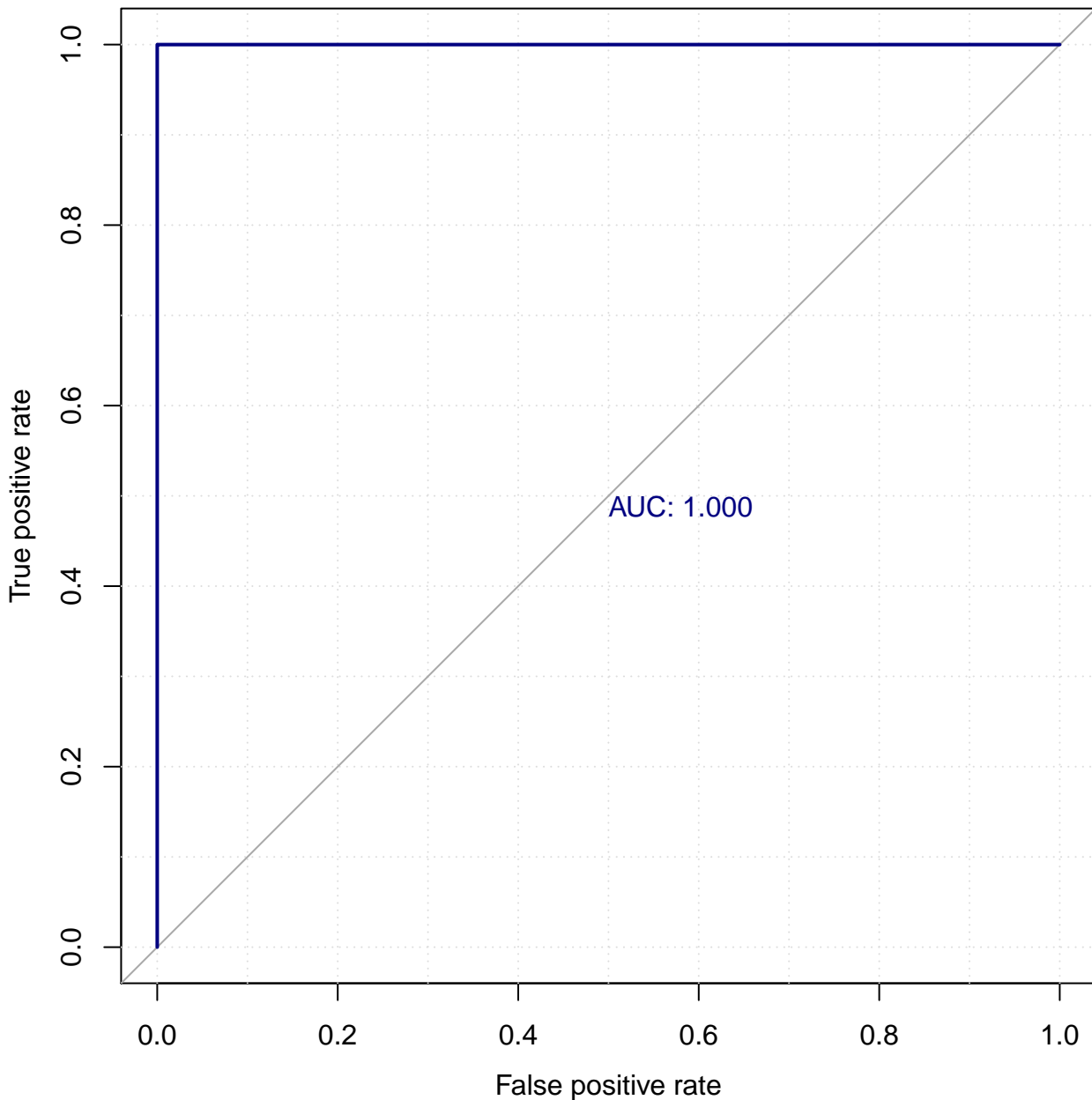

Supplement: Supplemental Information 2 [file peerj-11-15112-s002.zip › peerj-75361-Raw_data_result/Raw data/Result-X101SC21103966-Z01-J001-B1-42/4.MetDiffAnalysis/H.vs.NH/ROC_neg/Com_2167_neg_ROC.pdf]

# H.vs.NH

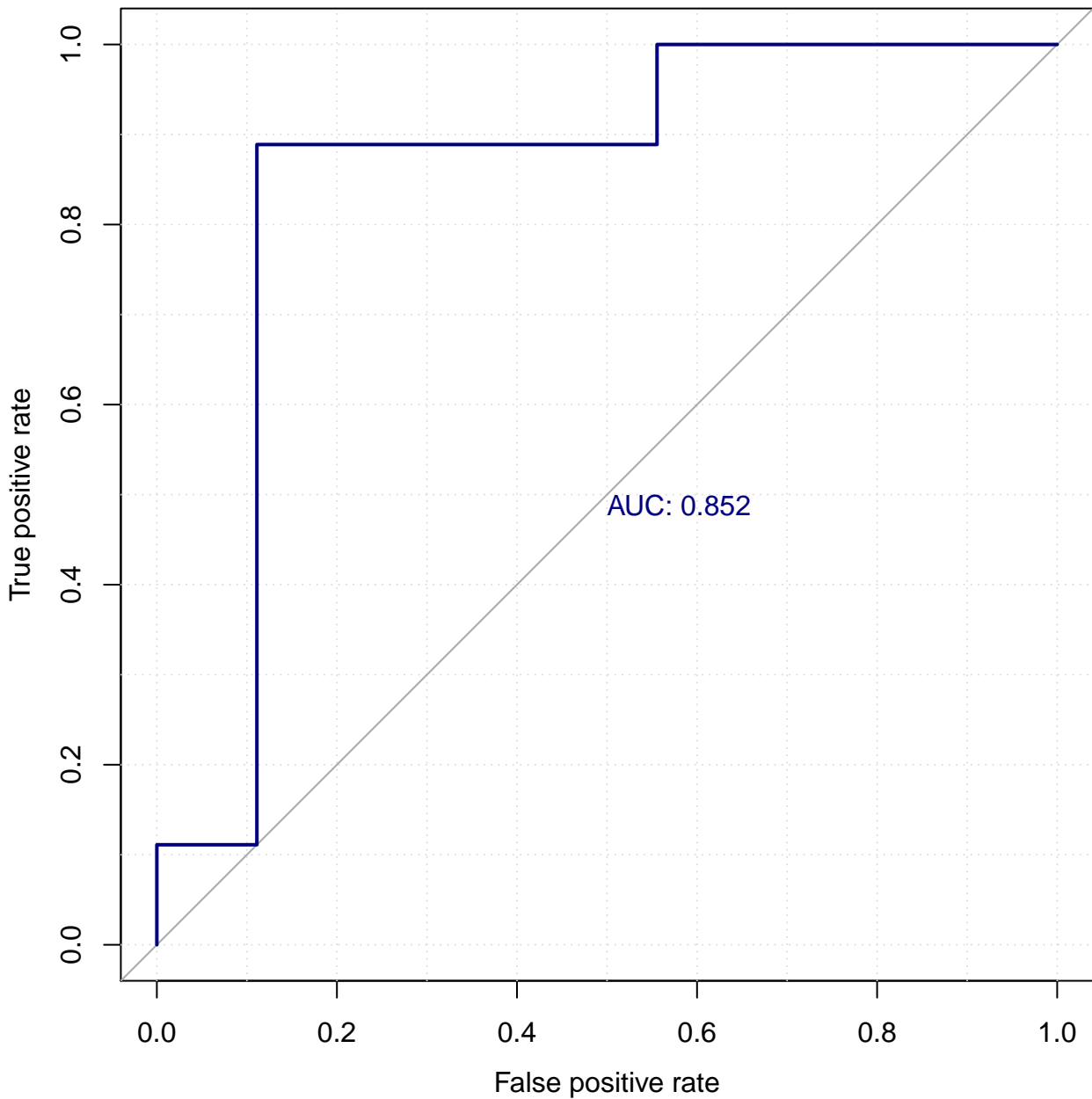

Supplement: Supplemental Information 2 [file peerj-11-15112-s002.zip › peerj-75361-Raw_data_result/Raw data/Result-X101SC21103966-Z01-J001-B1-42/4.MetDiffAnalysis/H.vs.NH/ROC_neg/Com_221_neg_ROC.pdf]

# H.vs.NH

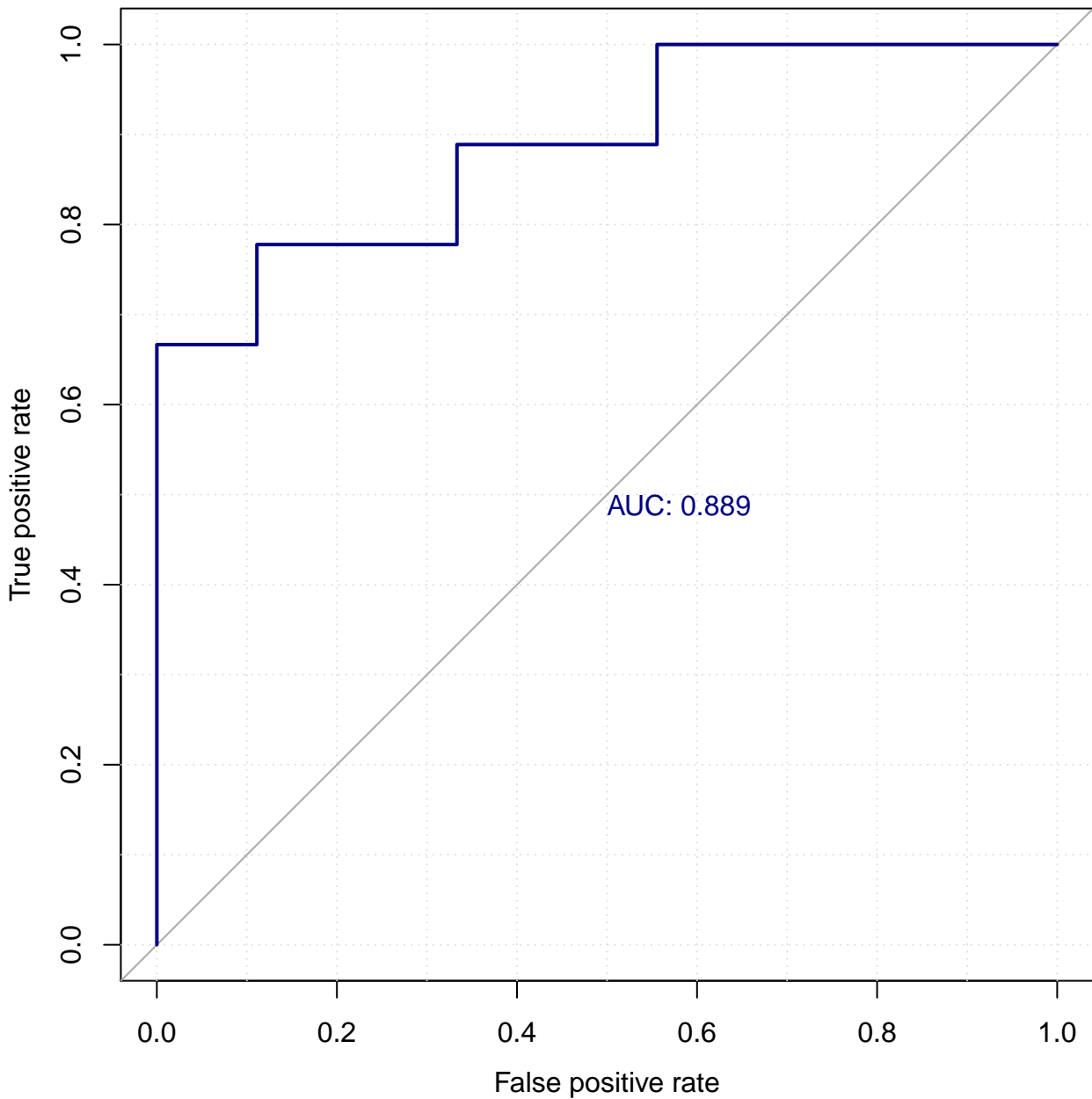

Supplement: Supplemental Information 2 [file peerj-11-15112-s002.zip › peerj-75361-Raw_data_result/Raw data/Result-X101SC21103966-Z01-J001-B1-42/4.MetDiffAnalysis/H.vs.NH/ROC_neg/Com_2253_neg_ROC.pdf]

H.vs.NH

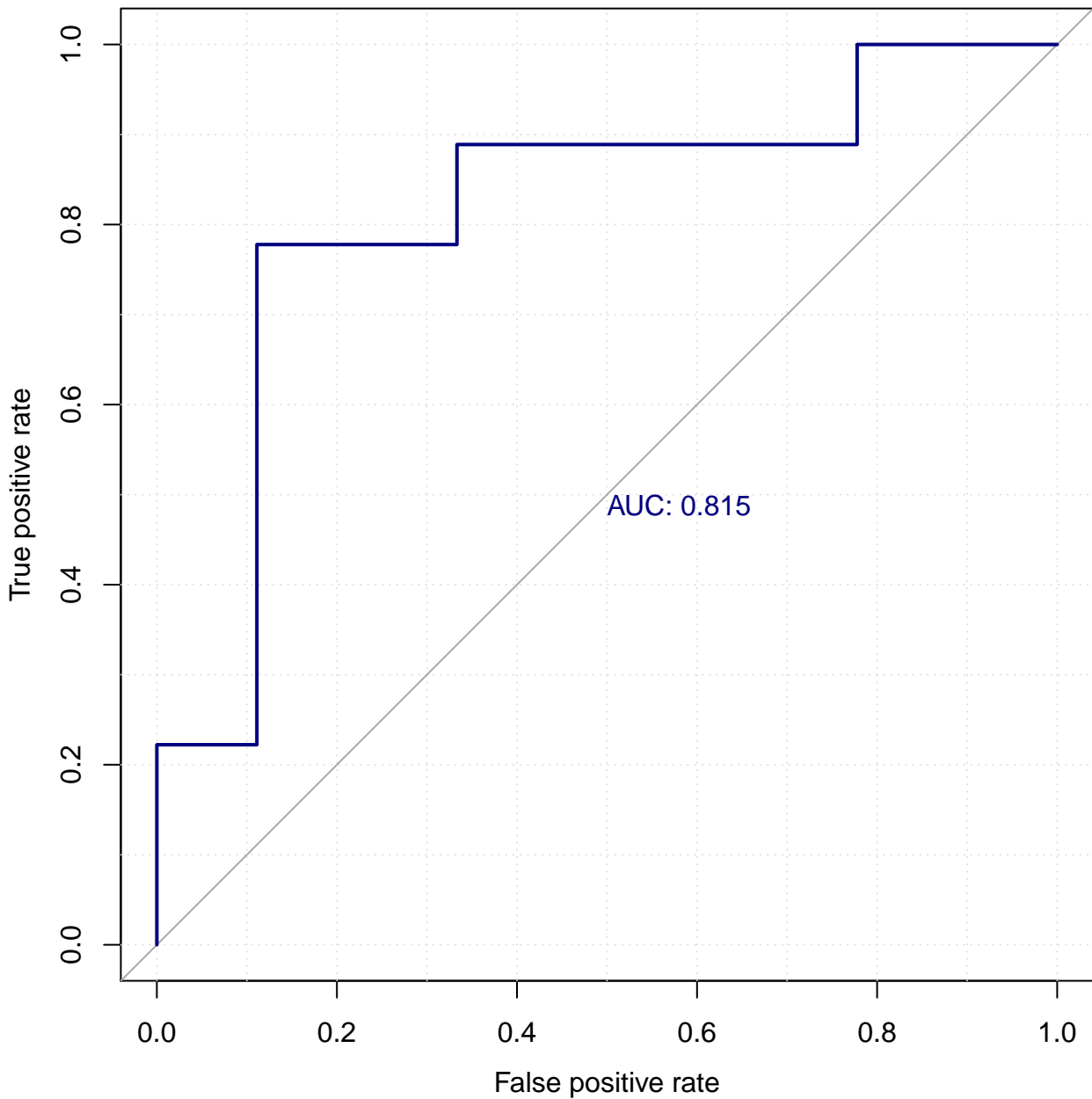

Supplement: Supplemental Information 2 [file peerj-11-15112-s002.zip › peerj-75361-Raw_data_result/Raw data/Result-X101SC21103966-Z01-J001-B1-42/4.MetDiffAnalysis/H.vs.NH/ROC_neg/Com_2318_neg_ROC.pdf]
