# Supplementary material for: Semen quality and seminal plasma metabolites in male rabbits (Oryctolagus cuniculus) under heat stress
Source: PeerJ. 2023 Apr 7;11:e15112. doi: 10.7717/peerj.15112 (PMC10103697; doi:10.7717/peerj.15112)
Supplement: Supplemental Information 2 [file peerj-11-15112-s002.zip › peerj-75361-Raw_data_result/Raw data/Result-X101SC21103966-Z01-J001-B1-42/4.MetDiffAnalysis/H.vs.NH/ROC_pos/Com_5899_pos_ROC.pdf]

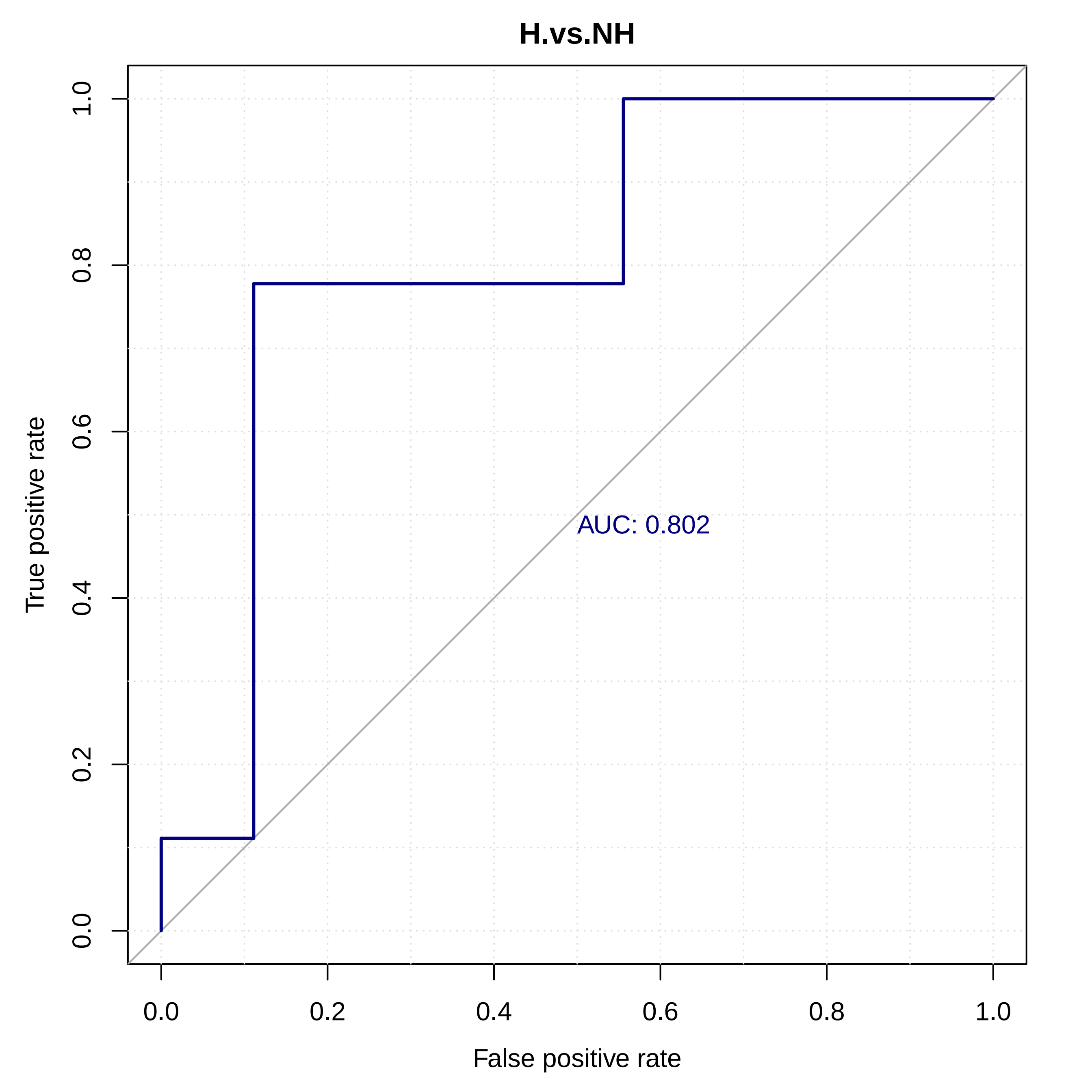
H.vs.NH
True positive rate
False positive rate
0.0
0.2
0.4
0.6
0.8
1.0
AUC: 0.802
