# Supplementary material for: Semen quality and seminal plasma metabolites in male rabbits (Oryctolagus cuniculus) under heat stress
Source: PeerJ. 2023 Apr 7;11:e15112. doi: 10.7717/peerj.15112 (PMC10103697; doi:10.7717/peerj.15112)
Supplement: Supplemental Information 2 [file peerj-11-15112-s002.zip › peerj-75361-Raw_data_result/Raw data/Result-X101SC21103966-Z01-J001-B1-42/5.MetKeggEnrichment/H.vs.NH/H.vs.NH_neg.KEGG_Enrich.scatterplot.pdf]

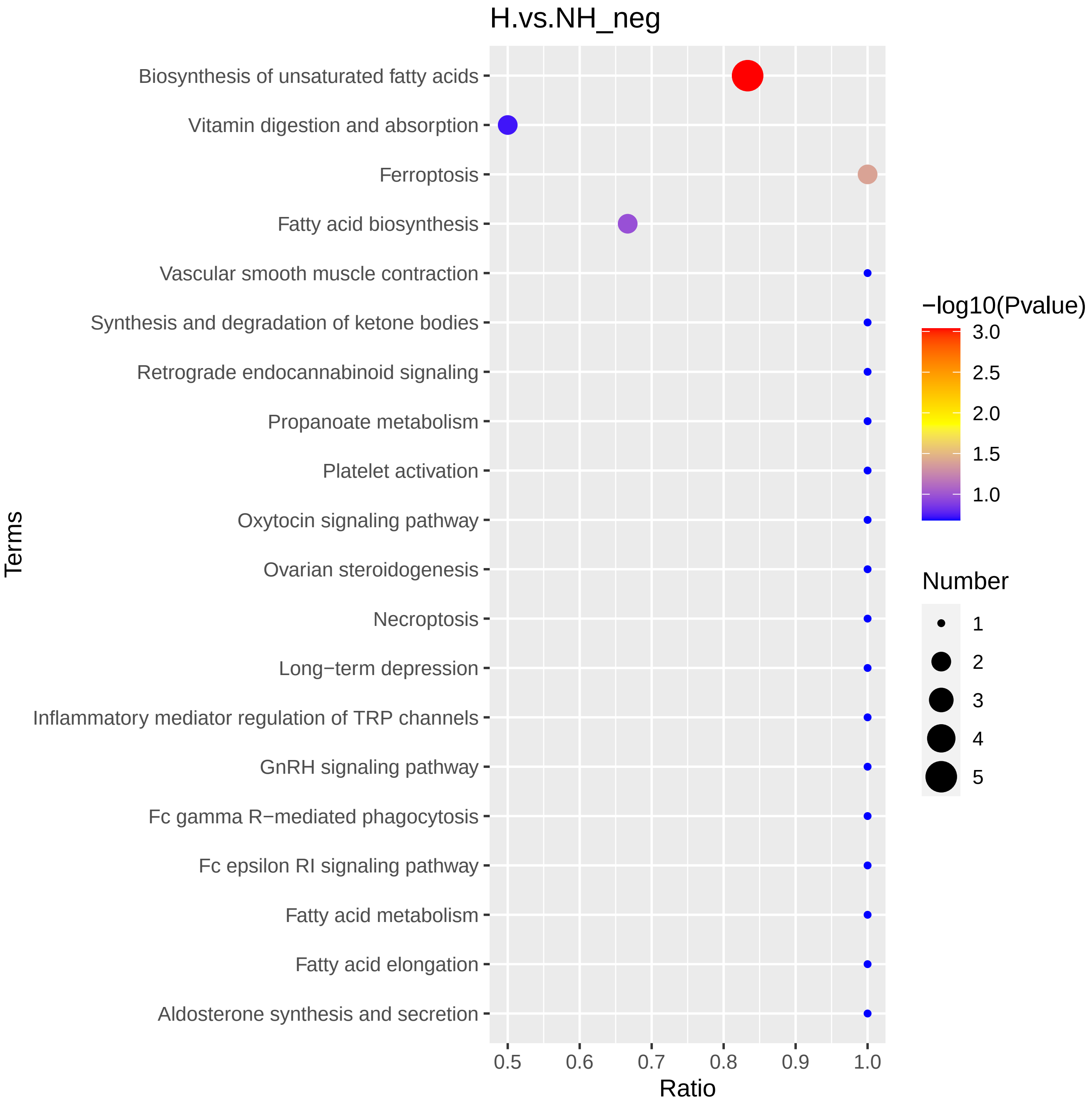

H.vs.NH_neg
Biosynthesis of unsaturated fatty acids
Vitamin digestion and absorption
Ferroptosis
Fatty acid biosynthesis
Vascular smooth muscle contraction
Synthesis and degradation of ketone bodies
Retrograde endocannabinoid signaling
Propanoate metabolism
Platelet activation
Oxytocin signaling pathway
Ovarian steroidogenesis
Necroptosis
Long−term depression
Inflammatory mediator regulation of TRP channels
GnRH signaling pathway
Fc gamma R−mediated phagocytosis
Fc epsilon RI signaling pathway
Fatty acid metabolism
Fatty acid elongation
Aldosterone synthesis and secretion
Terms
0.5
0.6
0.7
0.8
0.9
1.0
Ratio
−log10(Pvalue)
3.0
2.5
2.0
1.5
1.0
Number
1
2
3
4
5
