# Supplementary material for: Semen quality and seminal plasma metabolites in male rabbits (Oryctolagus cuniculus) under heat stress
Source: PeerJ. 2023 Apr 7;11:e15112. doi: 10.7717/peerj.15112 (PMC10103697; doi:10.7717/peerj.15112)
Supplement: Supplemental Information 2 [file peerj-11-15112-s002.zip › peerj-75361-Raw_data_result/Raw data/Result-X101SC21103966-Z01-J001-B1-42/5.MetKeggEnrichment/H.vs.NH/H.vs.NH_neg_KEGG_map/KEGG.map.html]

Pathway Enrichment


  

# The most enriched pathway terms of H.vs.NH\_neg

Statistic method: hypergeometric test

FDR correction method: Benjamini and Hochberg

| MapID | MapTitle | Pvalue | AdjustedPv | x | y | n | N | EnrichDirect | GeneIDs |
| --- | --- | --- | --- | --- | --- | --- | --- | --- | --- |
| map01040 | Biosynthesis of unsaturated fatty acids | 0.000922931923171624 | 0.0415319365427231 | 5 | 6 | 12 | 58 | Over | Com\_2167\_neg Com\_232\_neg Com\_256\_neg Com\_549\_neg Com\_8194\_neg |
| map04216 | Ferroptosis | 0.0399274047186933 | 0.46551724137931 | 2 | 2 | 12 | 58 | Over | Com\_256\_neg Com\_549\_neg |
| map00061 | Fatty acid biosynthesis | 0.105522426756547 | 0.46551724137931 | 2 | 3 | 12 | 58 | Over | Com\_2167\_neg Com\_232\_neg |
| map04977 | Vitamin digestion and absorption | 0.186025408348457 | 0.46551724137931 | 2 | 4 | 12 | 58 | Over | Com\_4206\_neg Com\_87\_neg |
| map00062 | Fatty acid elongation | 0.206896551724138 | 0.46551724137931 | 1 | 1 | 12 | 58 | Over | Com\_232\_neg |
| map00072 | Synthesis and degradation of ketone bodies | 0.206896551724138 | 0.46551724137931 | 1 | 1 | 12 | 58 | Over | Com\_383\_neg |
| map00640 | Propanoate metabolism | 0.206896551724138 | 0.46551724137931 | 1 | 1 | 12 | 58 | Over | Com\_383\_neg |
| map01212 | Fatty acid metabolism | 0.206896551724138 | 0.46551724137931 | 1 | 1 | 12 | 58 | Over | Com\_232\_neg |
| map04217 | Necroptosis | 0.206896551724138 | 0.46551724137931 | 1 | 1 | 12 | 58 | Over | Com\_256\_neg |
| map04270 | Vascular smooth muscle contraction | 0.206896551724138 | 0.46551724137931 | 1 | 1 | 12 | 58 | Over | Com\_256\_neg |
| map04611 | Platelet activation | 0.206896551724138 | 0.46551724137931 | 1 | 1 | 12 | 58 | Over | Com\_256\_neg |
| map04664 | Fc epsilon RI signaling pathway | 0.206896551724138 | 0.46551724137931 | 1 | 1 | 12 | 58 | Over | Com\_256\_neg |
| map04666 | Fc gamma R-mediated phagocytosis | 0.206896551724138 | 0.46551724137931 | 1 | 1 | 12 | 58 | Over | Com\_256\_neg |
| map04723 | Retrograde endocannabinoid signaling | 0.206896551724138 | 0.46551724137931 | 1 | 1 | 12 | 58 | Over | Com\_256\_neg |
| map04730 | Long-term depression | 0.206896551724138 | 0.46551724137931 | 1 | 1 | 12 | 58 | Over | Com\_256\_neg |
| map04750 | Inflammatory mediator regulation of TRP channels | 0.206896551724138 | 0.46551724137931 | 1 | 1 | 12 | 58 | Over | Com\_256\_neg |
| map04912 | GnRH signaling pathway | 0.206896551724138 | 0.46551724137931 | 1 | 1 | 12 | 58 | Over | Com\_256\_neg |
| map04913 | Ovarian steroidogenesis | 0.206896551724138 | 0.46551724137931 | 1 | 1 | 12 | 58 | Over | Com\_256\_neg |
| map04921 | Oxytocin signaling pathway | 0.206896551724138 | 0.46551724137931 | 1 | 1 | 12 | 58 | Over | Com\_256\_neg |
| map04925 | Aldosterone synthesis and secretion | 0.206896551724138 | 0.46551724137931 | 1 | 1 | 12 | 58 | Over | Com\_256\_neg |
| map00071 | Fatty acid degradation | 0.373865698729582 | 0.509816861903976 | 1 | 2 | 12 | 58 | Over | Com\_232\_neg |
| map00120 | Primary bile acid biosynthesis | 0.373865698729582 | 0.509816861903976 | 1 | 2 | 12 | 58 | Over | Com\_34\_neg |
| map00280 | Valine, leucine and isoleucine degradation | 0.373865698729582 | 0.509816861903976 | 1 | 2 | 12 | 58 | Over | Com\_383\_neg |
| map00310 | Lysine degradation | 0.373865698729582 | 0.509816861903976 | 1 | 2 | 12 | 58 | Over | Com\_383\_neg |
| map00590 | Arachidonic acid metabolism | 0.373865698729582 | 0.509816861903976 | 1 | 2 | 12 | 58 | Over | Com\_256\_neg |
| map00591 | Linoleic acid metabolism | 0.373865698729582 | 0.509816861903976 | 1 | 2 | 12 | 58 | Over | Com\_256\_neg |
| map00620 | Pyruvate metabolism | 0.373865698729582 | 0.509816861903976 | 1 | 2 | 12 | 58 | Over | Com\_325\_neg |
| map00740 | Riboflavin metabolism | 0.373865698729582 | 0.509816861903976 | 1 | 2 | 12 | 58 | Over | Com\_4206\_neg |
| map00920 | Sulfur metabolism | 0.373865698729582 | 0.509816861903976 | 1 | 2 | 12 | 58 | Over | Com\_34\_neg |
| map04066 | HIF-1 signaling pathway | 0.373865698729582 | 0.509816861903976 | 1 | 2 | 12 | 58 | Over | Com\_87\_neg |
| map04080 | Neuroactive ligand-receptor interaction | 0.373865698729582 | 0.509816861903976 | 1 | 2 | 12 | 58 | Over | Com\_34\_neg |
| map04726 | Serotonergic synapse | 0.373865698729582 | 0.509816861903976 | 1 | 2 | 12 | 58 | Over | Com\_256\_neg |
| map04923 | Regulation of lipolysis in adipocytes | 0.373865698729582 | 0.509816861903976 | 1 | 2 | 12 | 58 | Over | Com\_256\_neg |
| map00240 | Pyrimidine metabolism | 0.508037334716101 | 0.617883244924987 | 1 | 3 | 12 | 58 | Over | Com\_1719\_neg |
| map00350 | Tyrosine metabolism | 0.508037334716101 | 0.617883244924987 | 1 | 3 | 12 | 58 | Over | Com\_383\_neg |
| map00430 | Taurine and hypotaurine metabolism | 0.508037334716101 | 0.617883244924987 | 1 | 3 | 12 | 58 | Over | Com\_34\_neg |
| map00480 | Glutathione metabolism | 0.508037334716101 | 0.617883244924987 | 1 | 3 | 12 | 58 | Over | Com\_87\_neg |
| map00053 | Ascorbate and aldarate metabolism | 1 | 1 | 1 | 4 | 12 | 58 | Over | Com\_87\_neg |
| map00650 | Butanoate metabolism | 1 | 1 | 1 | 4 | 12 | 58 | Over | Com\_383\_neg |
| map02010 | ABC transporters | 1 | 1 | 1 | 4 | 12 | 58 | Over | Com\_34\_neg |
