# Supplementary material for: Semen quality and seminal plasma metabolites in male rabbits (Oryctolagus cuniculus) under heat stress
Source: PeerJ. 2023 Apr 7;11:e15112. doi: 10.7717/peerj.15112 (PMC10103697; doi:10.7717/peerj.15112)
Supplement: Supplemental Information 2 [file peerj-11-15112-s002.zip › peerj-75361-Raw_data_result/Raw data/Result-X101SC21103966-Z01-J001-B1-42/5.MetKeggEnrichment/H.vs.NH/H.vs.NH_pos.KEGG_Enrich.scatterplot.pdf]

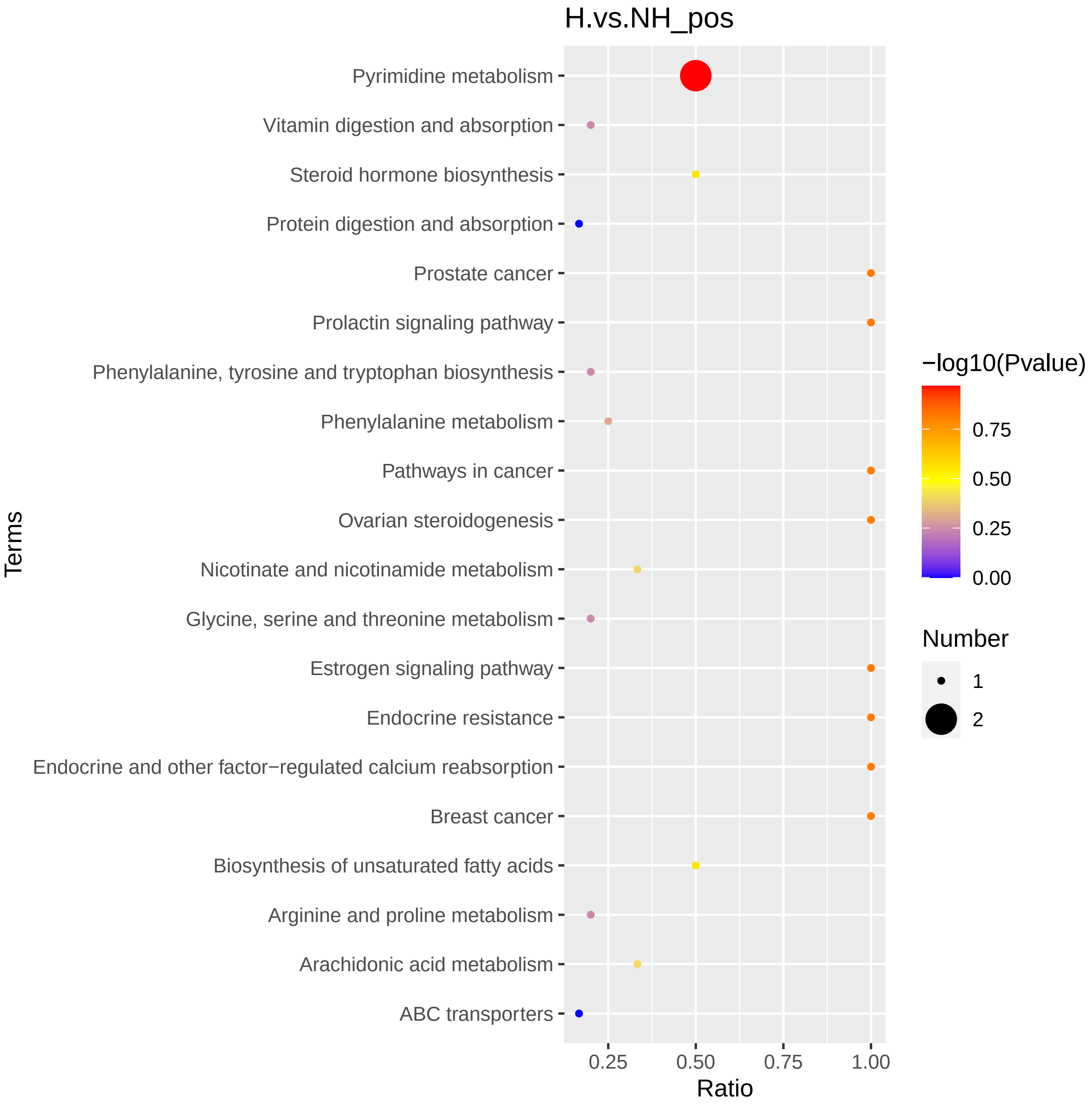
H.vs.NH_pos
Pyrimidine metabolism
Vitamin digestion and absorption
Steroid hormone biosynthesis
Protein digestion and absorption
Prostate cancer
Prolactin signaling pathway
Phenylalanine, tyrosine and tryptophan biosynthesis
Phenylalanine metabolism
Pathways in cancer
Ovarian steroidogenesis
Nicotinate and nicotinamide metabolism
Glycine, serine and threonine metabolism
Estrogen signaling pathway
Endocrine resistance
Endocrine and other factor−regulated calcium reabsorption
Breast cancer
Biosynthesis of unsaturated fatty acids
Arginine and proline metabolism
Arachidonic acid metabolism
ABC transporters
Terms
0.25
0.50
0.75
1.00
Ratio
−log10(Pvalue)
0.75
0.50
0.25
0.00
Number
1
2
