# Supplementary material for: Semen quality and seminal plasma metabolites in male rabbits (Oryctolagus cuniculus) under heat stress
Source: PeerJ. 2023 Apr 7;11:e15112. doi: 10.7717/peerj.15112 (PMC10103697; doi:10.7717/peerj.15112)
Supplement: Supplemental Information 2 [file peerj-11-15112-s002.zip › peerj-75361-Raw_data_result/Raw data/Result-X101SC21103966-Z01-J001-B1-42/5.MetKeggEnrichment/H.vs.NH/H.vs.NH_pos_KEGG_map/KEGG.map.html]

Pathway Enrichment


  

# The most enriched pathway terms of H.vs.NH\_pos

Statistic method: hypergeometric test

FDR correction method: Benjamini and Hochberg

| MapID | MapTitle | Pvalue | AdjustedPv | x | y | n | N | EnrichDirect | GeneIDs |
| --- | --- | --- | --- | --- | --- | --- | --- | --- | --- |
| map00240 | Pyrimidine metabolism | 0.107937479952177 | 0.390432098765432 | 2 | 4 | 11 | 72 | Over | Com\_1138\_pos Com\_1664\_pos |
| map01522 | Endocrine resistance | 0.152777777777778 | 0.390432098765432 | 1 | 1 | 11 | 72 | Over | Com\_4952\_pos |
| map04913 | Ovarian steroidogenesis | 0.152777777777778 | 0.390432098765432 | 1 | 1 | 11 | 72 | Over | Com\_4952\_pos |
| map04915 | Estrogen signaling pathway | 0.152777777777778 | 0.390432098765432 | 1 | 1 | 11 | 72 | Over | Com\_4952\_pos |
| map04917 | Prolactin signaling pathway | 0.152777777777778 | 0.390432098765432 | 1 | 1 | 11 | 72 | Over | Com\_4952\_pos |
| map04961 | Endocrine and other factor-regulated calcium reabsorption | 0.152777777777778 | 0.390432098765432 | 1 | 1 | 11 | 72 | Over | Com\_4952\_pos |
| map05200 | Pathways in cancer | 0.152777777777778 | 0.390432098765432 | 1 | 1 | 11 | 72 | Over | Com\_4952\_pos |
| map05215 | Prostate cancer | 0.152777777777778 | 0.390432098765432 | 1 | 1 | 11 | 72 | Over | Com\_4952\_pos |
| map05224 | Breast cancer | 0.152777777777778 | 0.390432098765432 | 1 | 1 | 11 | 72 | Over | Com\_4952\_pos |
| map00140 | Steroid hormone biosynthesis | 0.284037558685446 | 0.593896713615023 | 1 | 2 | 11 | 72 | Over | Com\_4952\_pos |
| map01040 | Biosynthesis of unsaturated fatty acids | 0.284037558685446 | 0.593896713615023 | 1 | 2 | 11 | 72 | Over | Com\_1621\_pos |
| map00590 | Arachidonic acid metabolism | 0.39654594232059 | 0.701581282567198 | 1 | 3 | 11 | 72 | Over | Com\_6244\_pos |
| map00760 | Nicotinate and nicotinamide metabolism | 0.39654594232059 | 0.701581282567198 | 1 | 3 | 11 | 72 | Over | Com\_261\_pos |
| map00360 | Phenylalanine metabolism | 0.492748763110061 | 0.734471918657917 | 1 | 4 | 11 | 72 | Over | Com\_4747\_pos |
| map00260 | Glycine, serine and threonine metabolism | 0.574804110254022 | 0.734471918657917 | 1 | 5 | 11 | 72 | Over | Com\_7\_pos |
| map00330 | Arginine and proline metabolism | 0.574804110254022 | 0.734471918657917 | 1 | 5 | 11 | 72 | Over | Com\_447\_pos |
| map00400 | Phenylalanine, tyrosine and tryptophan biosynthesis | 0.574804110254022 | 0.734471918657917 | 1 | 5 | 11 | 72 | Over | Com\_358\_pos |
| map04977 | Vitamin digestion and absorption | 0.574804110254022 | 0.734471918657917 | 1 | 5 | 11 | 72 | Over | Com\_261\_pos |
| map02010 | ABC transporters | 1 | 1 | 1 | 6 | 11 | 72 | Over | Com\_7\_pos |
| map04974 | Protein digestion and absorption | 1 | 1 | 1 | 6 | 11 | 72 | Over | Com\_358\_pos |
