# Supplementary material for: Semen quality and seminal plasma metabolites in male rabbits (Oryctolagus cuniculus) under heat stress
Source: PeerJ. 2023 Apr 7;11:e15112. doi: 10.7717/peerj.15112 (PMC10103697; doi:10.7717/peerj.15112)
Supplement: Supplemental Information 2 [file peerj-11-15112-s002.zip › peerj-75361-Raw_data_result/Raw data/Result-X101SC21103966-Z01-J001-B1-42/5.MetKeggEnrichment/H.vs.NH/H.vs.NH_pos_KEGG_map/src/04810.html]

map04810


Close
